# Identification of a Pair of Linear or Cyclic Naturally Inspired Bifunctional Lipopeptide Antibiotics That Overcome Antimicrobial Resistance

**DOI:** 10.1002/advs.202509796

**Published:** 2025-09-11

**Authors:** Lei Li, Yiwen Zhou, Yuzhu Wu

**Affiliations:** ^1^ State Key Laboratory of Microbial Metabolism and School of Life Sciences and Biotechnology Shanghai Jiao Tong University 800 Dongchuan Rd. Shanghai 200240 China

**Keywords:** antimicrobial resistance, lipopeptides, mouse model, natural products, underexplored microbial sources

## Abstract

The emergence of multidrug‐resistant pathogens presents a major clinical challenge worldwide. The underexplored bacterial genus *Aquimarina* harbors a large biosynthetic potential for the discovery of new classes of antibiotics with distinctive modes of action, which can be unearthed in a scalable manner using a synthetic bioinformatic natural product approach. Here, the discovery of a pair of linear or cyclic bifunctional cationic lipopeptide antibiotics, aquicidine L and aquicidine C4, with opposite antibacterial spectra, is reported, which are chemically synthesized on the basis of distinct structure prediction of the aquicidine gene cluster from *Aquimarina*. Aquicidine L mainly targets both anionic lipopolysaccharide and phosphatidylethanolamine in the bacterial membrane and is efficacious against two different meropenem‐resistant Gram‐negative pathogens in murine peritonitis‐sepsis models. In contrast, aquicidine C4 mainly binds to both anionic cardiolipin and phosphatidylglycerol in the membrane and proves effective at treating methicillin‐ or vancomycin‐resistant Gram‐positive pathogen infections in vivo. Owing to the dual‐mechanism features of aquicidine L or aquicidine C4, both of them are absent in detectable resistance in laboratory tests. These findings provide a pair of naturally inspired and mechanistically interesting therapeutic leads for evading antimicrobial resistance.

## Graphical abstract

The underexplored bacterial genus *Aquimarina* harbors a large biosynthetic potential for discovering new antibiotics. Here, the authors identify a pair of linear or cyclic bifunctional cationic lipopeptide antibiotics, aquicidine L and C4, inspired by the aquicidine gene cluster from *Aquimarina*. Aquicidine L and C4 show opposite antibacterial spectra without detectable resistance and are active against multidrug‐resistant pathogens in vivo.

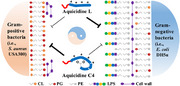

## 1 Introduction

The emergence and widespread distribution of multidrug‐resistant (MDR) pathogens represent a serious and growing risk to global public health, which contributes annually to 1.27 million deaths worldwide and 10 million potential deaths by 2050 absent effective countermeasures.^[^ ^1^, ^2^, ^3^ ^]^ There is still an urgent need for developing new classes of antibiotics with distinctive modes of action (MoAs) and low resistance frequency.^[^ ^4^, ^5^ ^]^ However, the pace of antibiotic development has not matched the rate of global dissemination of antimicrobial resistance (AMR).^[^ ^6^ ^]^ The reasons for this are complex, and one key contributing factor is the high rediscovery rate of natural product (NP) antibiotics due to the overmining of the conventional microbial sources (i.e., Actinomycetes).^[^ ^5^, ^7^ ^]^ There is an obvious advantage to unearth the biosynthetic potential of underexplored microbial sources for the discovery of distinctive structural scaffolds, thus facilitating the development of new drug leads to combat antibiotic‐resistant pathogens.^[^ ^7^, ^8^, ^9^ ^]^

The bacterial genus *Aquimarina* (phylum Bacteroidetes, order Flavobacteriaceae) from the rare marine biosphere harbors a large biosynthetic potential awaiting discovery.^[^ ^10^, ^11^ ^]^ In genomes of *Aquimarina* strains, there is a rich and untapped repertoire of NP biosynthetic gene clusters (BGCs) distinct from characterized gene clusters, which might contribute to their adaptation to diverse ecological niches.^[^ ^11^ ^]^ However, since the discovery of the first *Aquimarina* strain in 2005,^[^ ^12^ ^]^ only three classes of polyketides and one class of non‐ribosomal peptides (NRPs) have been identified from *Aquimarina* through traditional fermentation analysis^[^ ^13^, ^14^, ^15^ ^]^ (Figure S1, Supporting Information). The discovery paradigm is limited by the fact that most BGCs are not actively expressed or expressed at low levels under standard laboratory conditions, even when examined with diverse BGC‐activating strategies.^[^ ^16^, ^17^ ^]^ Along with the increasing accuracy of NPs’ structure prediction and the advancement of modern synthetic organic chemistry, it is now possible to rapidly access a BGC‐encoded metabolite by synthesizing its bioinformatically predicted structure (that is, a synthetic bioinformatic natural product (syn‐BNP)).^[^ ^18^, ^19^, ^20^, ^21^ ^]^ The “biology‐free” synBNP approach can not only access silent BGCs in scale and speed but also overcome the limitation that microbial strains harboring BGCs‐of‐interest are generally unavailable due to their dispersive storage worldwide.

Here, we systematically assessed a panel of NRP BGCs from the underexplored genus *Aquimarina* using the synBNP approach. By chemically synthesizing 17 peptides inspired by the seven NRP BGCs from *Aquimarina*, we rapidly identified seven new lipopeptides against different MDR bacterial pathogens. Among them, a pair of linear or cyclic cationic lipopeptide antibiotics showed unique features with not only opposite antibacterial spectra but also dual molecular targets. The linear peptide aquicidine L showed potent anti‐Gram‐negative activity by mainly targeting both anionic lipopolysaccharide (LPS) and phosphatidylethanolamine (PE) in the bacterial membrane. By contrast, the cyclopeptide aquicidine C4 showed potent anti‐Gram‐positive activity by mainly binding to both anionic cardiolipin (CL) and phosphatidylglycerol (PG) in the membrane. In particular, the dual‐mechanism features of aquicidine L or aquicidine C4 allowed them to kill bacterial pathogens without detectable resistance in laboratory tests. Furthermore, we demonstrate that both aquicidine L and aquicidine C4 were active against two different MDR Gram‐negative and Gram‐positive pathogens in murine peritonitis‐sepsis models, respectively, making them appealing candidates for antimicrobial drug development. Assessing the biosynthetic potential of underexplored microbial sources by the synBNP approach proves to be a productive approach to identify distinctive chemical entities in a scalable manner for developing mechanistically interesting, in vivo active antibiotics without resistant development.

## 2 Results and Discussion

**Figure 1:**
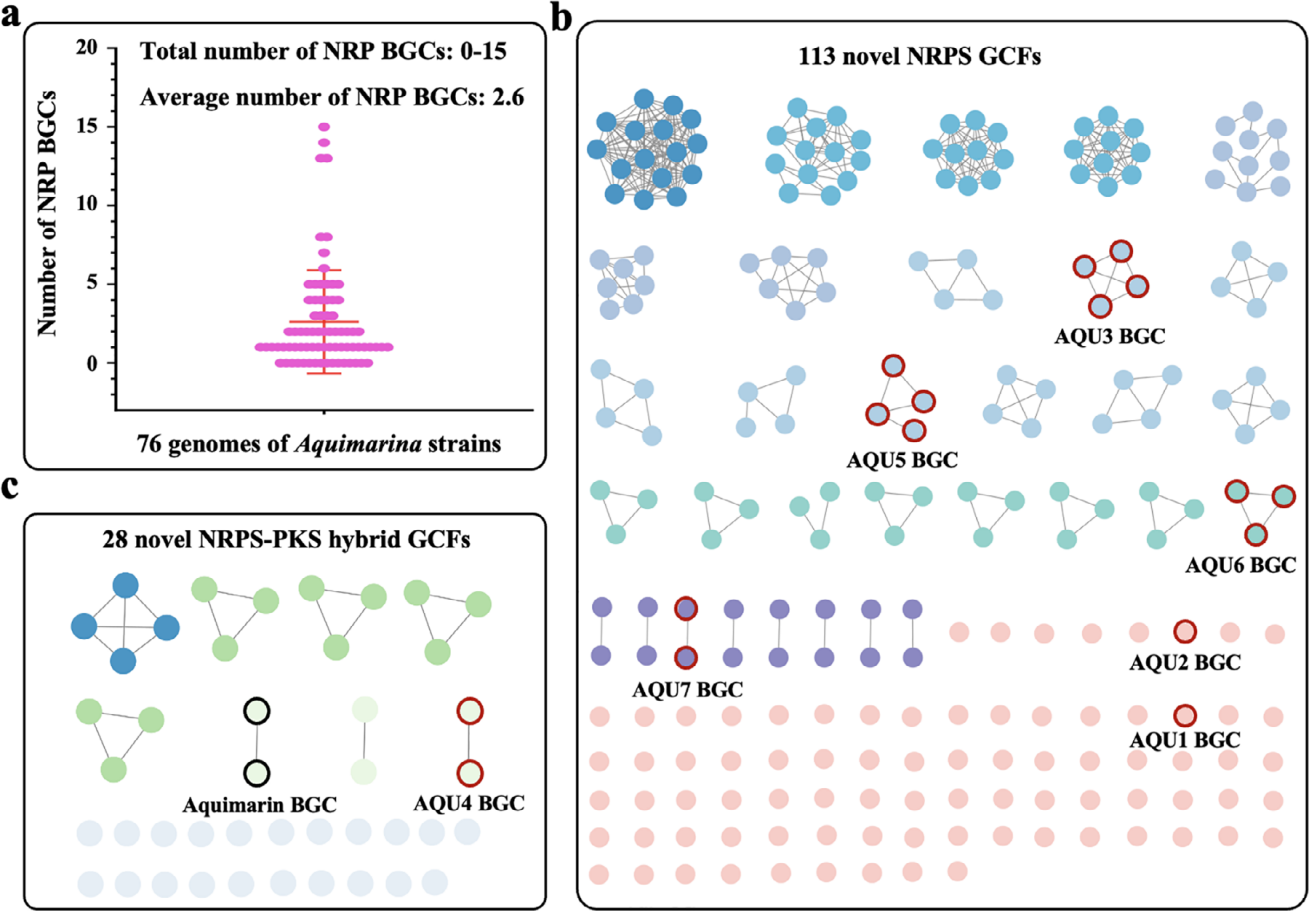
The distribution of NRP BGCs in the underexplored bacterial genus *Aquimarina*. a) The number of NRP BGCs in 76 genome‐sequenced *Aquimarina* strains. b) The distribution of NRPS GCFs in *Aquimarina* with BiGSCAPE. c) The distribution of NRPS‐PKS hybrid GCFs in *Aquimarina* with BiGSCAPE. Each node (circle) represents a single NRPS BGC, and grey edges (lines) denote pairwise similarities above the clustering threshold based on domain architecture and gene order conservation.

**Figure 2:**
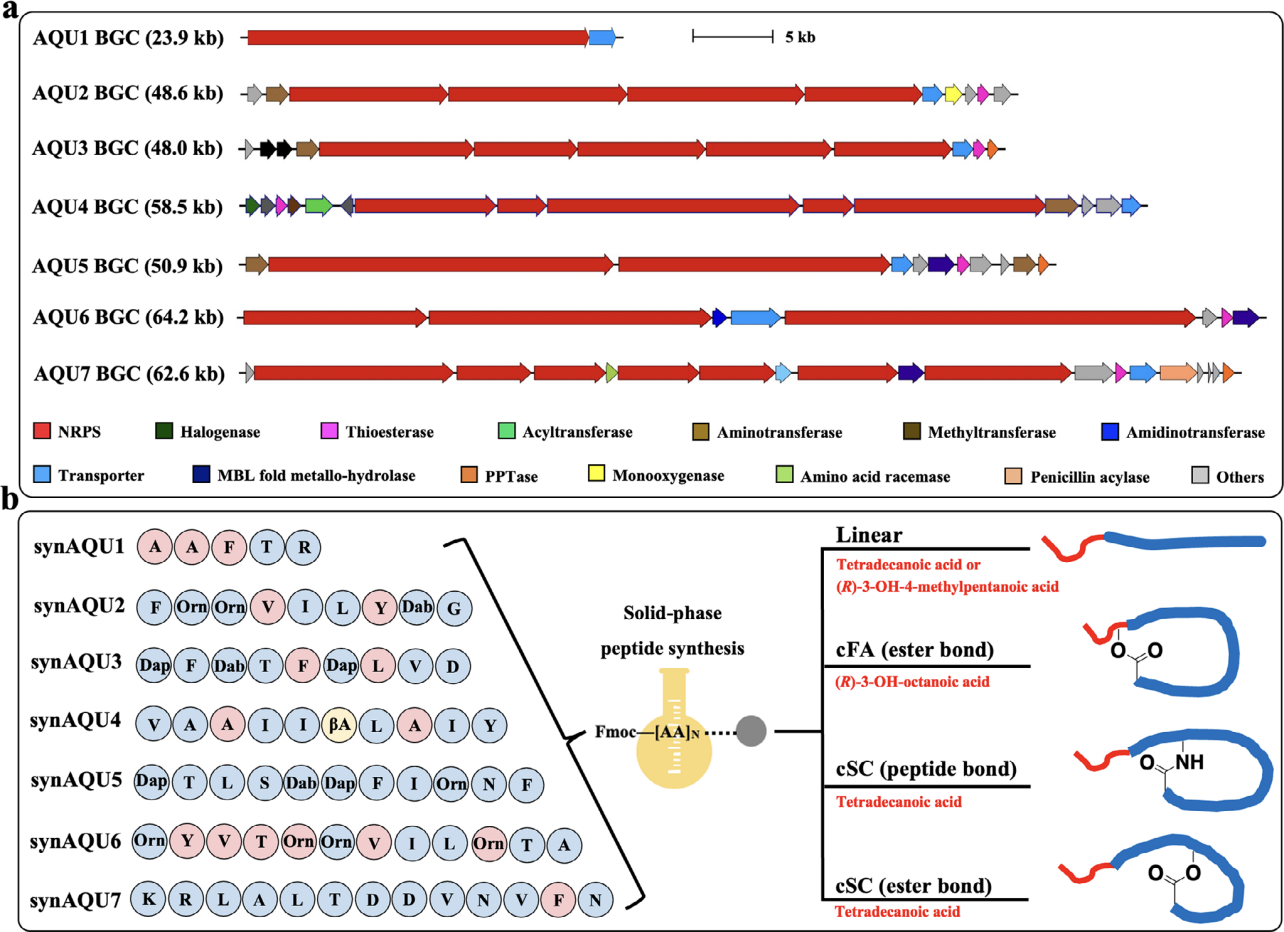
BGCs, structure prediction, and synthesis of synAQUs. a) The seven selected representative AQU BGCs from Aquimarina. b) Structural prediction and synthesis of synAQUs. Tetradecanoic acid‐derivatized linear peptides could be cyclized through a nucleophilic amino acid side chain (cSC). (R)‐3‐hydroxy‐octanic acid‐derivatized linear peptides cyclized through either the hydroxyl group of the fatty acid (cFA) or through a nucleophilic amino acid side chain (cSC). AAs marked in blue and red represent L‐AAs and D‐AAs, respectively. Dab: 2,4‐diaminobutyric acid, Dap: 2,3‐Diaminopropionic acid, Orn: ornithine, and βA: beta‐Alanine.

To systematically access the biosynthetic potential of *Aquimarina*, we first analyzed the NP BGC distribution of all 76 genome‐sequenced *Aquimarina* strains with antiSMASH.^[^ ^22^ ^]^ As shown in Figure S2 (Supporting Information), we totally detected 1,000 BGCs across all genomes grouped in 25 NP families. The average number of BGCs reached up to 13.2 with NRP synthases (NRPSs), polyketide synthases (PKSs), and terpene synthases (TSs) ranking as the most frequent BGC‐encoded antibiotic candidates. The total number of NRP BGCs in a single genome was from 0 to 15, and the average number reached up to 2.6, which provides a rich repertoire for the discovery of antimicrobial peptides with distinctive structural scaffolds (**Figure** **1**a). With the BiGSCAPE algorithm,^[^ ^23^ ^]^ 113 NRPS gene cluster families (GCFs) and 29 NRPS‐PKS hybrid GCFs have been predicted. Only one GCF consisting of two highly similar BGCs encodes the known polyketide‐peptide hybrid antibiotics aquimarins with activity against several Gram‐positive and Gram‐negative bacteria (Figure 1b,c). We then manually analyzed all 141 unidentified NRPS or NRPS‐PKS hybrid GCFs following the three standards: 1) complete BGCs; 2) predicted peptides with at least five building blocks; 3) structure prediction of peptides with high confidence. Finally, seven GCFs‐of‐interest were selected from six *Aquimarina* strains (Figure 1c), and one representative BGC of each GCF‐of‐interest was shown and named as the AQU1 to AQU7 BGC in **Figure** **2**a.

In all seven representative AQU BGCs, the expression of all NRPS genes in each BGC was found to follow the same direction, which makes prediction of BGC‐encoding linear peptide sequences highly accurate (Figure 2a; Figures S3–S9, Supporting Information). Meanwhile, the amino acid incorporated by every A‐domain found in these AQU BGCs could be predicted by A‐domain substrate specificity analysis^[^ ^24^ ^]^ (Tables S1 and S8, Supporting Information). The linear peptide encoded by each AQU BGC was predicted to be the direct precursor to the final synAQU peptide. To chemically synthesize naturally inspired synAQUs, we elected to design synAQU1, synAQU2, synAQU5, synAQU6, and synAQU7 to be *N*‐acylated with tetradecanoic acid, a common fatty acid observed in NRP biosynthesis.^[^ ^25^ ^]^ Considering the sequence of predicted peptide inspired by the AQU3 BGC is similar to that of the known cyclic lipopeptide pedopeptin B^[^ ^26^ ^]^ (Table S9, Supporting Information), (*R*)‐3‐hydroxy‐octanoic acid was used as the fatty acid in the synthesis of both the linear lipopeptide synAQU3‐L and cyclic lipopeptides synAQU3‐C1, synAQU3‐C3, synAQU3‐C4, and synAQU3‐cFA. Meanwhile, the gene architecture and encoding enzymes of the AQU4 BGC are also similar to those of the known lipopeptide aquimarin BGC^[^ ^14^ ^]^ (Figures S6 and S10, Supporting Information), (*R*)‐3‐hydroxy‐4‐methylpentanoic acid was used as the fatty acid in the synthesis of the linear lipopeptide synAQU4‐L. In total, 17 synAQUs, including seven linear lipopeptides and ten cyclic lipopeptides, were designed and synthesized based on the standard Fmoc chemistry‐based solid‐phase peptide synthesis strategy (Figure 2b; Figure S11, Supporting Information). The identity of each synAQU was confirmed by high‐resolution MS (Figures S12–S18 and Table S10, Supporting Information).

**Figure 3:**
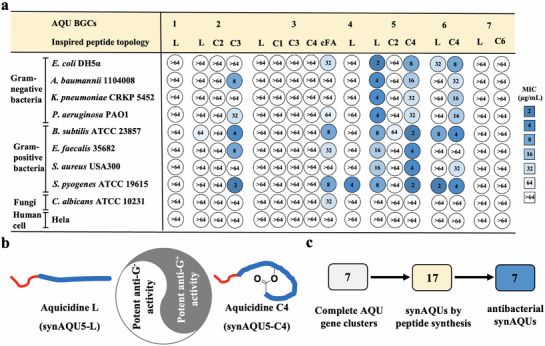
Activities of 17 naturally inspired synAQUs by the seven representative AQU BGCs from Aquimarina. a) Activities of all 17 synAQUs against microorganisms and human cells (MIC: µg mL^−1^). b) Aquicidine L (synAQU5‐L) and aquicidine C4 (synAQU5‐C4) showed opposite antibacterial spectra. Aquicidine L was active against Gram‐negative bacteria, but aquicidine C4 showed potent anti‐Gram‐positive activity. c) A total of 17 linear or cyclic synAQU peptides were synthesized based on bioinformatic prediction of the seven representative AQU BGCs from Aquimarina, and eventually seven synAQUs showed antibacterial activities.

All 17 synAQUs were initially assayed for antimicrobial activity against four Gram‐negative bacteria, four Gram‐positive bacteria, and one fungal pathogen. As shown in **Figure** **3**a, synAQU1‐L, synAQU7‐L, or synAQU7‐C6 cyclized through the hydroxyl of Thr‐6 was not active against all nine tested strains. For the three naturally inspired lipopeptides by the AQU2 BGC, only synAQU2‐C3 was cyclized through the amino of Orn‐3 and showed good activity against *Acinetobacter baumannii* and three Gram‐positive bacterial strains. For five synAQU3‐series lipopeptides, only synAQU3‐cFA was cyclized through the hydroxyl of (*R*)‐3‐hydroxy‐octanoic acid and showed moderate activity against Gram‐positive bacteria. Noteworthy, the known cyclic lipopeptide pedopeptin B has only one different building block from synAQU3‐cFA but shows potent anti‐Gram‐negative activity^[^ ^26^ ^]^ (Table S9, Supporting Information). As reported previously, aquimarins showed good activities against several Gram‐positive and Gram‐negative bacteria,^[^ ^14^ ^]^ but synAQU4‐L had a narrow antibacterial spectrum only with good activity against two *Streptococcus* pathogens (Figure 3a; Table S11, Supporting Information). Intriguingly, synAQU6‐L and synAQU6‐C4 with cyclization through the hydroxyl of Thr‐4 showed similar antibacterial spectra against several Gram‐positive bacteria (Figure 3a; Table S11, Supporting Information). The phenomenon that the linear or cyclic peptides have similar antibacterial spectra is rare and has been observed only for a few paired lipopeptides, such as the synthetic compound LL‐NH2 and the natural product laterocidine^[^ ^27^ ^]^ (Figures S19 and S20, Supporting Information).

In all 17 synAQUs, the linear lipopeptide synAQU5‐L and cyclic lipopeptide synAQU5‐C4 cyclized through the hydroxyl of Ser‐4 were compelling because they not only showed potent antibacterial activities but also had opposite antibacterial spectra. Intriguingly, synAQU5‐C2 cyclized through the hydroxyl of Thr‐2 was not active against all tested strains (Figure 3a). As shown in Figure 3a,b, the linear peptide synAQU5‐L showed potent activity against the four tested Gram‐negative bacteria but weaker activity against the four tested Gram‐positive bacteria. By contrast, synAQU5‐C4 showed potent activity against Gram‐positive bacteria but weak activity against Gram‐negative bacteria. To the best of our knowledge, the conversion of antibacterial spectra between linear or cyclic peptides has not yet reported.^[^ ^28^, ^29^, ^30^ ^]^ We have called the active structures synAQU5‐L and synAQU5‐C4 as aquicidine L and aquicidine C4, respectively.

In broader bioactivity screening, the opposite antibacterial spectra of aquicidine L and aquicidine C4 have been further confirmed (Table S11, Supporting Information). Aquicidine L was active against all Gram‐negative bacteria tested, with MICs ranged from 2 to 8 µg ml^−1^. It was also active against all five tested meropenem‐resistant Gram‐negative pathogens, including two *A. baumannii* strains, two *Klebsiella pneumonia* strains, and one *Pseudomonas aeruginosa* strain, all of which are considered the critical or high groups in the WHO Global Priority Pathogens List (Table S11, Supporting Information). Meanwhile, aquicidine C4 showed potent activity against all Gram‐positive bacteria that we tested except for *Mycobacterium smegmatis* mc^2^ 155, with MICs ranged from 2 to 4 µg ml^−1^. It was also active against two vancomycin‐resistant *Enterococcus* clinical isolates and the methicillin‐resistant *Staphylococcus aureus* USA300, all of which are also considered the high group in the WHO Global Priority Pathogens List. Neither aquicidine L nor aquicidine C4 was active against the two tested *Candida albicans* strains (Table S11, Supporting Information).

**Figure 4:**
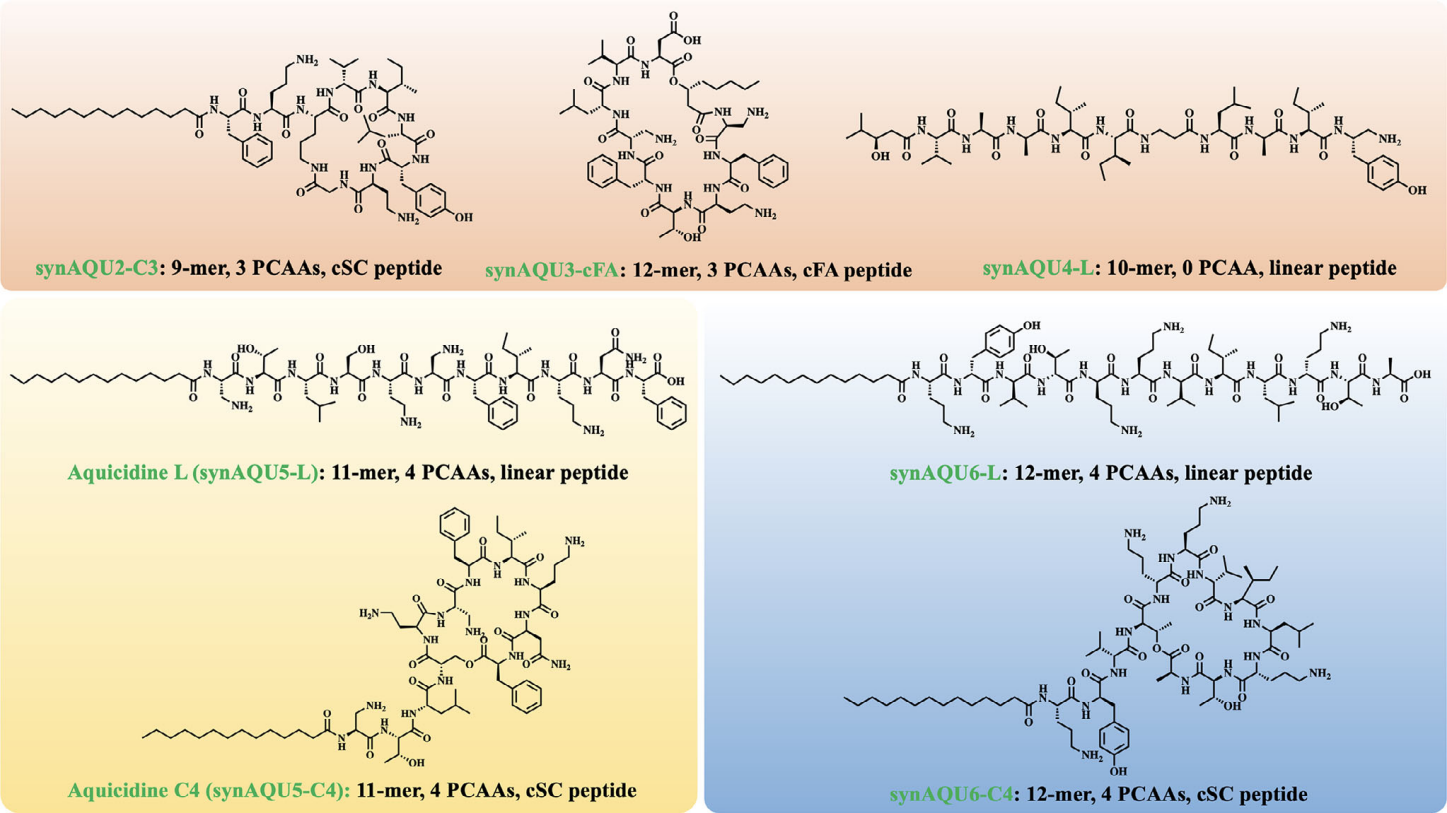
Structures of seven synAQU lipopeptide antibiotics. The key features, including peptide length, the number of positively charged amino acids (PCAAs), and the way of peptide cyclization, were also shown.

Collectively, by synthesizing naturally inspired 17 peptides by the seven representative NRP BGCs from *Aquimarina*, seven new AMPs were rapidly identified with activities against different MDR bacterial pathogens (Figures 3c and **4**). Their structures were further confirmed using 1D/2D NMR data (Figures S21 and S122, Supporting Information). Compared to the traditional bacterial fermentation analysis, the synBNP approach proves to be a productive approach to identify antibiotics with distinctive structural scaffolds from underexplored microbial sources in a scalable manner.

**Figure 5:**
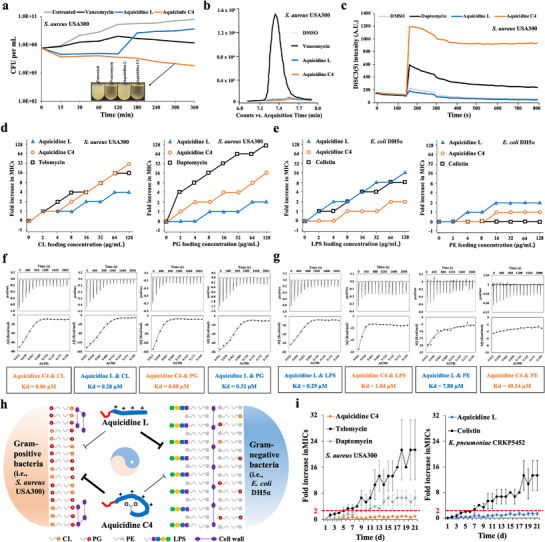
Bactericidal effects and mode‐of‐action analysis of aquicidine L and aquicidine C4. a) Bactericidal activity of aquicidine L or aquicidine C4 against S. aureus USA300. Cultures were incubated with each antibiotic (4× MIC). The number of viable cells was counted (*n* = 3). Vancomycin was used as the control. b) Accumulation of UDP‐MurNAc‐pentapeptide after treating S. aureus cultures with aquicidine L or aquicidine C4 (8× MIC) was monitored by LC‐MS analysis. Vancomycin was used as the control. c) Membrane depolarization in aquicidine L‐ or aquicidine C4‐treated S. aureus cultures was monitored using DISC3(5) dyes. Daptomycin was used as the control. d) The antibacterial activity of aquicidine C4 against S. aureus was determined in the presence of different concentrations of CL or PG (*n* = 2). e) The antibacterial activity of aquicidine L against E. coli was determined in the presence of different concentrations of LPS or PE (*n* = 2). f) Isothermal titration of CL or PG into aquicidine C4 or aquicidine L. g) Isothermal titration of LPS or PE into aquicidine L or aquicidine C4. h) Diagram of modes of action for aquicidine C4 against Gram‐positive bacteria and aquicidine L against Gram‐negative bacteria. i) Resistance acquisition during serial passaging of S. aureus USA300 and K. pneumoniae CRKP5452 in the presence of sub‐MIC levels of aquicidine C4 and aquicidine L, respectively.

To explore why aquicidine L and aquicidine C4 had opposite antibacterial spectra, the detailed MOA studies were performed. In a time‐dependent killing curve analysis using *S. aureus* USA300 as the tested strain, we found that aquicidine C4 showed bactericidal activity at 4× MIC treatment concentration, which decreased the number of viable cells by two orders of magnitude after 5 h. By contrast, aquicidine L only inhibited the growth of *S. aureus* USA300 within 2 h after its treatment, and the number of viable cells was significantly increased after 2 h (**Figure** **5**a). As shown in Figure S123a (Supporting Information), aquicidine L significantly led to cell lysis, and aquicidine C4 also slightly led to cell lysis in *E. coli* after 12 h at 16× MIC treatment concentration. The results indicated that aquicidine L and aquicidine C4 could inhibit the growth of Gram‐negative pathogens (i.e., *E. coli* DH5α) and Gram‐positive pathogens (i.e., *S. aureus* USA300), respectively, via similar membrane‐targeting mechanisms. Scanning electron microscopy images of aquicidine L or C4‐treated *S. aureus* strains showed cell collapse over time. Notably, aquicidine C4 lysed the majority of cells after 180 min of treatment (Figure S124, Supporting Information). To elucidate aquicidine C4's MOA, we attempted to raise resistant mutants by direct plating of *S. aureus* USA300 on aquicidine C4‐containing solid media, but the appeared colonies did not show more than a one‐fold increase in MIC. Using a similar approach, we also failed to identify *E. coli* resistant mutants for aquicidine L. In addition, LC‐MS analysis of *S. aureus* cultures exposed to aquicidine L or aquicidine C4 (8 × MIC) did not show an obvious accumulation of UDP‐MurNAc‐pentapeptide, the key precursor of bacterial cell wall biosynthesis (Figure 5b). To explore the possibility of aquicidine C4 having a detergent‐like activity against *S. aureus*, we tested it for membrane depolarization and cell lytic activities using 3,3′‐dipropylthiadicarboncyanine iodide (DiSC3(5)) and SYTOX fluorescence assays, respectively. As shown in Figure 5c and Figure S123b (Supporting Information), aquicidine C4, not aquicidine L caused a significant increase in both DiSC3(5) and SYTOX fluorescence, indicating that it induced membrane depolarization and cell lysis in *S. aureus*. Collectively, the results indicate that aquicidine C4 potentially targets one or more cell membrane components, thus resulting in the bactericidal effect. Meanwhile, compared to altering a protein target through genomic DNA mutations, it is more difficult to introduce changes in a small‐molecule target. Considering that aquicidine L‐ or aquicidine C4‐resistant mutants were not identified, we proposed that they possibly bind to small molecules rather than proteins as the mode of inhibiting bacterial growth.

To identify metabolites that interact with aquicidine L or aquicidine C4, we screened a panel of cell membrane components, including anionic LPS, anionic CL, anionic PG, PE, phosphatidylcholine (PC, the main membrane component in human cells), menaquinone (MK), and ubiquinone (UQ), for their ability to suppress aquicidine L's or aquicidine C4's antibacterial activities. We observed that MK, UQ or PC did not show dose‐dependent inhibition of aquicidine L's or aquicidine C4's antibacterial activities (Figure S125, Supporting Information). However, two of these components that we tested, both CL and PG showed stronger dose‐dependent inhibition of aquicidine C4's antibacterial activity compared to aquicidine L using *S. aureus* USA300 as the tested strain (Figure 5d). Meanwhile, two of the components that we tested, both LPS and PE showed stronger dose‐dependent inhibition of aquicidine L's antibacterial activity compared to aquicidine C4 using *E. coli* DH5α as the tested strain (Figure 5e). Many known cationic lipopeptides (i.e., colistin, macolacin, and laterocidine) often interact with anionic cell membrane components (i.e., LPS), thus showing anti‐Gram‐negative activity.^[^ ^31^, ^32^, ^33^ ^]^ Both aquicidine L and aquicidine C4 are cationic lipopeptides with four positively charged residues, 1‐L‐Dap, 5‐L‐Dab, 6‐L‐Dap and 9‐L‐Orn, which have the abilities to bind to anionic metabolites. Using isothermal titration calorimetry, we observed that although both aquicidine L and aquicidine C4 bound CL or PG, the binding affinities between aquicidine C4 and CL or PG were higher than those of aquicidine L (Figure 5f). By contrast, aquicidine L had a stronger binding affinity to LPS or PE than aquicidine C4 (Figure 5g; Figure S126, Supporting Information).

Previous studies indicated that LPS is only distributed in Gram‐negative not Gram‐positive, bacteria, and PE is the main component in the Gram‐negative bacterial cell membrane.^[^ ^34^, ^35^ ^]^ For example, the ratios of PE, PG, and CL are 80%, 15% and 5%, respectively, in *E. coli*. For *K. pneumoniae*, the ratios of PE, PG, and CL are 82%, 5% and 6%, respectively. However, both CL and PG are the main components in the cell membrane of some Gram‐positive bacteria. For example, the ratios of PE, PG, and CL are 0%, 58% and 42%, respectively, in *S. aureus*.^[^ ^34^, ^35^ ^]^ Due to the distinct characteristics on membrane composition in Gram‐negative and Gram‐positive bacteria, the unique MOAs of aquicidine L and aquicidine C4 made them show opposite antibacterial spectra: the linear aquicidine L mainly targets both LPS and PE, thus showing potent anti‐Gram‐negative activity, and the cyclic aquicidine C4 mainly binds to both CL and PG, thus showing potent anti‐Gram‐positive activity (Figure 5h).

Although many known antibiotics have been reported to bind unspecifically to Gram‐positive bacterial cell membranes by a physical adsorption mechanism, specific phospholipid‐peptide interactions are rare.^[^ ^36^, ^37^ ^]^ Until now, a few antibiotics have been identified that bind to a phospholipid, such as CL and PG.^[^ ^36^ ^]^ These include the Gram‐positive‐active antibiotics telomycin and LL‐A0341β1 targeting CL as well as daptomycin targeting PG.^[^ ^38^, ^39^, ^40^ ^]^ Telomycin is in the pre‐clinical development for the use of MDR Gram‐positive pathogen infections, and daptomycin has been widely used clinically as a topical lipopeptide antibiotic. Unfortunately, Gram‐positive pathogens exposed to telomycin, which binds to CL or daptomycin, which mainly binds to PG, have been reported to easily develop resistance.^[^ ^38^, ^40^ ^]^ By contrast, aquicidine C4 simultaneously targeted both CL and PG, which possibly makes it have a very low resistance rate compared to telomycin or daptomycin.^[^ ^41^ ^]^ Because we failed to raise mutants resistant to aquicidine C4 by plating it on antibiotic‐containing solid media, we tried to identify resistant mutants by daily serial passage in the presence of sub‐MIC levels of aquicidine C4 using telomycin and daptomycin as the controls. As shown in Figure 5i, *S. aureus* USA300 rapidly developed resistance to both telomycin and daptomycin with increasing MICs by 6.7‐ to 21.3‐fold. By contrast, after 21 days of constant exposure to aquicidine C4, no higher than twofold MIC change was observed (Figure 5i). On the other hand, compared to other known only LPS‐targeting antibiotics (i.e., colistin and macolacin),^[^ ^31^, ^32^ ^]^ aquicidine L was able to simultaneously bind to both LPS and PE in Gram‐negative bacteria. Although the aquicidine L's binding affinity to LPS in the outer membrane was higher than to PE in bacterial cytoplasmic membrane (Figure 5e,g), we proposed that aquicidine L will also possibly have a very low resistance rate to Gram‐negative bacteria due to its dual molecular targets.^[^ ^41^ ^]^ As shown in Figure 5i, *Klebsiella pneumoniae* CRKP5452 developed resistance to colistin with an increased MIC by 13.3‐fold, but no higher than two‐fold MIC change was observed for aquicidine L. Finally, we sequenced the genomes of colistin‐resistant *K. penumoniae* CRKP5452, telomycin‐resistant *S. aureus* USA300 as well as daptomycin‐resistant *S. aureus* USA300, and identified their corresponding lipid synthesis mutants, including *K. pneumoniae* CRKP5452 PhoQ^L348Q^, *S. aureus* USA300 Cls2^A338stop^ and *S. aureus* USA300 MprF^T345A^, respectively (Figure S127, Supporting Information). To the best of our knowledge, there is no known PE‐targeting, Gram‐negative‐active antibiotic and therefore we did not generate the PE synthesis mutants for aquicidine L. Colistin, telomycin and daptomycin showed 16‐fold, 32‐fold and 16‐fold increase in MICs against the resistant mutants *K. pneumoniae* CRKP5452 PhoQ^L348Q^, *S. aureus* USA300 Cls2^A338stop^ and *S. aureus* USA300 MprF^T345A^ compared to the corresponding wild types, respectively (Figure S127, Supporting Information). Intriguingly, aquicidine L showed only 4‐fold increase in MIC against the LPS‐resistant mutant *K. pneumoniae* CRKP5452 PhoQ^L348Q^. In particular, aquicidine L also exhibited good activities with MIC of 8 µg ml^−1^ against the two clinically isolated, colistin‐resistant strains *K. pneumoniae* NCTC 5056 and 15580 (Figure S127, Supporting Information). The results indicated that LPS might be not the only target for aquicidine L and aquicidine L was predicted to bind to both LPS and PE simultaneously, thus showing a dual‐mechanism, synergistic inhibitory effect against *K. pneumoniae* CRKP5452. Similarly, aquicidine C4 also showed only 4‐fold increase in MICs against the telomycin‐resistant mutant *S. aureus* USA300 Cls2^A338stop^ or the daptomycin‐resistant mutant *S. aureus* USA300 MprF^T345A^ (Figure S127, Supporting Information). Aquicidine C4 was predicted to bind to both CL and PG simultaneously, thus showing a dual‐mechanism, synergistic inhibitory effect against *S. aureus* USA300. Collectively, our results indicate that the dual‐mechanism features of aquicidine L or aquicidine C4 made them difficult to develop resistance in laboratory tests and thus become promising lead structures to evade antibacterial resistance.

To test in vivo antibacterial activities of aquicidine L or aquicidine C4, we first observed their cytotoxicities and haemolytic activities. Even at the highest concentration we tested, neither aquicidine L nor aquicidine C4 showed cytotoxicity against the human cell line HeLa or haemolytic activity (Figure S128a,b, Supporting Information). We also demonstrate that both aquicidine L and aquicidine C4 had no obvious acute toxicities in mouse at the highest concentration we tested (250 mg kg^−1^) (Figure S128c, Supporting Information).

**Figure 6:**
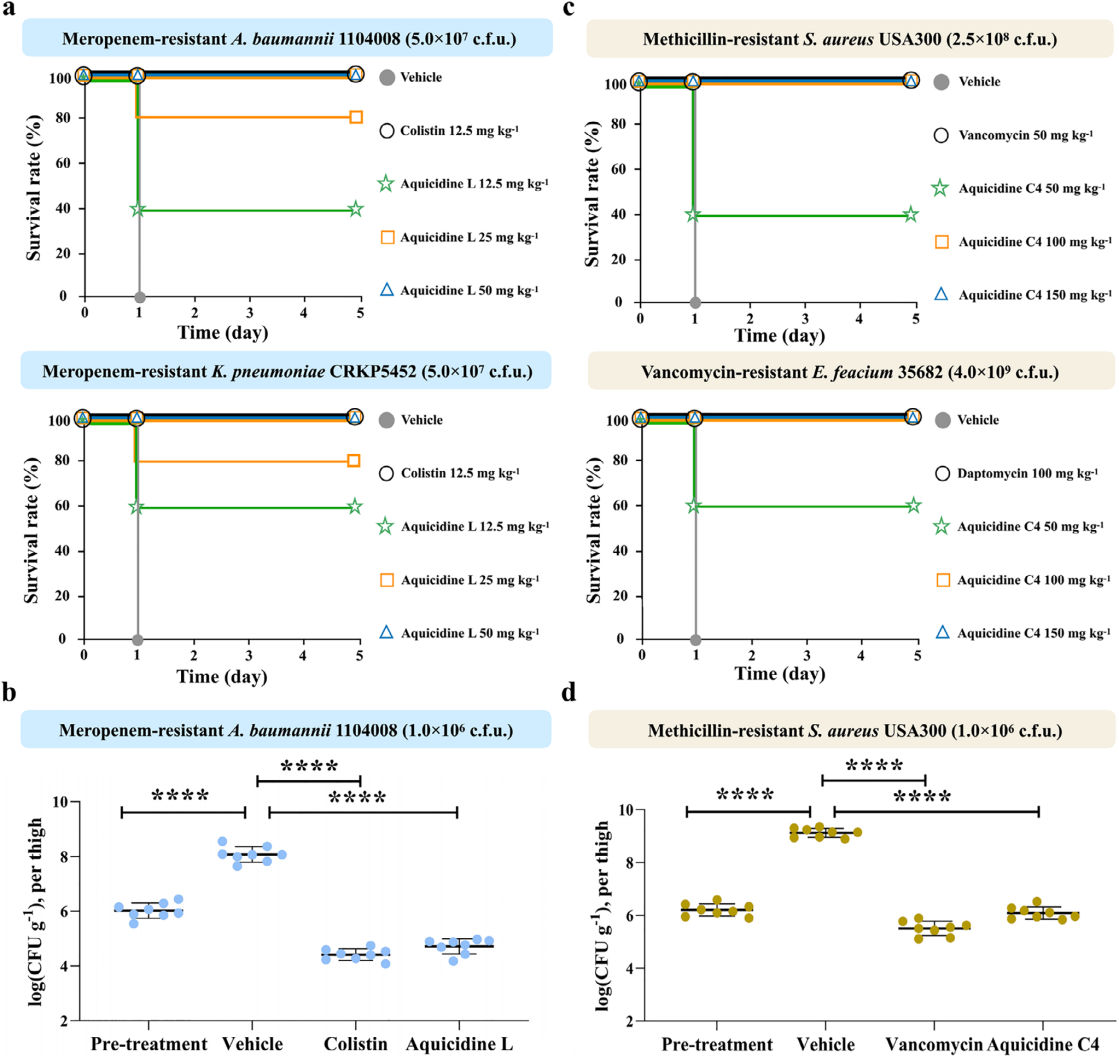
The antibacterial activities of aquicidin L and aquicidine C4 in both a murine peritonitis‐sepsis model and a neutropenic thigh infection model. a) Aquicidine L (12.5, 25, or 50 mg kg^−1^) or colistin (12.5 mg kg^−1^) was subcutaneously injected 1 h after intraperitoneal administration of *A. baumannii* 1104008 or *K. pneumoniae* CRKP5452 into mice (*n* = 5). b) CFU counts from a neutropenic thigh infection model using *A. bauamnnii* 1104008. Mice were subcutaneously given aquicidine L (25 mg kg^−1^) or colistin (25 mg kg^−1^). c) Aquicidine C4 (50, 100, or 150 mg kg^−1^), daptomycin (100 mg kg^−1^) with 15 mm CaCl_2,_ or vancomycin (50 mg kg^−1^) were subcutaneously injected 1 h after intraperitoneal administration of *S. aureus* USA300 or *E. faecium* 35682 into mice (n = 5). d) CFU counts from a neutropenic thigh infection model using *S. aureus* USA300. Mice were subcutaneously given aquicidine C4 (50 mg kg^−1^) or vancomycin (50 mg kg^−1^). 20% solutol was used as the vehicle. Significant differences between groups were analysed by one‐way analysis of variance (****P < 0.0001) (*n*  =  8 thighs).

Using both a murine peritonitis‐sepsis model and a neutropenic thigh infection model, we examined the in vivo efficacy of aquicidine L and aquicidine C4 against MDR Gram‐negative and Gram‐positive pathogens, respectively. Compared to treatment with vehicle alone (20% solutol), treatment of a meropenem‐resistant *A. baumannii* 1104008 or *K. pneumonia* CRKP5452 strain infection with aquicidine L (12.5, 25, and 50 mg kg^−1^) significantly decreased the mortality of infected mice, and a minimal dose of 50 mg kg^−1^ aquicidine L was required for 100% survival (**Figure** **6**a). Meanwhile, the bacterial burden in each infected thigh was determined after 24 h. Similar to colistin, aquicidine L showed potent activity against meropenem‐resistant *A. baumannii* 1104008, resulting in a 3log_10_ reduction in CFUs compared with the vehicle (P < 0.0001) (Figure 6b). On the other side, treatment of a vancomycin‐resistant *Enterococcus faecium* 35682 or methicillin‐resistant *S. aureus* USA300 infection with aquicidine C4 (50, 100, and 150 mg kg^−1^) also dramatically decreased the mortality of infected mice. A minimal dose of 100 mg kg^−1^ aquicidine C4 was required for 100% survival in both mouse peritonitis‐sepsis models (Figure 6c). Similar to vancomycin, aquicidine C4 showed potent activity against methicillin‐resistant *S. aureus* USA300, resulting in a 3log_10_ reduction in CFUs compared with the vehicle (P < 0.0001) (Figure 6d). Collectively, our results suggested that aquicidine L and aquicidine C4 could be appealing drug leads for the development of next‐generation antibacterial therapeutics for addressing the emergent AMR crisis.

## 3 Conclusion

In this study, we accessed a panel of NRP BGCs from the underexplored bacterial genus *Aquimarina* in a scalable manner using the “biology‐free” synBNP approach, thus resulting in the rapid identification of up to seven new lipopeptide antibiotics with good activities against different MDR pathogens. Traditional fermentation screening platforms have failed to introduce new classes of antibiotics in the last decades, partially due to overmining conventional microbial sources. Underexplored sources, including unexplored ecological niches, host microbiomes, as well as untapped bacterial or fungal taxa, open up new opportunities to discover drug leads with new chemical and mechanistic characteristics for the development of next‐generation antibiotics, such as teixobactin, darobactin, and clovibactin.^[^ ^8^, ^42^, ^43^, ^44^ ^]^

Furthermore, the syn‐BNP approach that relies on structure prediction followed by chemical synthesis of biologically produced small molecules efficiently overcomes the resource‐intensive and time‐consuming limitations of traditional screening platforms and provides a high‐throughput, inspirational interdisciplinary pipeline to mine and unearth the hidden biosynthetic potential of underexplored microbial sources.^[^ ^18^, ^45^, ^46^, ^47^ ^]^

In all 17 naturally inspired synAQUs that we synthesized, aquicidine L and aquicidine C4 were compelling with opposite antibacterial spectra and dual molecular targets. To the best of our knowledge, the conversion of antibacterial spectra of paired linear and cyclic peptides has not yet been reported in previous studies.^[^ ^28^, ^29^, ^30^ ^]^ After testing different software for the structure prediction of small peptides, we found that it was difficult to predict the secondary structures of aquicidine L or aquicidine C4 because both of them contain three non‐canonical residues (_L_‐Dap, _L_‐Dab, and _L_‐Orn). Therefore, _L_‐Lys was first used to replace _L_‐Dap, _L_‐Dab or _L_‐Orn in aquicidine L or aquicidine C4, considering _L_‐Lys exhibits similar physicochemical properties to the three non‐canonical residues. As shown in Figure S129 (Supporting Information), aquicidine L (Lys‐substituted) was predicted to form an alpha‐helix with two hydrophobic sides. The helical structure could lead aquicidine L to create a water‐lipid interface at both outer and inner membranes in Gram‐negative bacteria. Meanwhile, the hydrophobic nature of aquicidine L was predicted to be critical for interacting with target membranes, thus embedding into the lipid bilayer of both outer and inner membranes in Gram‐negative bacteria (i.e., *E. coli*).^[^ ^48^ ^]^ Our results also indicated that aquicidine L indeed showed higher binding affinities to LPS and PE than aquicidine C4 (Figure 5g). In contrast, aquicidine C4 (Lys‐substituted) was predicted to be characterized by a closed‐loop structure formed through an ester bond between the fourth AA (_L_‐Ser‐4) and the last AA (_L_‐Phe‐11). Generally, cyclic structures restrict the conformational freedom of each ingredient inside the cycle, thus forcing the cyclopeptide into a more rigid secondary structure and diminishing the entropic term of Gibbs energy.^[^ ^48^ ^]^ Therefore, we speculated that the cyclic feature of cationic aquicidine C4 could increase its binding affinities to the anionic phospholipids CL or PG in Gram‐positive pathogens (i.e., *S. aureus*), which were also confirmed by the ITC experiments (Figure 5f). Together, although both AA components and charges of aquicidine L and aquicidine C4 are almost same, their distinct secondary structures may lead to different binding affinities to phospholipids in Gram‐positive and Gram‐negative bacteria, thus showing the unique opposite antibacterial spectra.

Generally, after the ring of a cyclic peptide is opened, the corresponding linear peptide has three varied antibacterial spectra: 1) comparable or lower activity against the same bacterial pathogens (i.e., the synthetic compound LL‐NH2 and the natural product laterocidine, the synthetic compound LB‐NH2 and the natural product brevicidine)^[^ ^27^ ^]^; 2) no activity (i.e., the natural product pelgipeptin B’ and the natural product pelgipeptin B)^[^ ^49^ ^]^; 3) more specific activity (i.e., the synthetic compound bacaucin‐1 and the natural product bacaucin)^[^ ^50^ ^]^ (Figures S19 and S20, Supporting Information). Natural product isolation has often relied on traditional fermentation screening analysis, and this process is difficult to produce paired linear or cyclic peptides at the same time due to the strict biosynthetic logic of NRPs.^[^ ^51^ ^]^ The synBNP approach proves effective at simultaneously assessing the activity of naturally inspired paired linear or cyclic peptides, thus revealing the unique phenomenon that aquicidine L and aquicidine C4 had opposite antibacterial spectra.

The dual‐mechanism features of aquicidine L or aquicidine C4 allowed them to kill bacterial pathogens without detectable resistance in laboratory tests. Although we did not observe aquicidine L or aquicidine C4 resistance in MDR pathogens, this does not rule out that their resistant mutants might eventually appear with broader environmental exposure. Meanwhile, aquicidine L was active in vivo against meropenem‐resistant *A. baumannii* and *K. pneumoniae* clinical isolates, and aquicidine C4 also showed potent activity against vancomycin‐resistant *E. faecium* and methicillin‐resistant *S. aureus* clinical isolates in murine peritonitis‐sepsis models or neutropenic thigh infection models, providing two easily scaled therapeutic leads for these troubling MDR bacterial pathogens. In the future, more animal model studies will still be required for exploring the remaining issues that have to be addressed to move aquicidine L or aquicidine C4 through therapeutic development into clinical use. In summary, aquicidine L's and aquicidine C4's unique MOAs, absence of detectable resistance, and in vivo activities make them appealing lead compounds for developing next‐generation lipopeptide antibiotics that could address the growing global AMR threat.

## 4 Experimental Section

4.1

4.1.1

##### Chemical Reagents and Consumables

Reagents for solid‐phase peptide synthesis (SPPS), including 2‐chlorotrityl resin, *N*‐Fmoc amino acids, DCM (dichloromethane), benzoyl chloride, DIPEA (*N*,*N*‐diisopropylethylamine), DMAP (4‐dimethylaminopyridine), DMF (*N*,*N*‐dimethylformamide), HATU (*O*‐(7‐azabenzotriazol‐1‐yl)‐*N*,*N*,*N*,*N*′‐tetramethyluronium hexafluorophosphate), HFIP (hexafluoroisopropanol), palladium [Pd(PPh3)4], PyAOP ((7‐azabenzotriazol‐1‐yloxy) tripyrrolidinophosphonium hexafluorophosphate), TFA (trifluoroacetic acid) and triisopropylsilane, were purchased from GL Biochem (Shanghai, CN). The synthetic lipids 1‐Palmitoyl‐2‐oleoyl‐phosphatidylethanolamine (PE, catalogue no. P293160, 98%) and 1‐Palmitoyl‐2‐oleoyl‐sn‐glycero‐3‐phospho‐rac‐(1‐glycerol) (PG, catalogue no. P400387, 98%) were purchased from Aladdlin (Shanghai, CN). Cardiolipin (CL, catalogue no. C1649) was purchased from Sigma–Aldrich (Darmstadt, DEU). Lipopolysaccharides (LPS, Abcone catalogue no. L26331) from *Escherichia coli* O55:B5 were purchased from Abcone (Shanghai, CN).

##### Solid Phase Peptide Synthesis (SPPS)

synAQU peptides were synthesized on 2‐chlorotrityl chloride resin using a standard Fmoc chemistry‐based solid‐phase peptide synthesis method.

For the synthesis of linear synAQU peptides (synAQU1‐L, synAQU2‐L, synAQU3‐L, synAQU4‐L, synAQU5‐L, synAQU6‐L, synAQU7‐L), peptide synthesis started from the last AA of each synAQU peptide, which was loaded on 2‐cholorotrityl resin (0.3 g, 0.552 mmol g^−1^) and was swollen in DCM for 20 min, drained as well as washed with DMF (3 mL, 3×). By using Fmoc‐protected amino acids (2 equiv., relative to resin loading) mixed with HATU (2 equiv.) and DIPEA (2 equiv.) in DMF (5 mL), individual amino acids were coupled step by step. Then, coupling reactions were performed for 1 h with occasional swirling and washed with DMF (3 mL, 3×). Fmoc‐deprotection was done using 20% piperidine in DMF (3 mL) for 7 min and repeated twice. The resin was washed with DMF (3 mL, 5×) and then coupled with a subsequent *N*‐Fmoc amino acid.

For the synthesis of synAQU peptides with peptide bond‐mediated cyclization (synAQU2‐C2, synAQU2‐C3, synAQU3‐C1, synAQU3‐C3), peptide synthesis started from the last AA of each synAQU peptide, which was loaded on 2‐cholorotrityl resin (0.3 g, 0.552 mmol/g) and was swollen in DCM for 20 min, drained as well, and washed with DMF (3 mL, 3x). By using Fmoc‐protected amino acids (2 equiv., relative to resin loading) mixed with HATU (2 equiv.) and DIPEA (2 equiv.) in DMF (5 mL), individual amino acids were coupled step by step. Then, coupling reactions were performed for 1 h with occasional swirling and washed with DMF (3 mL, 3×). The alloc group of Dap, Dab, or Orn was first removed by two rounds of palladium [Pd(PPh3)4]‐catalyzed deprotection in DCM. In each round, plladium (0.25 equivalent) and phenlsilane (12 equivalents) were added to 2‐chlorotrityl resins suspended in DCM and reacted for 1 hr. The resin was removed by 20% HFIP in DCM for 2 h. Then, soluble peptides were cyclized using PYAOP (8 equivalents) and DIPEA (30 equivalents) in DMF and extracted using DCM and H_2_O with 1% formic acid. Cyclic synAQU peptides were dissolved in methanol and dried under vacuum overnight.

For the synthesis of synAQU peptides with ester bond‐mediated cyclization (synAQU3‐C4, synAQU3‐cFA, synAQU5‐C2, synAQU5‐C4, synAQU6‐C4, synAQU7‐C6), peptide synthesis started from the fifth AA of each synAQU peptide, which was loaded on 2‐cholorotrityl resin (0.3 g, 0.552 mmol g^−1^) and was swollen in DCM for 20 min, drained as well as washed with DMF (3 mL, 3×). By using Fmoc‐protected amino acids (2 equiv., relative to resin loading) mixed with HATU (2 equiv.) and DIPEA (2 equiv.) in DMF (5 mL), individual amino acids were coupled step by step. Coupling reactions were performed for 1 h with occasional swirling and washed with DMF (3 mL, 3×). Then, resin was mixed with benzoyl chloride (20 equivalents), DIPEA (40 equivalents), the last AA of synAQU peptide (20 equivalents), DMAP (0.8 equivalents) in DCM (10 mL) and gently rotated for 72 h. After ester bond formation, the remaining amino acids were coupled as described above. The resin was removed by 20% HFIP in DCM for 2 h. Then, soluble peptides were cyclized using PYAOP (8 equivalents) and DIPEA (30 equivalents) in DMF and extracted using DCM and H_2_O with 1% formic acid. Cyclic synAQU peptides were dissolved in methanol and dried under vacuum overnight.

For final cleavage of all linear or cyclic synAQU peptides, each peptide was dissolved in 3 mL of cleavage cocktail (95% TFA, 2.5% triisopropylsilane, and 2.5% H_2_O) for 1.5 h. A cold mixture of diethyl ether:hexane (1:1) was then added and kept for 10 min at ‐20 °C to precipitate each synAQU peptide. Finally, peptide pellets were collected, redissolved in 5 mL of methanol, and dried under vacuum.

##### Peptide Purification and Identification

Crude synAQUs were purified using an Agilent 1260 Series High‐Performance Liquid Chromatography (HPLC) equipped with a XBridge BEH C18 OBD Prep column (10 × 150 mm, 5 µm). Each synAQU peptide was eluted using a linear gradient from 20 to 50% gradient of CH_3_CN. The identity of each synAQU peptide was determined by HPLC‐HRMS analysis using an Agilent 1290 Series HPLC coupled to a 6546 Series QTOF mass spectrometer equipped with a XBridge C18 Column (4.6 × 150 mm, 5 µm) and controlled by Masshunter software. Furthermore, a Bruker Avance DMX 600 MHz spectrometer equipped with cryogenic probes was used to acquire the ^1^H, ^13^C, DEPT135, ^1^H‐^1^H COSY, ^1^H‐^13^C HSQC, and ^1^H‐^13^C HMBC NMR spectra for all 17 synAQUs. All spectra were recorded at 25 °C in DMSO‐*d*_6_. Chemical shift values were reported in parts in million (ppm) and referenced to residual solvent signals: 2.50 ppm (^1^H) and 39.52 ppm (^13^C).

##### Minimum Inhibitory Concentration (MIC) Assay

All MIC assays were performed two independent times (n = 2) in 96‐well plates using a broth micro‐dilution method. The lowest concentration of that inhibited visible microbial growth was recorded as the MIC value.

For most of bacterial strains, overnight cultures were diluted 5000‐fold in LB broth. For *E. faecium* and *S. aureus*, overnight cultures were diluted 1000‐ and 10000‐fold in LB broth, respectively. 100 µL of diluted cells was mixed with 100 µL of LB broth containing each peptide ranging from 64 to 0.125 µg mL^−1^ at 2‐fold serial dilutions. Plates were incubated at 37 °C for 16 h, and the MIC value was recorded.

For fungal strains, overnight cultures were diluted 2000‐fold in YPD broth. 100 µL of diluted cells was mixed with 100 µL of YPD broth containing each peptide ranging from ranged from 64 to 0.125 µg mL^−1^ at 2‐fold serial dilutions. Plates were incubated at 30 °C for 16 h, and the MIC value was determined.

For *M. smegmatis* mc^2^ 155, the strain was cultured in 7H9 broth (supplemented with 0.2% glucose, 0.2% glycerol, and 0.05% tyloxapol) for 48 h at 37 °C and 200 rpm. The culture was diluted to an OD_600_ of 0.005 using 7H9 broth, and 100 µL of diluted cells was added to 100 µL of 7H9 broth containing each compound ranging from 64 to 0.125 µg mL^−1^ at 2‐fold serial dilutions. After an incubation of 48 h at 37 °C, 30 µL of Alamar Blue cell viability reagent was added into each well. The wells that remained blue were determined to contain MICs of each compound after an additional incubation of 24 h at 37 °C.

##### Time‐dependent Killing Assay

Overnight cultures of *S. aureus* USA300 were diluted 1:10000 in LB broth and incubated for 2 h at 37 °C and 200 rpm. Then the cultures were treated with 8x the MIC of each peptide. After 15, 30, 60, 120, 180, 240, 300, and 360 min, 100 µL of cell suspension were collected, serially diluted, and plated on LB agar plates. After an incubation of 16 h at 37 °C, bacterial colonies were counted. Experiments were performed three independent times (*n* = 3).

##### UDP‐MurNAc‐Pentapeptide Accumulation Assay

To determine the effects of aquicidine L or aquicidine C4 on bacterial cell wall biosynthesis of *S. aureus* USA300, the accumulation of the precursor UDP‐MurNAc‐pentapeptide was investigated in antibiotic‐treated cell cultures. 50 µL of overnight cultures were transferred to 5 mL of fresh LB broth and cultured to 0.5 of OD_600_ at 37 °C and 200 rpm. The cultures were treated with 130 µg/mL of chloramphenicol for 15 min at 37 °C and 200 rpm, and 128 µg/mL of aquicidine L or 32 µg/mL aquicidine C4 was added. 8 µg/mL of vancomycin and DMSO were used as positive and negative controls, respectively. After an incubation of 1 hr, the cell pellet from 0.5 mL of the cell cultures was resuspended in 30 µL of ddH_2_O and then boiled for 15 min. Finally, the supernatant was collected and then detected using an Agilent 1290 Series High‐Performance Liquid Chromatography (HPLC) coupled to a 6546 Series QTOF mass spectrometer equipped with an XBridge C18 column (4.6 × 150 mm, 5 µm). Solvent A = H_2_O (0.1% v/v formic acid) and solvent B = CH_3_CN (0.1% v/v formic acid) were used for HPLC analysis with 0.4 mL min^−1^ of flow rate at 25 °C. Gradient conditions were as follows: t = 0‐1 min, 3% solvent B; t = 2‐7 min, 3‐95% solvent B; t = 7‐8 min, 95% solvent B; t = 8–9 min, 95‐3% solvent B; t = 9–10 min, 3% solvent B.

##### Membrane Depolarization Assay

Bacterial membrane depolarization assay was performed in 384‐well black microtiter plates. Overnight cell cultures of *S. aureus* USA300 were collected and resuspended in PBS buffer to an OD_600_ of 0.5. Then, 100 µL of the diluted cell suspension and 50 µL of 20 µm DiSC_3_(5) dye were added to 300 µL of PBS buffer, which was incubated for 15 min at room temperature in the dark. 50 µL of 2 m KCl was then added and incubated for another 15 min. Fluorescence intensity was determined continually at 2 s intervals (Ex/Em 643/675 nm) using a Tecan spark multimode microplate reader. When the signal stabilized, the appropriate amount of aquicidine L or aquicidine C4 (6.4 mg mL^−1^ in DMSO, 4 × MIC) was added. DMSO and daptomycin were used as negative and positive controls, respectively. Data were shown as the relative intensity with respect to the average fluorescence signal prior to the addition of each compound. All assays were performed two independent times (*n* = 2), and a representative fluorescence recording was presented.

##### Membrane Lysis Assay

Bacterial membrane lysis assay was performed in 384‐well black microtiter plates. Overnight cultures of *S. aureus* USA300 were collected and resuspended in PBS buffer to an OD_600_ of 0.5. 1 µL of 5 mM SYTOX Green dye was added to 2.5 mL of the diluted cell suspension, which was incubated for 10 min at room temperature in the dark. Fluorescence intensity was detected continually at 2 s intervals (Ex/Em 488/523 nm) using a Tecan spark multimode microplate reader. When the fluorescence signal stabilized, the appropriate amount of each compound (6.4 mg mL^−1^ in DMSO, 8 × MIC) was added. DMSO and daptomycin (with 15 mM CaCl_2_) were used as negative and positive controls, respectively. Data were shown as the relative intensity with respect to the average fluorescence signal prior to the addition of each compound. All membrane lysis assays were performed two independent times (*n* = 2), and a representative fluorescence recording was presented.

##### Membrane Component Feeding Assay

The effects of bacterial membrane components on aquicidine L's or aquicidine C4's anti‐bacterial activities were evaluated using *E. coli* DH5α or *S. aureus* USA300. The general MIC assay described above was used by following the changes: different concentrations of LPS, PE, CL, PG, PC, UQ, and MK ranged from 2 to 128 µg mL^−1^ were mixed with aquicidine L or aquicidine C4. All experiments were done in triplicate (*n* = 3) and repeated two independent times (*n* = 2).

##### Isothermal Titration Calorimetry (ITC)

Calorimetric experiments were performed to evaluate the interaction between CL, PE, PG, or LPS and aquicidine L or aquicidine C4. To determine the affinity between aquicidine L or aquicidine C4 and CL, PE, or PG, both 2 mm CL, PE, or PG and 0.2 mm aquicidine L or aquicidine C4 were dissolved in 20 mm HEPES buffer (pH 7.5). To determine the interaction between LPS and aquicidine L or aquicidine C4, 2 mm LPS and 0.2 mm aquicidine L or aquicidine C4 were dissolved in H_2_O. Sequential injections of CL, PE, PG, or LPS into the calorimetric cell filled with aquicidine L or aquicidine C4 were repeated 20 times with equilibration intervals of 200 s. Data were collected by using a MicroCal PEAQ‐ITC instrument and calculated for the equilibrium dissociation constant (KD), change of enthalpy (ΔH), and change of entropy (ΔG).

##### Raising Resistant Mutants in Liquid Medium

Overnight cultures *S. aureus* USA300 were diluted 10000‐fold in LB broth, and 100 µL of dilute cell suspension was added to 96‐well plates containing 100 µL serially diluted aquicidine C4, telomycin, and daptomycin (with 15 mm CaCl_2_). Overnight cultures *K. pneumoniae* CRKP5452 were diluted 5000‐fold in LB broth, and 100 µL of dilute cell suspension was added to 96‐well plates containing 100 µL serially diluted aquicidine L and colistin. After an incubation of 24 h at 37 °C, the MIC value was determined. For the next round of assays, an aliquot from the overnight culture with the second‐highest antibiotic concentration that showed cloudy growth was diluted 10000‐ or 5000‐fold in LB broth and mixed with serially diluted antibiotics. The MIC value was recorded as described above, and the process was repeated daily for 21 days. Experiments were performed two independent times (*n* = 2).

##### Genome Sequencing of Antibiotic Resistant Mutants

Genomes were extracted from cultures of *S. aureus* USA300 or *K. pneumoniae* CRKP5452 colonies that showed an elevated MIC relative to the wild‐type, and sequenced by the MGI's DNBSEQ‐T7 platform. Single‐nucleotide polymorphisms (SNPs) for telomycin‐, daptomycin‐ or colistin‐resistant mutants were identified using SNIPPY by mapping DNBSEQ‐seq reads to the reference genome of *S. aureus* USA300 or *K. pneumoniae* CRKP5452.

##### Cytotoxicity Assay

The cytotoxicity of each synAQU peptide was assessed using a cell counting kit‐8 (CCK‐8) assay. HeLa cells were first seeded in a 96‐well flat‐bottom microtiter plate with a density of ∼5000 cells per well and incubated in the DMEM medium supplemented with 10% FBS at 37 °C with 5% CO_2_ for 24 h. Then, the DMEM medium was replaced with 100 µL of fresh DMEM medium with 10% FBS containing aquicidine L or aquicidine C4 at 10 serially diluted concentrations from 64 to 0.125 µg mL^−1^. After an incubation of 48 h at 37 °C with 5% CO_2_, 10 µL of a CCK‐8 solution was added into each well. After 2 h at 37 °C with 5% CO_2_, the OD_450_ value for each well was detected for measuring the cytotoxicity of aquicidine L or aquicidine C4 using a Tecan spark multimode microplate reader. 0.25% DMSO and taxol were used as the negative and positive, respectively. All cytotoxicity experiments were done in triplicate (*n* = 3) and repeated two independent times (*n* = 2).

##### Haemolytic Assay

A red blood cell diffusion assay was used to measure the haemolytic activities of aquicidine L and aquicidine C4. Aquicidine L and aquicidine C4 were dissolved in 10% DMSO with serially diluted concentrations from 100 to 12.5 µg mL^−1^. 10% (v/v) DMSO and 10% (v/v) Triton X‐100 were used as negative and positive controls, respectively. 15 µL of each serially diluted aquicidine L or aquicidine C4 was added to the surface of a 5% sheep blood agar plate and incubated at 20 °C for 24 h. The size of the transparent ring, which appeared in the blood agar plate was calculated to determine the haemolytic activity. The 5% sheep blood agar contains: 14 g/L of tryptone, 4.5 g/L of peptone, 4.5 g/L of yeast extract, 5.0 g/L of NaCl, 5% (v/v) of sterile defibrous sheep blood, and 12.5 g/L of Agar. Experiments were repeated two independent times (*n* = 2), and a representative picture was shown.

##### Acute Toxicity Assay

For acute toxicity assessment, three mice were given a single dose of aquicidine L or aquicidine C4 at 250 mg kg^−1^ via intraperitoneal injection. Mice were observed twice daily for mortality and morbidity as well as for possible signs of acute toxicity. The experiment was totally monitored for five days.

##### Mouse Peritonitis‐Sepsis Model

Six‐week‐old female ICR mice were used in all experiments and housed in a temperature‐controlled room (25 °C) with 12‐h light/dark cycle and 30% relative humidity. Mice were observed twice daily for mortality and morbidity. Abnormal clinical signs were recorded if observed and totally monitored for five days. The detailed methods for the mouse peritonitis‐sepsis model were as follows.

Meropenem‐resistant *A. baumannii *1104008 or *K. pneumoniae* CRKP 5452 was cultured in LB broth at 37 °C fro 16 h and diluted with 5% type II porcine stomach mucin and 0.9% NaCl. All mice were rendered neurotropic via intraperitoneal injection of cyclophosphamide at 150 mg/kg on day‐4 and day‐1 before infection. *A. baumannii *or *K. pneumoniae* cell cultures were diluted to provide a challenge inoculum of 5.0 × 10^7^ CFU in 0.5 mL and then administered into each mouse via intraperitoneal injection. 25 mice were randomly grouped into five per cohort (*n* = 5) and each cohort was given a single dose of either vehicle (20% solutol), aquicidine L at 12.5, 25, or 50 mg kg^−1^, and colistin at 12.5 mg kg^−1^ 1 h after infection via subcutaneous injection and repeated every 12 h up to 36 h (totally four injections).

Vancomycin‐resistant *E. faecium* 35682 was cultured in BHI broth at 37 °C fro 16 h and diluted with 5% type II porcine stomach mucin and 0.9% NaCl. *E. faecium* cell cultures were diluted to provide a challenge inoculum of 4.0 × 10^9^ CFU in 0.5 mL and then administered into each mouse via intraperitoneal injection. 25 mice were randomly grouped into five per cohort (*n* = 5) and each cohort was given a single dose of either vehicle (20% solutol), aquicidine C4 at 50, 100 or 150 mg/kg and daptomycin at 100 mg kg^−1^ with 15 mm CaCl_2_ 1 h after infection via subcutaneous injection.

Methicillin‐resistant *S. aureus* USA300 was cultured in LB broth at 37 °C for 16 h and diluted with 7% type II porcine stomach mucin with 0.2 mm FeNH_4_‐citrate. *S. aureus* cell cultures were diluted to provide a challenge inoculum of 2.5 × 10^8^ CFU in 0.5 mL and then administered into each mouse via intraperitoneal injection. 25 mice were randomly grouped into five per cohort (*n* = 5) and each cohort was given a single dose of either vehicle (20% solutol), aquicidine C4 at 50, 100, or 150 mg kg^−1^, and vancomycin at 50 mg kg^−1^ 1 hr after infection via subcutaneous injection.

##### Neutropenic Thigh Infection Model

Six‐week‐old female ICR mice were used in all experiments and housed in a temperature‐controlled room (25 °C) with 12‐h light/dark cycle and 30% relative humidity. Meropenem‐resistant *A. baumannii* 1104008 or methicillin‐resistant *S. aureus* USA300 was cultured in the MH broth containing 50 µg mL^−1^ of gentamycin and the MH broth, respectively, at 37 °C and 200 rpm overnight. Then, the strains *A. baumannii* 1104008 or *S. aureus* USA300 were washed twice in 0.9% saline and further diluted to verify the bacterial suspension with a challenge inoculum of ≈1.0 × 10^6^ CFU in a volume of 0.05 mL per mouse thigh. Mice were rendered neutropenic by receiving 150 and 100 mg kg^−1^ of cyclophosphamide via intraperitoneal injection on day‐4 and day‐1 before infection, respectively. For treating the thigh infection caused by *A. baumannii* 1104008, mice were given 100 µL of vehicle (20% solutol), colistin (25 mg kg^−1^, 20% solutol), or aquicidine L (25 mg kg^−1^, 20% solutol) at 2, 8, 14, and 20 h after post‐infection via subcutaneous injection. For treating the thigh infection caused by *S. aureus* USA300, mice were given 200 µL of vehicle (20% solutol), vancomycin (50 mg kg^−1^, 20% solutol) or aquicidine L (50 mg kg^−1^, 20% solutol) at 2, 10, and 18 h post‐infection via subcutaneous injection. At 2 h after infection, mice in the untreated control infection group were euthanized to determine the starting thigh bacterial burden (*n*  =  4 mice, *n*  =  8 thighs). All mice were observed after infection for morbidity, and any abnormal signs were recorded. Mice were euthanized at the experimental endpoint of 24 h after infection (*n*  =  4 mice, *n*  =  8 thighs). Finally, thigh muscles were removed, weighed, homogenized, and enumerated to count CFUs for bacterial burden after plating on MH agar containing 50 µg mL^−1^ of gentamicin for *A. baumannii* 1104008 or MH agar for *S. aureus* USA300. The treatment efficacies of aquicidine L or C4 were determined as the bacterial burden reduction in the thighs relative to both the vehicle and colistin‐ or vancomycin‐treated controls. All graphic data were analyzed using the GraphPad Prism software (Prism10).

##### Prediction of Secondary Structures for Aquicidine L (Lys‐Substituted) and Aquicidine C4 (Lys‐Substituted)

The peptide backbones of aquicidine L or aquicidine C4 were modeled by substituting non‐canonical residues (L‐Dap, L‐Dab, and L‐Orn) with L‐Lys. The initial structures of aquicidine L (Lys‐substituted) or aquicidine C4 (Lys‐substituted) were predicted were generated using the software ColabFold and AfCycDesign, respectively, followed by the manual attachment of a tetradecanoic acid lipid chain in the software PyMOL. The final secondary structures of aquicidine L (Lys‐substituted) or aquicidine C4 (Lys‐substituted) were energy‐minimized using the software RDKit (MMFF94 force field, 2 0000 iterations) for the optimization of their geometries. The helices, loops, and lipid tails of their secondary structures were highlighted to illustrate key features.

##### Ethics Statement

The Shanghai Public Health Clinical Center Animal Care and Use Committee approved all animal procedures under the protocol 2024‐A002‐02. We have complied with all relevant ethical regulations for animal use.

##### Statistical Analysis

For each experiment, three independent biological replicates (*n* = 3) were used unless stated otherwise. Data were shown as mean values ± s.e.m and no data were excluded from the statistical analyses. No specific statistical approach was used to predetermine sample size. All experiments were not randomized except for the mouse model.

## Conflict of interest

The authors declare no conflict of interest.

## Supporting information



Supporting Information

## Data Availability

The data that support the findings of this study are available in the supplementary material of this article.
