# Supplementary material for: Identification of a Pair of Linear or Cyclic Naturally Inspired Bifunctional Lipopeptide Antibiotics That Overcome Antimicrobial Resistance
Source: Adv Sci (Weinh). 2025 Sep 11;12(45):e09796. doi: 10.1002/advs.202509796 (PMC12677607; doi:10.1002/advs.202509796)
Supplement: Supplementary file 1 — Supporting Information [file ADVS-12-e09796-s001.pdf]

Identification of a Pair of Linear or Cyclic Naturally Inspired Bifunctional Lipopeptide Antibiotics That Overcome Antimicrobial Resistance

Lei Li*, Yiwen Zhou, Yuzhu Wu

State Key Laboratory of Microbial Metabolism and School of Life Sciences and Biotechnology
Shanghai Jiao Tong University,
800 Dongchuan Rd., Shanghai, 200240, China
E-mail: *lei.li@sjtu.edu.cn

Table of Contents:

1. Table S1: Predicted linear peptide sequences of the seven selected petides synAQUs 1-7

2. Table S2: Predicted monomer building blocks used by synAQU1

3. Table S3: Predicted monomer building blocks used by synAQU2

4. Table S4: Predicted monomer building blocks used by synAQU3

5. Table S5: Predicted monomer building blocks used by synAQU4

6. Table S6: Predicted monomer building blocks used by synAQU5

7. Table S7: Predicted monomer building blocks used by synAQU6

8. Table S8: Predicted monomer building blocks used by synAQU7

9. Table S9: Comparsion of peptide sequences of pedopeptin B and synAQU3

10. Table S10: High-resolution mass spectrometry (HRMS) (ES+) data for all compounds synthesized in this study

11. Table S11: Activities of seven selected synAQUs against microorganisms (MIC: μg/mL)

12. Table S12: Summary of change of enthalpy (ΔH) and change of entropy (ΔG) in isothermal titration

13. Figure S1: Known natural products from *Aquimarina*

14. Figure S2. The distribution of natural product BGCs in 76 *Aquimarina* strains

15. Figure S3: The AQU1 biosynthetic gene cluster analysis

16. Figure S4: The AQU2 biosynthetic gene cluster analysis

17. Figure S5: The AQU3 biosynthetic gene cluster analysis

18. Figure S6: The AQU4 biosynthetic gene cluster analysis

19. Figure S7: The AQU5 biosynthetic gene cluster analysis

20. Figure S8: The AQU6 biosynthetic gene cluster analysis

21. Figure S9: The AQU7 biosynthetic gene cluster analysis

22. Figure S10: The aquimarin biosynthetic gene cluster analysis

23. Figure S11: Predicted structures and sequence resources of all compounds synthesized in this study

24. Figure S12: HRMS spectrum of synAQU1-L

25. Figure S13: HRMS spectra of synAQU2-L, synAQU2-C2 and synAQU2-C3

26. Figure S14: HRMS spectra of synAQU3-L, synAQU3-C1, synAQU3-C3, synAQU3-C4 and synAQU3-cFA

27. Figure S15: HRMS spectrum of synAQU4-L

28. Figure S16: HRMS spectra of aquicidine L (synAQU5-L), synAQU5-C2 and aquicidine C4 (synAQU5-C4)

29. Figure S17: HRMS spectra of synAQU6-L and synAQU6-C4

30. Figure S18: HRMS spectra of synAQU7-L and synAQU7-C6

31. Figure S19: Structures of known paired ring-opening or -closing non-ribosomal peptides

32. Figure S20: Bioactivities of known paired ring-opening or -closing non-ribosomal peptides

33. Figure S21: ^{1}H NMR spectrum of synAQU1-L in DMSO-d_6 (600 MHz)

34. Figure S22: ^{13}C NMR spectrum of synAQU1-L in DMSO-d_6 (150 MHz)

35. Figure S23: DEPT135 NMR spectrum of synAQU1-L in DMSO-d_6 (150 MHz)

36. Figure S24: ^{1}H-^{1}H COSY NMR spectrum of synAQU1-L in DMSO-d_6 (600 MHz)

37. Figure S25: ^{1}H-^{13}C HSQC NMR spectrum of synAQU1-L in DMSO-d_6 (600 MHz)

38. Figure S26: ^{1}H-^{13}C HMBC NMR spectrum of synAQU1-L in DMSO-d_6 (600 MHz)

39. Figure S27: ^{1}H NMR spectrum of synAQU2-L in DMSO-d_6 (600 MHz)

40. Figure S28: ^{13}C NMR spectrum of synAQU2-L in DMSO-d_6 (150 MHz)

41. Figure S29: DEPT135 NMR spectrum of synAQU2-L in DMSO-d_6 (150 MHz)

42. Figure S30: ^{1}H-^{1}H COSY NMR spectrum of synAQU2-L in DMSO-d_6 (600 MHz)

43. Figure S31: ^{1}H-^{13}C HSQC NMR spectrum of synAQU2-L in DMSO-d_6 (600 MHz)

44. Figure S32: ^{1}H-^{13}C HMBC NMR spectrum of synAQU2-L in DMSO-d_6 (600 MHz)

45. Figure S33: ^{1}H NMR spectrum of synAQU2-C2 in DMSO-d_6 (600 MHz)

46. Figure S34: ^{13}C NMR spectrum of synAQU2-C2 in DMSO-d_6 (150 MHz)

47. Figure S35: DEPT135 NMR spectrum of synAQU2-C2 in DMSO-d_6 (150 MHz)

48. Figure S36: ^{1}H-^{1}H COSY NMR spectrum of synAQU2-C2 in DMSO-d_6 (600 MHz)

49. Figure S37: ^{1}H-^{13}C HSQC NMR spectrum of synAQU2-C2 in DMSO-d_6 (600 MHz)

50. Figure S38: ^{1}H-^{13}C HMBC NMR spectrum of synAQU2-C2 in DMSO-d_6 (600 MHz)

51. Figure S39: ^{1}H NMR spectrum of synAQU2-C3 in DMSO-d_6 (600 MHz)

52. Figure S40: ^{13}C NMR spectrum of synAQU2-C3 in DMSO-d_6 (150 MHz)

53. Figure S41: DEPT135 NMR spectrum of synAQU2-C3 in DMSO-d_6 (150 MHz)

54. Figure S42: ^{1}H-^{1}H COSY NMR spectrum of synAQU2-C3 in DMSO-d_6 (600 MHz)

55. Figure S43: ^{1}H-^{13}C HSQC NMR spectrum of synAQU2-C3 in DMSO-d_6 (600 MHz)

56. Figure S44: ^{1}H-^{13}C HMBC NMR spectrum of synAQU2-C3 in DMSO-d_6 (600 MHz)

57. Figure S45: ^{1}H NMR spectrum of synAQU3-L in DMSO-d_6 (600 MHz)

58. Figure S46: ^{13}C NMR spectrum of synAQU3-L in DMSO-d_6 (150 MHz)

59. Figure S47: DEPT135 NMR spectrum of synAQU3-L in DMSO-d_6 (150 MHz)

60. Figure S48: ^{1}H-^{1}H COSY NMR spectrum of synAQU3-L in DMSO-d_6 (600 MHz)

61. Figure S49: ^{1}H-^{13}C HSQC NMR spectrum of synAQU3-L in DMSO-d_6 (600 MHz)

62. Figure S50: ^{1}H-^{13}C HMBC NMR spectrum of synAQU3-L in DMSO-d_6 (600 MHz)

63. Figure S51: ^{1}H NMR spectrum of synAQU3-C1 in DMSO-d_6 (600 MHz)

64. Figure S52: ^{13}C NMR spectrum of synAQU3-C1 in DMSO-d_6 (150 MHz)

65. Figure S53: DEPT135 NMR spectrum of synAQU3-C1 in DMSO-d_6 (150 MHz)

66. Figure S54: ^{1}H-^{1}H COSY NMR spectrum of synAQU3-C1 in DMSO-d_6 (600 MHz)

67. Figure S55: ^{1}H-^{13}C HSQC NMR spectrum of synAQU3-C1 in DMSO-d_6 (600 MHz)

68. Figure S56: ^{1}H-^{13}C HMBC NMR spectrum of synAQU3-C1 in DMSO-d_6 (600 MHz)

69. Figure S57: ^{1}H NMR spectrum of synAQU3-C3 in DMSO-d_6 (600 MHz)

70. Figure S58: ^{13}C NMR spectrum of synAQU3-C3 in DMSO-d_6 (150 MHz)

71. Figure S59: DEPT135 NMR spectrum of synAQU3-C3 in DMSO-d_6 (150 MHz)

72. Figure S60: ^{1}H-^{1}H COSY NMR spectrum of synAQU3-C3 in DMSO-d_6 (600 MHz)

73. Figure S61: ^{1}H-^{13}C HSQC NMR spectrum of synAQU3-C3 in DMSO-d_6 (600 MHz)

74. Figure S62: ^{1}H-^{13}C HMBC NMR spectrum of synAQU3-C3 in DMSO-d_6 (600 MHz)

75. Figure S63: ^{1}H NMR spectrum of synAQU3-C4 in DMSO-d_6 (600 MHz)

76. Figure S64: ^{13}C NMR spectrum of synAQU3-C4 in DMSO-d_6 (150 MHz)

77. Figure S65: DEPT135 NMR spectrum of synAQU3-C4 in DMSO-d_6 (150 MHz)

78. Figure S66: ^{1}H-^{1}H COSY NMR spectrum of synAQU3-C4 in DMSO-d_6 (600 MHz)

79. Figure S67: ^{1}H-^{13}C HSQC NMR spectrum of synAQU3-C4 in DMSO-d_6 (600 MHz)

80. Figure S68: ^{1}H-^{13}C HMBC NMR spectrum of synAQU3-C4 in DMSO-d_6 (600 MHz)

81. Figure S69: ^{1}H NMR spectrum of synAQU3-cFA in DMSO-d_6 (600 MHz)

82. Figure S70: ^{13}C NMR spectrum of synAQU3-cFA in DMSO-d_6 (150 MHz)

83. Figure S71: DEPT135 NMR spectrum of synAQU3-cFA in DMSO-d_6 (150 MHz)

84. Figure S72: ^{1}H-^{1}H COSY NMR spectrum of synAQU3-cFA in DMSO-d_6 (600 MHz)

85. Figure S73: ^{1}H-^{13}C HSQC NMR spectrum of synAQU3-cFA in DMSO-d_6 (600 MHz)

86. Figure S74: ^{1}H-^{13}C HMBC NMR spectrum of synAQU3-cFA in DMSO-d_6 (600 MHz)

87. Figure S75: ^{1}H NMR spectrum of synAQU4-L in DMSO-d_6 (600 MHz)

88. Figure S76: ^{13}C NMR spectrum of synAQU4-L in DMSO-d_6 (150 MHz)

89. Figure S77: DEPT135 NMR spectrum of synAQU4-L in DMSO-d_6 (150 MHz)

90. Figure S78: ^{1}H-^{1}H COSY NMR spectrum of synAQU4-L in DMSO-d_6 (600 MHz)

91. Figure S79: ^{1}H-^{13}C HSQC NMR spectrum of synAQU4-L in DMSO-d_6 (600 MHz)

92. Figure S80: ^{1}H-^{13}C HMBC NMR spectrum of synAQU4-L in DMSO-d_6 (600 MHz)

93. Figure S81: ^{1}H NMR spectrum of aquicidine L (synAQU5-L) in DMSO-d_6 (600 MHz)

94. Figure S82: ^{13}C NMR spectrum of aquicidine L (synAQU5-L) in DMSO-d_6 (150 MHz)

95. Figure S83: DEPT135 NMR spectrum of aquicidine L (synAQU5-L) in DMSO-d_6 (150 MHz)

96. Figure S84: ^{1}H-^{1}H COSY NMR spectrum of aquicidine L (synAQU5-L) in DMSO-d_6 (600 MHz)

97. Figure S85: ^{1}H-^{13}C HSQC NMR spectrum of aquicidine L (synAQU5-L) in DMSO-d_6 (600 MHz)

98. Figure S86: ^{1}H-^{13}C HMBC NMR spectrum of aquicidine L (synAQU5-L) in DMSO-d_6 (600 MHz)

99. Figure S87: ^{1}H NMR spectrum of synAQU5-C2 in DMSO-d_6 (600 MHz)

100. Figure S88: ^{13}C NMR spectrum of synAQU5-C2 in DMSO-d_6 (150 MHz)

101. Figure S89: DEPT135 NMR spectrum of synAQU5-C2 in DMSO-d_6 (150 MHz)

102. Figure S90: ^{1}H-^{1}H COSY NMR spectrum of synAQU5-C2 in DMSO-d_6 (600 MHz)

103. Figure S91: ^{1}H-^{13}C HSQC NMR spectrum of synAQU5-C2 in DMSO-d_6 (600 MHz)

104. Figure S92: ^{1}H-^{13}C HMBC NMR spectrum of synAQU5-C2 in DMSO-d_6 (600 MHz)

105. Figure S93: ^{1}H NMR spectrum of aquicidine C4 (synAQU5-C4) in DMSO-d_6 (600 MHz)

106. Figure S94: ^{13}C NMR spectrum of aquicidine C4 (synAQU5-C4) in DMSO-d_6 (150 MHz)

107. Figure S95: DEPT135 NMR spectrum of aquicidine C4 (synAQU5-C4) in DMSO-d_6 (150 MHz)

108. Figure S96: ^{1}H-^{1}H COSY NMR spectrum of aquicidine C4 (synAQU5-C4) in DMSO-d_6 (600 MHz)

109. Figure S97: ^{1}H-^{13}C HSQC NMR spectrum of aquicidine C4 (synAQU5-C4) in DMSO-d_6 (600 MHz)

110. Figure S98: ^{1}H-^{13}C HMBC NMR spectrum of aquicidine C4 (synAQU5-C4) in DMSO-d_6 (600 MHz)

111. Figure S99: ^{1}H NMR spectrum of synAQU6-L in DMSO-d_6 (600 MHz)

112. Figure S100: ^{13}C NMR spectrum of synAQU6-L in DMSO-d_6 (150 MHz)

113. Figure S101: DEPT135 NMR spectrum of synAQU6-L in DMSO-d_6 (150 MHz)

114. Figure S102: ^{1}H-^{1}H COSY NMR spectrum of synAQU6-L in DMSO-d_6 (600 MHz)

115. Figure S103: ^{1}H-^{13}C HSQC NMR spectrum of synAQU6-L in DMSO-d_6 (600 MHz)

116. Figure S104: ^{1}H-^{13}C HMBC NMR spectrum of synAQU6-L in DMSO-d_6 (600 MHz)

117. Figure S105: ^{1}H NMR spectrum of synAQU6-C4 in DMSO-d_6 (600 MHz)

118. Figure S106: ^{13}C NMR spectrum of synAQU6-C4 in DMSO-d_6 (150 MHz)

119. Figure S107: DEPT135 NMR spectrum of synAQU6-C4 in DMSO-d_6 (150 MHz)

120. Figure S108: ^{1}H-^{1}H COSY NMR spectrum of synAQU6-C4 in DMSO-d_6 (600 MHz)

121. Figure S109: ^{1}H-^{13}C HSQC NMR spectrum of synAQU6-C4 in DMSO-d_6 (600 MHz)

122. Figure S110: ^{1}H-^{13}C HMBC NMR spectrum of synAQU6-C4 in DMSO-d_6 (600 MHz)

123. Figure S111: ^{1}H NMR spectrum of synAQU7-L in DMSO-d_6 (600 MHz)

124. Figure S112: ^{13}C NMR spectrum of synAQU7-L in DMSO-d_6 (150 MHz)

125. Figure S113: DEPT135 NMR spectrum of synAQU7-L in DMSO-d_6 (150 MHz)

126. Figure S114: ^{1}H-^{1}H COSY NMR spectrum of synAQU7-L in DMSO-d_6 (600 MHz)

127. Figure S115: ^{1}H-^{13}C HSQC NMR spectrum of synAQU7-L in DMSO-d_6 (600 MHz)

128. Figure S116: ^{1}H-^{13}C HMBC NMR spectrum of synAQU7-L in DMSO-d_6 (600 MHz)

129. Figure S117: ^{1}H NMR spectrum of synAQU7-C6 in DMSO-d_6 (600 MHz)

130. Figure S118: ^{13}C NMR spectrum of synAQU7-C6 in DMSO-d_6 (150 MHz)

131. Figure S119: DEPT135 NMR spectrum of synAQU7-C6 in DMSO-d_6 (150 MHz)

132. Figure S120: ^{1}H-^{1}H COSY NMR spectrum of synAQU7-C6 in DMSO-d_6 (600 MHz)

133. Figure S121: ^{1}H-^{13}C HSQC NMR spectrum of synAQU7-C6 in DMSO-d_6 (600 MHz)

134. Figure S122: ^{1}H-^{13}C HMBC NMR spectrum of synAQU7-C6 in DMSO-d_6 (600 MHz)

135. Figure S123: The bactericidal effects against *E. coli* DH5α (a) and the membrane lysis effects on *S. aureus* USA300 (b) of aquicidine L and C4

136. Figure S124: Scanning electron microscopy image of *S. aureus* USA300 cultures treated with aquicidine L or C4

137. Figure S125: The antibacterial activities of aquicidine L and aquicidine C4 in the presence of MK, UQ or PC

138. Figure S126: Isothermal titration of CL, PG, LPS or PE into the buffer (20 mM HEPES buffer or ddH_2O)

139. Figure S127: The antibacterial activities of aquicidine L or C4 against drug-resistant pathogens

140. Figure S128: Cytotoxicities, haemolytic activities and *in vivo* acute toxicities of aquicidine L and aquicidine C4

141. Figure S129: Predicted secondary structures of aquicidine L (Lys-substituted) and aquicidine C4 (Lys-substituted)

Table S1. Predicted linear peptide sequences of the seven selected petides synAQUs 1-7

Peptides	A1	A2	A3	A4	A5	A6	A7	A8	A9	A10	A11	A12	A13
**synAQU1**	D-Ala	D-Ala	D-Phe	L-Thr	L-Arg								
**synAQU2**	L-Phe	L-Orn	L-Orn	D-Val	L-Ile	L-Leu	D-Tyr	L-Dab	Gly				
**synAQU3**	L-Dap	L-Phe	L-Dab	L-Thr	D-Phe	L-Dap	D-Leu	L-Val	L-Asp				
**synAQU4**	L-Val	L-Ala	D-Ala	L-Ile	L-Ile	β-Ala	L-Leu	D-Ala	L-Ile	L-Tyr			
**synAQU5**	L-Dap	L-Thr	L-Leu	L-Ser	L-Dab	L-Dap	L-Phe	L-Ile	L-Orn	L-Asn	L-Phe		
**synAQU6**	L-Orn	D-Tyr	D-Val	D-Thr	D-Orn	L-Orn	D-Val	L-Ile	L-Leu	D-Orn	L-Thr	L-Ala	
**synAQU7**	L-Lys	L-Arg	L-Leu	L-Ala	L-Leu	L-Thr	L-Asp	L-Asp	L-Val	L-Asn	L-Val	D-Phe	L-Asn

Table S2. Predicted monomer building blocks used by synAQU1. Four A-domain substrate prediction sources, including Nrpys, SVM, Stachelhaus and our in-house manual examination of characterized BGCs, were used to predict the substrate of each A-domain. The Stachelhaus code that consists of 10 A-domain active site residues (positions 235, 236, 239, 278, 299, 301, 322, 330, 331 and 517) are shown for each A-domain.

	235	236	239	278	299	301	322	330	331	517	Nrpys	SVM	Stachelhaus	In-house	Final version
A1	D	V	H	N	H	A	V	I	Y	K	Ala	NA	Ala	Ala	D-Ala
A2	D	V	H	N	H	A	V	I	Y	K	Ala	NA	Ala	Ala	D-Ala
A3	D	A	F	T	I	A	E	V	A	K	Tyr	Tyr	Phe	Phe	D-Phe
A4	D	F	W	N	I	G	M	V	H	K	Thr	Thr	Thr	Thr	L-Thr
A5	D	A	E	D	V	G	A	I	T	K	Arg	NA	Arg	Arg	L-Arg

Table S3. Predicted monomer building blocks used by synAQU2. Four A-domain substrate prediction sources, including Nrpys, SVM, Stachelhaus and our in-house manual examination of characterized BGCs, were used to predict the substrate of each A-domain. The Stachelhaus code that consists of 10 A-domain active site residues (positions 235, 236, 239, 278, 299, 301, 322, 330, 331 and 517) are shown for each A-domain.

	235	236	239	278	299	301	322	330	331	517	Nrpys	SVM	Stachelhaus	In-house	Final version
A1	D	A	W	T	I	A	A	I	C	K	Phe	Phe	Phe	Phe	L-Phe
A2	D	A	G	E	N	G	S	V	D	K	Orn	Orn	Orn	Orn	L-Orn
A3	D	V	G	E	I	G	S	I	D	K	Orn	Orn	Orn	Orn	L-Orn
A4	D	A	F	W	L	G	G	T	F	K	Val	Val	Val	Val	D-Val
A5	D	G	Y	F	L	G	V	V	Y	K	Ile	Ile	Ile	Ile	L-Ile
A6	D	A	W	Y	L	G	N	V	V	K	Leu	Leu	Leu	Leu	L-Leu
A7	D	A	S	T	V	A	A	I	C	K	Tyr	Tyr	Tyr	Tyr	D-Tyr
A8	D	I	W	Q	L	T	A	D	D	K	NA	NA	Dab	Dab	L-Dab
A9	D	I	L	Q	L	C	L	I	W	K	Gly	Gly	Gly	Gly	Gly

Table S4. Predicted monomer building blocks used by synAQU3. Four A-domain substrate prediction sources, including Nrpys, SVM, Stachelhaus and our in-house manual examination of characterized BGCs, were used to predict the substrate of each A-domain. The Stachelhaus code that consists of 10 A-domain active site residues (positions 235, 236, 239, 278, 299, 301, 322, 330, 331 and 517) are shown for each A-domain.

	235	236	239	278	299	301	322	330	331	517	Nrpys	SVM	Stachelhaus	In-house	Final version
A1	D	I	W	Q	I	T	A	D	D	K	Asp	NA	Dap	Dap	L-Dap
A2	D	A	W	T	I	A	A	I	C	K	Phe	Phe	Phe	Phe	L-Phe
A3	D	I	W	Q	L	T	A	D	D	K	NA	NA	Dab	Dab	L-Dab
A4	D	F	W	N	I	G	M	V	H	K	Thr	Thr	Thr	Thr	L-Thr
A5	D	A	W	T	I	A	A	I	C	K	Phe	Phe	Phe	Phe	D-Phe
A6	D	I	W	Q	I	T	A	D	D	K	NA	NA	Dap	Dap	L-Dap
A7	D	A	W	Y	L	G	N	V	V	K	Leu	Leu	Leu	Leu	D-Leu
A8	D	A	I	W	I	G	G	T	F	K	Val	Val	Val	Val	L-Val
A9	D	L	T	K	I	G	H	I	G	K	Asp	Asp	Asp	Asp	L-Asp

Table S5. Predicted monomer building blocks used by synAQU4. Four A-domain substrate prediction sources, including Nrpys, SVM, Stachelhaus and our in-house manual examination of characterized BGCs, were used to predict the substrate of each A-domain. The Stachelhaus code that consists of 10 A-domain active site residues (positions 235, 236, 239, 278, 299, 301, 322, 330, 331 and 517) are shown for each A-domain.

	235	236	239	278	299	301	322	330	331	517	Nrpys	SVM	Stachelhaus	In-house	Final version
A1	D	I	W	Q	I	T	A	D	D	K	Asp	NA	Dap	Dap	L-Dap
A2	D	A	W	T	I	A	A	I	C	K	Phe	Phe	Phe	Phe	L-Phe
A3	D	I	W	Q	L	T	A	D	D	K	NA	NA	Dab	Dab	L-Dab
A4	D	F	W	N	I	G	M	V	H	K	Thr	Thr	Thr	Thr	L-Thr
A5	D	A	W	T	I	A	A	I	C	K	Phe	Phe	Phe	Phe	D-Phe
A6	D	I	W	Q	I	T	A	D	D	K	NA	NA	Dap	Dap	L-Dap
A7	D	A	W	Y	L	G	N	V	V	K	Leu	Leu	Leu	Leu	D-Leu
A8	D	A	I	W	I	G	G	T	F	K	Val	Val	Val	Val	L-Val
A9	D	L	T	K	I	G	H	I	G	K	Asp	Asp	Asp	Asp	L-Asp

Table S6. Predicted monomer building blocks used by synAQU5. Four A-domain substrate prediction sources, including Nrpys, SVM, Stachelhaus and our in-house manual examination of characterized BGCs, were used to predict the substrate of each A-domain. The Stachelhaus code that consists of 10 A-domain active site residues (positions 235, 236, 239, 278, 299, 301, 322, 330, 331 and 517) are shown for each A-domain.

	235	236	239	278	299	301	322	330	331	517	Nrpys	SVM	Stachelhaus	In-house	Final version
A1	D	I	W	E	M	V	A	D	D	K	NA	NA	Dap	Dap	L-Dap
A2	D	F	W	N	I	G	M	V	H	K	Thr	Thr	Thr	Thr	L-Thr
A3	D	A	W	Y	L	G	N	V	V	K	Leu	Leu	Leu	Leu	L-Leu
A4	D	V	W	H	L	S	L	I	D	K	Ser	Ser	Ser	Ser	L-Ser
A5	D	I	W	Q	L	T	A	D	D	K	NA	NA	Dab	Dab	L-Dab
A6	D	I	W	E	M	V	A	D	D	K	Dap	Dap	Dap	Dap	L-Dap
A7	D	A	W	T	I	A	A	I	C	K	Phe	Phe	Phe	Phe	L-Phe
A8	D	G	Y	F	L	G	V	V	Y	K	Ile	Ile	Ile	Ile	L-Ile
A9	D	V	G	E	I	G	S	I	D	K	Orn	Orn	Orn	Orn	L-Orn
A10	D	L	T	K	I	G	E	V	G	K	Asn	Asn	Asn	Asn	L-Asn
A11	D	A	W	T	V	A	A	I	C	K	Phe	Phe	Phe	Phe	L-Phe

Table S7. Predicted monomer building blocks used by synAQU6. Four A-domain substrate prediction sources, including Nrpys, SVM, Stachelhaus and our in-house manual examination of characterized BGCs, were used to predict the substrate of each A-domain. The Stachelhaus code that consists of 10 A-domain active site residues (positions 235, 236, 239, 278, 299, 301, 322, 330, 331 and 517) are shown for each A-domain.

	235	236	239	278	299	301	322	330	331	517	Nrpys	SVM	Stachelhaus	In-house	Final version
A1	D	V	G	E	V	G	S	I	D	K	Orn	Orn	Orn	Orn	L-Orn
A2	D	A	Y	T	I	A	A	V	C	K	Tyr	Tyr	Tyr	Tyr	D-Tyr
A3	D	A	I	W	I	G	G	T	F	K	Val	Val	Val	Val	D-Val
A4	D	F	W	N	T	G	M	V	H	K	Thr	Thr	Thr	Thr	D-Thr
A5	D	I	G	E	I	G	S	V	D	K	Orn	Orn	Orn	Orn	D-Orn
A6	D	V	G	E	S	G	S	V	D	K	Orn	Orn	Orn	Orn	L-Orn
A7	D	A	I	W	L	G	G	T	F	K	Val	Val	Val	Val	D-Val
A8	D	G	F	F	L	G	V	V	Y	K	Ile	Ile	Ile	Ile	L-Ile
A9	D	A	W	F	L	G	N	V	V	K	Leu	Leu	Leu	Leu	L-Leu
A10	D	V	S	E	I	G	S	I	D	K	Orn	Orn	Orn	Orn	D-Orn
A11	D	F	W	N	I	G	M	V	H	K	Thr	Thr	Thr	Thr	L-Thr
A12	D	V	P	N	C	C	I	V	Y	K	Ala	Ala	Ala	Ala	L-Ala

Table S8. Predicted monomer building blocks used by synAQU7. Four A-domain substrate prediction sources, including Nrpys, SVM, Stachelhaus and our in-house manual examination of characterized BGCs, were used to predict the substrate of each A-domain. The Stachelhaus code that consists of 10 A-domain active site residues (positions 235, 236, 239, 278, 299, 301, 322, 330, 331 and 517) are shown for each A-domain.

	235	236	239	278	299	301	322	330	331	517	Nrpys	SVM	Stachelhaus	In-house	Final version
A1	D	T	E	S	I	G	T	V	C	K	Lys	NA	Lys	Lys	L-Lys
A2	D	A	E	H	V	G	K	I	S	K	Asp	NA	Arg	Arg	L-Arg
A3	D	A	W	Y	L	G	N	V	V	K	Leu	Leu	Leu	Leu	L-Leu
A4	D	L	Y	N	N	A	L	T	Y	K	Ala	Ala	Ala	Ala	L-Ala
A5	D	A	W	Y	L	G	N	V	V	K	Leu	Leu	Leu	Leu	L-Leu
A6	D	F	W	S	I	G	M	V	H	K	Thr	Thr	Thr	Thr	L-Thr
A7	D	A	R	H	L	A	L	L	V	K	NA	NA	Asp	Asp	L-Asp
A8	D	L	T	K	L	G	H	I	G	K	Asp	Asp	Asp	Asp	L-Asp
A9	D	A	F	W	I	G	G	T	F	K	Val	Val	Val	Val	L-Val
A10	D	L	T	K	I	G	E	V	G	K	Asn	Asn	Asn	Asn	L-Asn
A11	D	A	F	W	L	G	A	T	F	K	Val	Val	Val	Val	L-Val
A12	D	A	W	T	I	A	A	I	C	K	Phe	Phe	Phe	Phe	D-Phe
A13	D	V	T	K	L	G	S	V	A	K	Asn	NA	Asn	Asn	L-Asn

Table S9. Comparsion of peptide sequences of pedopeptin B and synAQU3

Compound	A1	A2	A3	A4	A5	A6	A7	A8	A9	Starting domain	Fatty acid tail
**Pedopeptin B**	L-Dap	L-Phe	L-Dab	L-Thr	L-Leu	L-Dap	D-Leu	L-Val	L-Asp	Cs domain	(*R*)-3-hydroxy-octanoic acid
**synAQU3**	L-Dap	L-Phe	L-Dab	L-Thr	D-Phe	L-Dap	D-Leu	L-Val	L-Asp	PKS domain	(*R*)-3-hydroxy-octanoic acid

Table S10. High-resolution mass spectrometry (HRMS) (ES+) data for all compounds synthesized in this study

Name	Molecular formula	Calc m/z	Obsd m/z	Difference in ppm
synAQU1-L	$C_{39}H_{66}N_8O_8$	775.5076	775.5065	-1.42
synAQU2-L	$C_{65}H_{108}N_{12}O_{12}$	1249.8282	1249.8220	-4.97
synAQU2-C2	$C_{65}H_{106}N_{12}O_{11}$	1231.8177	1231.8133	-3.57
synAQU2-C3	$C_{65}H_{106}N_{12}O_{11}$	1231.8177	1231.8141	-2.92
synAQU3-L	$C_{55}H_{86}N_{12}O_{15}$	1155.6408	1155.6387	-1.82
synAQU3-C1	$C_{55}H_{84}N_{12}O_{14}$	1137.6303	1137.6285	-1.58
synAQU3-C3	$C_{55}H_{84}N_{12}O_{14}$	1137.6303	1137.6286	-1.49
synAQU3-C4	$C_{55}H_{84}N_{12}O_{14}$	1137.6303	1137.6279	-2.11
synAQU3-cFA	$C_{55}H_{84}N_{12}O_{14}$	1137.6303	1137.6280	-2.02
synAQU4	$C_{56}H_{97}N_{11}O_{12}$	1116.7391	1116.7391	0
Aquicidine L (synAQU5-L)	$C_{70}H_{116}N_{16}O_{16}$	1437.8828	1437.8797	-2.16
synAQU5-C2	$C_{70}H_{113}N_{16}O_{15}Na$	1441.8542	1441.8492	-3.47
Aquicidine C4 (synAQU5-C4)	$C_{70}H_{113}N_{16}O_{15}Na$	1441.8542	1441.8497	-3.12
synAQU6-L	$C_{76}H_{136}N_{16}O_{17}$	1546.0342	1546.0302	-2.59
synAQU6-C4	$C_{76}H_{134}N_{16}O_{16}$	1528.0236	1528.0203	-2.16
synAQU7-L	$C_{80}H_{135}N_{19}O_{22}$	1715.9902	1715.9859	-2.50
synAQU7-C6	$C_{80}H_{133}N_{19}O_{21}$	1696.9996	1696.9921	-4.42

Table S11. Activities of seven selected synAQUs against microorganisms (MIC: µg/mL). Merpenem and vancomycin were used as controls.

Type	Organism	Strain	synAQU2-C3	synAQU3-cFA	synAQU4-L	synAQU5-L	synAQU5-C4	synAQU6-L	synAQU6-C4	Meropenem	Vancomycin
Gram-negative	*Escherichia coli*	DH5α	>64	32	>64	2	8	32	8	<0.125	n.d.
	Acinetobacter baumannii	ATCC 19606	>64	>64	>64	8	32	>64	>64	0.5	n.d.
		ATCC 17978	4	64	>64	4	32	32	32	0.25	n.d.
		P53	8	>64	>64	8	>64	>64	>64	32	n.d.
		1104008	8	>64	>64	4	16	>64	32	16	n.d.
	Klebsiella pneumoniae	ATCC 13883	>64	>64	>64	8	>64	>64	32	<0.125	n.d.
		NCTC 5056	32	>64	>64	8	>64	>64	32	0.125	n.d.
		CRKP 5452	>64	>64	>64	4	32	>64	16	16	n.d.
		CRKP HS11286	>64	>64	>64	4	32	>64	32	8	n.d.
	Pseudomonas aeruginosa	ATCC 9027	>64	32	>64	4	16	32	>64	0.25	n.d.
		PAO1	32	64	>64	4	32	>64	16	16	n.d.
	Salmonella typhimurium	LXX	>64	64	>64	8	32	>64	32	0.125	n.d.
	Salmonella enterica	ATCC 14028	>64	>64	>64	4	32	>64	32	0.125	n.d.
	Shigella Castellani	LXX	>64	64	>64	8	32	>64	>64	0.125	n.d.
	Vibrio alginolyticus	XSBZ14	16	>64	>64	8	32	>64	>64	<0.125	n.d.
Gram-positive	*Bacllus subtilis*	ATCC 23857	4	8	>64	8	2	8	4	n.d.	0.25
	Enterococcus faecalis	ATCC 29212	32	>64	>64	32	4	>64	>64	n.d.	2
		26	8	16	>64	8	2	4	2	n.d.	1
		28	>64	>64	>64	16	4	>64	>64	n.d.	>64
	Enterococcus faecium	35682	8	32	>64	16	4	>64	32	n.d.	>64
	Staphylococcus aureus	Newman	>64	>64	>64	32	4	>64	>64	n.d.	0.5
		USA300	>64	>64	>64	16	4	>64	>64	n.d.	1
		ATCC 25923	16	>64	>64	32	4	>64	16	n.d.	1
		ATCC 6538	16	>64	>64	16	4	32	16	n.d.	1
	Streptococcus pyogenes	ATCC 19615	2	8	4	8	2	2	4	n.d.	0.5
	Streptococcus mutans	ATCC 25175	8	32	16	16	4	4	4	n.d.	2
	Mycobacterium smegmatis	mc^2 155	>64	>64	>64	>64	>64	>64	>64	n.d.	>64
Fungi	*Candida albicans*	ATCC 10231	>64	32	>64	>64	>64	>64	>64	n.d.	n.d.
	Candida albicans	ATCC 64550	>64	32	>64	>64	>64	>64	>64	n.d.	n.d.

Table S12. Summary of enthalpy change (ΔH), entropy change (ΔS) and Gibbs free energy change (ΔG) in isothermal titration

	Kd (μM)	ΔH (kcal/mol)	ΔS (kcal/mol/K)	ΔG (kcal/mol)
Aquicidine L & CL	0.28	-1.03	0.028	-9.38
Aquicidine C4 & CL	0.06	-0.73	0.040	-12.66
Aquicidine L & PG	0.32	-0.96	0.027	-9.01
Aquicidine C4 & PG	0.08	-0.81	0.035	-11.25
Aquicidine L & LPS	0.29	-0.91	0.030	-9.85
Aquicidine C4 & LPS	1.84	-1.93	0.019	-7.59
Aquicidine L & PE	7.88	-4.90	0.004	-6.09
Aquicidine C4 & PE	49.34	-27.30	-0.074	-5.24

a

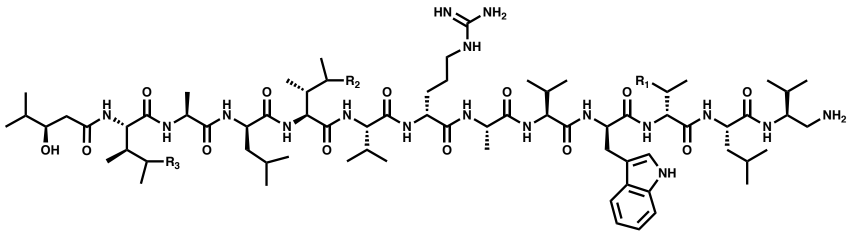

Aquimarin A: $R_1 = H$ $R_2 = Cl$ $R_3 = Cl$
Aquimarin B: $R_1 = CH_3$ $R_2 = Cl$ $R_3 = Cl$
Aquimarin C: $R_1 = H$ $R_2 = H$ $R_3 = H$
Aquimarin D: $R_1 = CH_3$ $R_2 = H$ $R_3 = H$
Aquimarin G: $R_1 = H$ $R_2 = H$ $R_3 = Cl$
Aquimarin H: $R_1 = CH_3$ $R_2 = H$ $R_3 = Cl$
Type: NRPS
Source: ***Aquimarina*** **sp. Ap349**

b

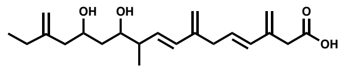

Cuniculene 6A
Type: Trans-AT PKS
Source: ***Aquimarina*** **sp. Ap349**

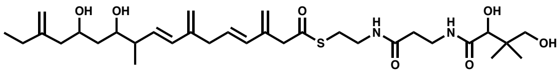

Cuniculene 6B
Type: Trans-AT PKS
Source: ***Aquimarina*** **sp. Ap349**

c

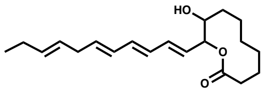

Didemnilactone B
Type: PKS
Source: ***Aquimarina muelleri*** **DSM 19832**

d

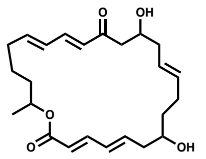

Macrolactin A 15-ketone
Type: PKS
Source: ***Aquimarina muelleri*** **DSM 19832**

Figure S1. Known natural products from the genus *Aquimarina*. The types and sources of natural products were shown.

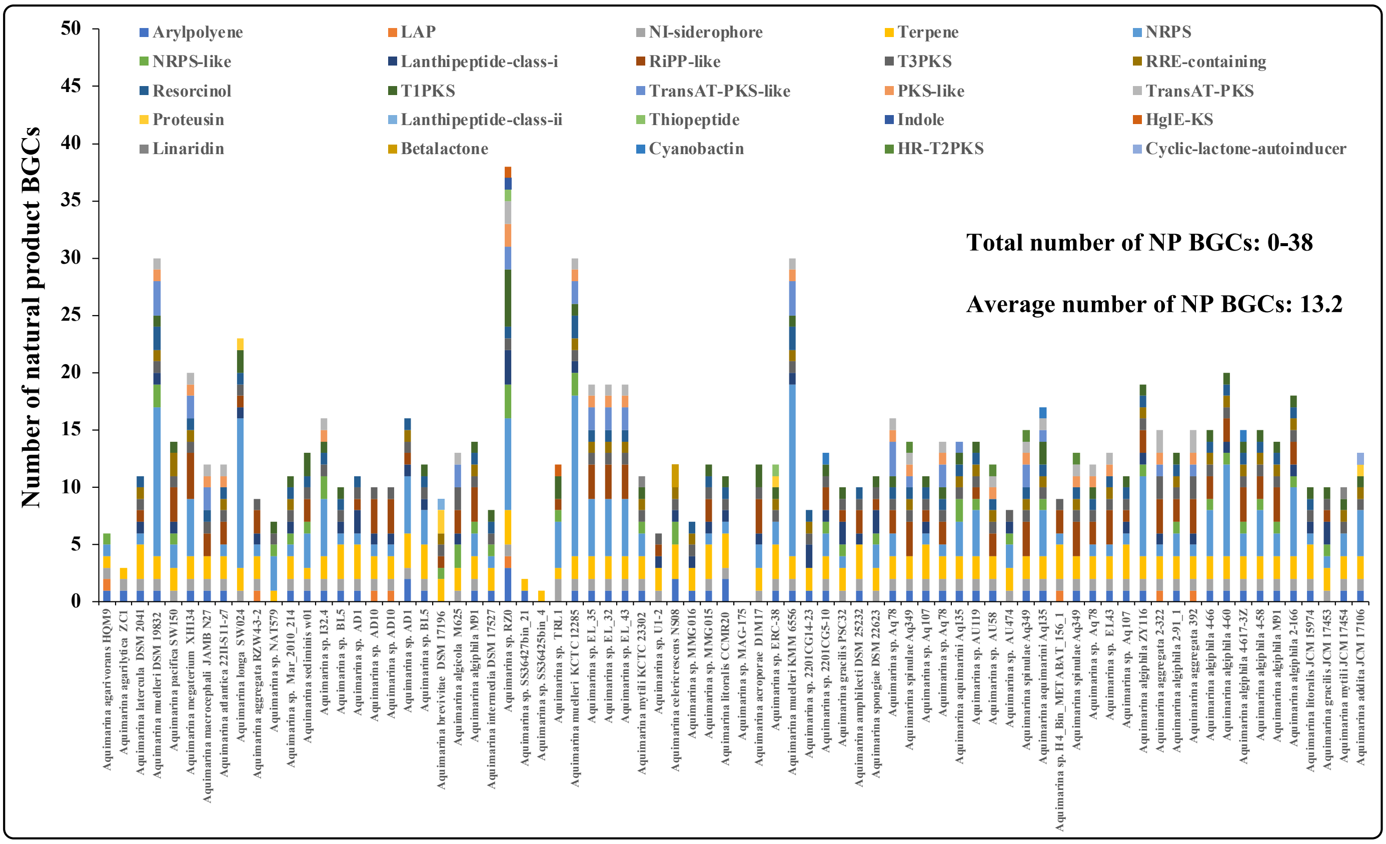

Figure S2. Distribution of natural product BGCs across genomes of 76 *Aquimarina* strains

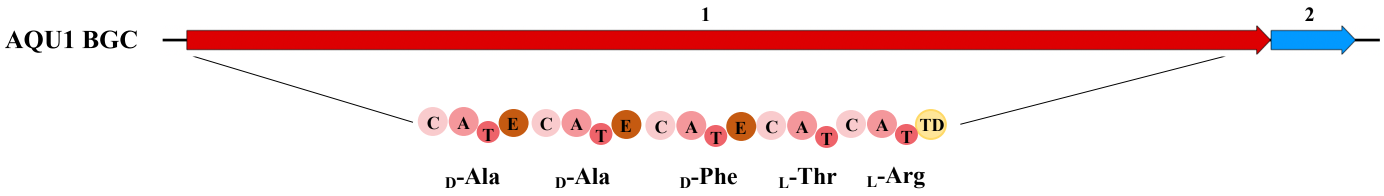

ORF	Gene size (bp)	Gene name	Proposed Function	Protein [Organism], Corresponding to Gene with Sequence Similarity	NCBI Gene Bank Accession Number	E-value	Protein ID%
1	21192	orf1	NRPS	non-ribosomal peptide synthase/polyketide synthase [uncultured Kordia sp.]	WP_298427782.1	0	45.99
2	1656	orf2	transporter	cyclic peptide export ABC transporter [Aquimarina sp. RZ0]	WP_062061820.1	0	67.04

Figure S3. The AQU1 biosynthetic gene cluster analysis. The AQU1 BGC has been deposited in GenBank under Accession numbers PQ867813.

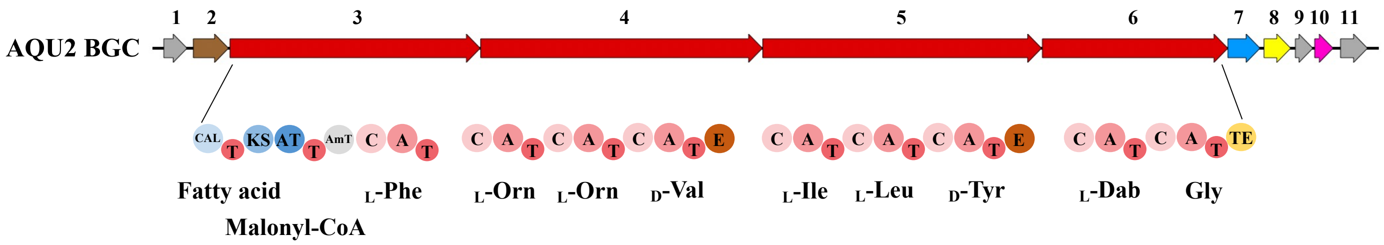

ORF	Gene size (bp)	Gene name	Proposed Function	Protein [Organism], Corresponding to Gene with Sequence Similarity	NCBI Gene Bank Accession Number	E-value	Protein ID%
1	921	orf1	unknown	cupin-like domain-containing protein [Tenacibaculum agarivorans]	WP_075343711.1	2e-153	67.24
2	1389	orf2	aspartate aminotransferase	aspartate aminotransferase family protein [Aquimarina atlantica]	WP_051575676.1	0	80.09
3	9894	orf3	NRPS/PKS	non-ribosomal peptide synthetase/type I polyketide synthase [Flavivirga jejuensis]	WP_303304554.1	0	52.73
4	11139	orf4	NRPS	non-ribosomal peptide synthetase [Aquimarina muelleri]	WP_027413623.1	0	47.44
5	11019	orf5	NRPS	non-ribosomal peptide synthetase [Aquimarina muelleri]	WP_027413623.1	0	42.05
6	7332	orf6	NRPS	non-ribosomal peptide synthetase [Aquimarina sp. Aq78]	WP_159098269.1	0	49.93
7	1272	orf7	transporter	MFS transporter [Aquimarina sp. Aq78]	WP_106795021.1	0	69.50
8	1056	orf8	monooxygenase	MupA/Atu3671 family FMN-dependent luciferase-like monooxygenase [Aquimarina longa]	WP_062052623.1	0	72.54
9	693	orf9	unknown	hypothetical protein [Kordia aestuariivivens]	WP_187563532.1	9e-95	62.67
10	732	orf10	thioesterase	thioesterase domain-containing protein [Tenacibaculum sp. MAR_2009_124]	WP_093672567.1	7e-102	61.60
11	1068	orf11	unknown	hypothetical protein [Aquimarina sp. AD10]	WP_118501237.1	0	70.11

Figure S4. The AQU2 biosynthetic gene cluster analysis. The AQU2 BGC has been deposited in GenBank under Accession numbers PQ867814.

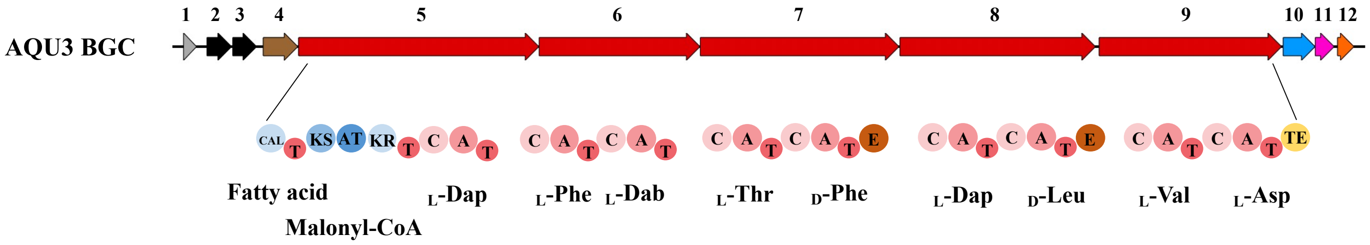

ORF	Gene size (bp)	Gene name	Proposed Function	Protein [Organism], Corresponding to Gene with Sequence Similarity	NCBI Gene Bank Accession Number	E-value	Protein ID%
1	519	orf1	transcription antiterminator	UpxY family transcription antiterminator [Aquimarina]	WP_025665193.1	$6e^{-103}$	88.89
2	990	orf2	Dap biosynthesis	2,3-diaminopropionate biosynthesis protein SbnA [uncultured Kordia sp.]	WP_298510507.1	$1e^{-101}$	50.94
3	960	orf3	Dap biosynthesis	2,3-diaminopropionate biosynthesis protein SbnB [Smithella sp.]	MDD5343017.1	$3e^{-126}$	56.92
4	1389	orf4	aspartate aminotransferase	aspartate aminotransferase family protein [Aquimarina megaterium]	WP_025665192.1	0	94.16
5	9654	orf5	NRPS/PKS	non-ribosomal peptide synthetase/type I polyketide synthase [Aquimarina sp. RZ0]	WP_149627151.1	0	47.94
6	6465	orf6	NRPS	non-ribosomal peptide synthetase [Tenacibaculum sp. E3R01]	WP_255414818.1	0	54.39
7	7986	orf7	NRPS	non-ribosomal peptide synthetase [Flavobacterium sp. Fl-318]	WP_319624926.1	0	44.51
8	7854	orf8	NRPS	non-ribosomal peptide synthetase [Chitinophaga sancti]	WP_072361453.1	0	43.89
9	7329	orf9	NRPS	non-ribosomal peptide synthetase [Aquimarina sp. TRL1]	WP_176027155.1	0	43.40
10	1281	orf10	transporter	MFS transporter [Aquimarina sp. TRL1]	WP_176027154.1	0	69.50
11	762	orf11	thioesterase	thioesterase domain-containing protein [Aquimarina atlantica]	WP_051575675.1	$8e^{-98}$	58.33
12	660	orf12	PPTase	4'-phosphopantetheinyl transferase superfamily protein [Aquimarina spinulae]	WP_108807990.1	$2e^{-142}$	88.13

Figure S5. The AQU3 biosynthetic gene cluster analysis. The AQU3 BGC has been deposited in GenBank under Accession numbers PQ867815.

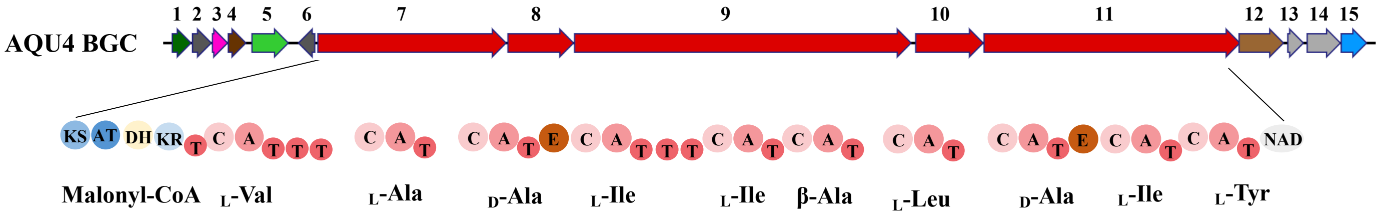

ORF	Gene size (bp)	Gene name	Proposed Function	Protein [Organism], Corresponding to Gene with Sequence Similarity	NCBI Gene Bank Accession Number	E-value	Protein ID%
1	906	orf1	halogenase	chlorinating enzyme [Aquimarina sp. BL5]	WP_118459668.1	0	83.72
2	903	orf2	transposase	IS1595 family transposase [Flavobacteriaceae bacterium]	QMU64132.1	1e^{-164}	72.33
3	729	orf3	thioesterase	thioesterase domain-containing protein [Aquimarina sp. BL5]	WP_118459667.1	1e^{-133}	77.31
4	843	orf4	methyltransferase	class I SAM-dependent methyltransferase [Aquimarina sp. BL5]	WP_118459666.1	0	86.38
5	1749	orf5	acyltransferase	acyltransferase domain-containing protein [Aquimarina sp. BL5]	WP_118459665.1	0	61.51
6	786	orf6	transposase	IS4 family transposase [Arenibacter sp. M-2]	WP_286107405.1	2e^{-150}	78.71
7	9066	orf7	NRPS/PKS	non-ribosomal peptide synthetase/type I polyketide synthase [Aquimarina sp. BL5]	WP_118459664.1	0	65.28
8	3162	orf8	NRPS	non-ribosomal peptide synthetase [Aquimarina sp. BL5]	WP_118459663.1	0	61.78
9	16212	orf9	NRPS	non-ribosomal peptide synthetase [Aquimarina sp. BL5]	WP_118459662.1	0	66.29
10	3252	orf10	NRPS	non-ribosomal peptide synthetase [Aquimarina sp. BL5]	WP_118459661.1	0	64.11
11	12327	orf11	NRPS	non-ribosomal peptide synthetase [Aquimarina sp. BL5]	WP_118459660.1	0	66.62
12	2139	orf12	aminotransferase	aminotransferase class III-fold pyridoxal phosphate-dependent enzyme [Aquimarina sp. BL5]	WP_118459659.1	0	72.46
13	753	orf13	unknown	outer membrane lipoprotein-sorting protein [Aquimarina sp. BL5]	WP_118459657.1	0	80.80
14	1599	orf14	unknown	MAD(P)-binding domain-containing protein [Aquimarina sp. BL5]	WP_118459656.1	0	78.34
15	1224	orf15	transporter	ABC transporter permease [Aquimarina sp. BL5]	RKM93420.1	0	78.38

Figure S6. The AQU4 biosynthetic gene cluster analysis. The AQU4 BGC has been deposited in GenBank under Accession numbers PQ867816.

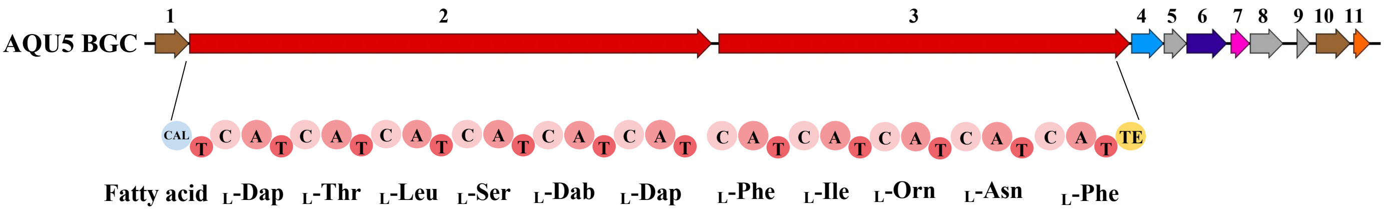

ORF	Gene size (bp)	Gene name	Proposed Function	Protein [Organism], Corresponding to Gene with Sequence Similarity	NCBI Gene Bank Accession Number	E-value	Protein ID%
1	1374	orf1	aspartate aminotransferase	aspartate aminotransferase family protein [Aquimarina atlantica]	WP_051575676.1	0	78.21
2	21420	orf2	NRPS/PKS	non-ribosomal peptide synthase/polyketide synthase [Aquimarina sp. EL_43]	WP_254514244.1	0	88.96
3	16860	orf3	NRPS	non-ribosomal peptide synthetase [Aquimarina sp. EL_43]	WP_254514245.1	0	90.82
4	1299	orf4	transporter	MFS transporter [Aquimarina megaterium]	WP_035095867.1	0	95.14
5	915	orf5	unknown	cupin-like domain-containing protein [Aquimarina longa]	WP_062055771.1	$1e^{-171}$	75.84
6	1629	orf6	MBL fold metallo-hydrolase	MBL fold metallo-hydrolase [Flavobacterium collinsii]	WP_173968376.1	0	81.27
7	759	orf7	thioesterase	thioesterase domain-containing protein [Aquimarina megaterium]	WP_025665195.1	$2e^{-174}$	93.65
8	1338	orf8	aspartate kinase	aspartate kinase [unclassified Aquimarina]	WP_196910732.1	0	87.19
9	522	orf9	transcription antiterminator	UpxY family transcription antiterminator [Aquimarina sp. Aq78]	WP_106793804.1	$2e^{-101}$	87.72
10	1389	orf10	aspartate aminotransferase	aspartate aminotransferase family protein [Aquimarina sp. Aq78]	WP_106793801.1	0	94.16
11	657	orf11	PPTase	4'-phosphopantetheinyl transferase superfamily protein [Aquimarina atlantica]	WP_034246182.1	$6e^{-151}$	95.43

Figure S7. The AQU5 biosynthetic gene cluster analysis. The AQU5 BGC has been deposited in GenBank under Accession numbers PQ867817.

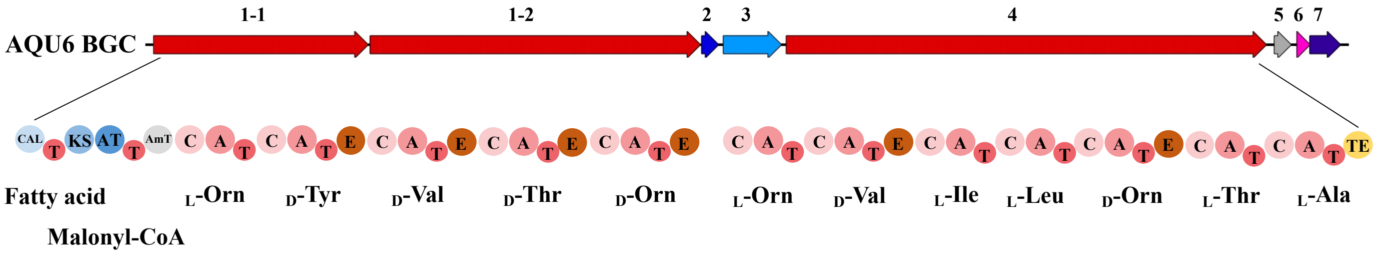

ORF	Gene size (bp)	Gene name	Proposed Function	Protein [Organism], Corresponding to Gene with Sequence Similarity	NCBI Gene Bank Accession Number	E-value	Protein ID%
1	11442	orf1-1	NRPS/PKS	hybrid non-ribosomal peptide synthetase/type I polyketide synthase [Flavobacterium sp. ov086]	WP_089350527.1	0	53.13
1	17595	orf1-2	NRPS	non-ribosomal peptide synthetase [Brevibacillus laterosporus]	WP_113755527.1	0	39.44
2	915	orf2	amidinotransferase	amidinotransferase [uncultured Aquimarina sp.]	WP_299608317.1	$8e^{-178}$	77.63
3	3108	orf3	transporter	cyclic peptide export ABC transporter [Candidatus Aminicenantes bacterium]	NIM13229.1	0	38.81
4	25575	orf4	NRPS	non-ribosomal peptide synthetase [Paenibacillus sp. IHBB 10380]	WP_044876767.1	0	39.40
5	915	orf5	unknown	cupin-like domain-containing protein [Aureisphaera galaxeae]	WP_272862966.1	$3e^{-159}$	69.74
6	711	orf6	thioesterase	thioesterase domain-containing protein [uncultured Kordia sp.]	WP_298510486.1	$4e^{-82}$	51.10
7	1653	orf7	MBL fold metallo-hydrolase	MBL fold metallo-hydrolase [Fulvivirga imtechensis]	WP_040496626.1	0	70.09

Figure S8. The AQU6 biosynthetic gene cluster analysis. The AQU6 BGC has been deposited in GenBank under Accession numbers PQ867818.

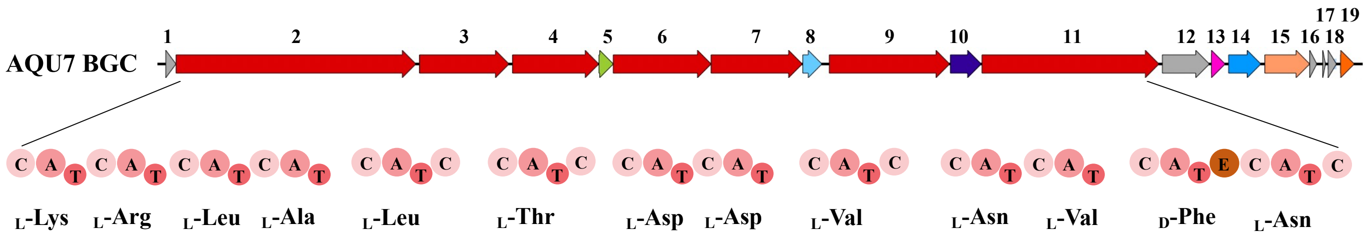

ORF	Gene size (bp)	Gene name	Proposed Function	Protein [Organism], Corresponding to Gene with Sequence Similarity	NCBI Gene Bank Accession Number	E-value	Protein ID%
1	528	orf1	transcription antiterminator	UpxY family transcription antiterminator [uncultured Aquimarina sp.]	WP_299258197.1	$2e^{-113}$	90.29
2	12438	orf2	NRPS	non-ribosomal peptide synthetase [uncultured Aquimarina sp.]	WP_299315822.1	0	70.18
3	4644	orf3	NRPS	non-ribosomal peptide synthetase [uncultured Aquimarina sp.]	WP_299258193.1	0	72.86
4	4473	orf4	NRPS	non-ribosomal peptide synthetase [Flavobacterium chilense]	WP_068843422.1	0	53.83
5	747	orf5	amino acid racemase	amino acid racemase [uncultured Aquimarina sp.]	WP_299258188.1	$7e^{-173}$	94.76
6	5079	orf6	NRPS	non-ribosomal peptide synthetase [uncultured Aquimarina sp.]	WP_299258185.1	0	90.84
7	4728	orf7	NRPS	non-ribosomal peptide synthetase [uncultured Aquimarina sp.]	WP_299258183.1	0	91.68
8	993	orf8	dioxygenase	TauD/TfdA family dioxygenase [uncultured Dokdonia sp.]	WP_299210495.1	0	67.58
9	6243	orf9	NRPS	non-ribosomal peptide synthetase [uncultured Aquimarina sp.]	WP_299258181.1	0	93.80
10	1605	orf10	MBL fold metallo-hydrolase	MBL fold metallo-hydrolase [uncultured Aquimarina sp.]	WP_299315163.1	0	83.15
11	9177	orf11	NRPS	non-ribosomal peptide synthetase [uncultured Aquimarina sp.]	WP_299258177.1	0	90.50
12	2385	orf12	TonB-dependent receptor	TonB-dependent receptor [uncultured Aquimarina sp.]	WP_299315160.1	0	63.68
13	717	orf13	thioesterase	thioesterase domain-containing protein [Aquimarina algiphila]	WP_282081311.1	$9e^{-100}$	60.25
14	1665	orf14	transporter	cyclic peptide export ABC transporter [uncultured Aquimarina sp.]	WP_299258171.1	0	94.77
15	2358	orf15	penicillin acylase	penicillin acylase family protein [uncultured Dokdonia sp.]	WP_299210481.1	0	63.06
16	366	orf16	unknown	hypothetical protein [uncultured Aquimarina sp.]	WP_299258167.1	$1e^{-70}$	94.21
17	144	orf17	unknown	hypothetical protein [uncultured Aquimarina sp.]	WP_299258166.1	$5e^{-17}$	90.70
18	432	orf18	unknown	hypothetical protein [uncultured Aquimarina sp.]	WP_299315148.1	$1e^{-49}$	62.24
19	717	orf19	PPTase	4'-phosphopantetheinyl transferase superfamily protein [uncultured Aquimarina sp.]	WP_299258164.1	$1e^{-152}$	86.13

Figure S9. The AQU7 biosynthetic gene cluster analysis. The AQU7 BGC has been deposited in GenBank under Accession numbers PQ867819.

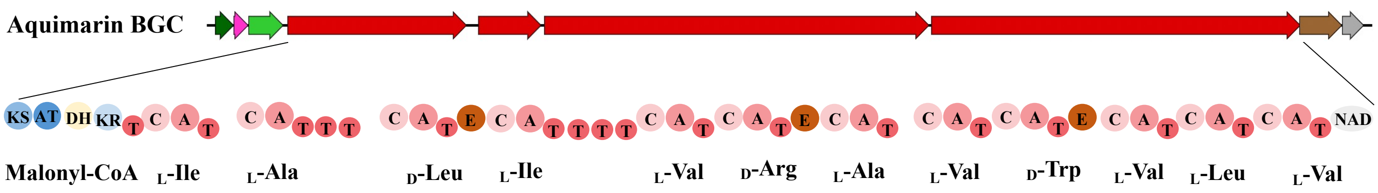

ORF	Gene size (bp)	Gene name	Proposed Function	Protein [Organism], Corresponding to Gene with Sequence Similarity	NCBI Gene Bank Accession Number	E-value	Protein ID%
1	906	orf1	halogenase	chlorinating enzyme [Aquimarina sp. ERC-38]	WP_265769587.1	0	79.12
2	711	orf2	thioesterase	thioesterase domain-containing protein [Aquimarina sp. BL5]	WP_118459667.1	$1e^{-101}$	57.94
3	1731	orf3	acyltransferase	acyltransferase domain-containing protein [Aquimarina sp. ERC-38]	WP_265769590.1	$2e^{-173}$	47.47
4	9003	orf4	NRPS/PKS	non-ribosomal peptide synthetase/type I polyketide synthase [Aquimarina sp. ERC-38]	WP_265769593.1	0	44.26
5	3144	orf5	NRPS	non-ribosomal peptide synthetase [Aquimarina sp. BL5]	WP_118459663.1	0	46.80
6	19380	orf6	NRPS	non-ribosomal peptide synthetase [Aquimarina sp. BL5]	WP_118459662.1	0	42.94
7	18579	orf7	NRPS	non-ribosomal peptide synthase/polyketide synthase [Chitinophaga varians]	WP_188106617.1	0	39.05
8	2127	orf8	aminotransferase	aminotransferase class III-fold pyridoxal phosphate-dependent enzyme [Aquimarina sp. ERC-38]	WP_265769598.1	0	55.54
9	1068	orf9	unknown	hypothetical protein [Telluribacter sp. SYSU D00476]	WP_247232842.1	0	39.70

Figure S10. The aquimarin biosynthetic gene cluster analysis

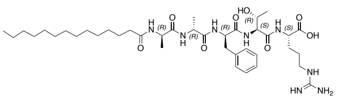

synAQU1-L
inspired by the AQU1 BGC
from *Aquimarina sp.* TRL1

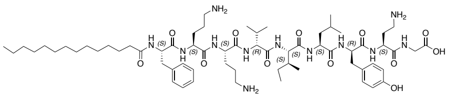

synAQU2-L
inspired by the AQU2 BGC
from *Aquimarina sp.* TRL1

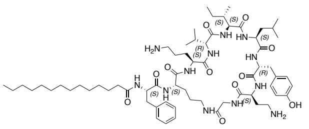

synAQU2-C2
inspired by the AQU2 BGC
from *Aquimarina sp.* TRL1

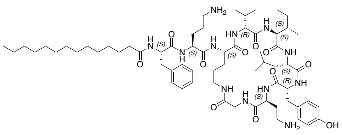

synAQU2-C3
inspired by the AQU2 BGC
from *Aquimarina sp.* TRL1

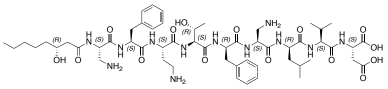

synAQU3-L
inspired by the AQU3 BGC
from *Aquimarina sp.* Aq78

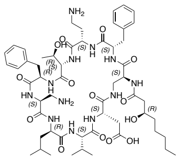

synAQU3-C1
inspired by the AQU3 BGC
from *Aquimarina sp.* Aq78

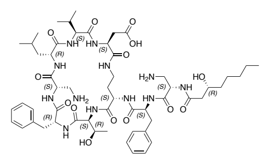

synAQU3-C3
inspired by the AQU3 BGC
from *Aquimarina sp.* Aq78

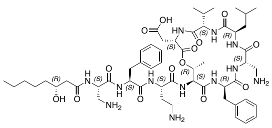

synAQU3-C4
inspired by the AQU3 BGC
from *Aquimarina sp.* Aq78

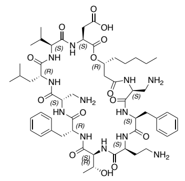

synAQU3-cFA
inspired by the AQU3 BGC
from *Aquimarina sp.* Aq78

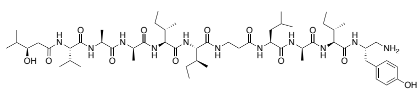

synAQU4-L
inspired by the AQU4 BGC
from *Aquimarina sp.* ERC-38

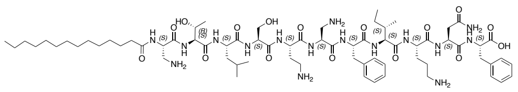

synAQU5-L (Aquicidine L)
inspired by the AQU5 BGC
from *Aquimarina atlantica* 22II-S11-z7

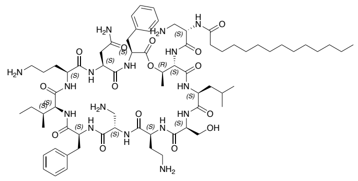

synAQU5-C2
inspired by the AQU5 BGC
from *Aquimarina atlantica* 22II-S11-z7

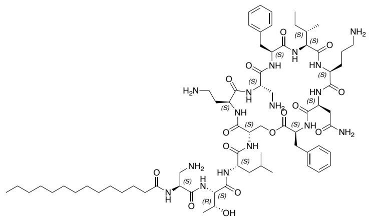

synAQU5-C4 (Aquicidine C4)
inspired by the AQU5 BGC
from *Aquimarina atlantica* 22II-S11-z7

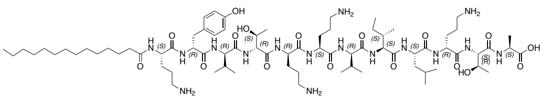

synAQU6-L
inspired by the AQU6 BGC
from *Aquimarina muelleri* KCTC 12285

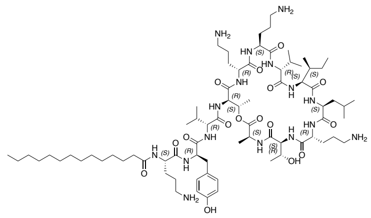

synAQU6-C4
inspired by the AQU6 BGC
from *Aquimarina muelleri* KCTC 12285

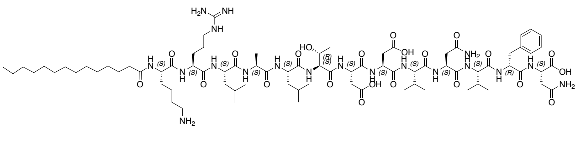

synAQU7-L
inspired by the AQU7 BGC
from *Aquimarina latercula* DSM 2041

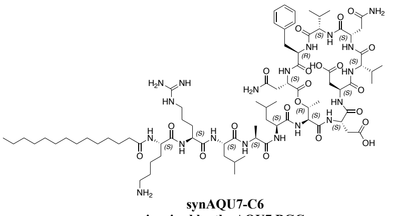

synAQU7-C6
inspired by the AQU7 BGC
from *Aquimarina latercula* DSM 2041

Figure S11. Predicted structures and sequence resources of all compounds synthesized in this study

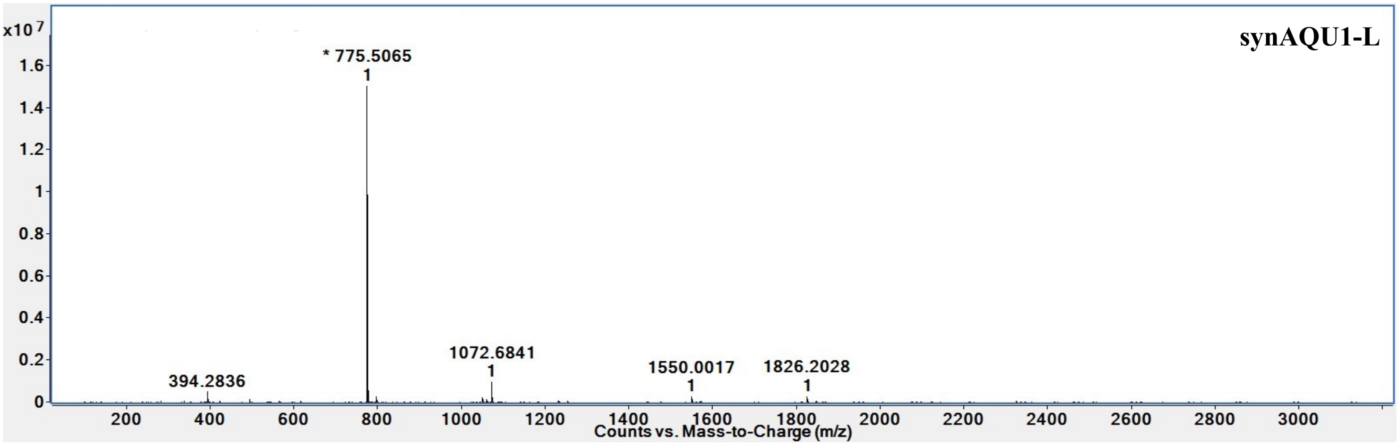

Figure S12. HRMS spectrum of synAQU1-L

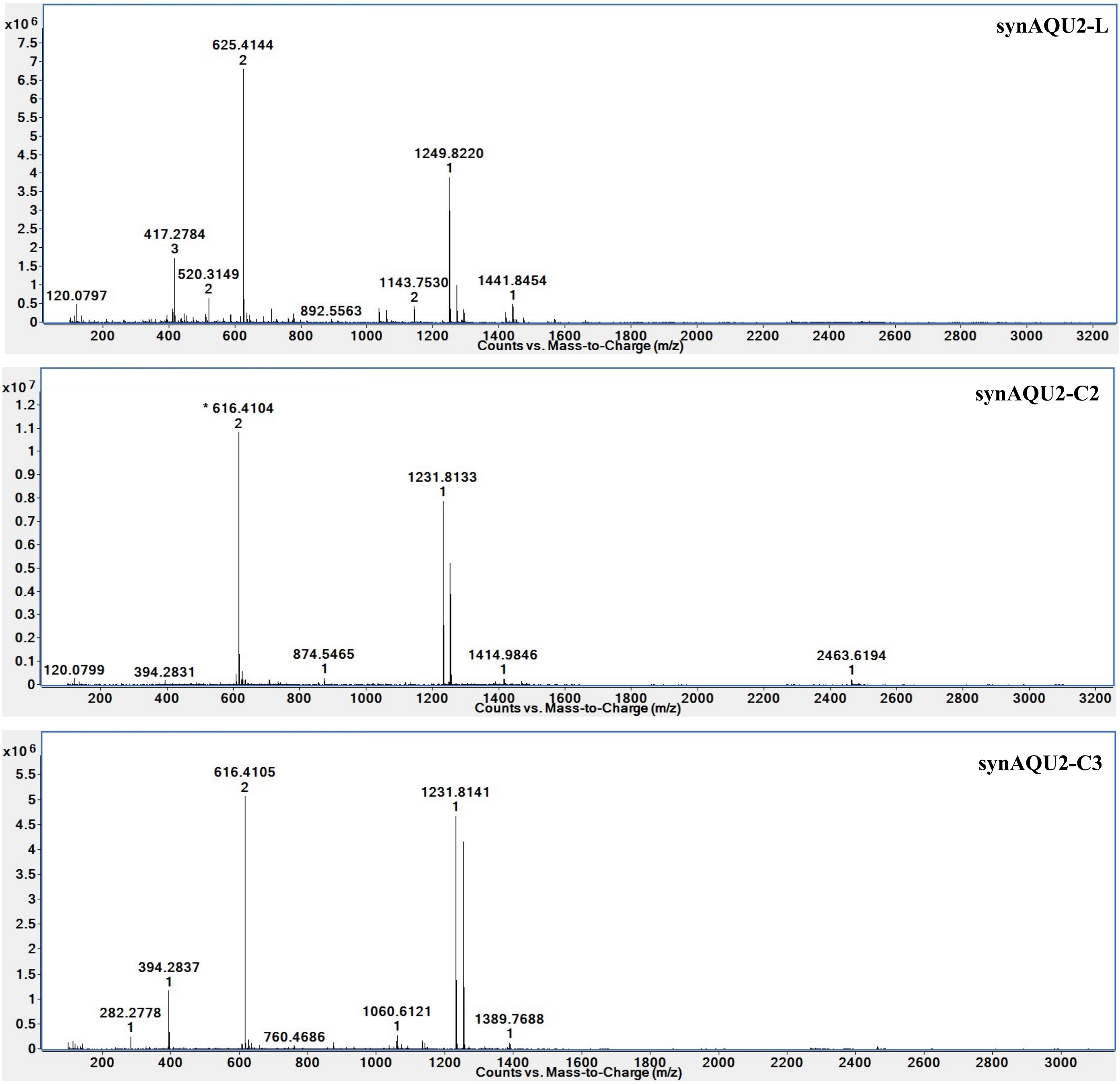

Figure S13. HRMS spectra of synAQU2-L, synAQU2-C2 and synAQU2-C3

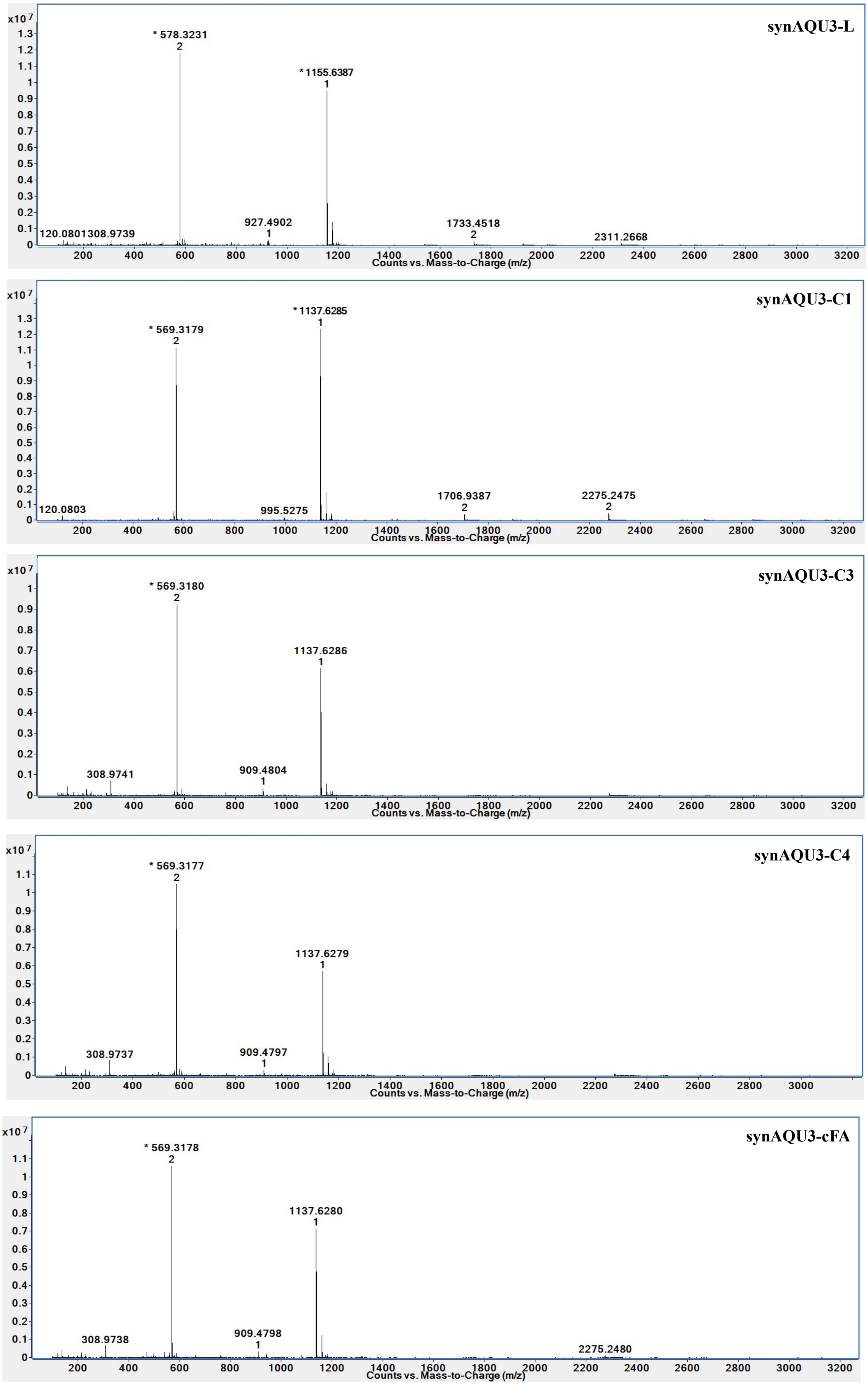

Figure S14. HRMS spectra of synAQU3-L, synAQU3-C1, synAQU3-C3, synAQU3-C4 and synAQU3-cFA

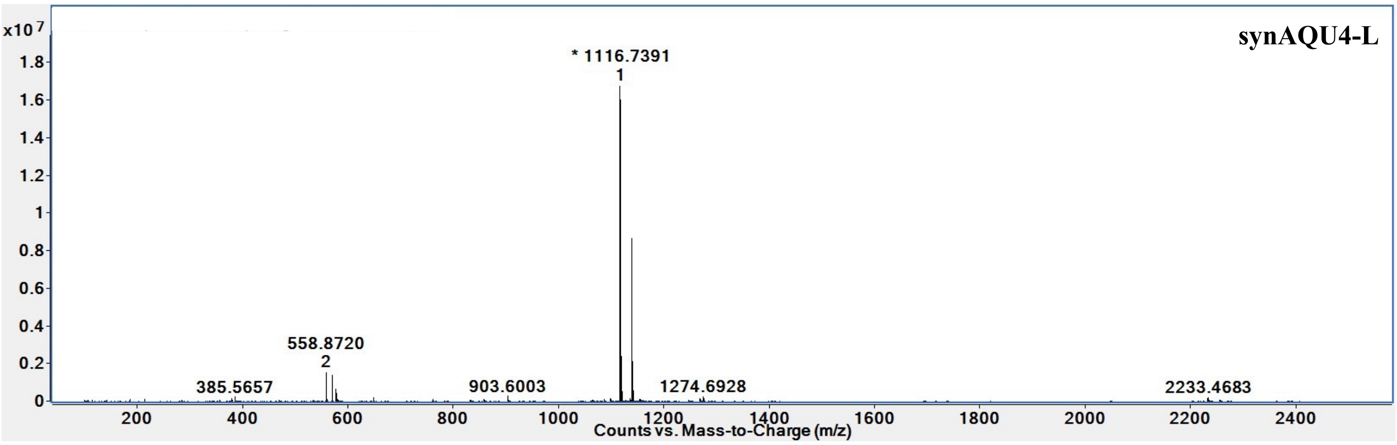

Figure S15. HRMS spectrum of synAQU4-L

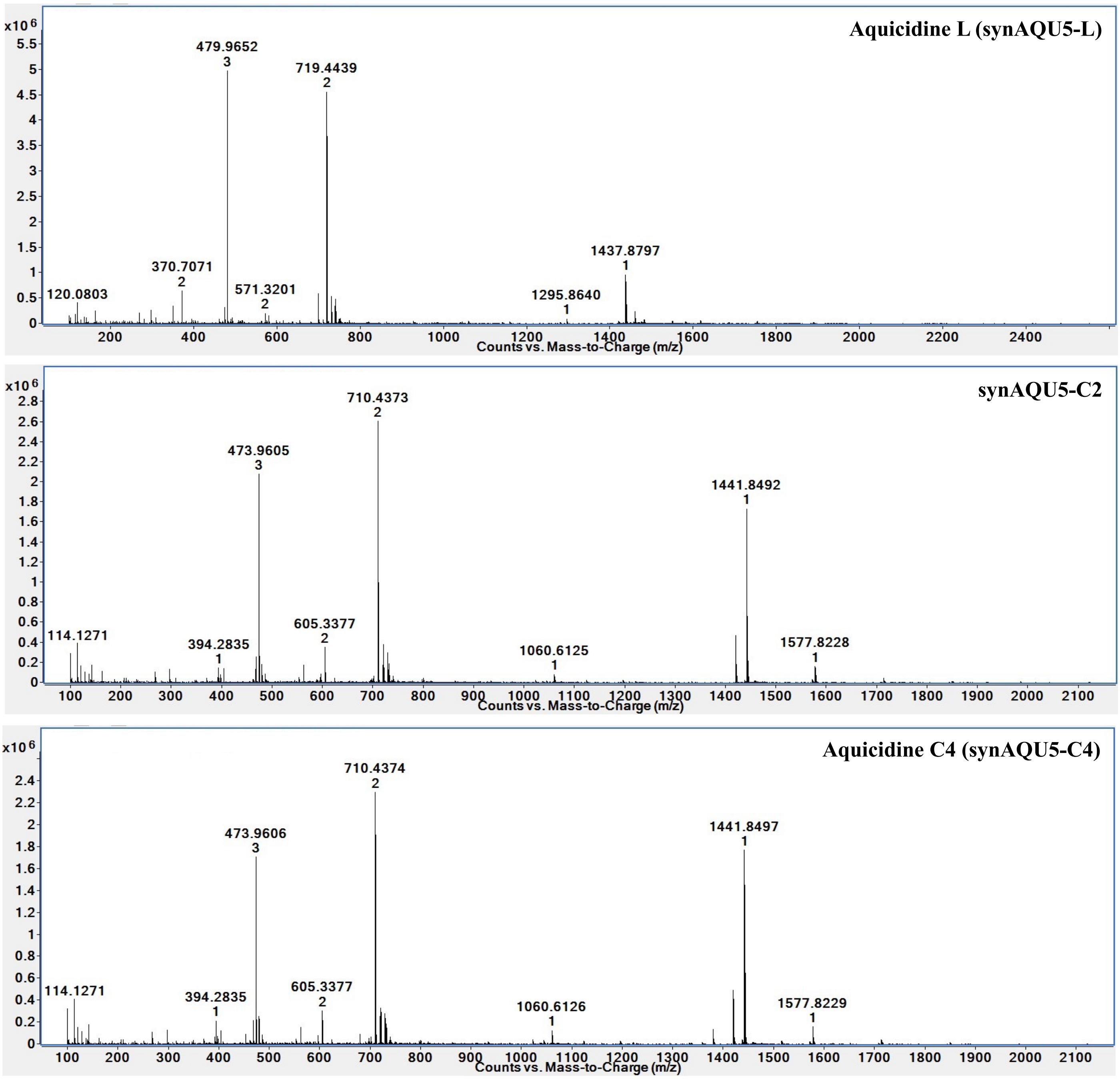

Figure S16. HRMS spectra of aquicidine L (synAQU5-L), synAQU5-C2 and aquicidine C4 (synAQU5-C4)

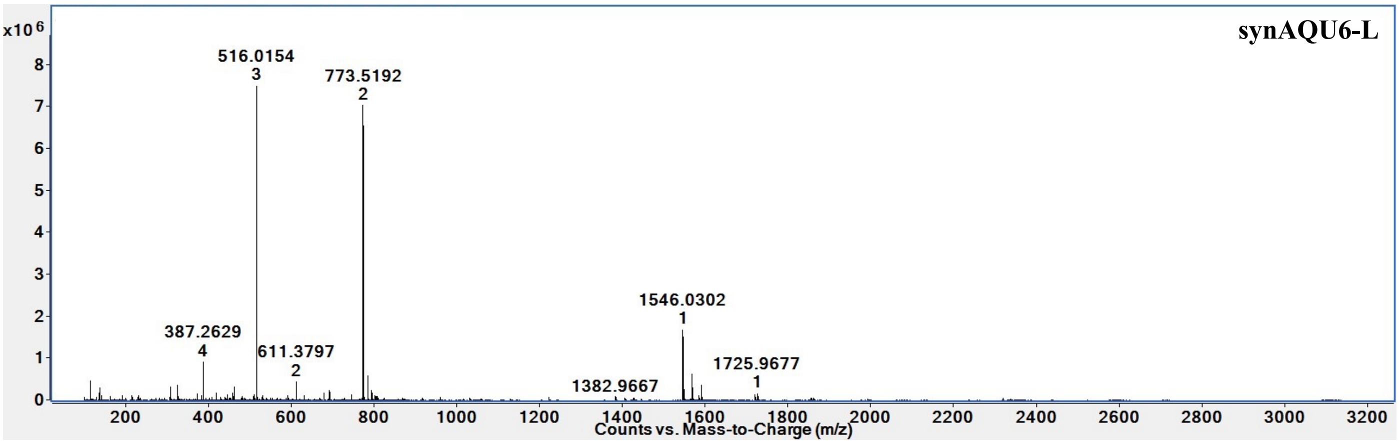

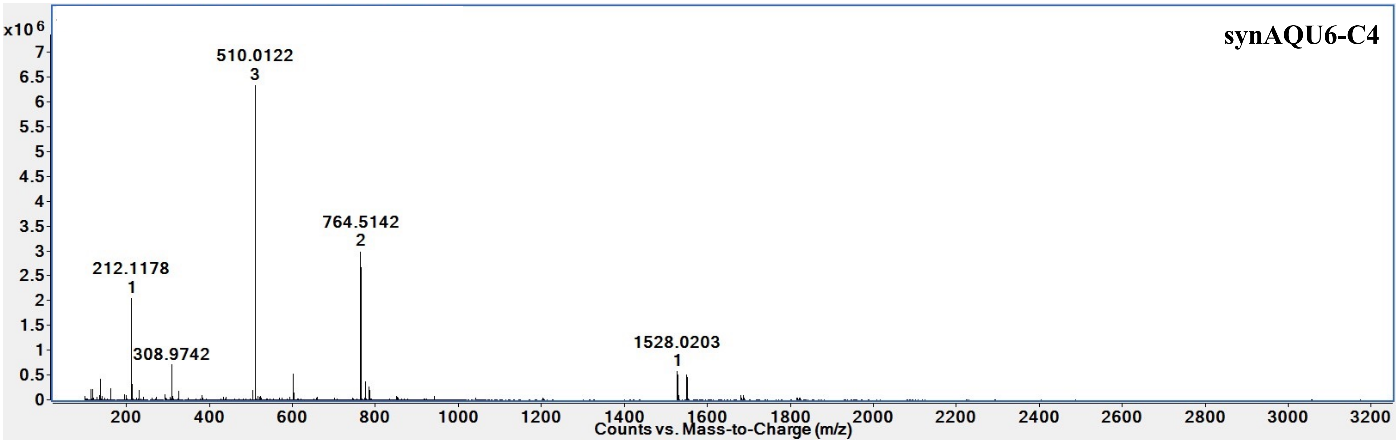

Figure S17. HRMS spectra of synAQU6-L and synAQU6-C4

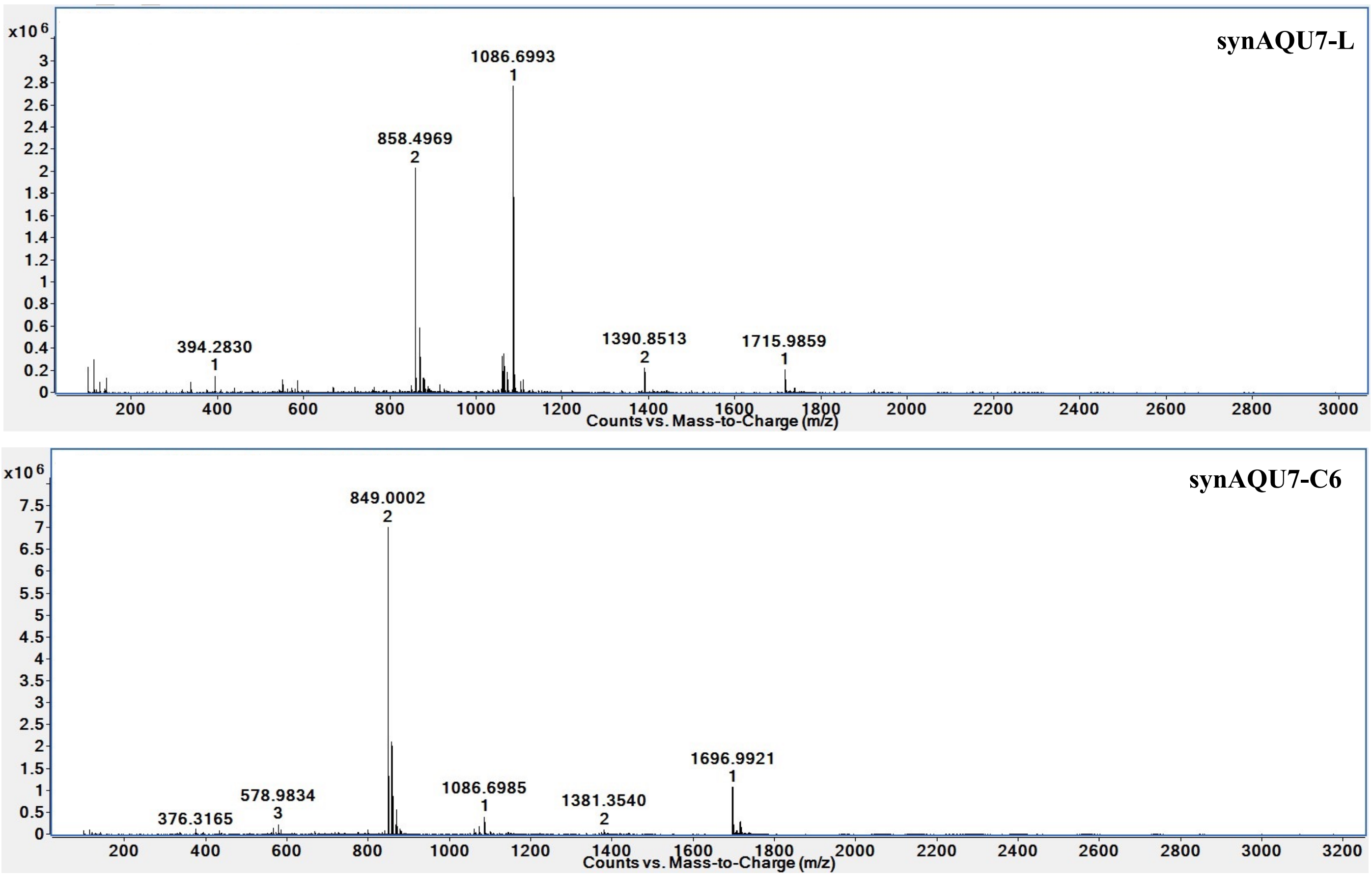

Figure S18. HRMS spectra of synAQU7-L and synAQU7-C6

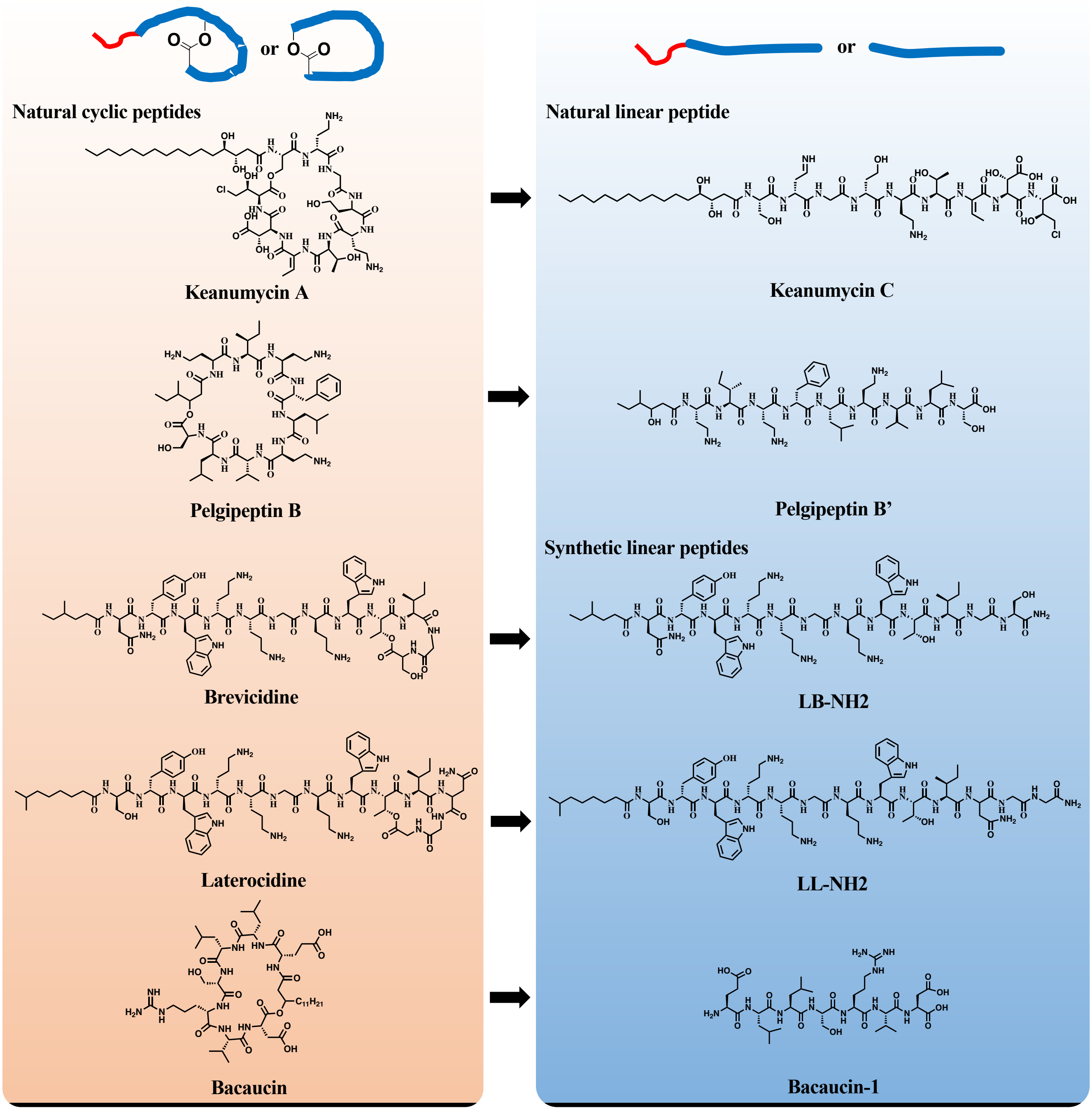

Figure S19. Structures of known paired ring-opening or -closing non-ribosomal peptides

Natural Cyclic Peptides	Natural or Synthetic Linear Peptides
**Keanumycin A**	**Keanumycin C**
**Potent anti-fungal activity**	**Not reported**
**Pelgipeptin B**	**Pelgipeptin B'**
**Broad anti-Gram negative and positive activity**	**No activity against Gram negative and positive bacteria**
**Brevicidine**	**LB-NH2**
**Potent anti-Gram negative activity (MIC = 2-4 µg/mL)**	**Moderate anti-Gram negative activity (MIC = 16 µg/mL)**
**Laterocidine**	**LL-NH2**
**Potent anti-Gram negative activity (MIC = 2-4 µg/mL)**	**Good anti-Gram negative activity (MIC = 4-8 µg/mL)**
**Bacaucin**	**Bacaucin-1**
**1) Broad antibacterial activity<br>2) Hemolytic<br>3) Cytotoxic**	**1) Specific anti-bacterial activity against MRSA<br>2) No hemolytic activity<br>3) No cytotoxic activity**

Figure S20. Bioactivities of known paired ring-opening or -closing non-ribosomal peptides

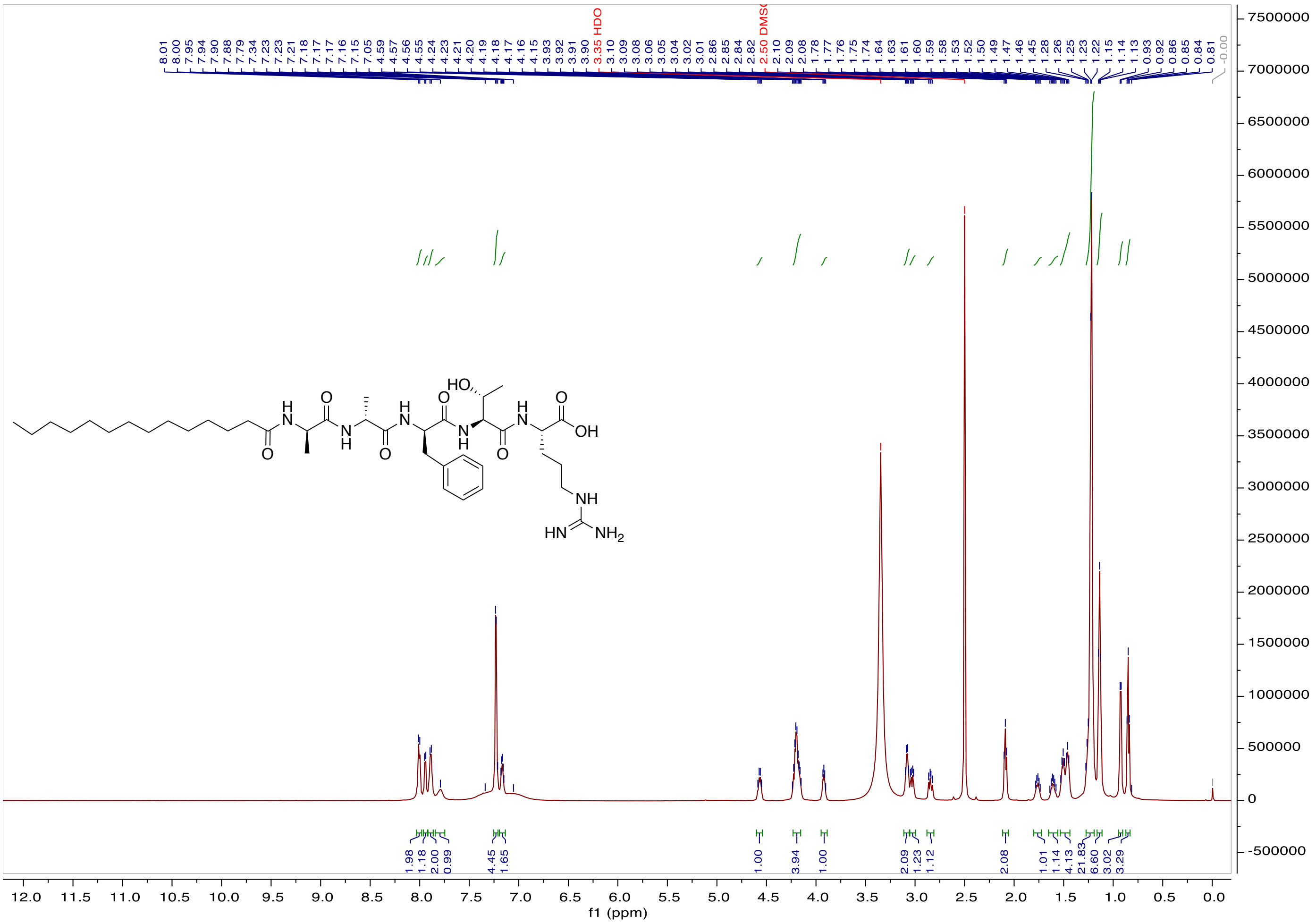

Figure S21. ^{1}H NMR spectrum of synAQU1-L in DMSO-d_6 (600 MHz)

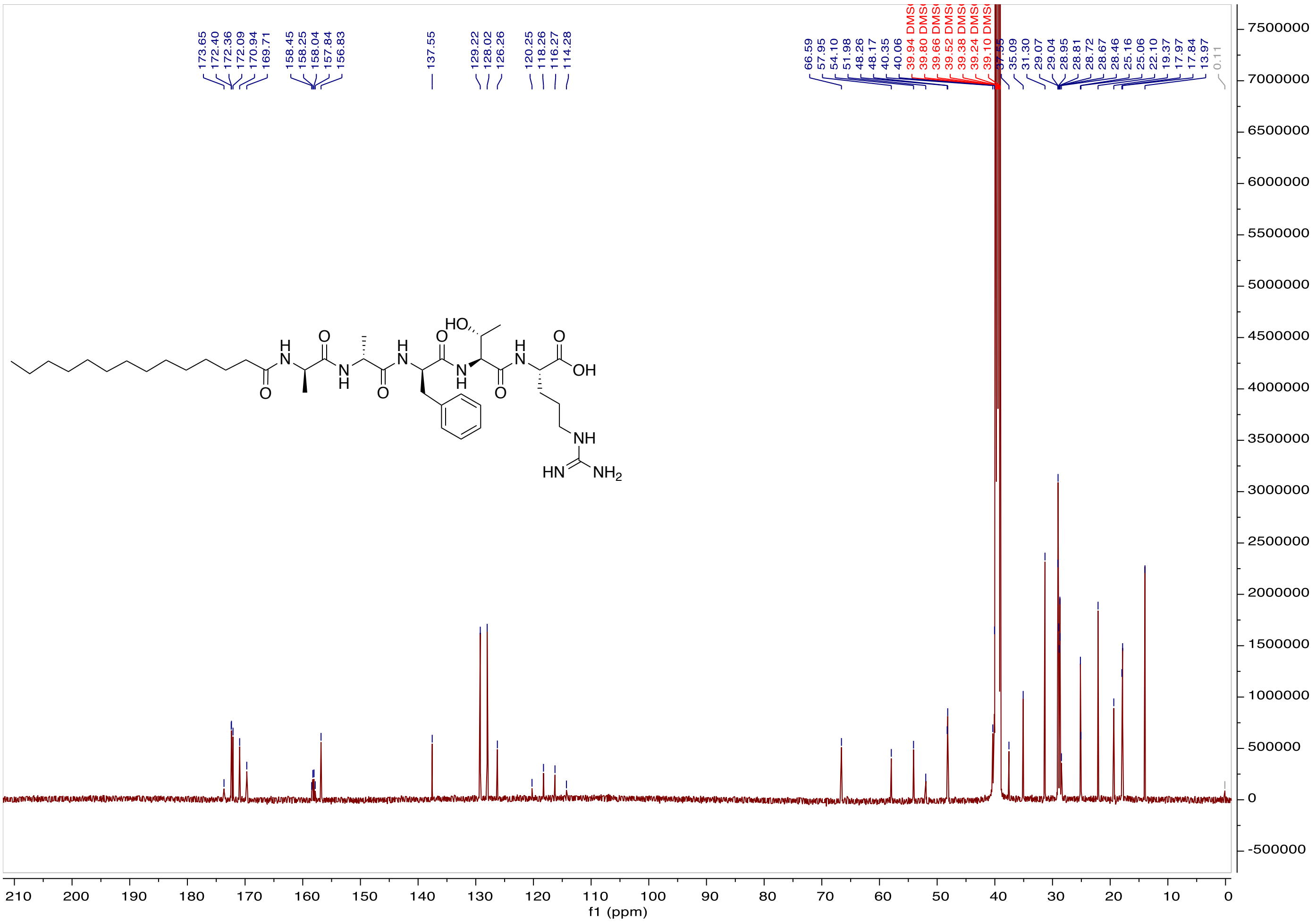

Figure S22. ^{13}C NMR spectrum of synAQU1-L in DMSO-d_6 (150 MHz)

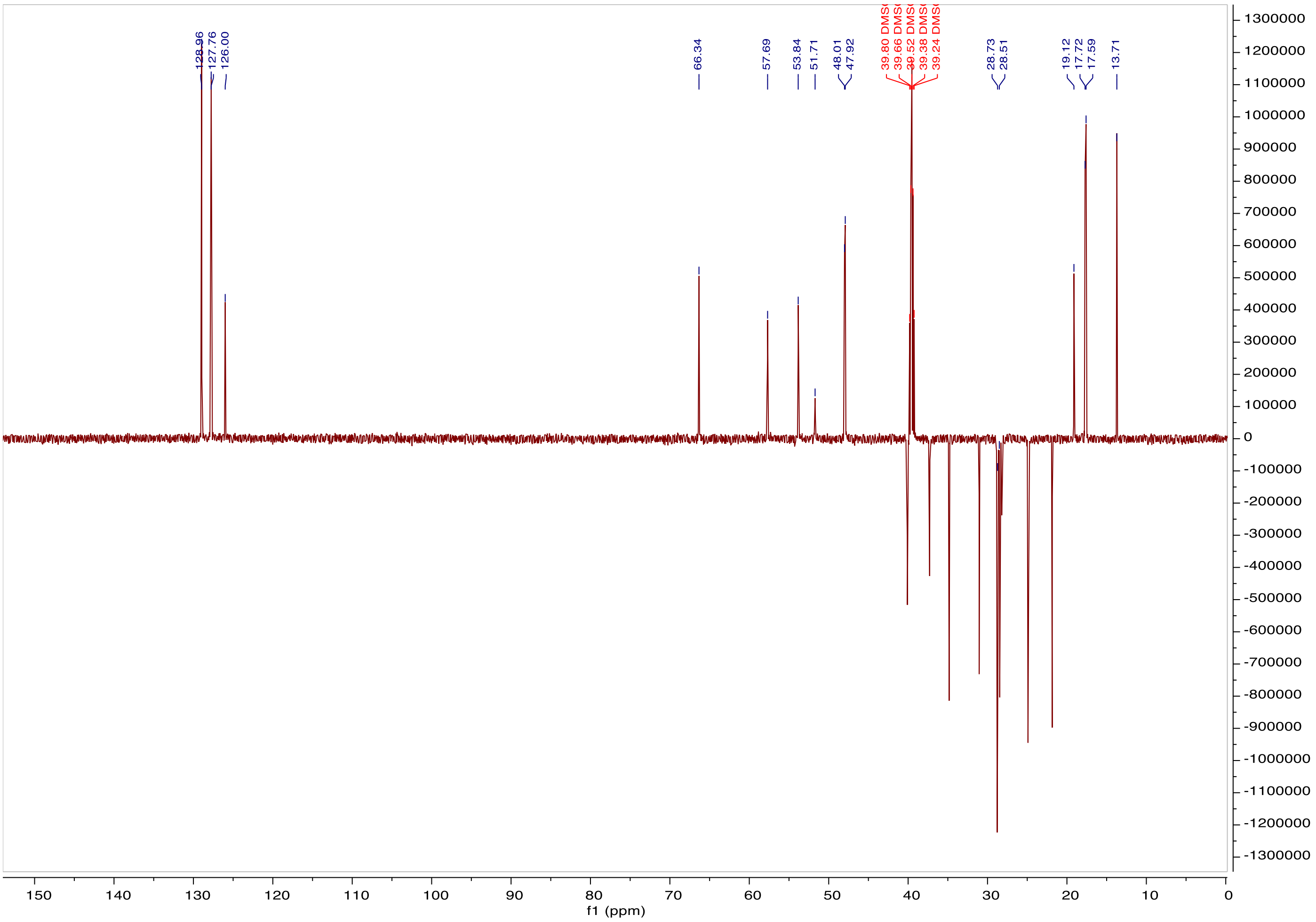

Figure S23. DEPT135 NMR spectrum of synAQU1-L in DMSO-d_6 (150 MHz)

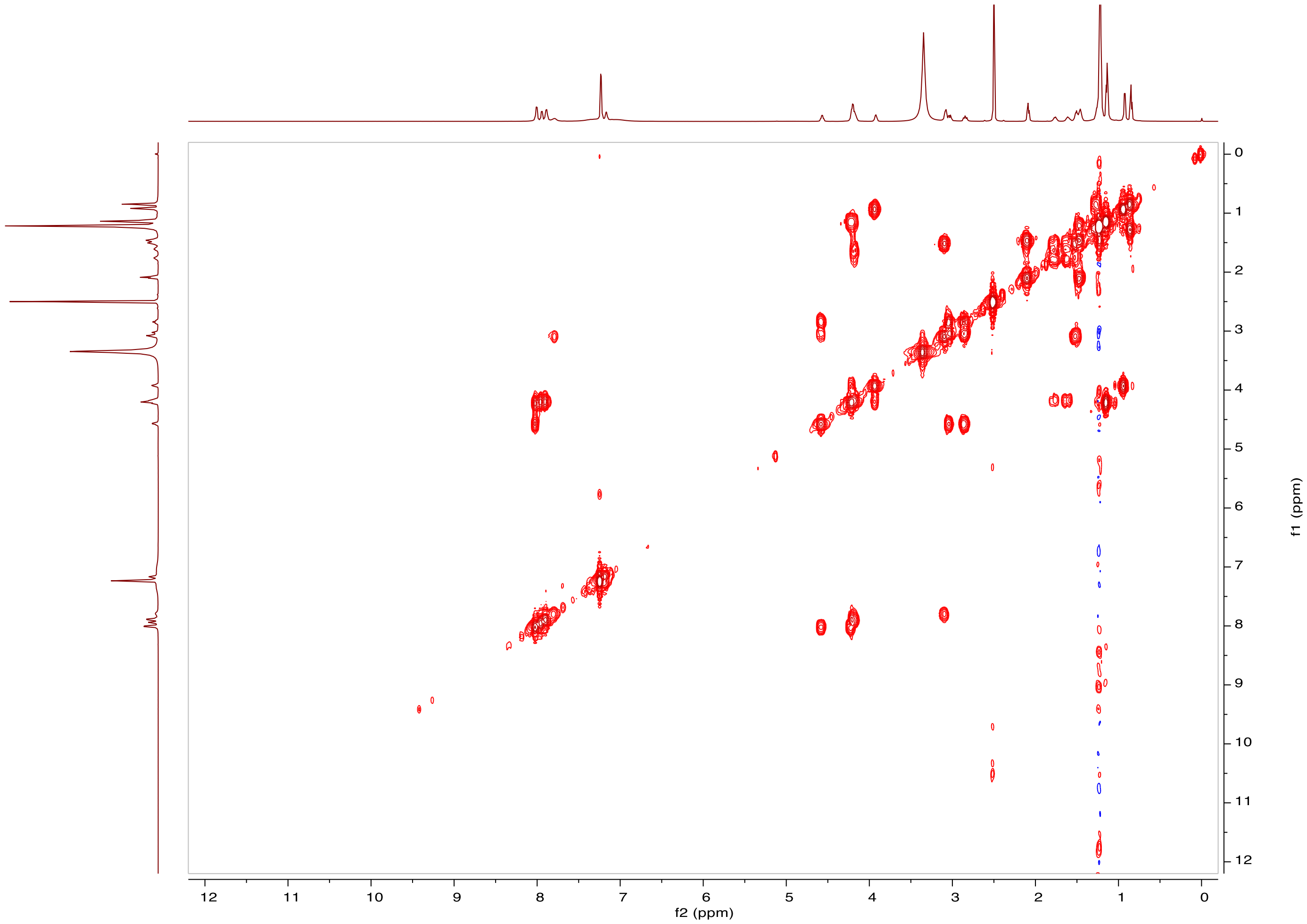

Figure S24. ^{1}H-^{1}H COSY NMR spectrum of synAQU1-L in DMSO-d_6 (600 MHz)

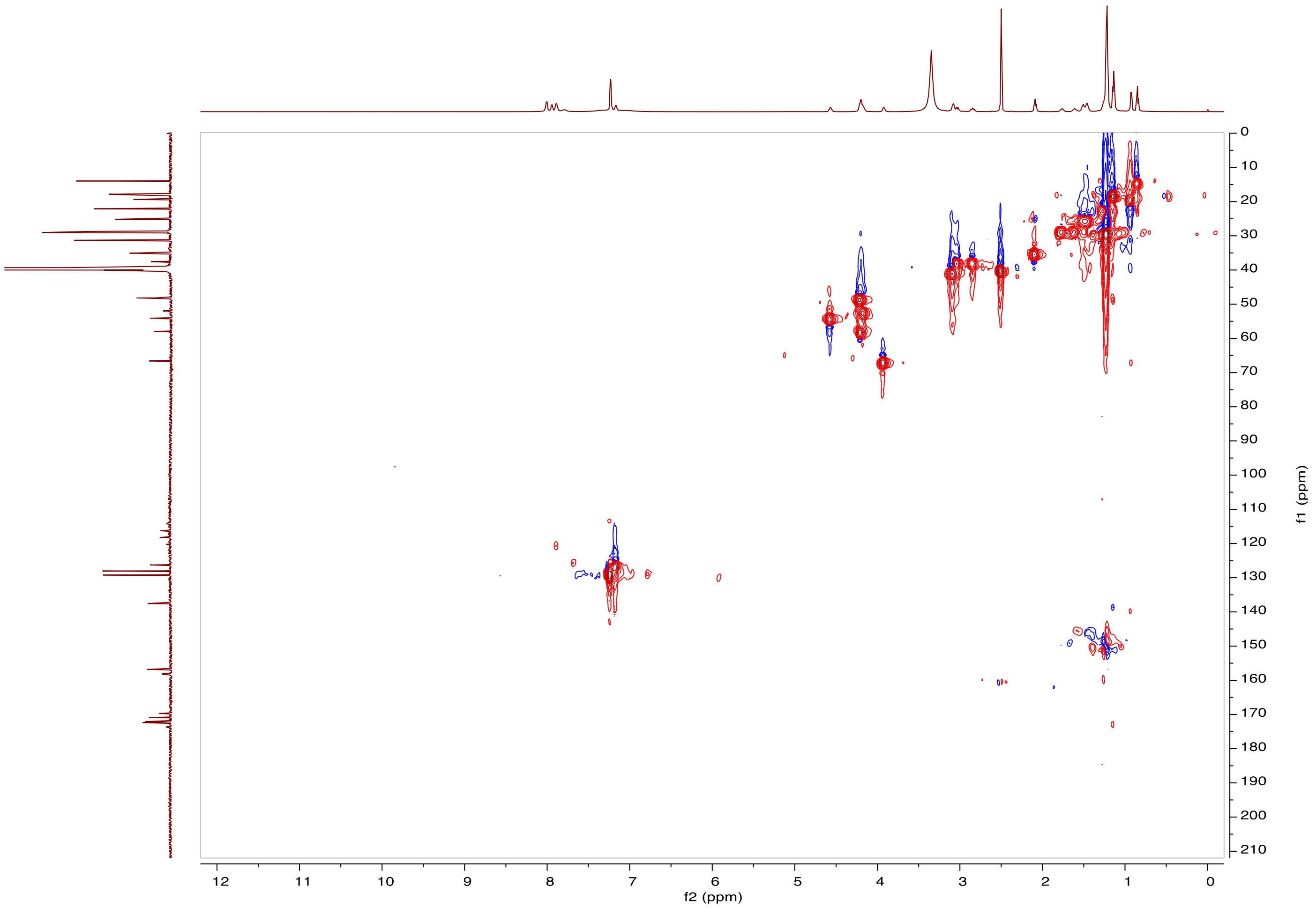

Figure S25. ^{1}H-^{13}C HSQC NMR spectrum of synAQU1-L in DMSO-d_6 (600 MHz)

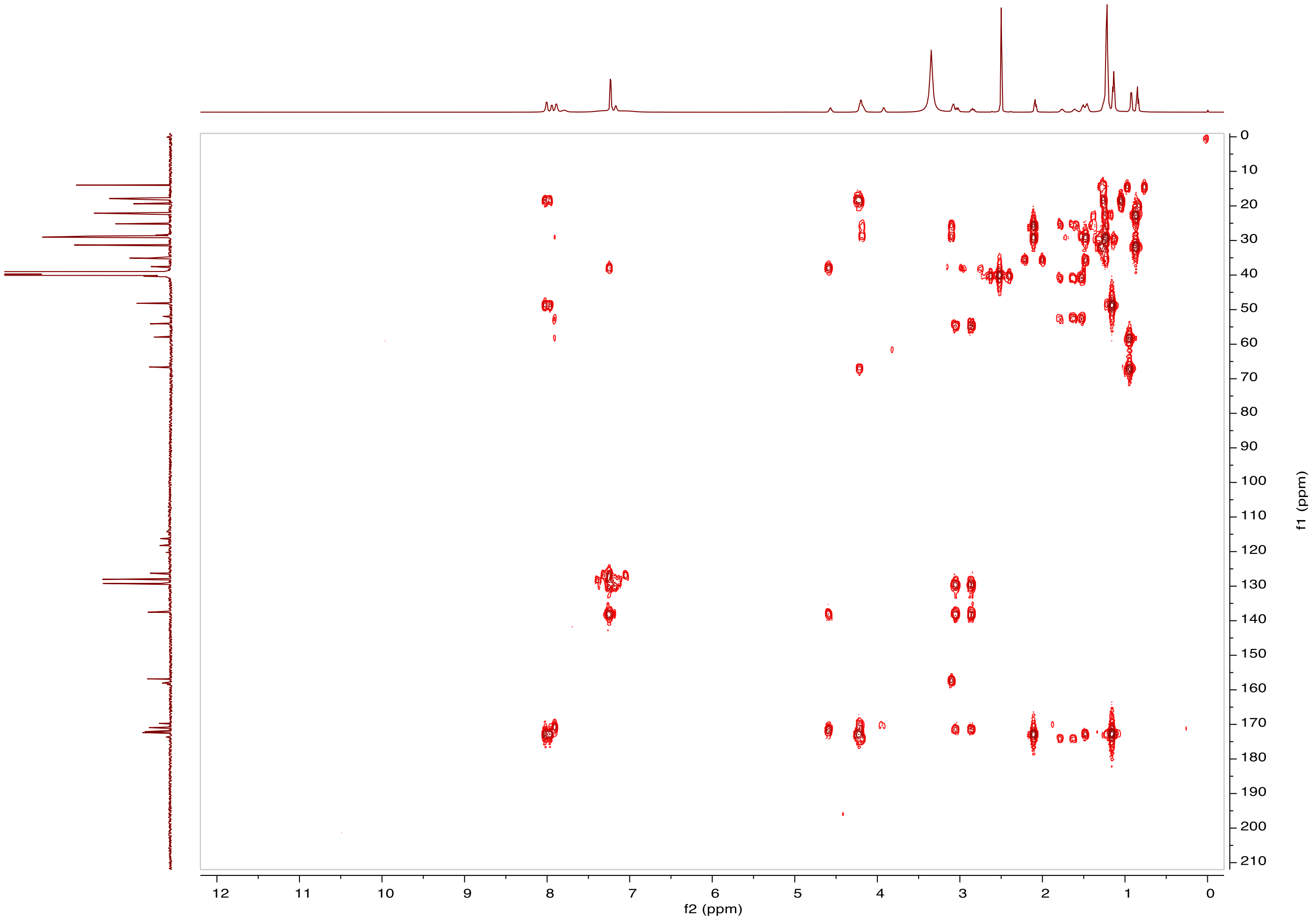

Figure S26. ^{1}H-^{13}C HMBC NMR spectrum of synAQU1-L in DMSO-d_6 (600 MHz)

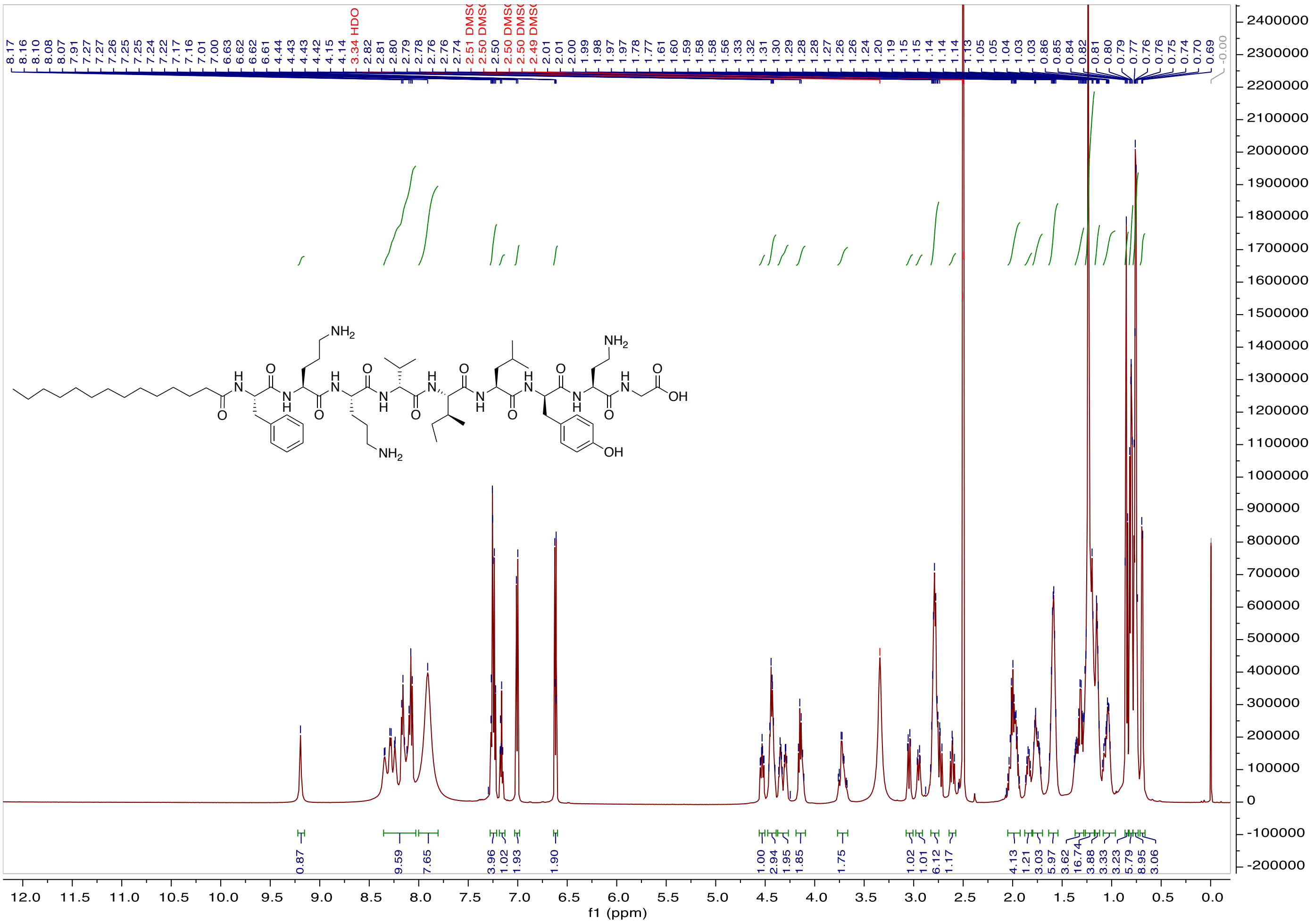

Figure S27. ^{1}H NMR spectrum of synAQU2-L in DMSO-d_6 (600 MHz)

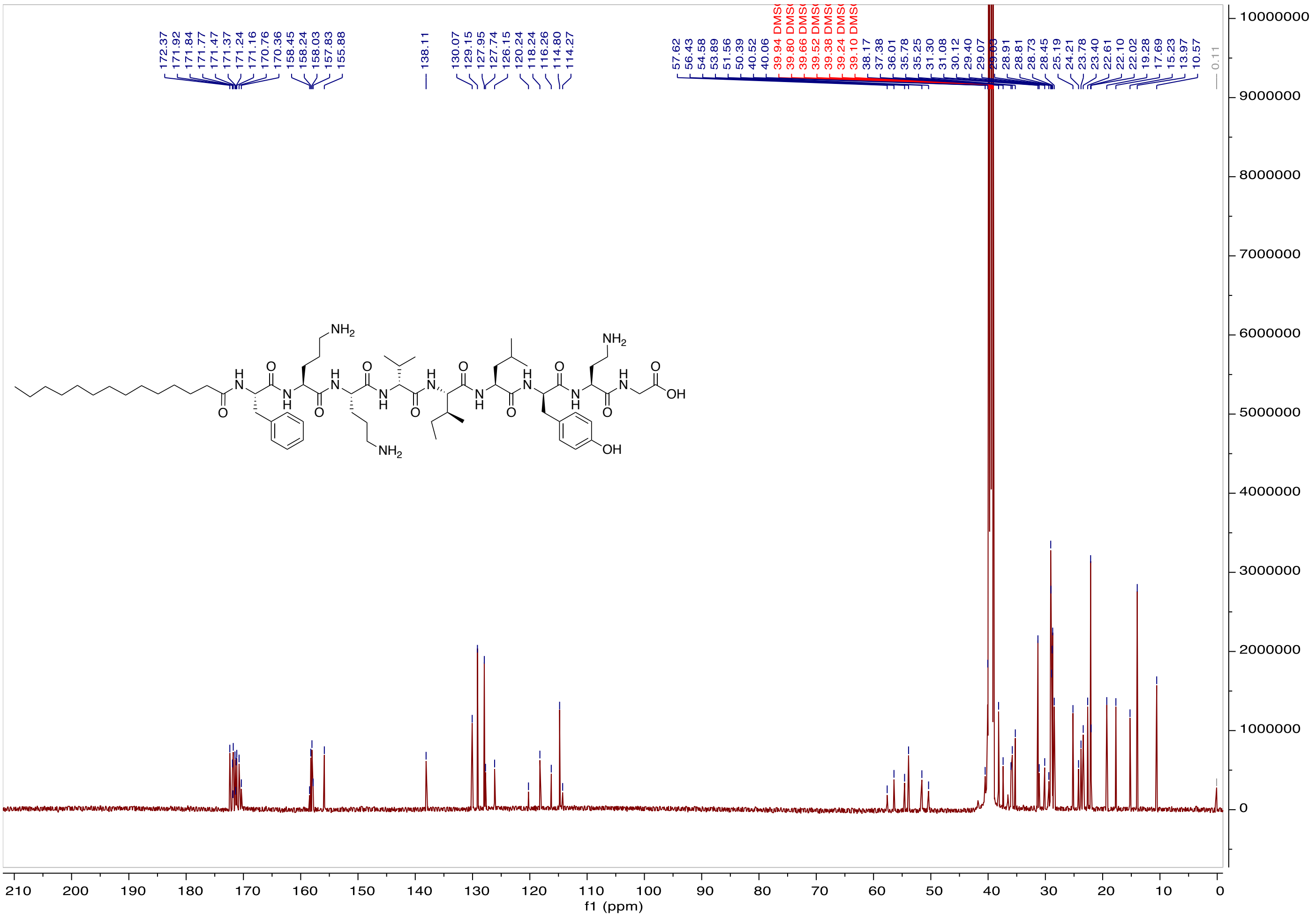

Figure S28. ^{13}C NMR spectrum of synAQU2-L in DMSO-d_6 (150 MHz)

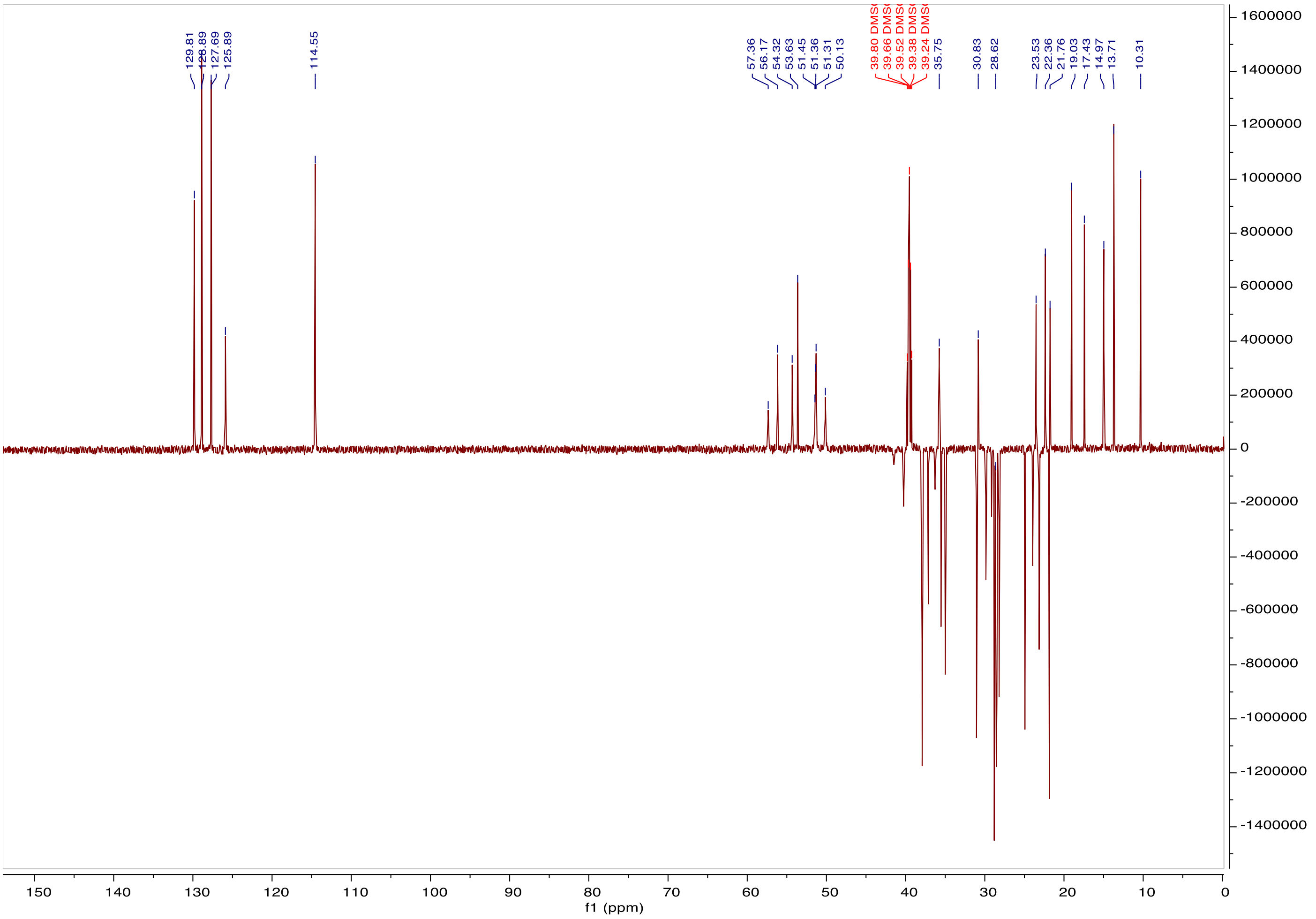

Figure S29. DEPT135 NMR spectrum of synAQU2-L in DMSO-d_6 (150 MHz)

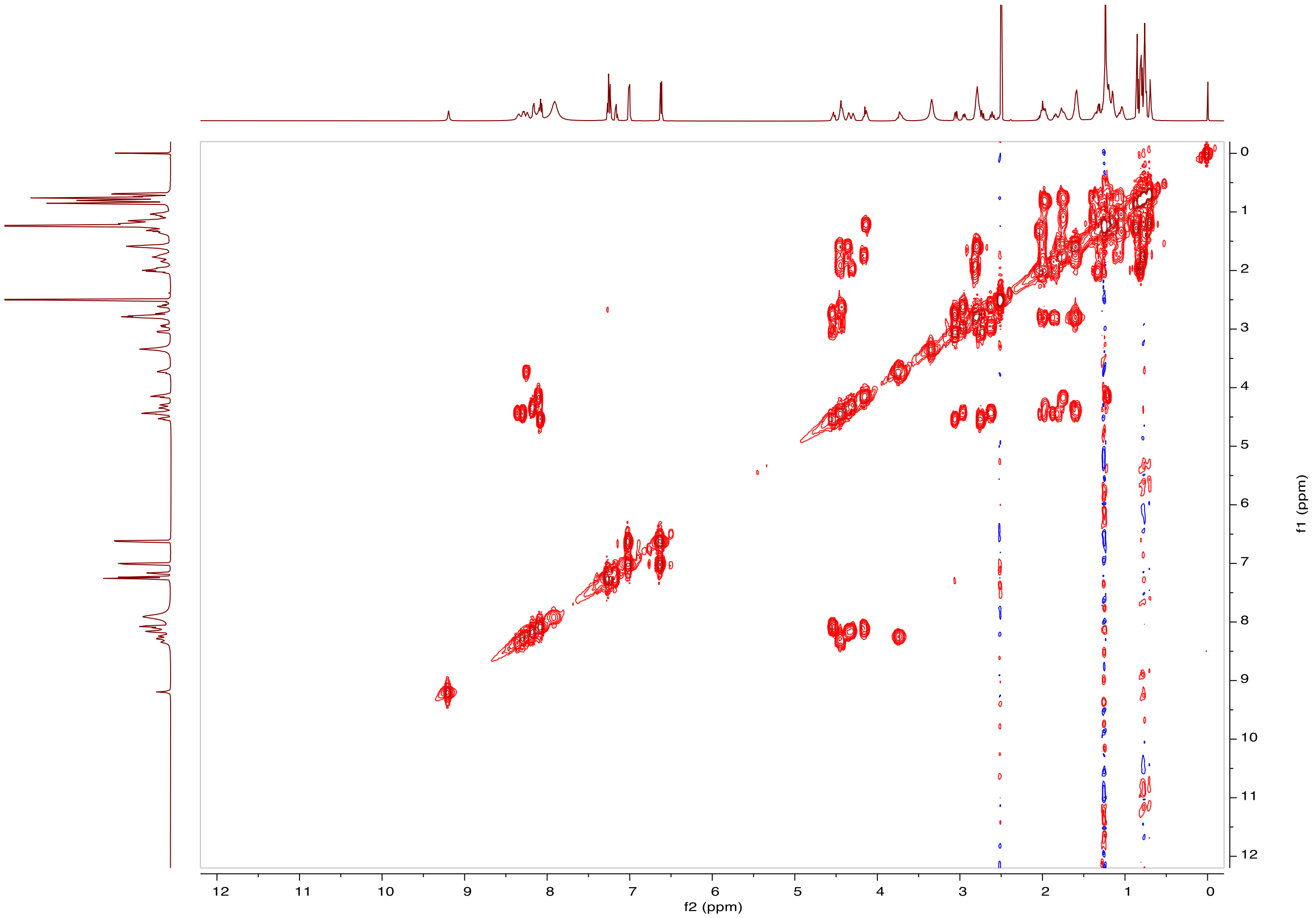

Figure S30. ^{1}H-^{1}H COSY NMR spectrum of synAQU2-L in DMSO-d_6 (600 MHz)

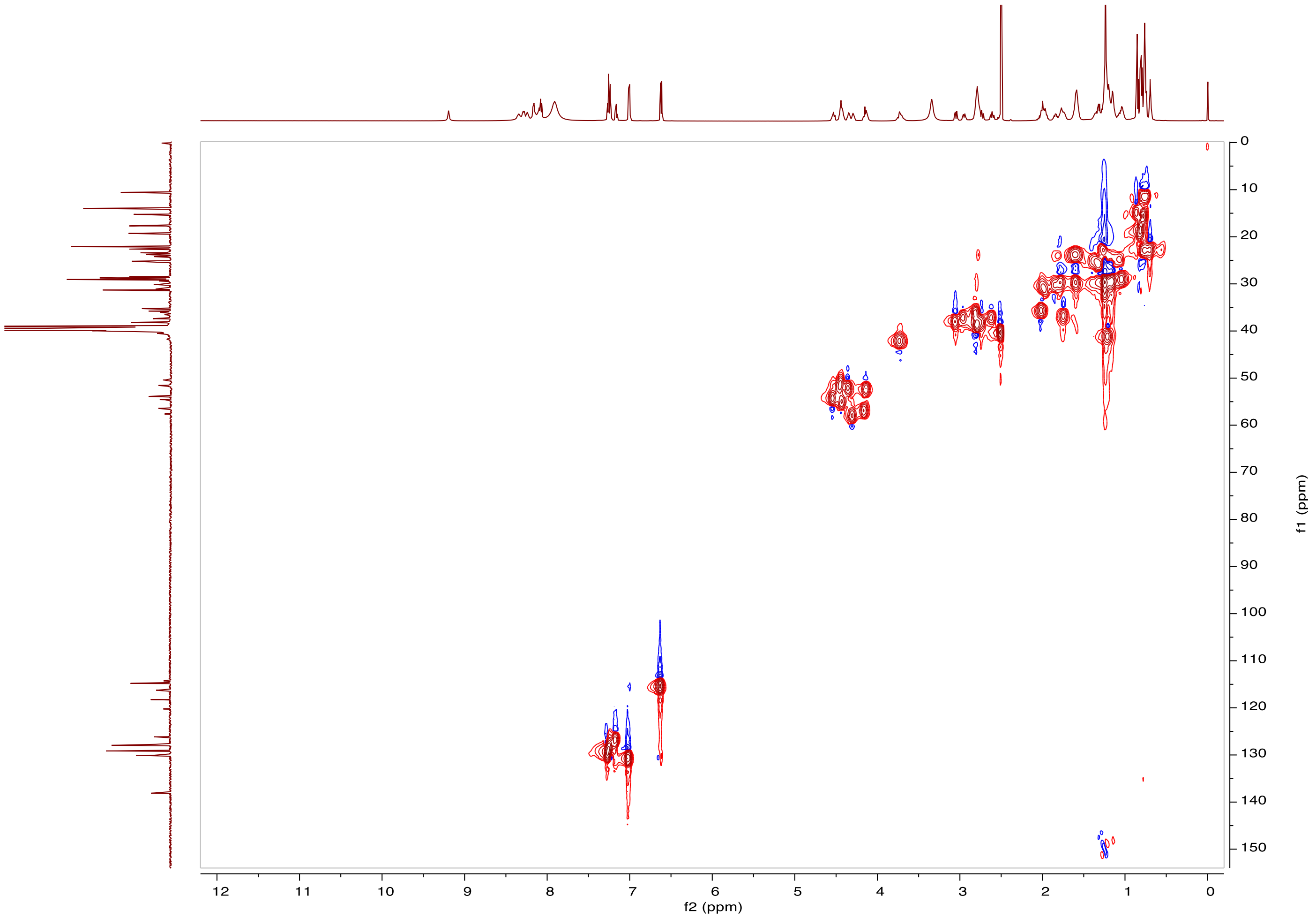

Figure S31. ^{1}H-^{13}C HSQC NMR spectrum of synAQU2-L in DMSO-d_6 (600 MHz)

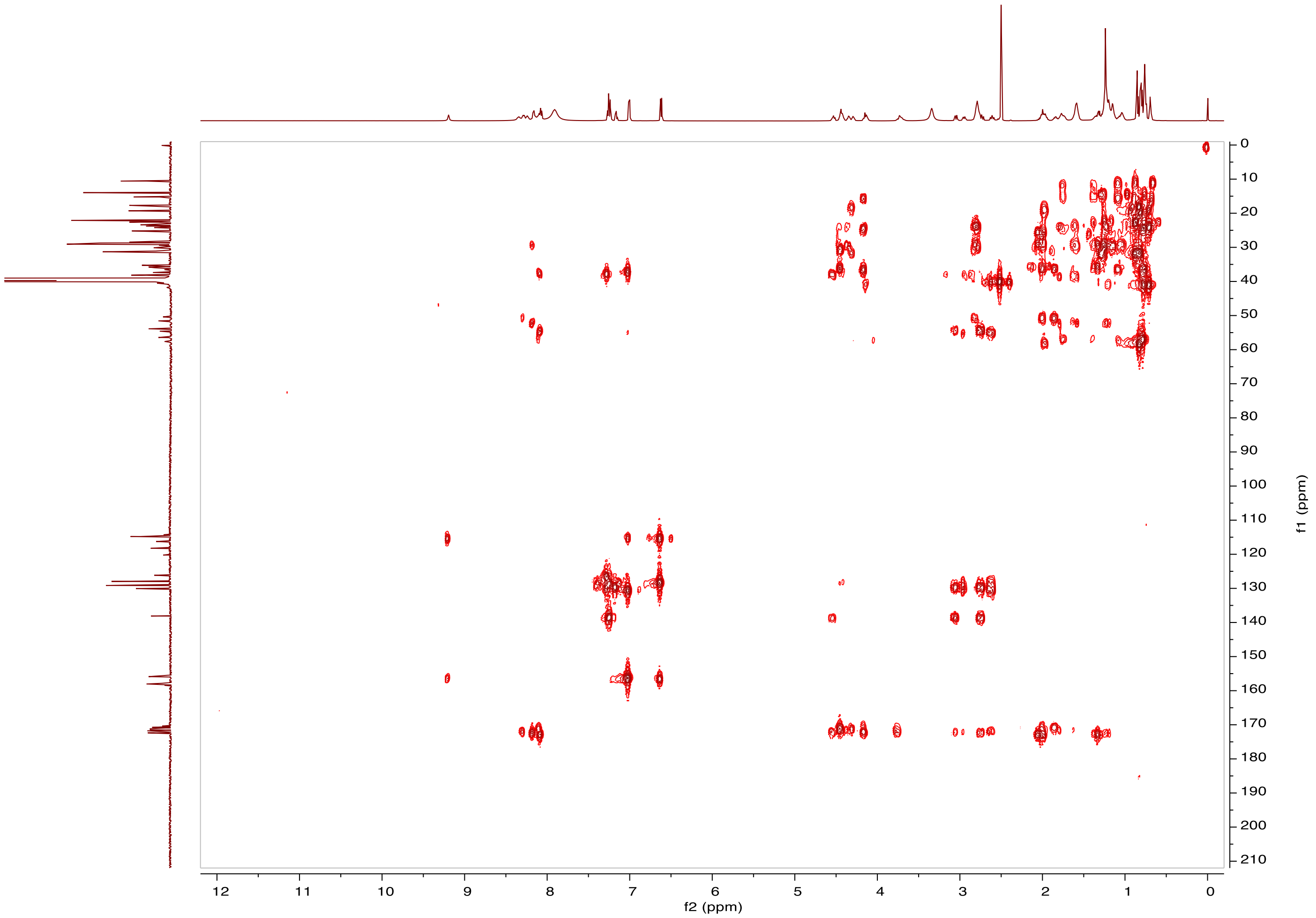

Figure S32. ^{1}H-^{13}C HMBC NMR spectrum of synAQU2-L in DMSO-d_6 (600 MHz)

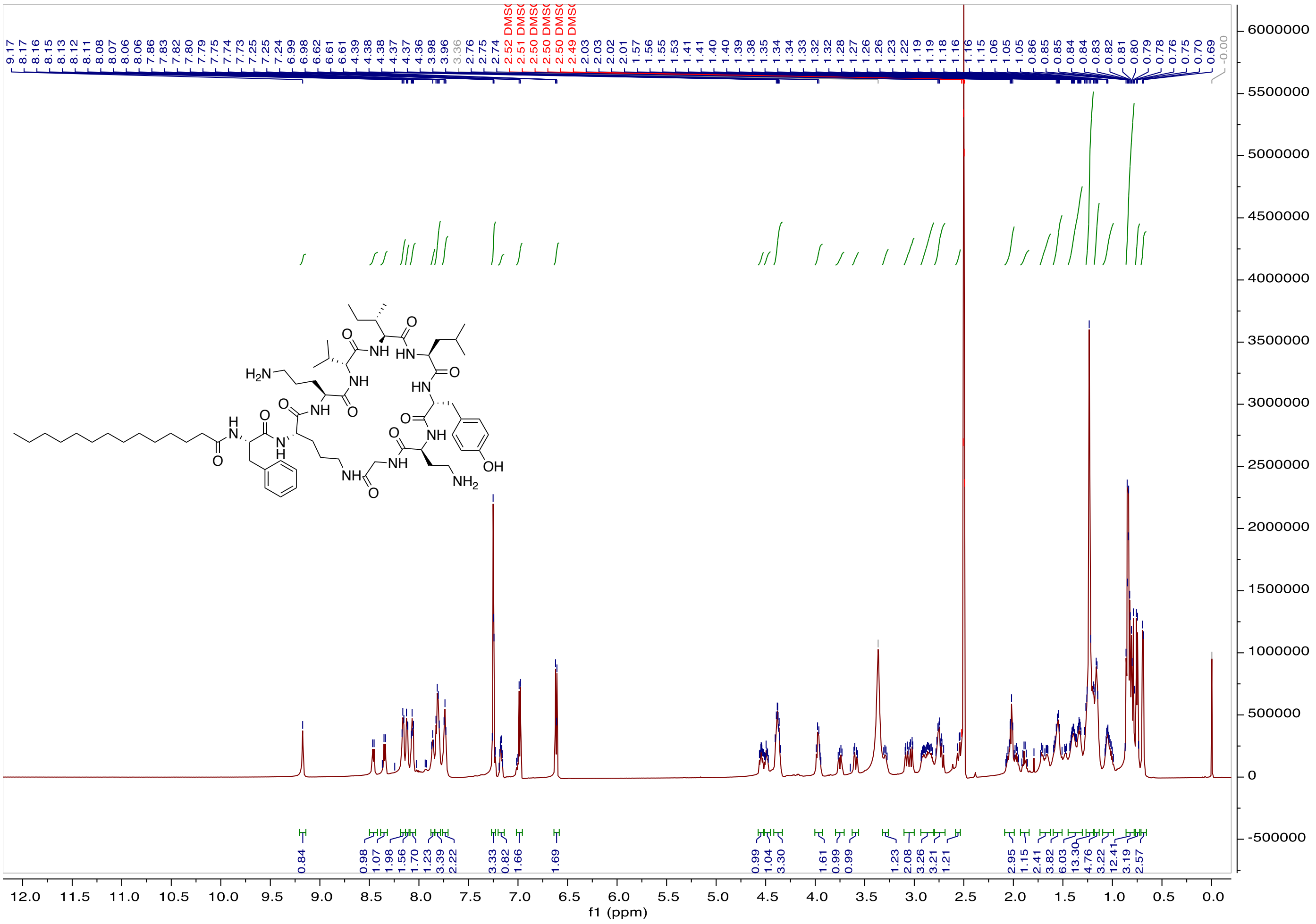

Figure S33. ^{1}H NMR spectrum of synAQU2-C2 in DMSO-d_6 (600 MHz)

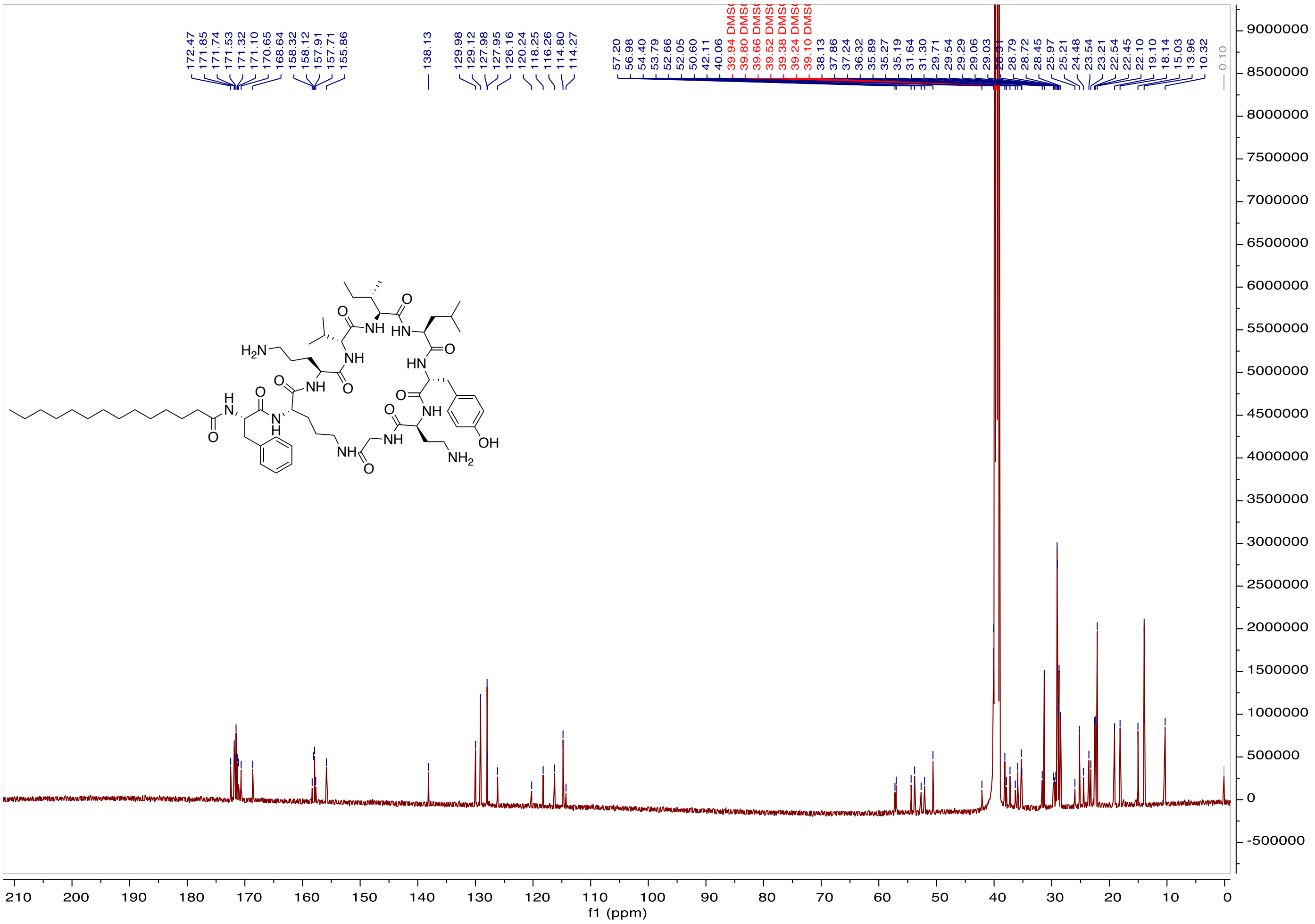

Figure S34. ^{13}C NMR spectrum of synAQU2-C2 in DMSO-d_6 (150 MHz)

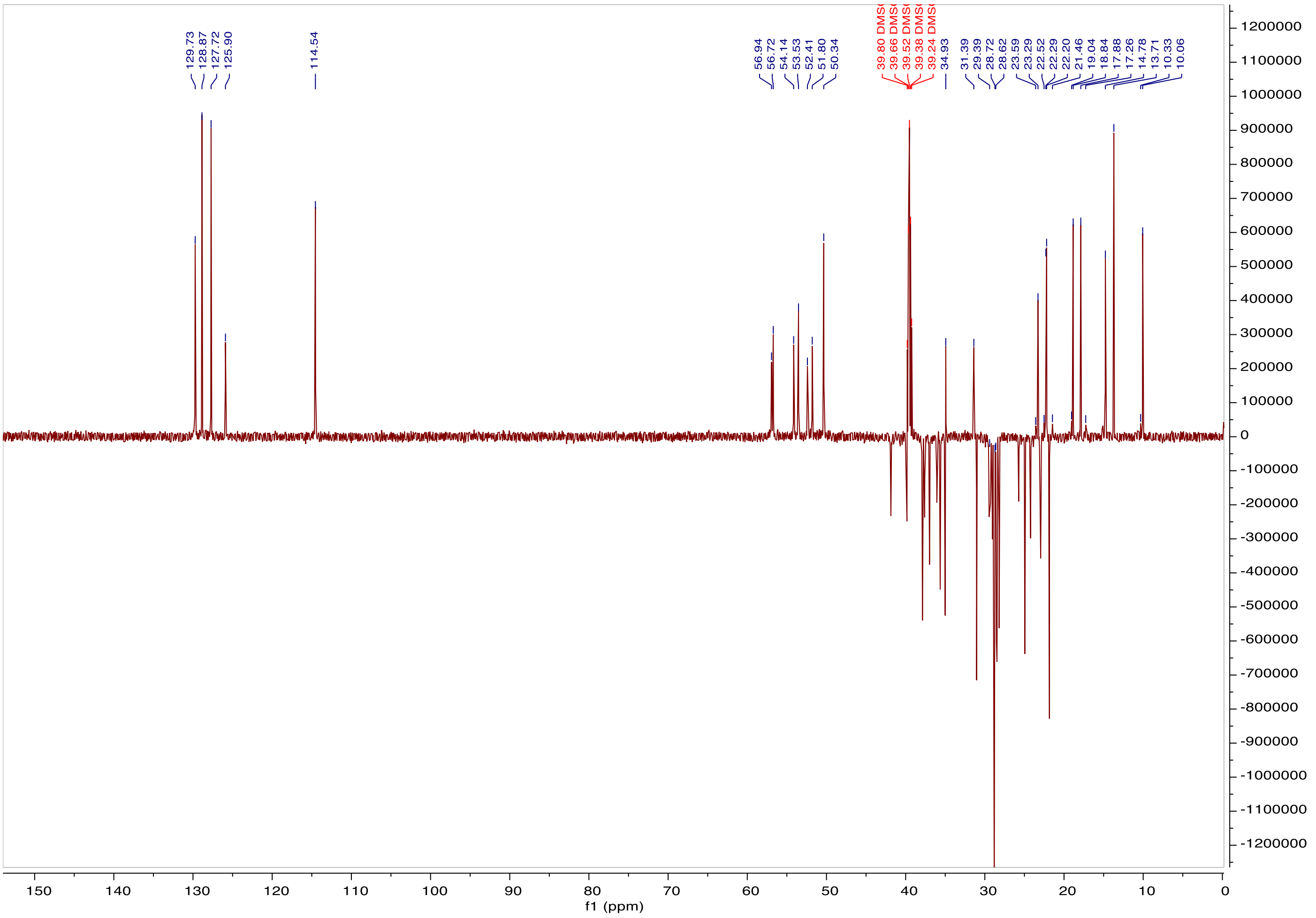

Figure S35. DEPT135 NMR spectrum of synAQU2-C2 in DMSO-d_6 (150 MHz)

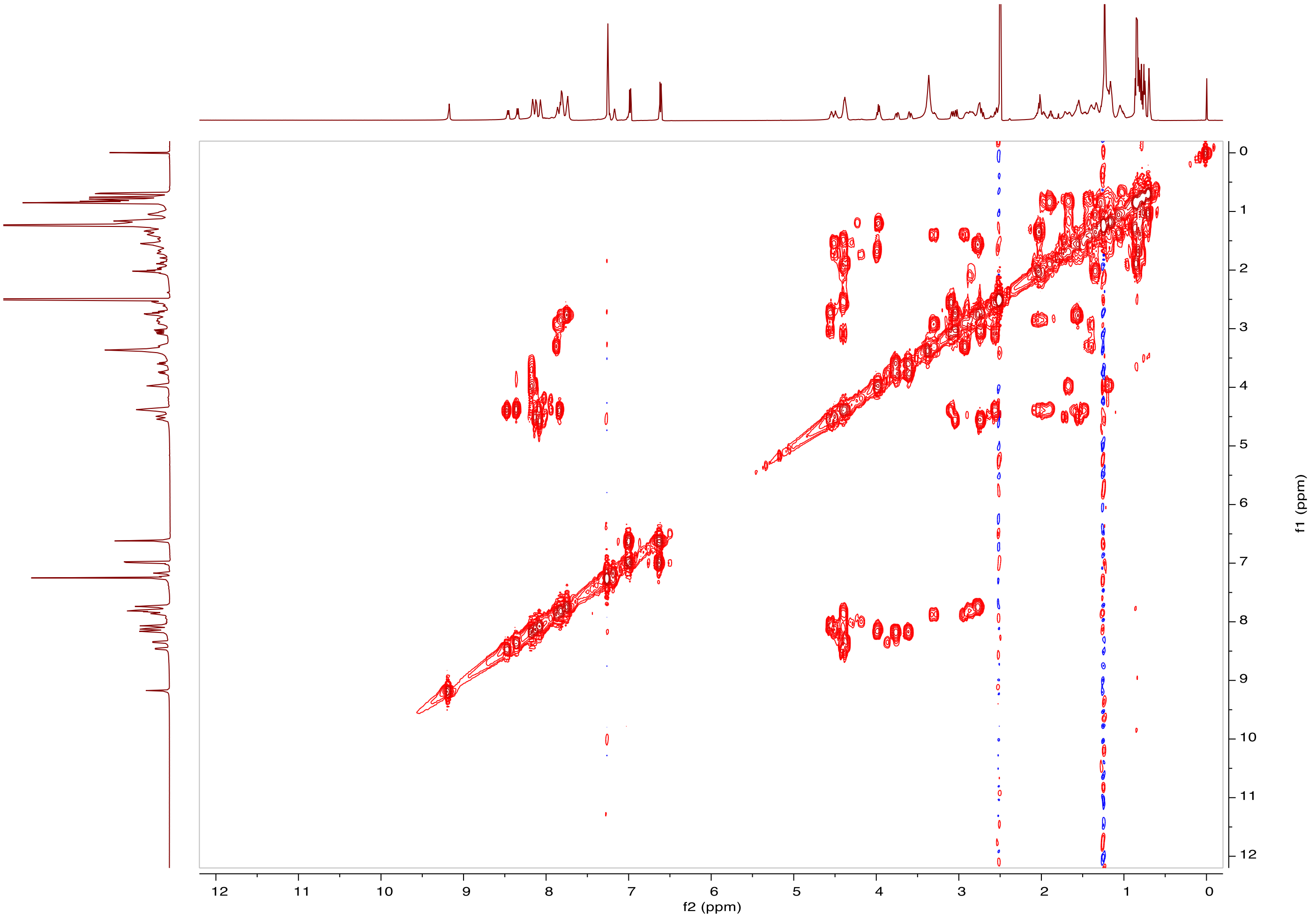

Figure S36. ^1H-^1H COSY NMR spectrum of synAQU2-C2 in DMSO-d_6 (600 MHz)

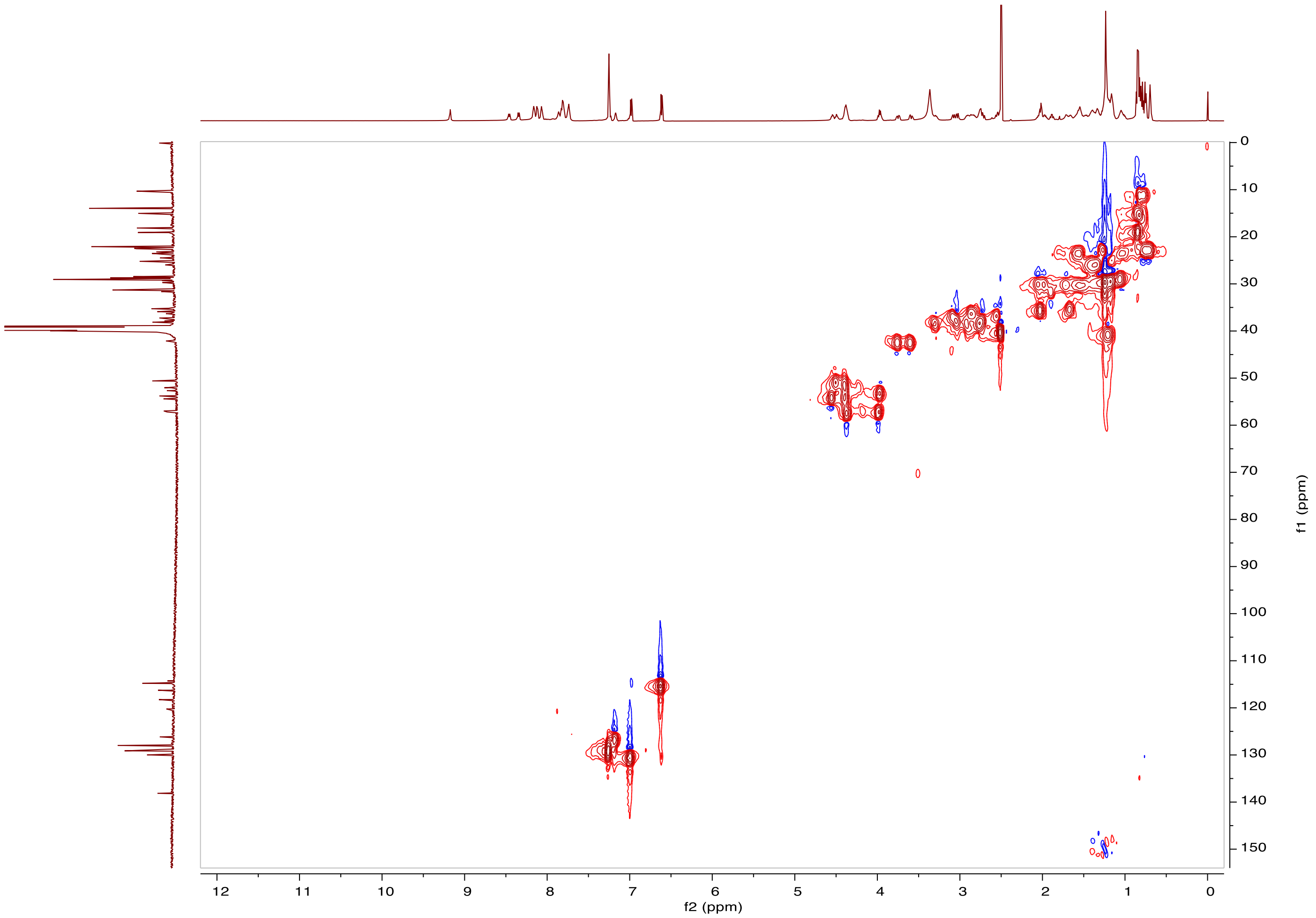

Figure S37. ^{1}H-^{13}C HSQC NMR spectrum of synAQU2-C2 in DMSO-d_6 (600 MHz)

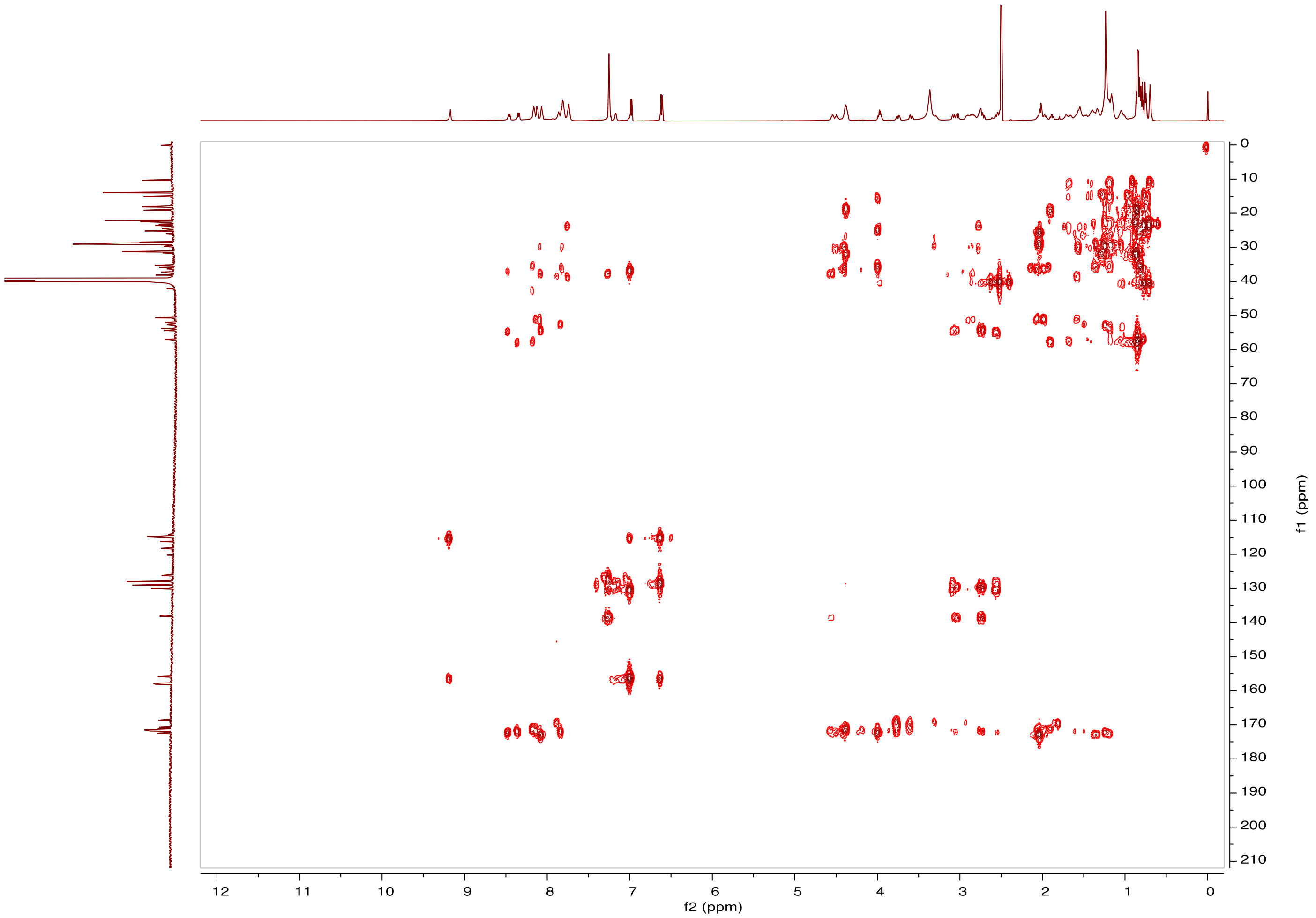

Figure S38. ^{1}H-^{13}C HMBC NMR spectrum of synAQU2-C2 in DMSO-d_6 (600 MHz)

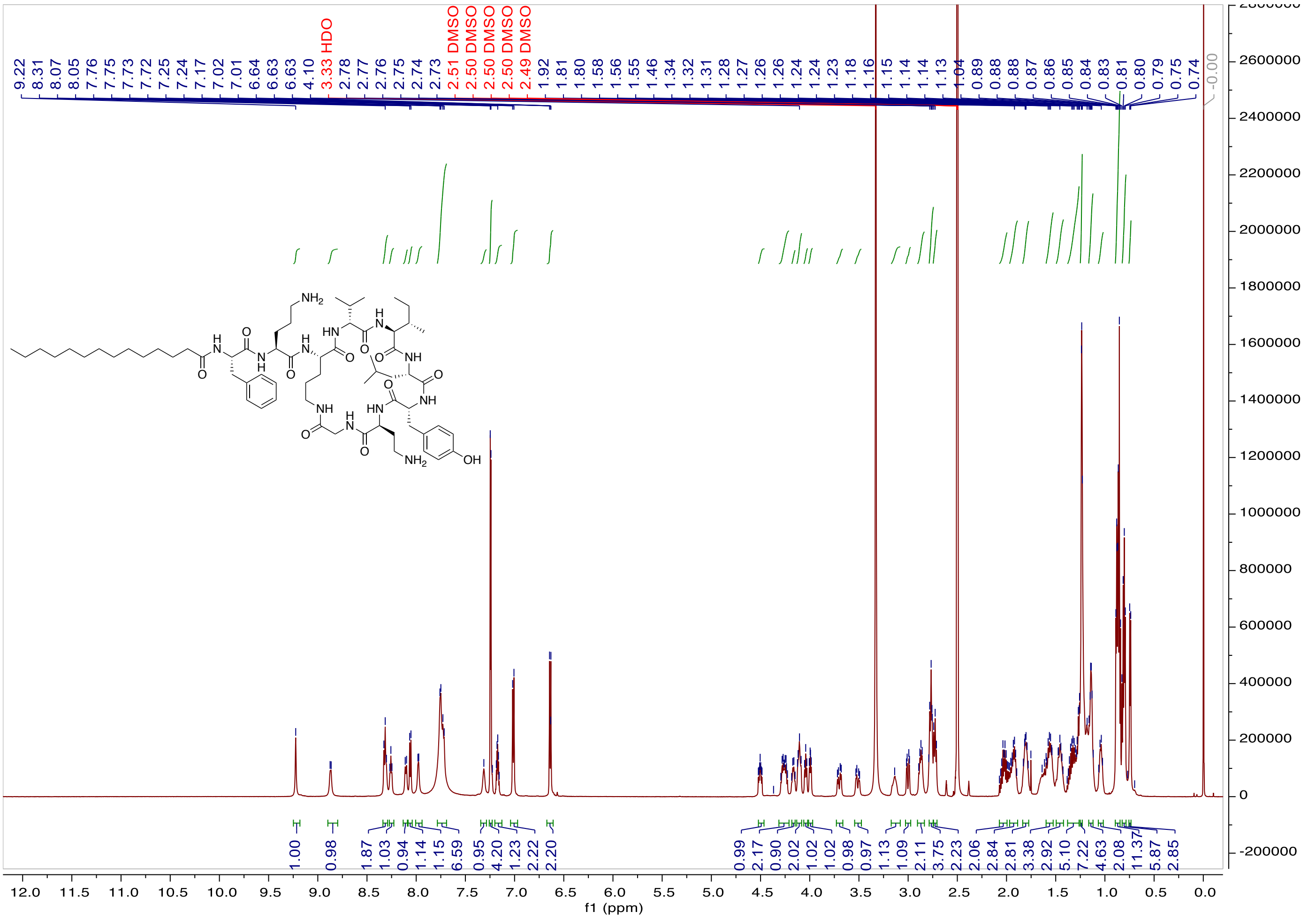

Figure S39. ^{1}H NMR spectrum of synAQU2-C3 in DMSO-d_6 (600 MHz)

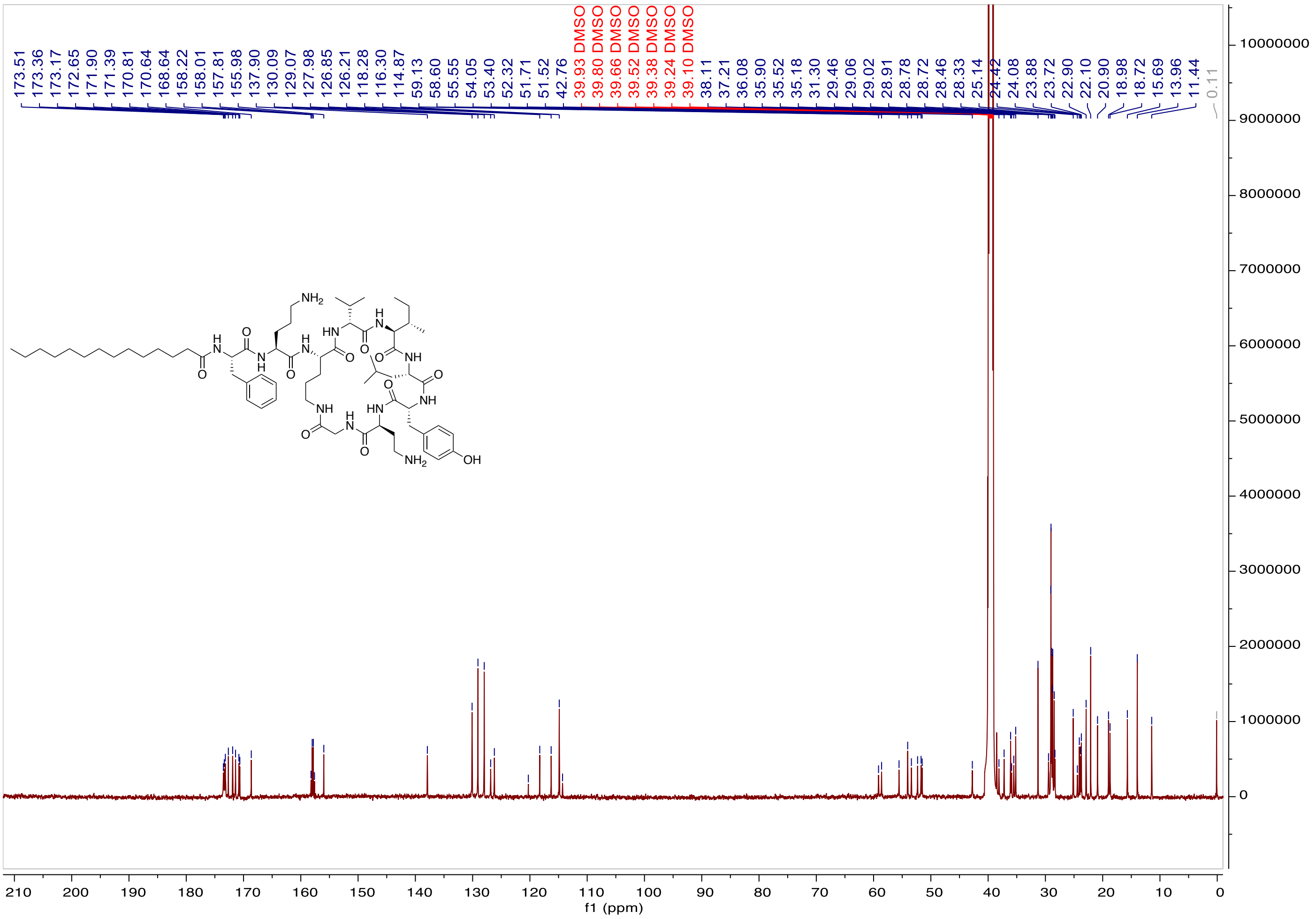

Figure S40. ^{13}C NMR spectrum of synAQU2-C3 in DMSO-d_6 (150 MHz)

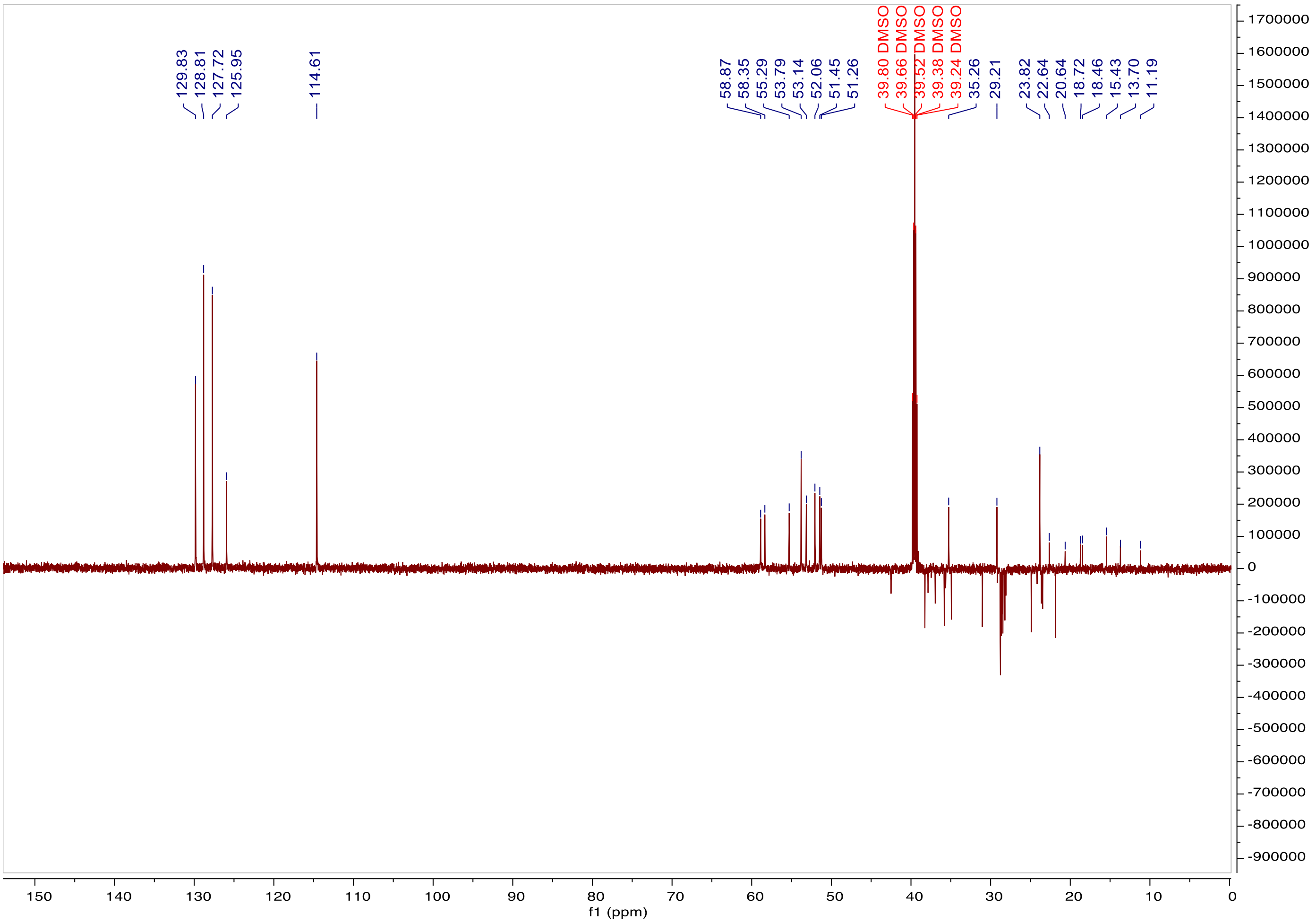

Figure S41. DEPT135 NMR spectrum of synAQU2-C3 in DMSO-d_6 (150 MHz)

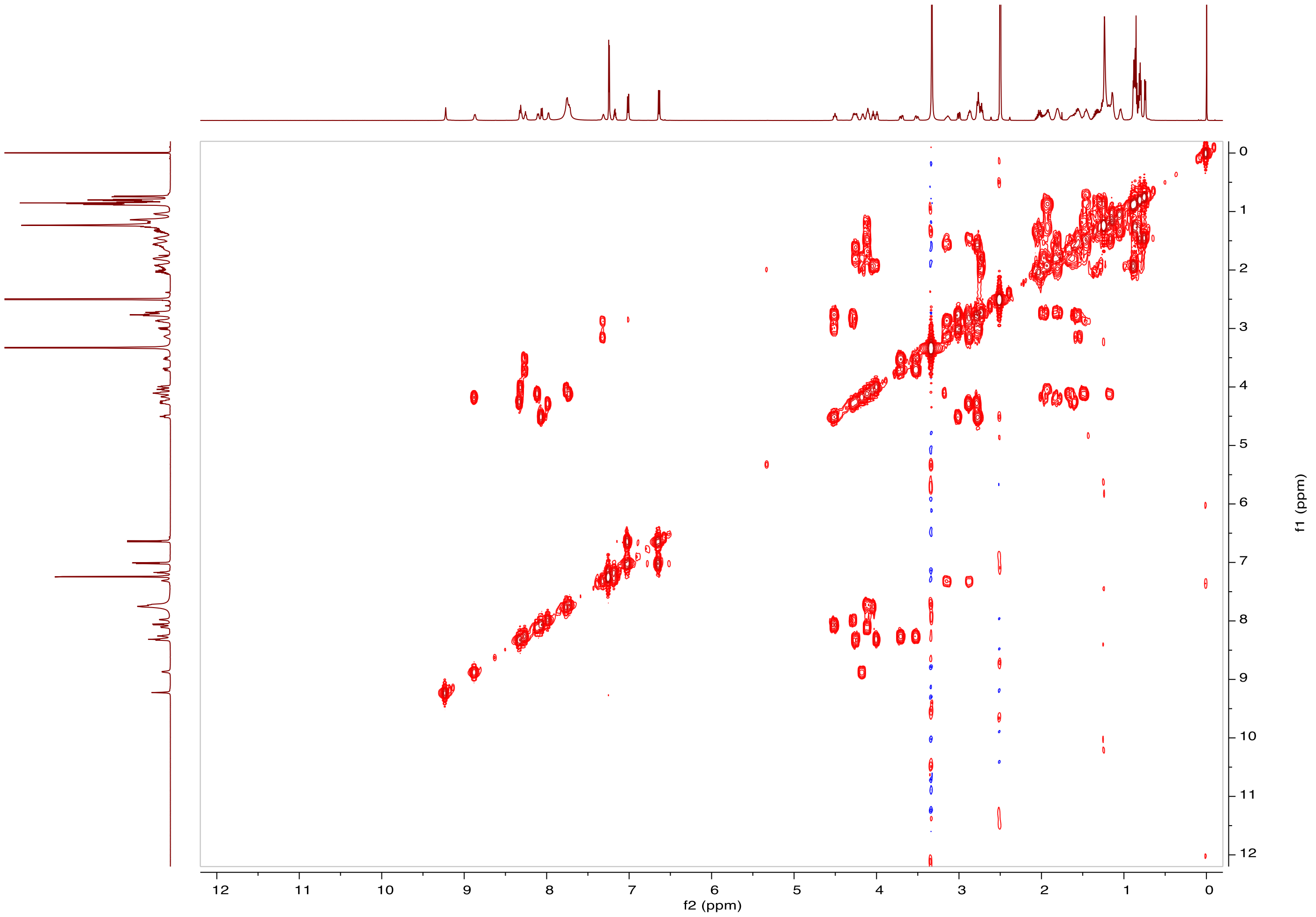

Figure S42. ^{1}H-^{1}H COSY NMR spectrum of synAQU2-C3 in DMSO-d_6 (600 MHz)

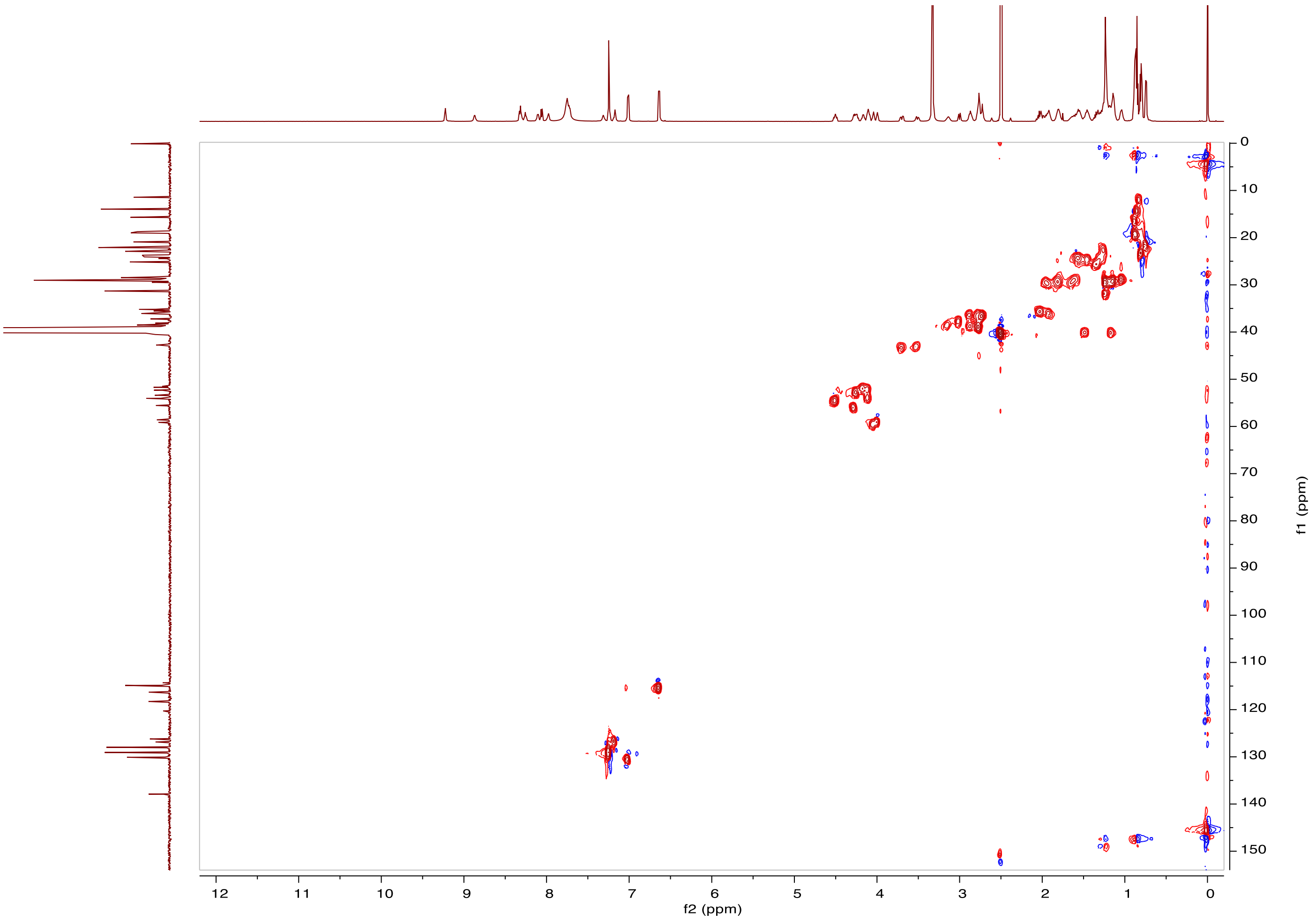

Figure S43. ^{1}H-^{13}C HSQC NMR spectrum of synAQU2-C3 in DMSO-d_6 (600 MHz)

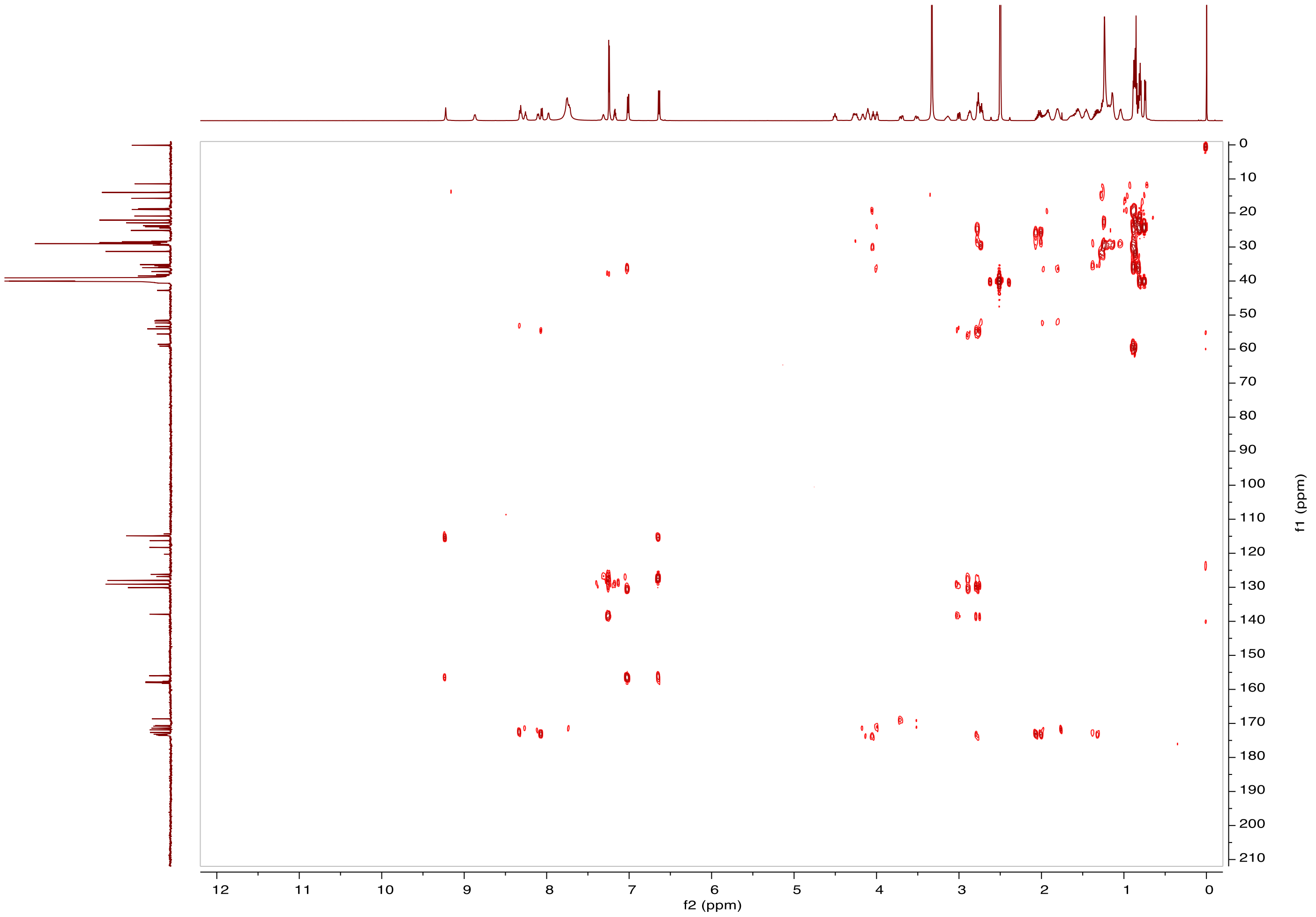

Figure S44. ^{1}H-^{13}C HMBC NMR spectrum of synAQU2-C3 in DMSO-d_6 (600 MHz)

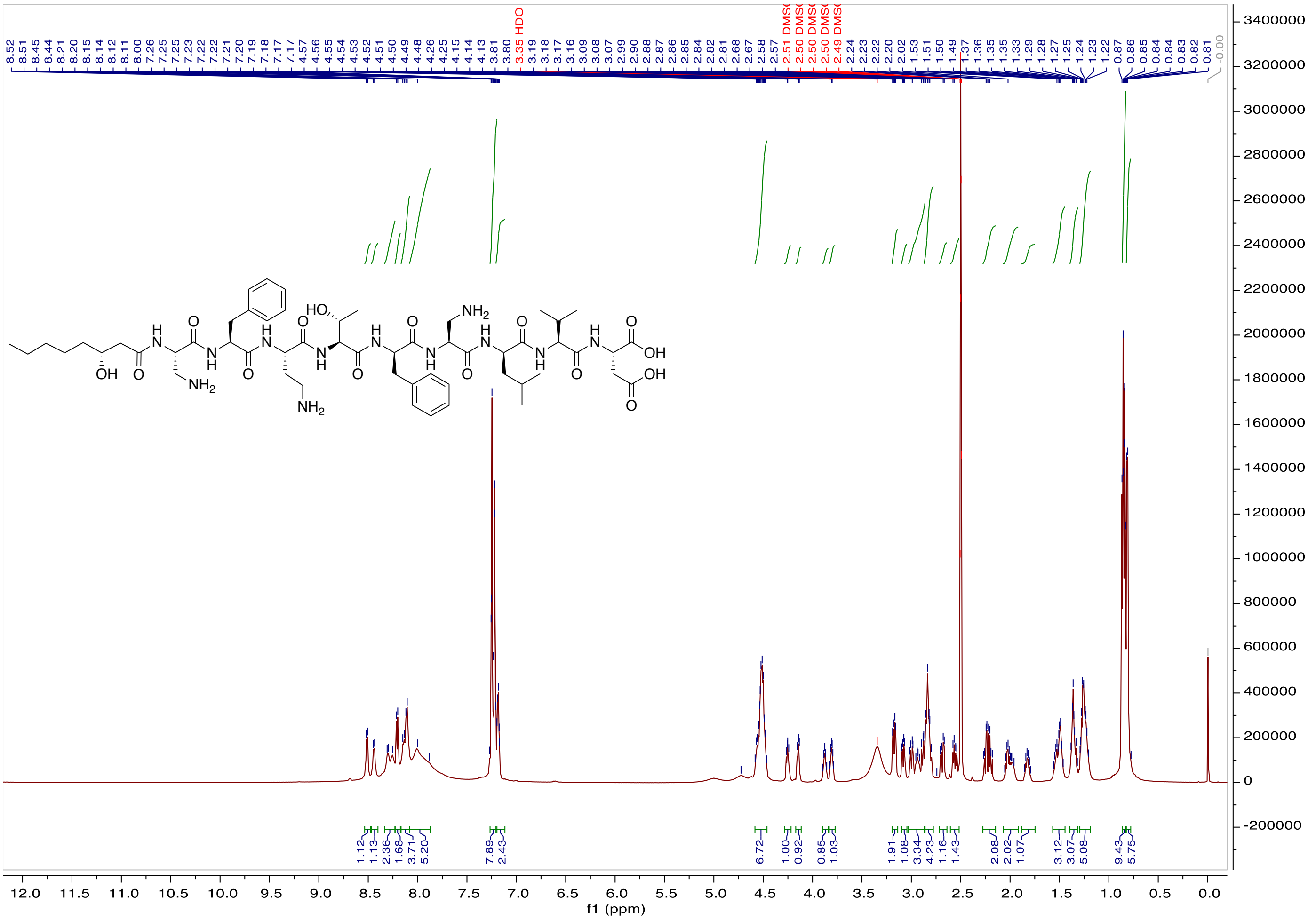

Figure S45. ^{1}H NMR spectrum of synAQU3-L in DMSO-d_6 (600 MHz)

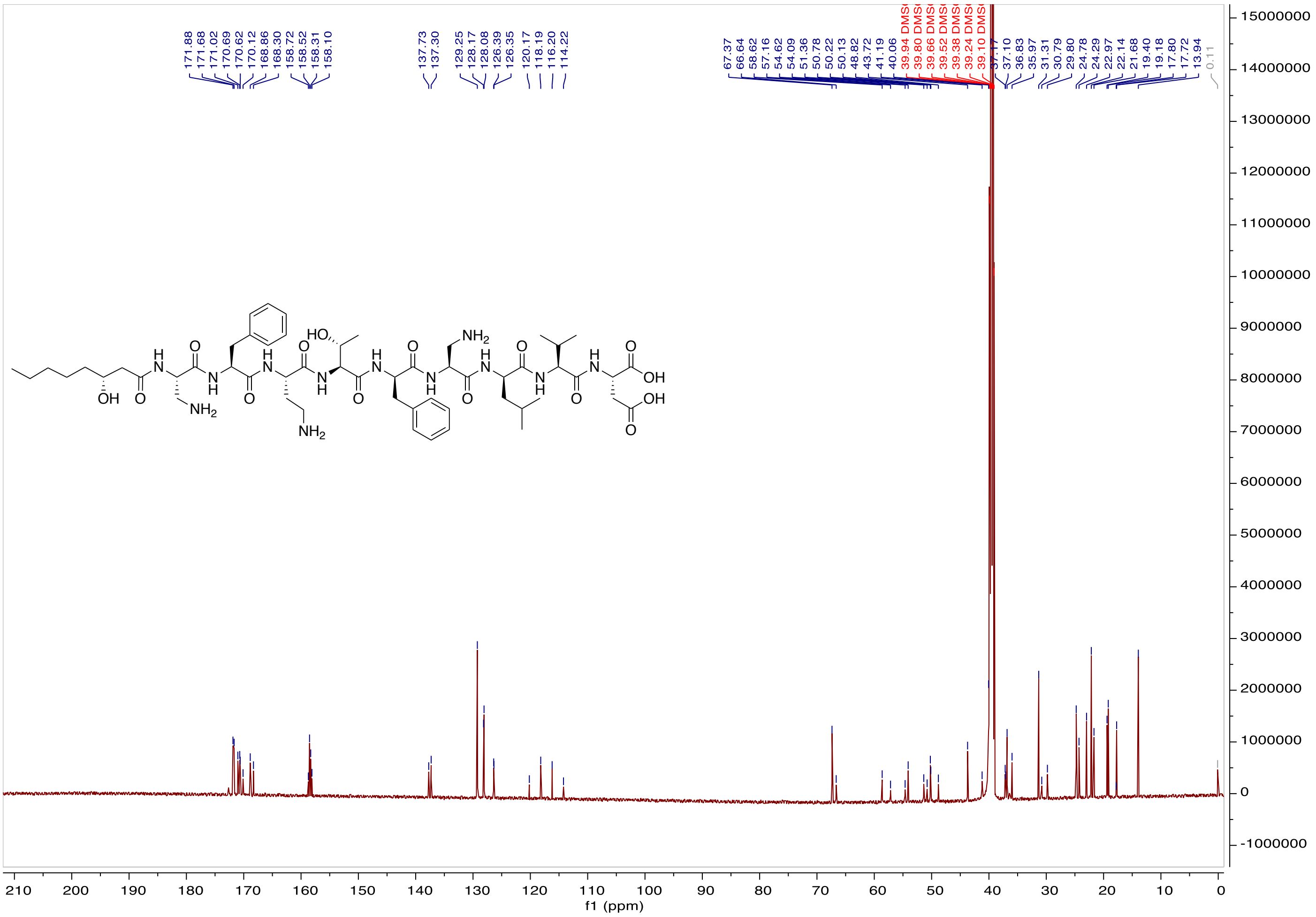

Figure S46. ^{13}C NMR spectrum of synAQU3-L in DMSO-d_6 (150 MHz)

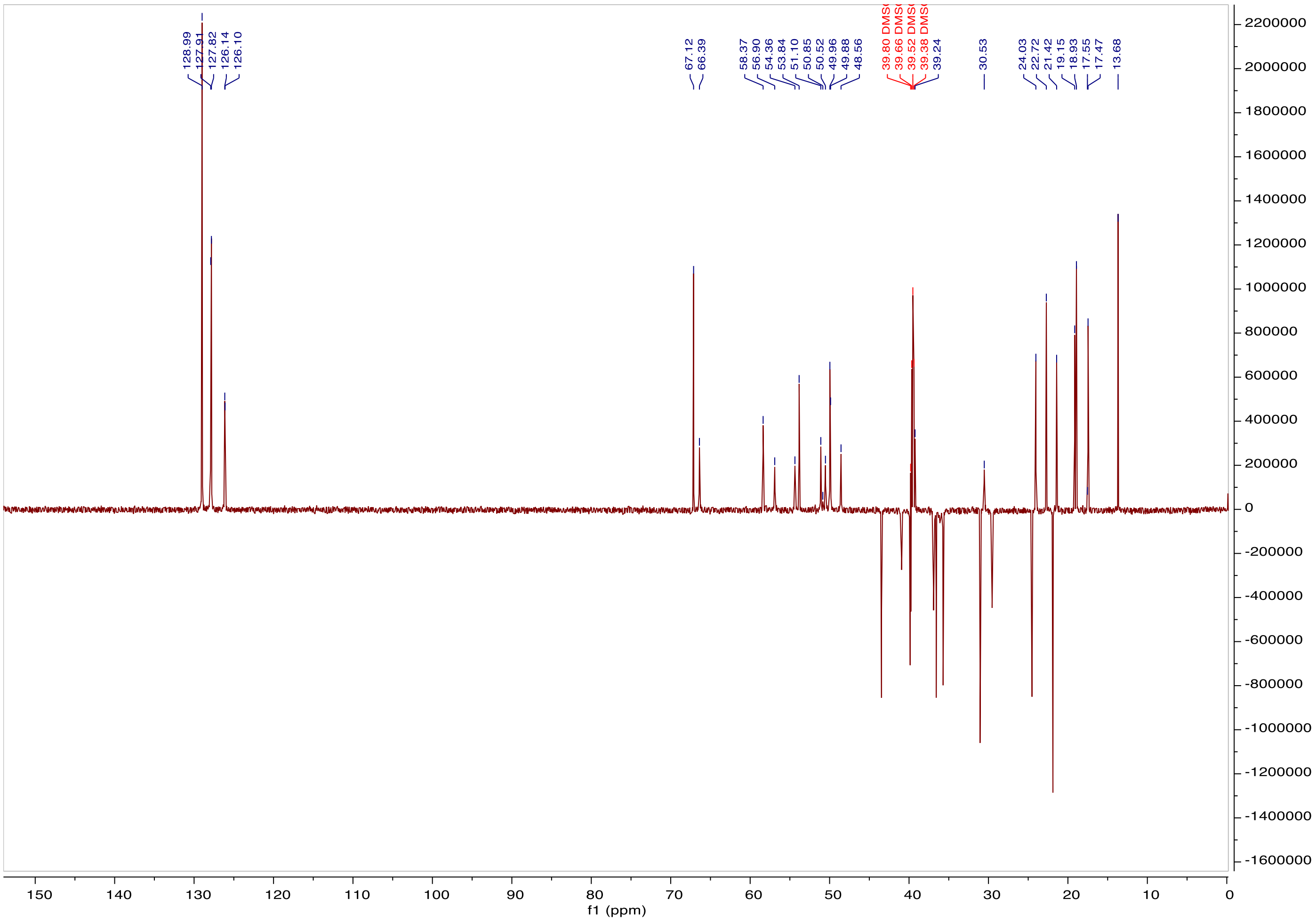

Figure S47. DEPT135 NMR spectrum of synAQU3-L in DMSO-d_6 (150 MHz)

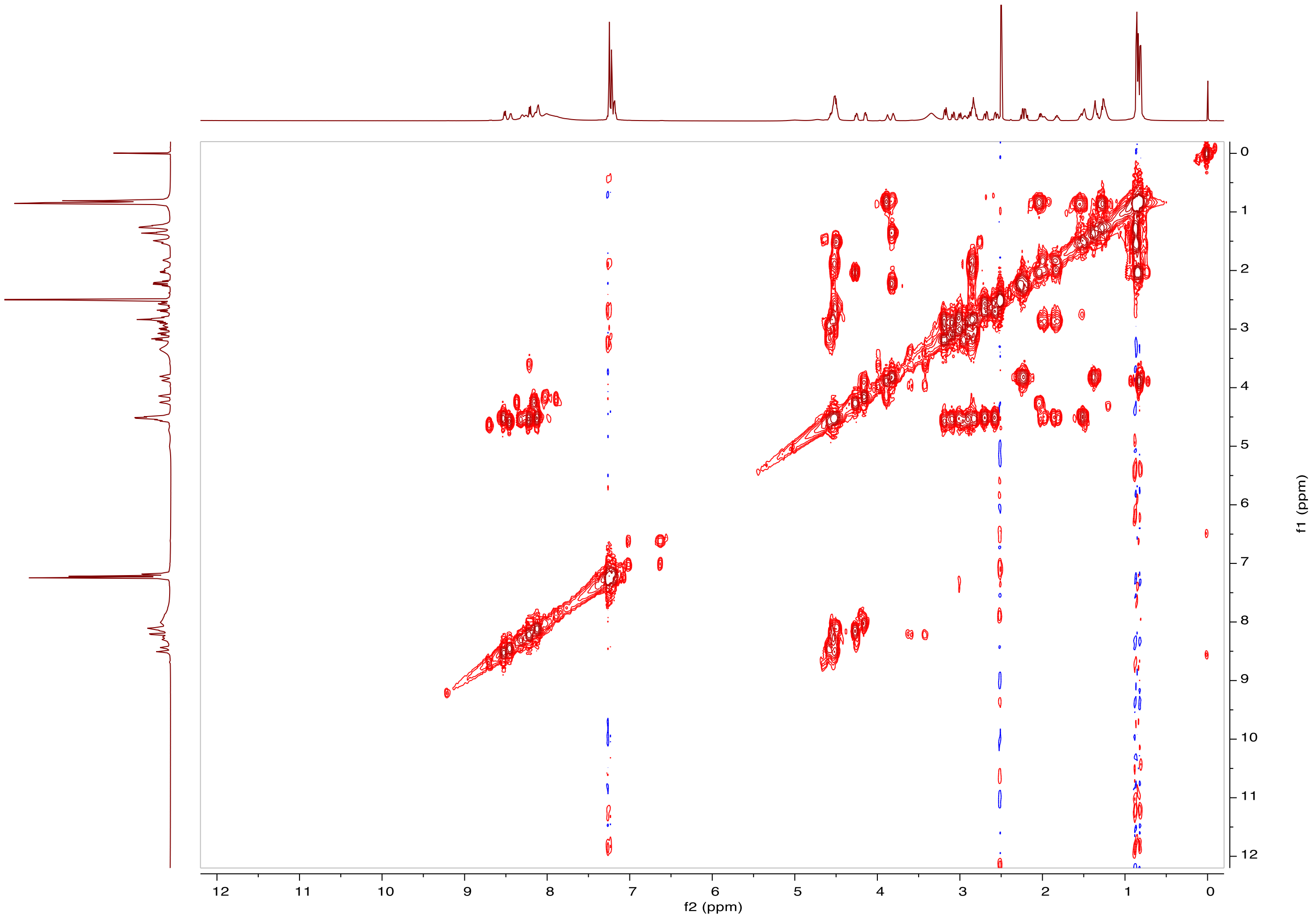

Figure S48. ^{1}H-^{1}H COSY NMR spectrum of synAQU3-L in DMSO-d_6 (600 MHz)

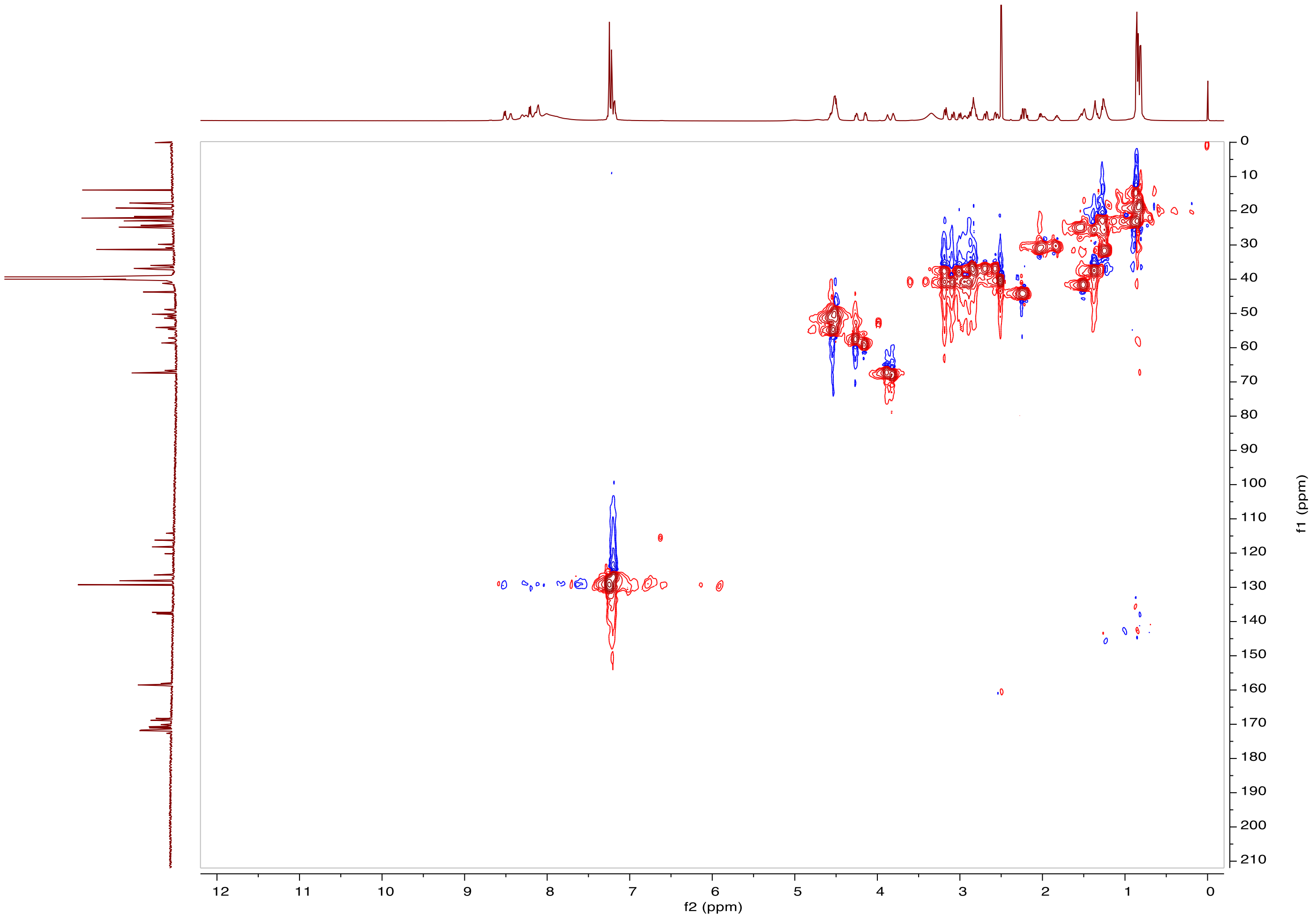

Figure S49. ^{1}H-^{13}C HSQC NMR spectrum of synAQU3-L in DMSO-d_6 (600 MHz)

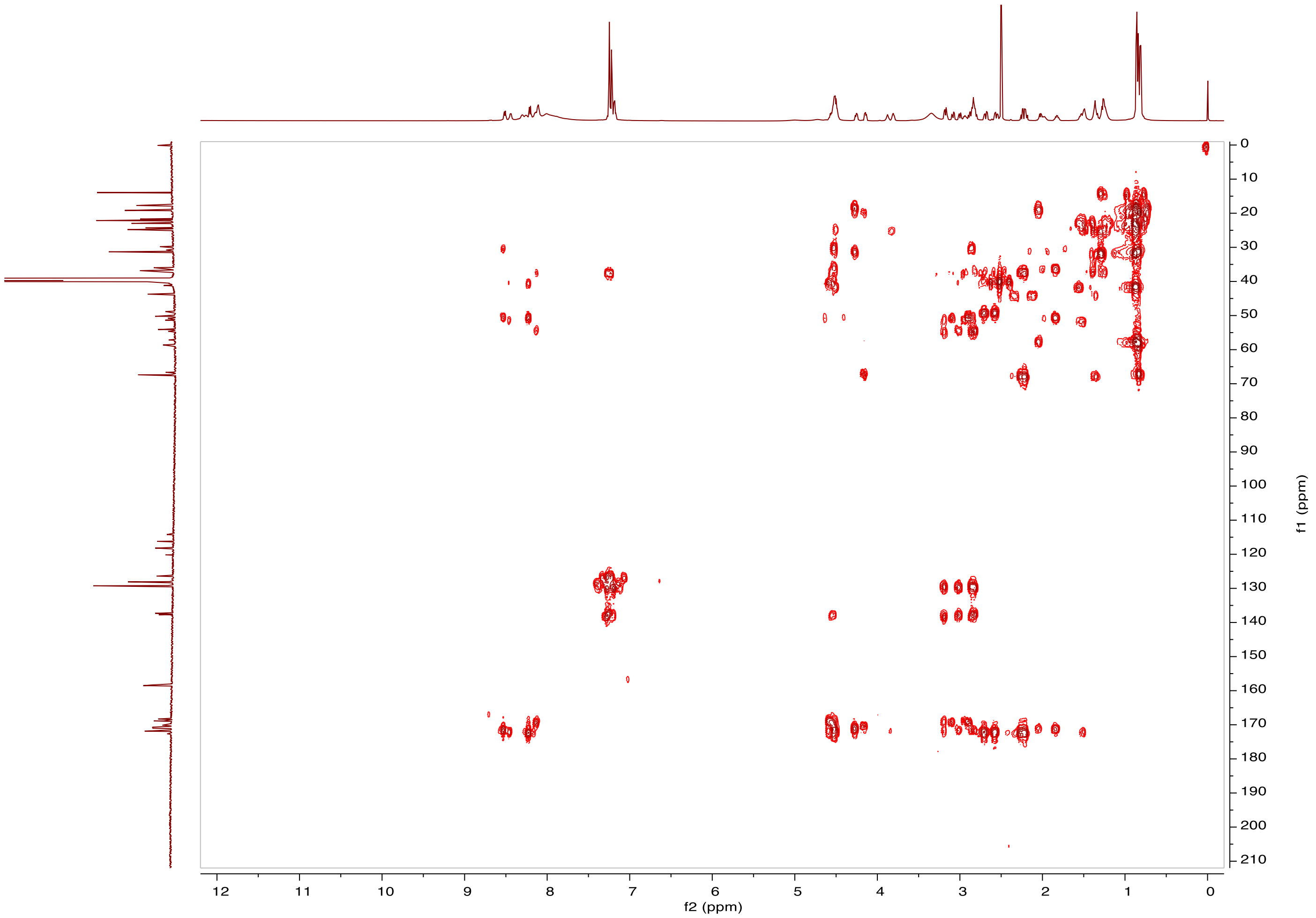

Figure S50. ^{1}H-^{13}C HMBC NMR spectrum of synAQU3-L in DMSO-d_6 (600 MHz)

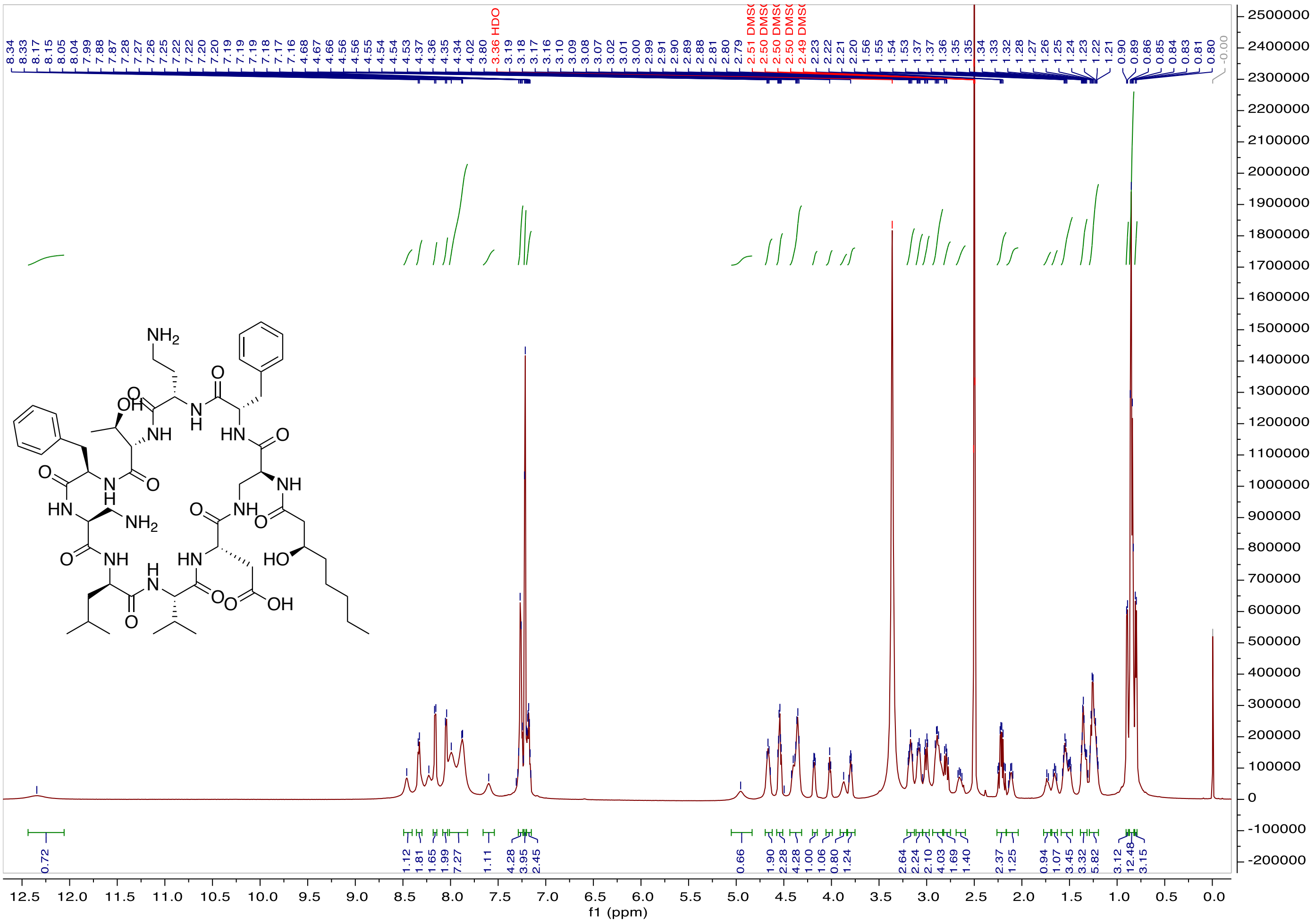

Figure S51. ^{1}H NMR spectrum of synAQU3-C1 in DMSO-d_6 (600 MHz)

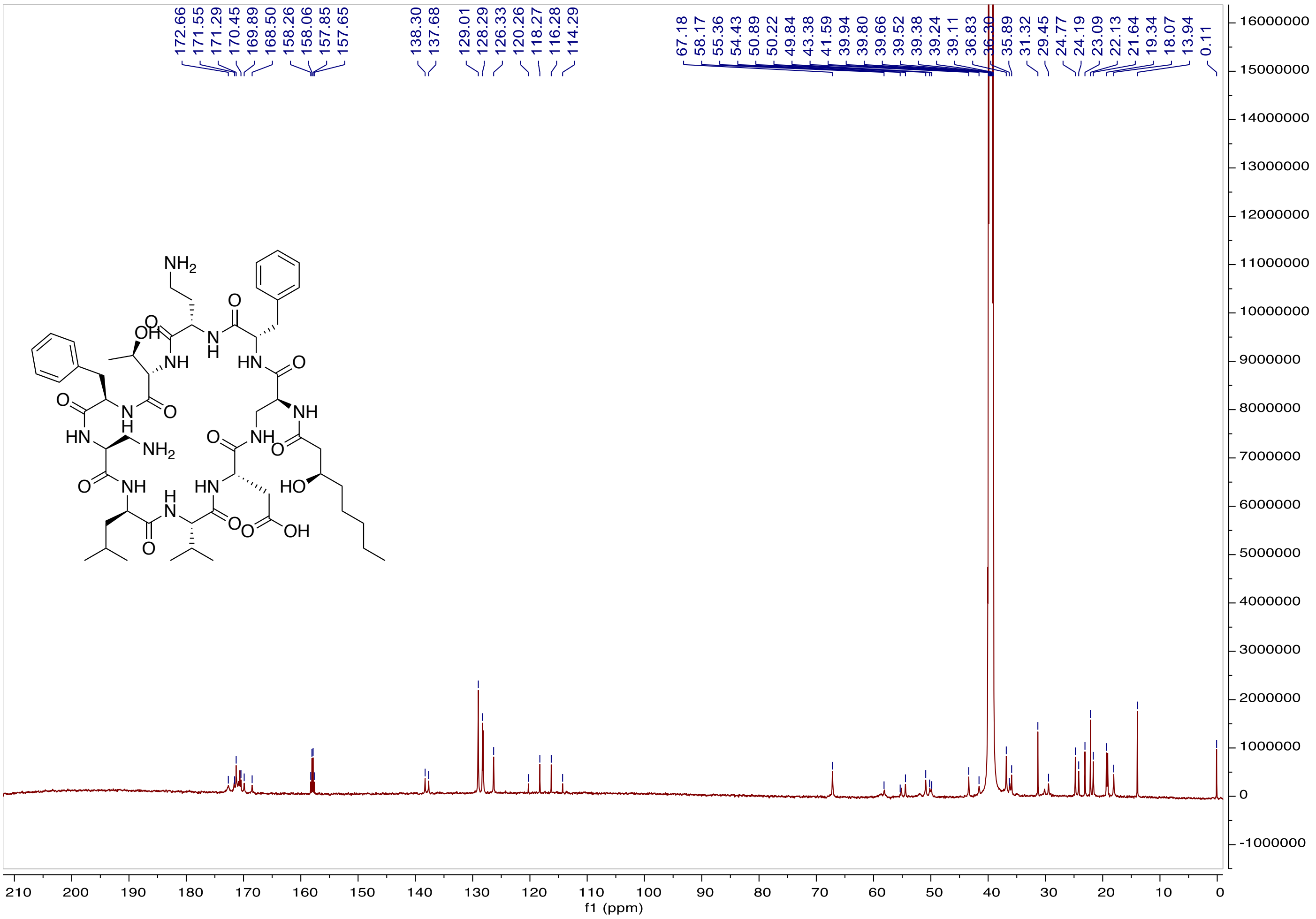

Figure S52. ^{13}C NMR spectrum of synAQU3-C1 in DMSO-d_6 (150 MHz)

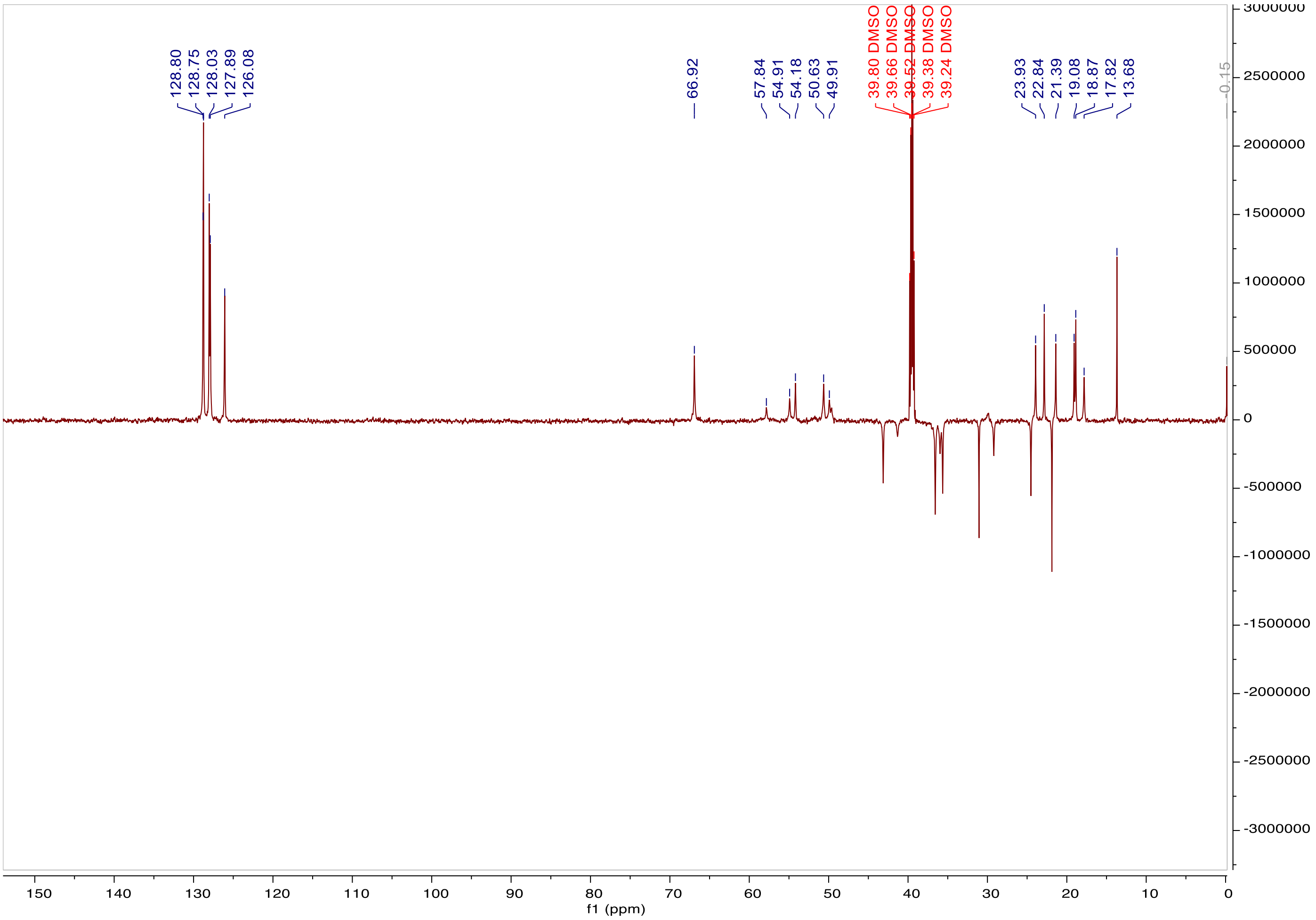

Figure S53. DEPT135 NMR spectrum of synAQU3-C1 in DMSO-d_6 (150 MHz)

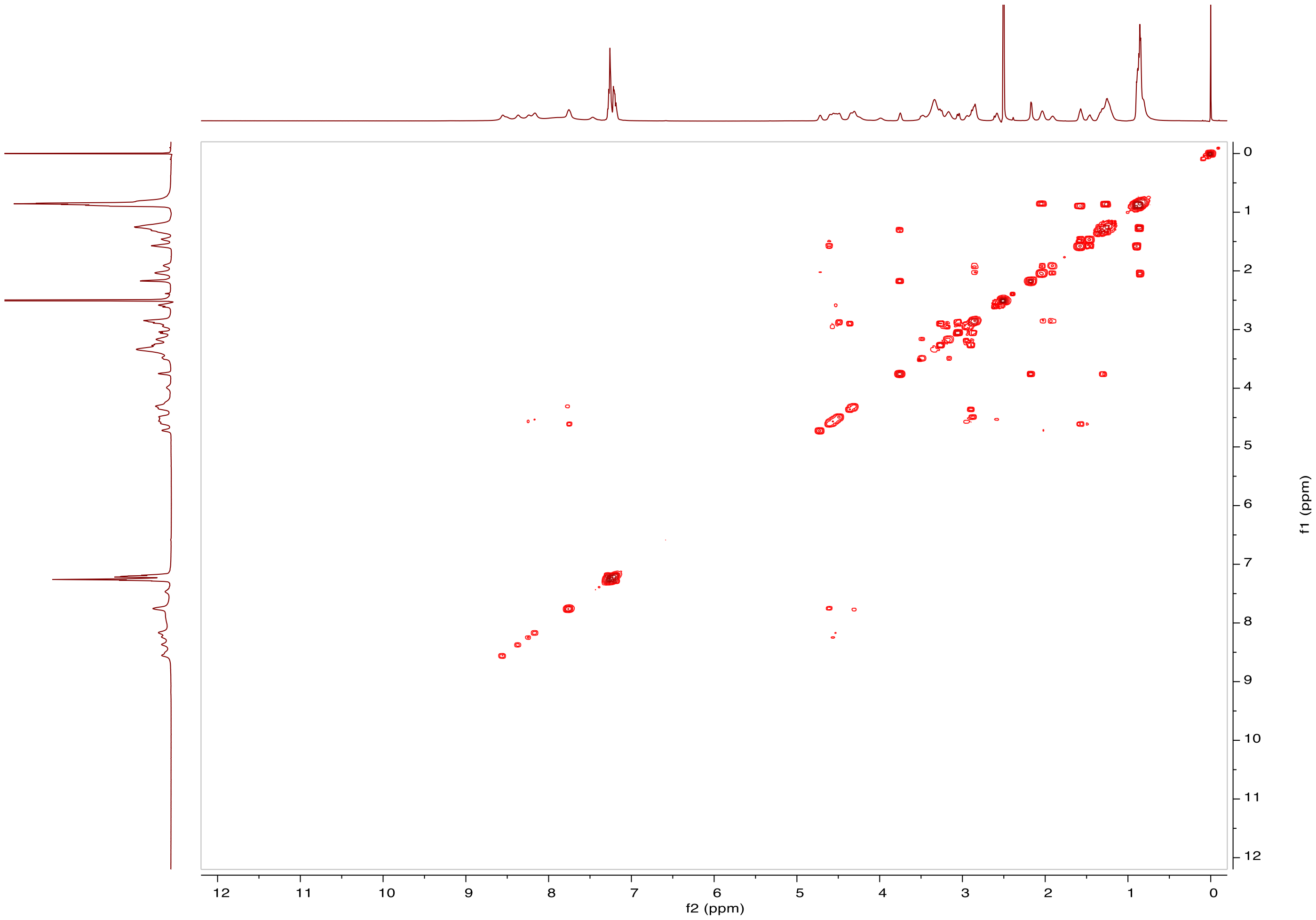

Figure S54. ^1H-^1H COSY NMR spectrum of synAQU3-C1 in DMSO-d_6 (600 MHz)

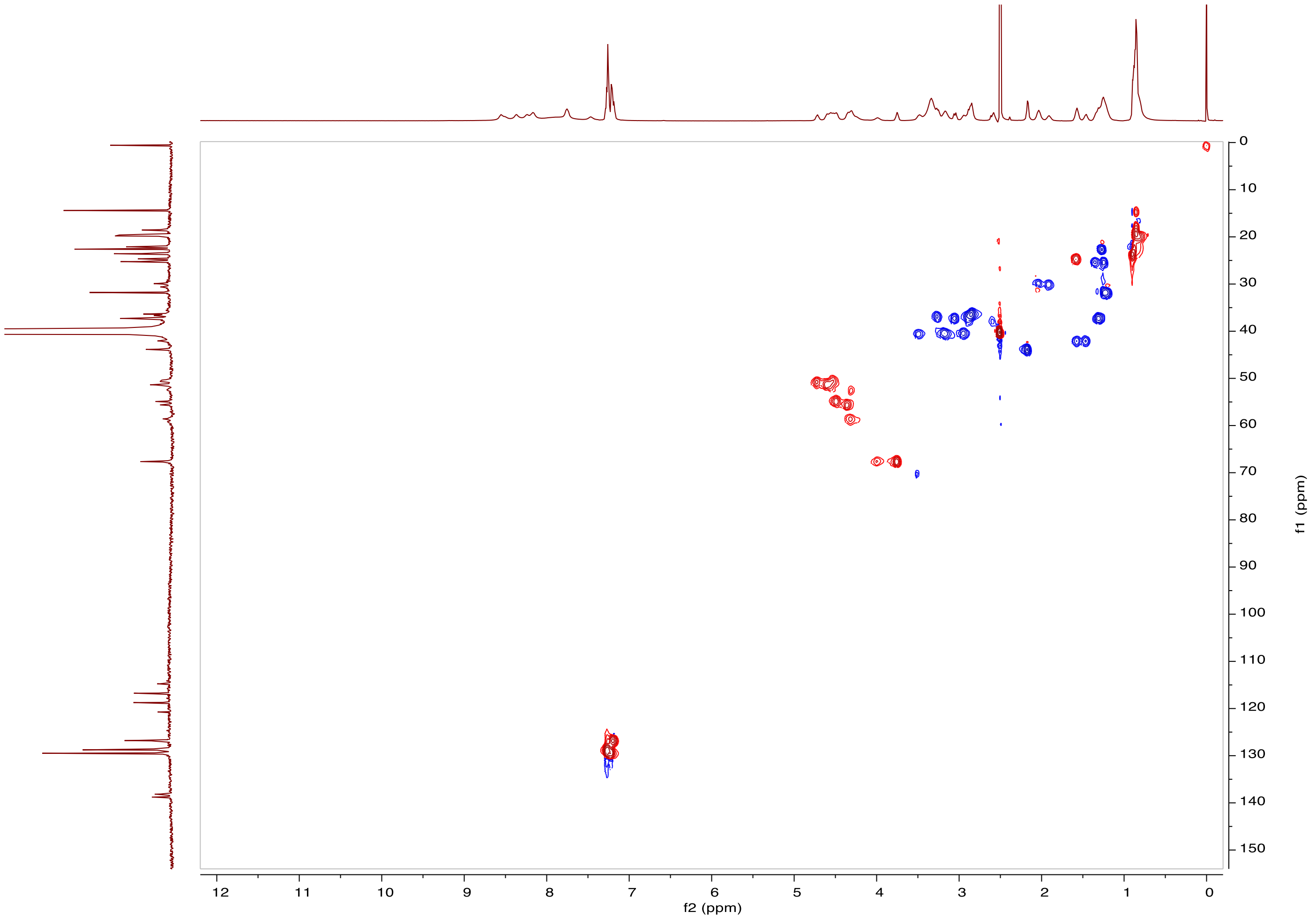

Figure S55. ^{1}H-^{13}C HSQC NMR spectrum of synAQU3-C1 in DMSO-d_6 (600 MHz)

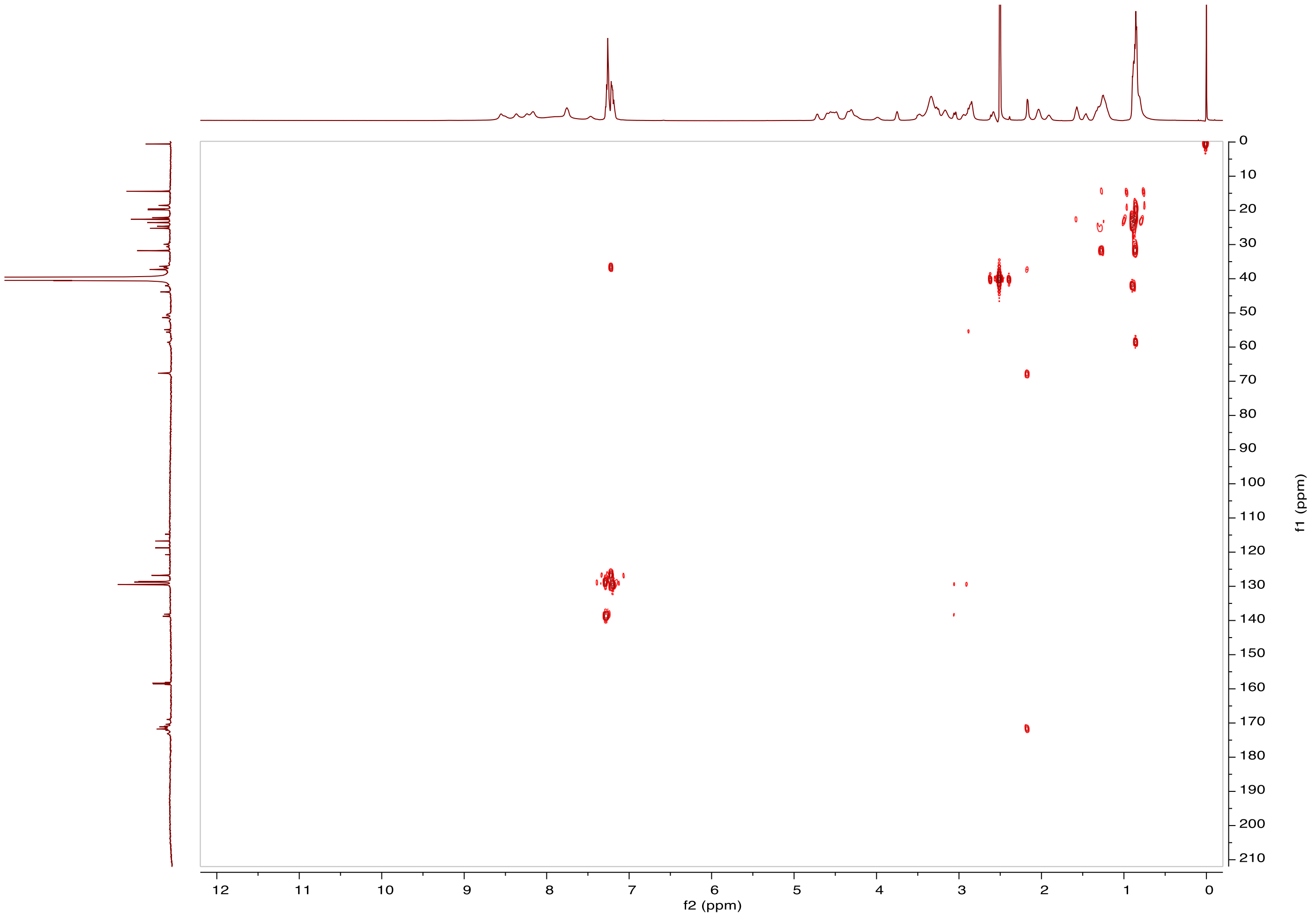

Figure S56. ^{1}H-^{13}C HMBC NMR spectrum of synAQU3-C1 in DMSO-d_6 (600 MHz)

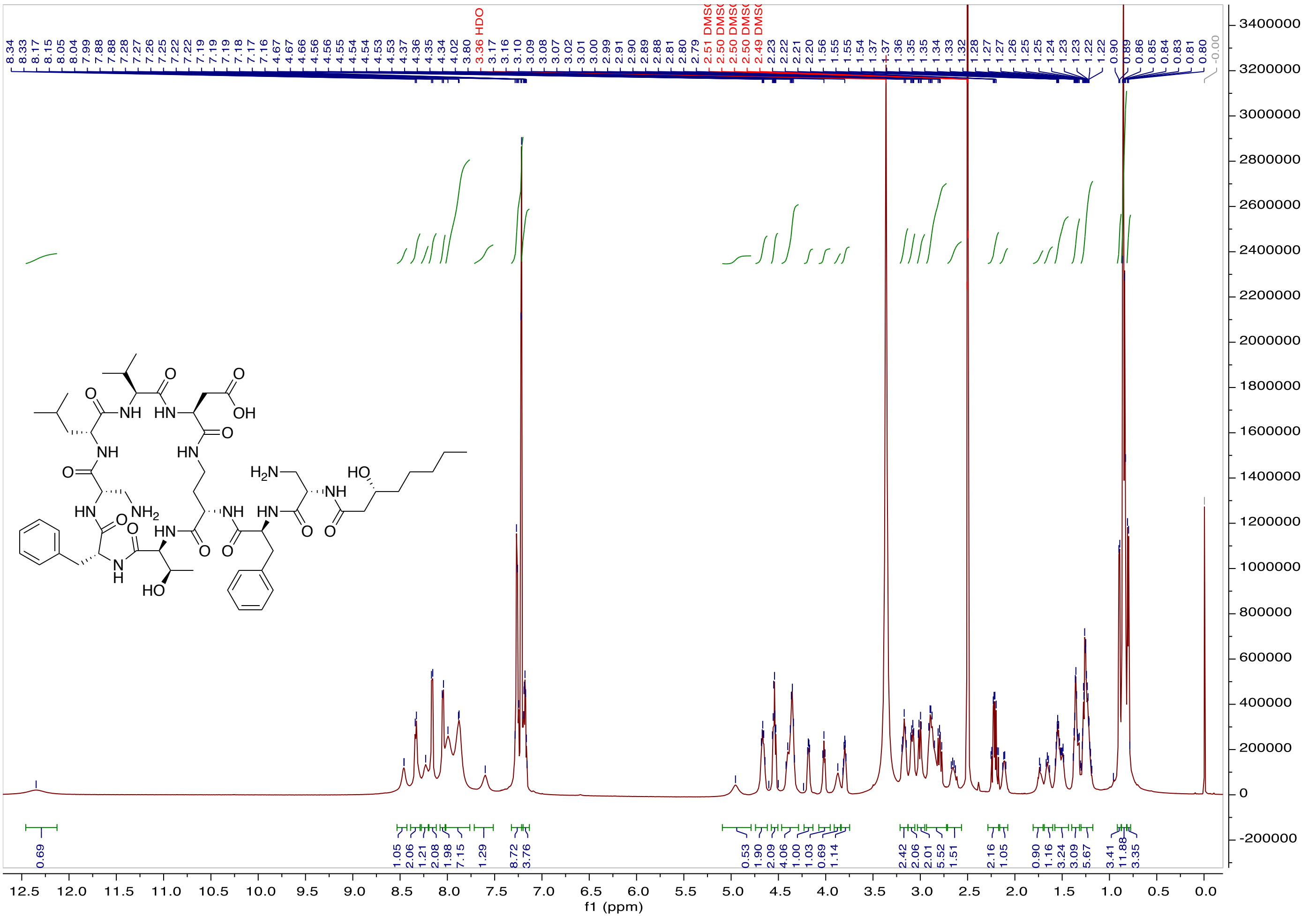

Figure S57. ^{1}H NMR spectrum of synAQU3-C3 in DMSO-d_6 (600 MHz)

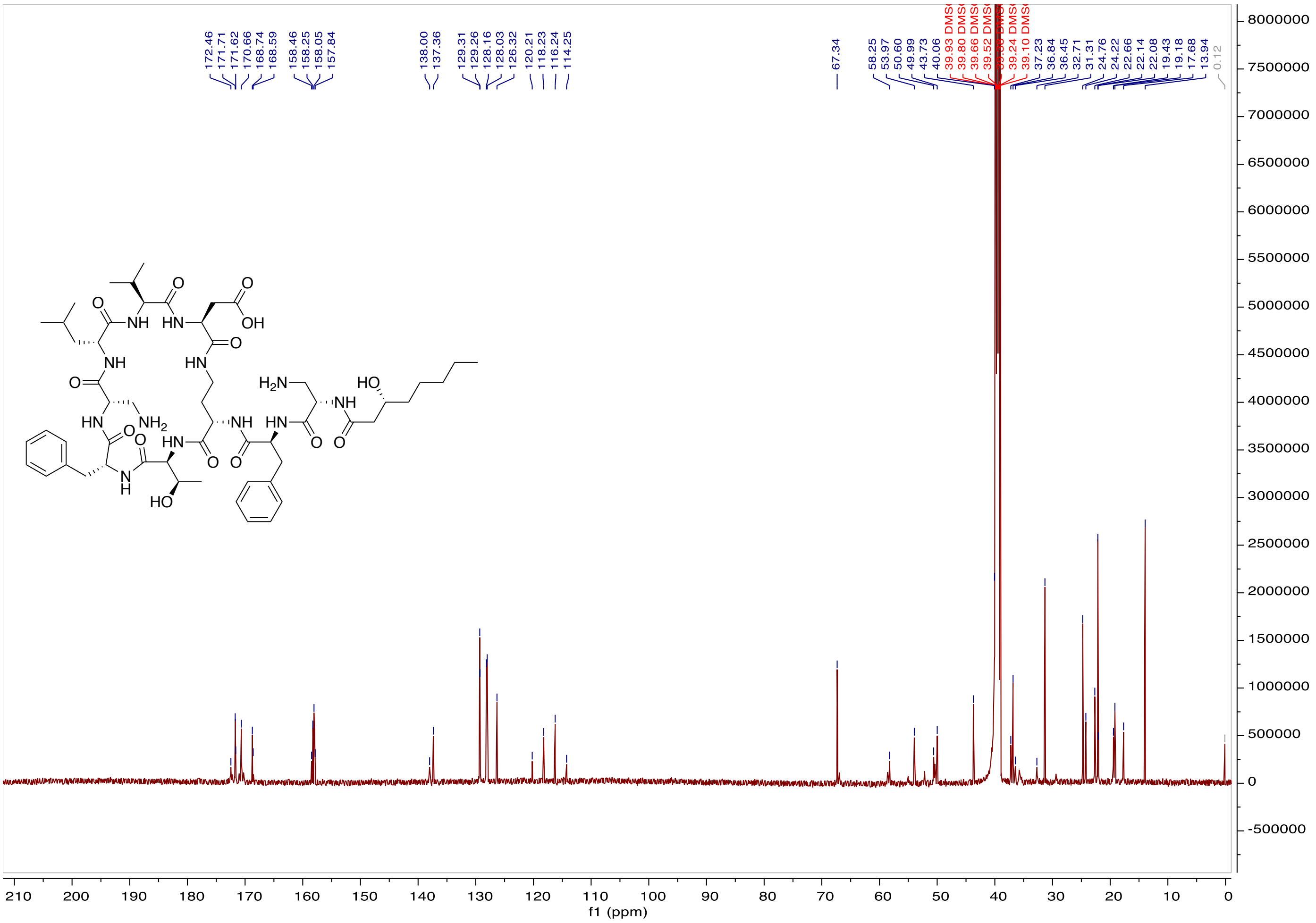

Figure S58. ^{13}C NMR spectrum of synAQU3-C3 in DMSO-d_6 (150 MHz)

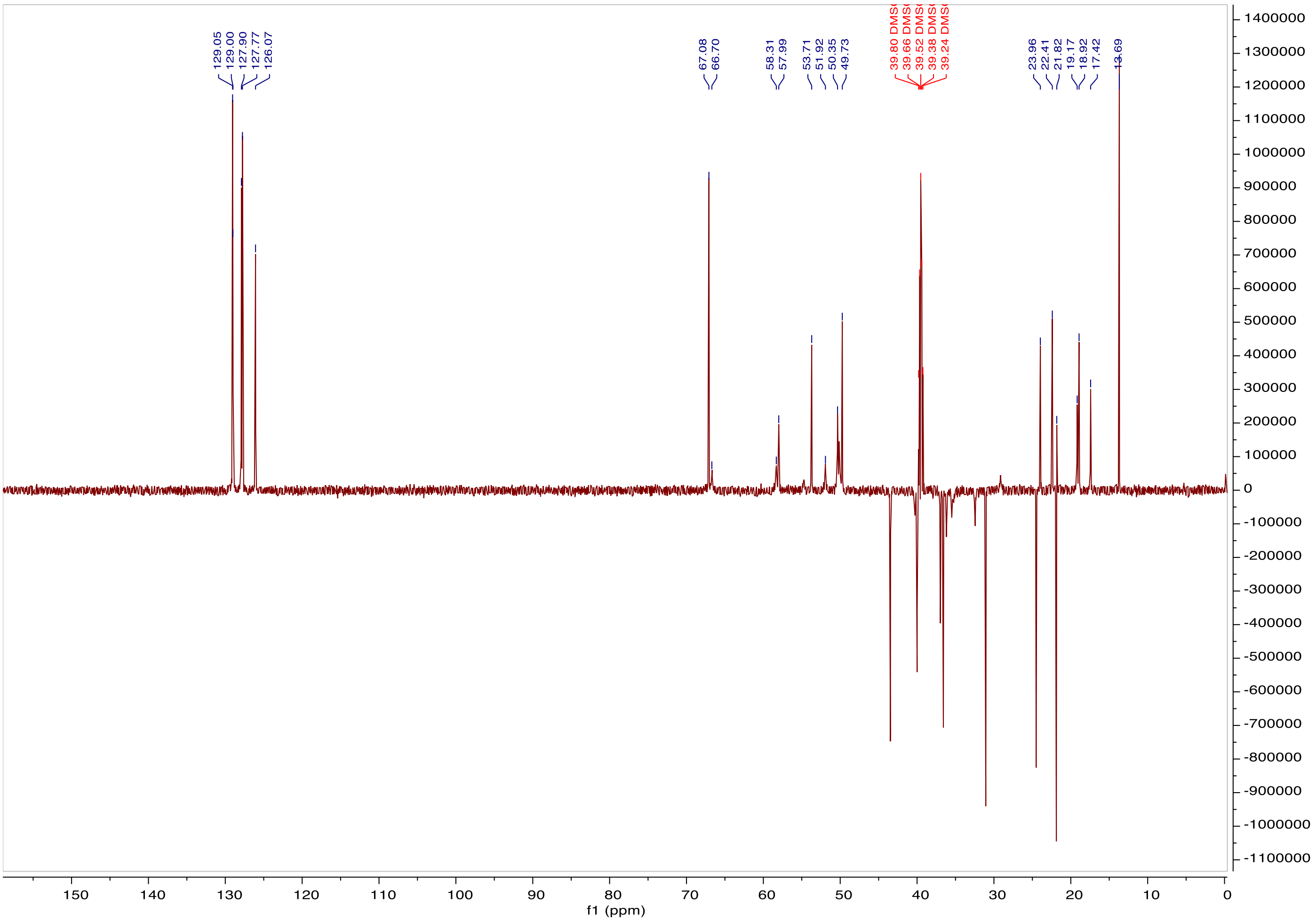

Figure S59. DEPT135 NMR spectrum of synAQU3-C3 in DMSO-d_6 (150 MHz)

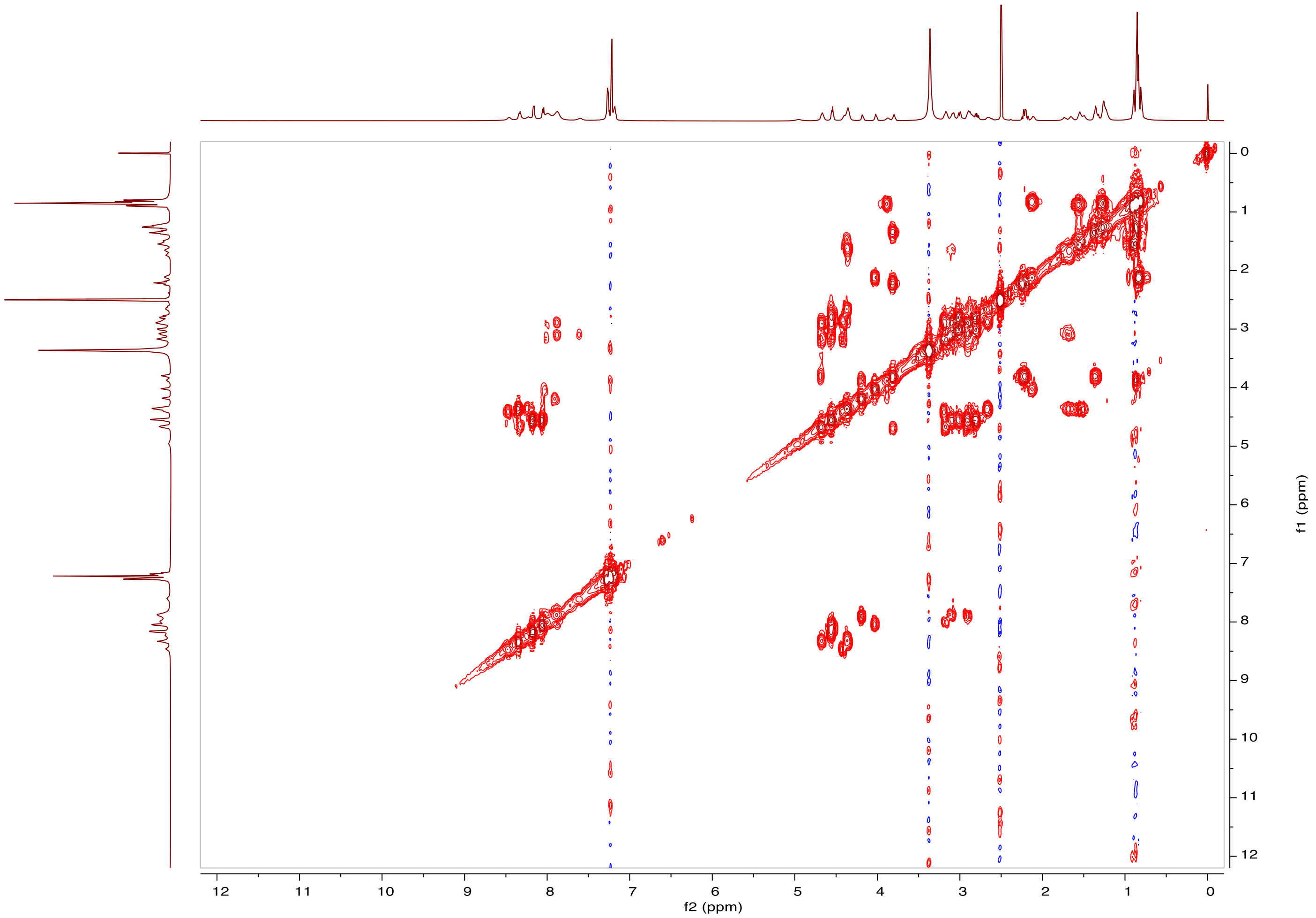

Figure S60. ^{1}H-^{1}H COSY NMR spectrum of synAQU3-C3 in DMSO-d_6 (600 MHz)

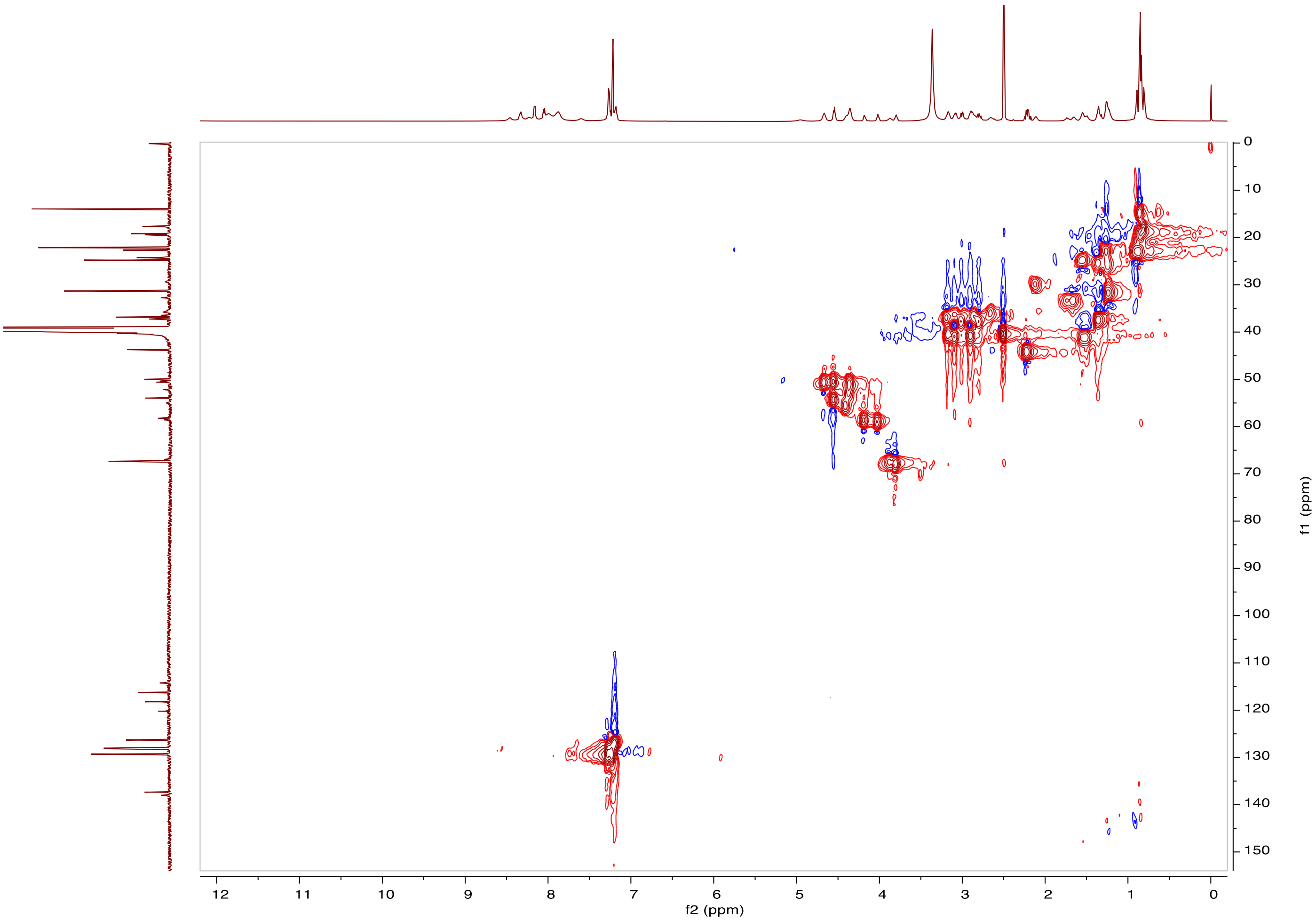

Figure S61. ^{1}H-^{13}C HSQC NMR spectrum of synAQU3-C3 in DMSO-d_6 (600 MHz)

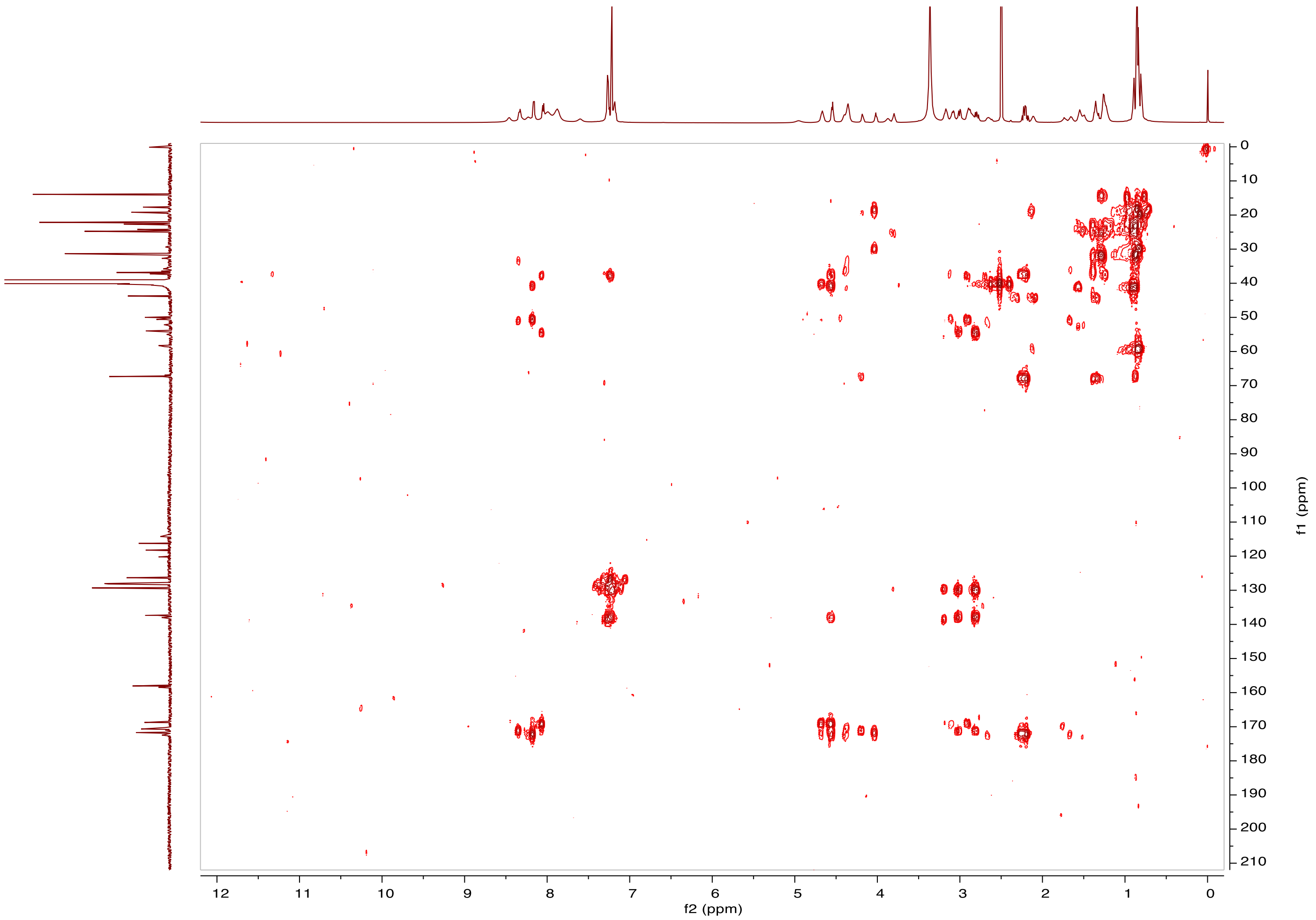

Figure S62. ^{1}H-^{13}C HMBC NMR spectrum of synAQU3-C3 in DMSO-d_6 (600 MHz)

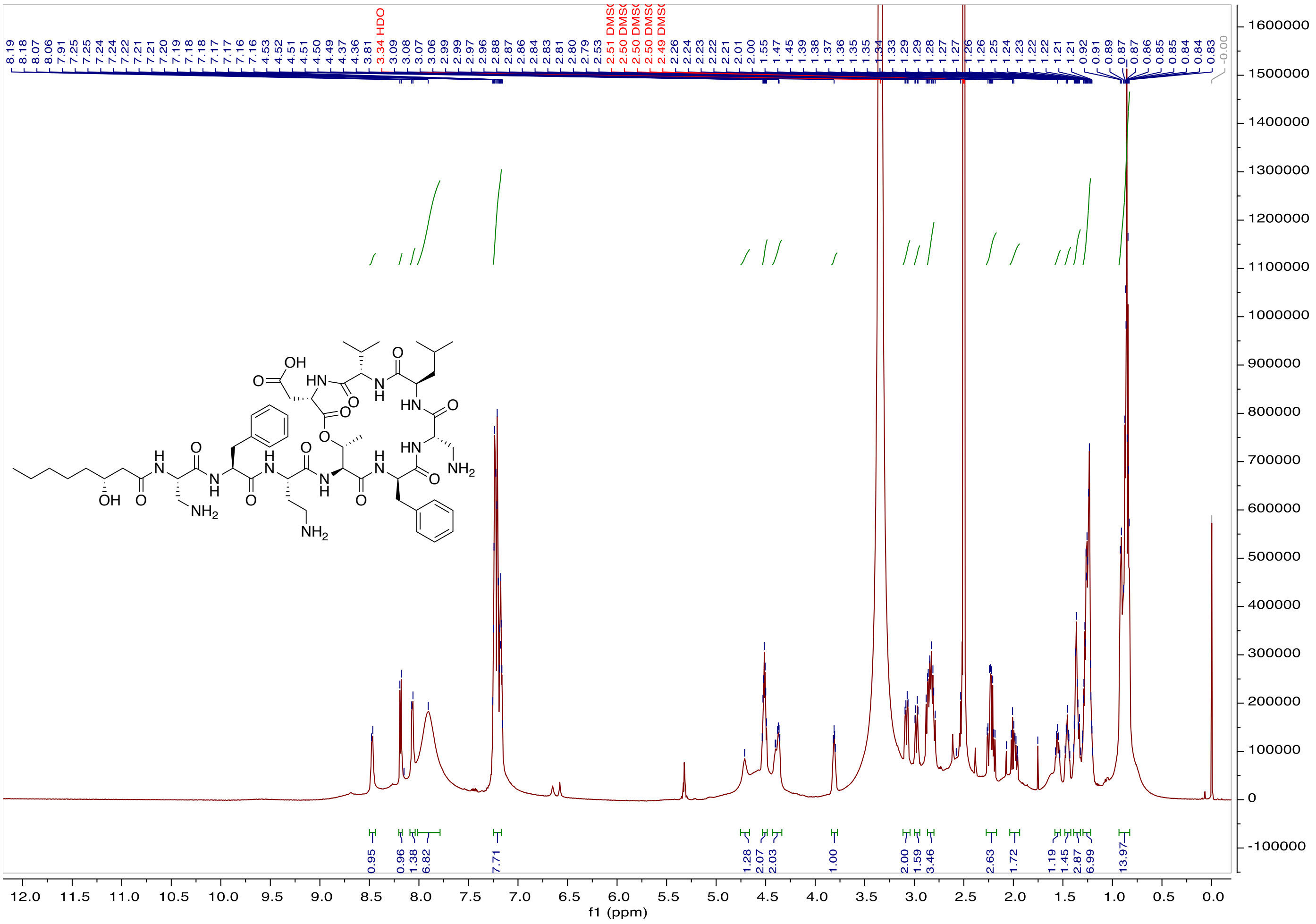

Figure S63. ^{1}H NMR spectrum of synAQU3-C4 in DMSO-d_6 (600 MHz)

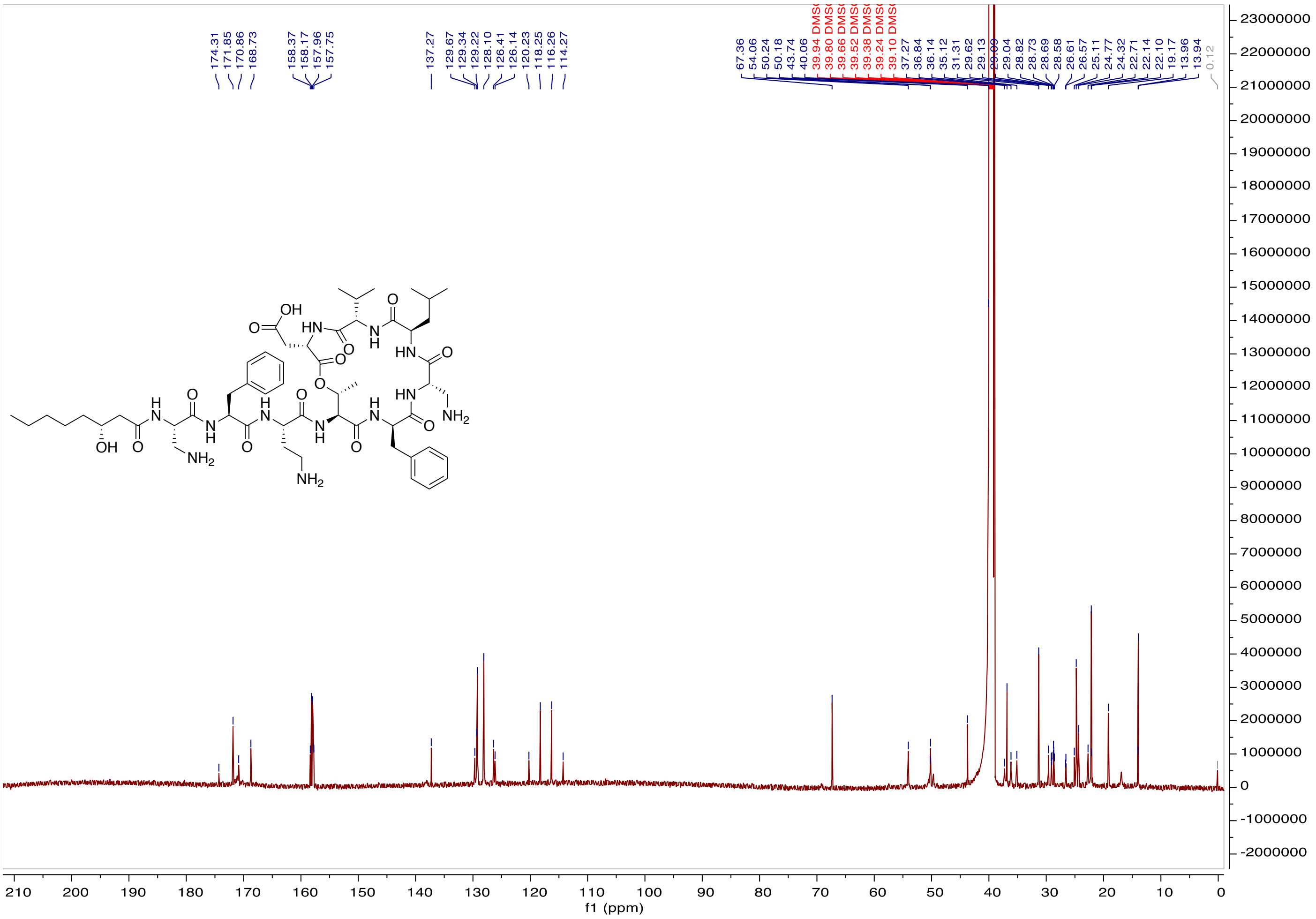

Figure S64. ^{13}C NMR spectrum of synAQU3-C4 in DMSO-d_6 (150 MHz)

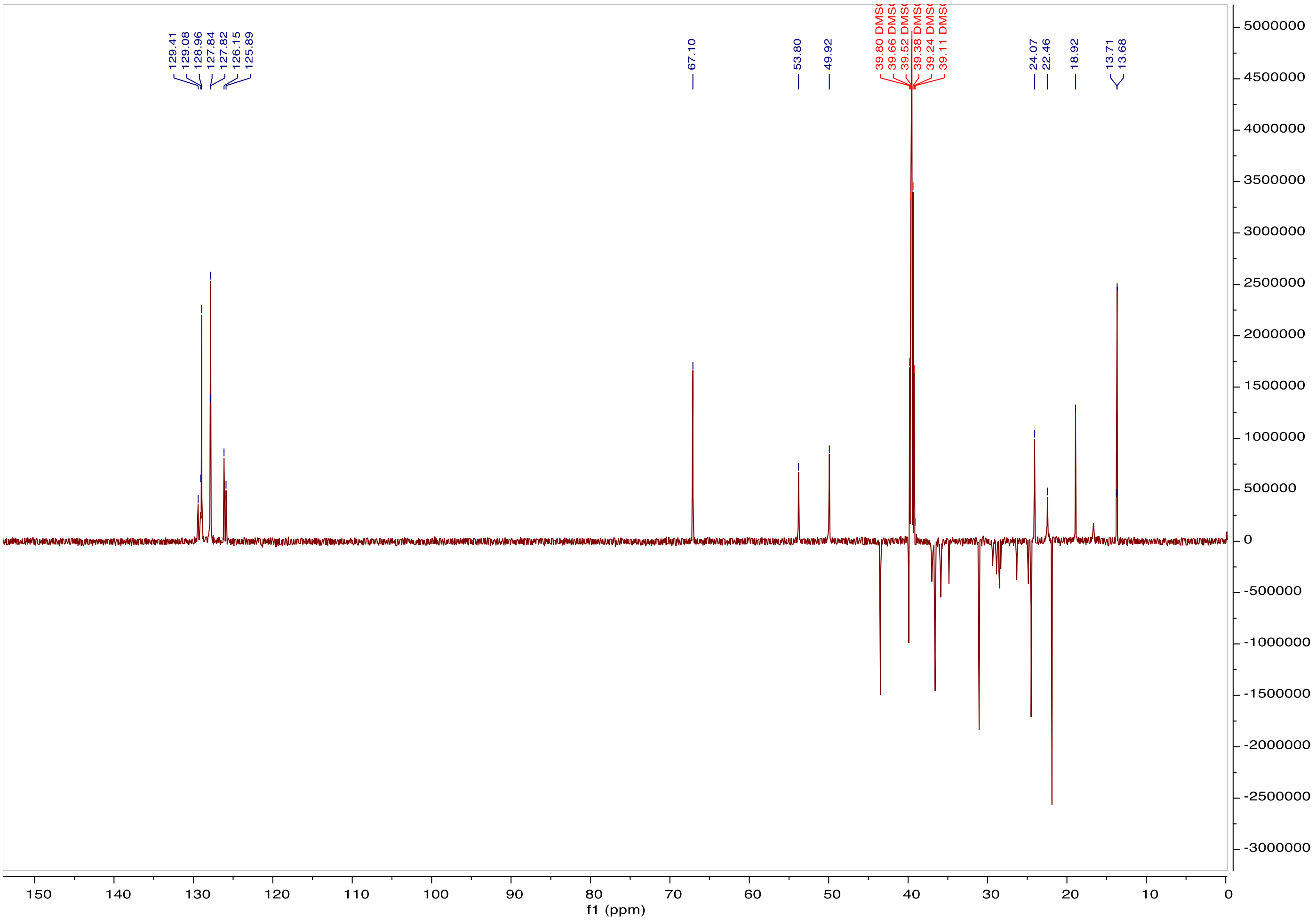

Figure S65. DEPT135 NMR spectrum of synAQU3-C4 in DMSO-d_6 (150 MHz)

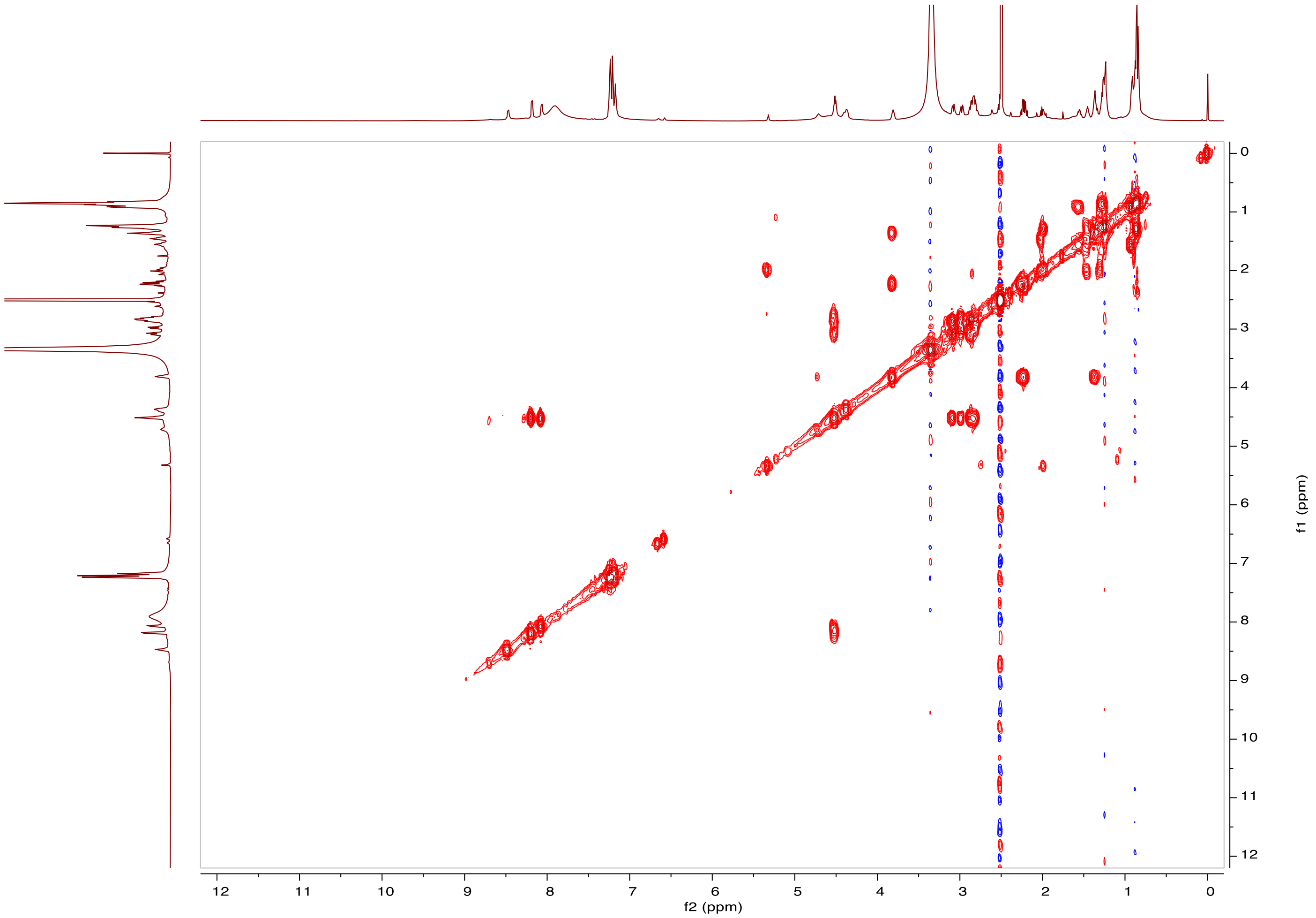

Figure S66. ^{1}H-^{1}H COSY NMR spectrum of synAQU3-C4 in DMSO-d_6 (600 MHz)

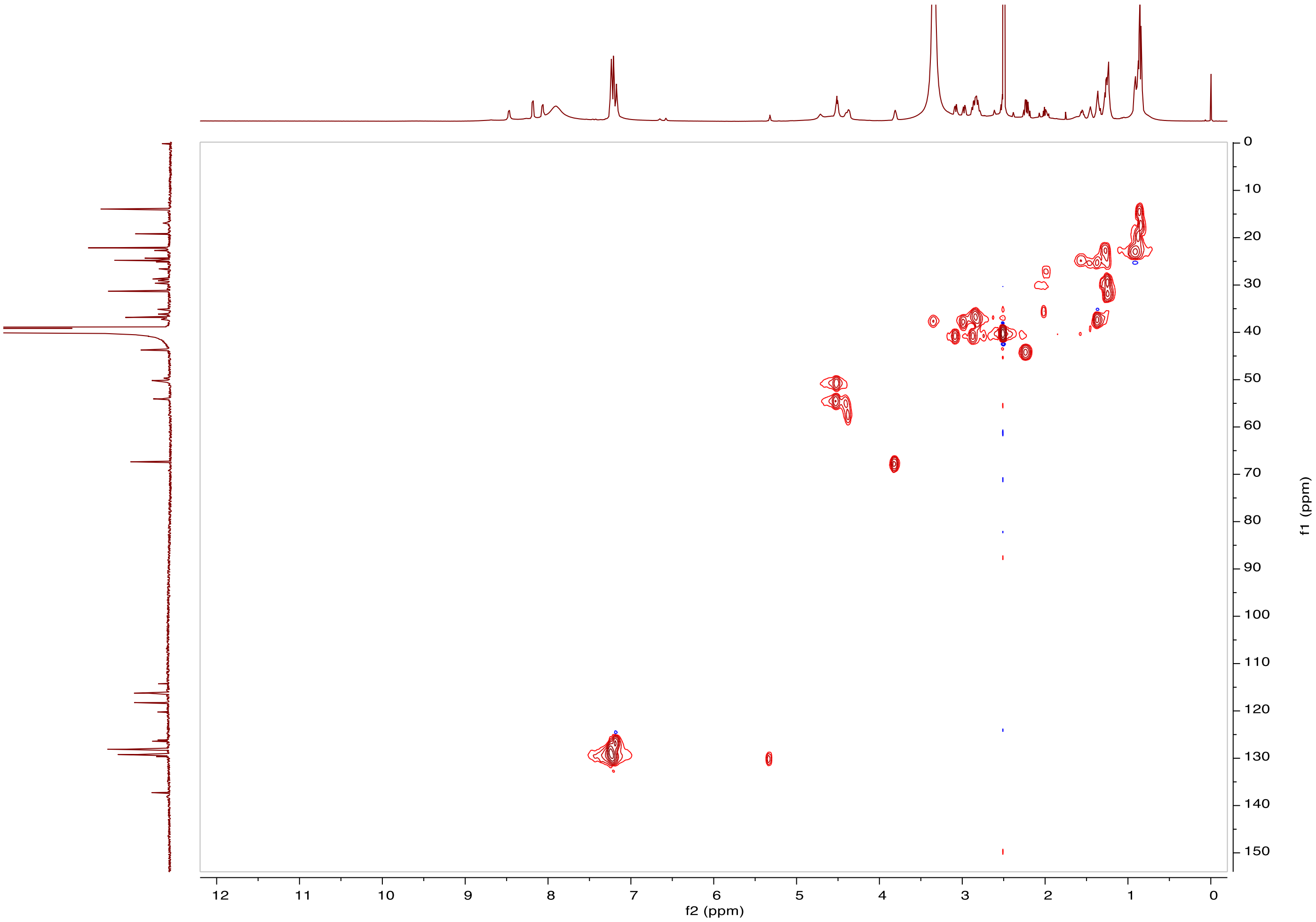

Figure S67. ^{1}H-^{13}C HSQC NMR spectrum of synAQU3-C4 in DMSO-d_6 (600 MHz)

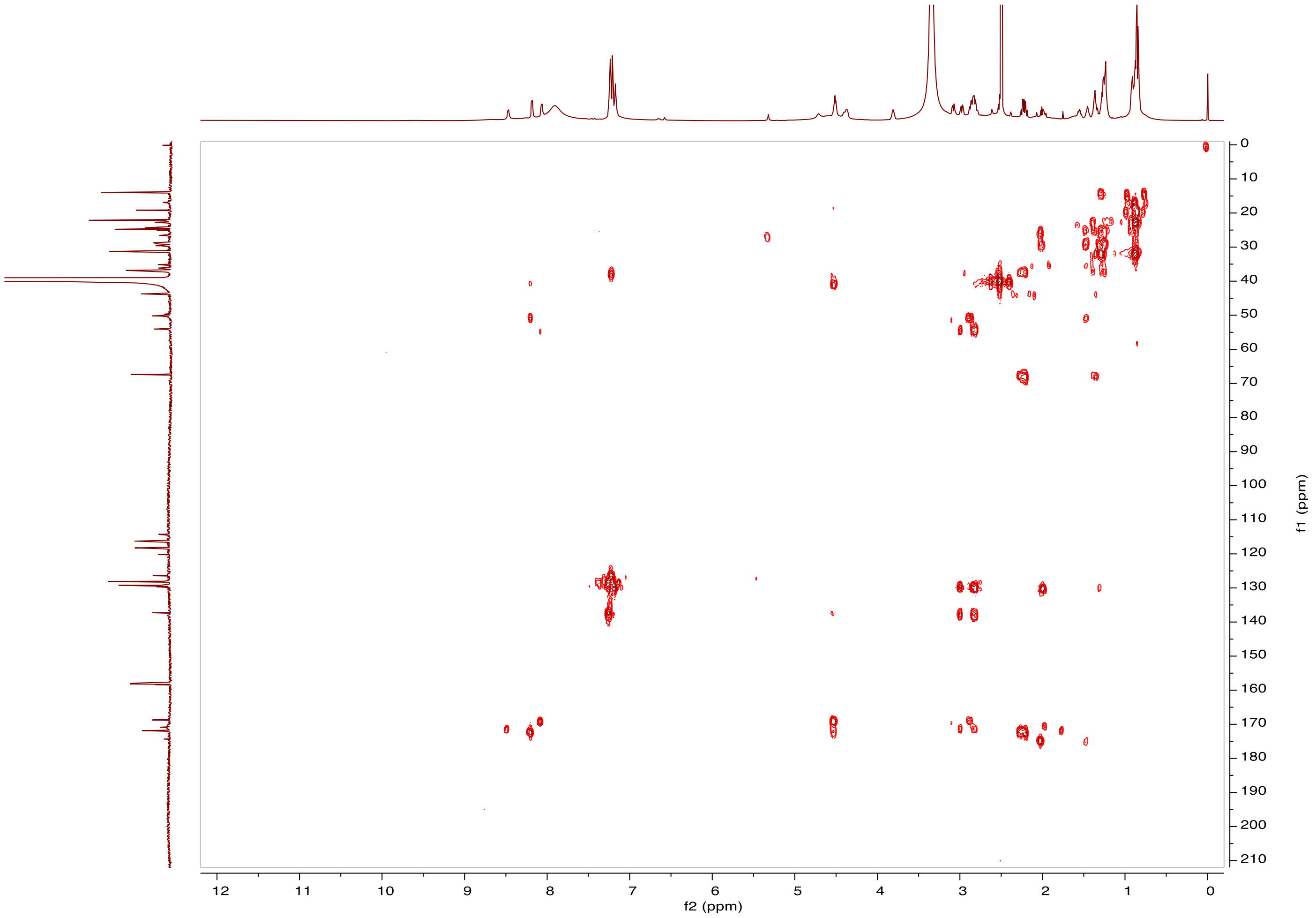

Figure S68. ^{1}H-^{13}C HMBC NMR spectrum of synAQU3-C4 in DMSO-d_6 (600 MHz)

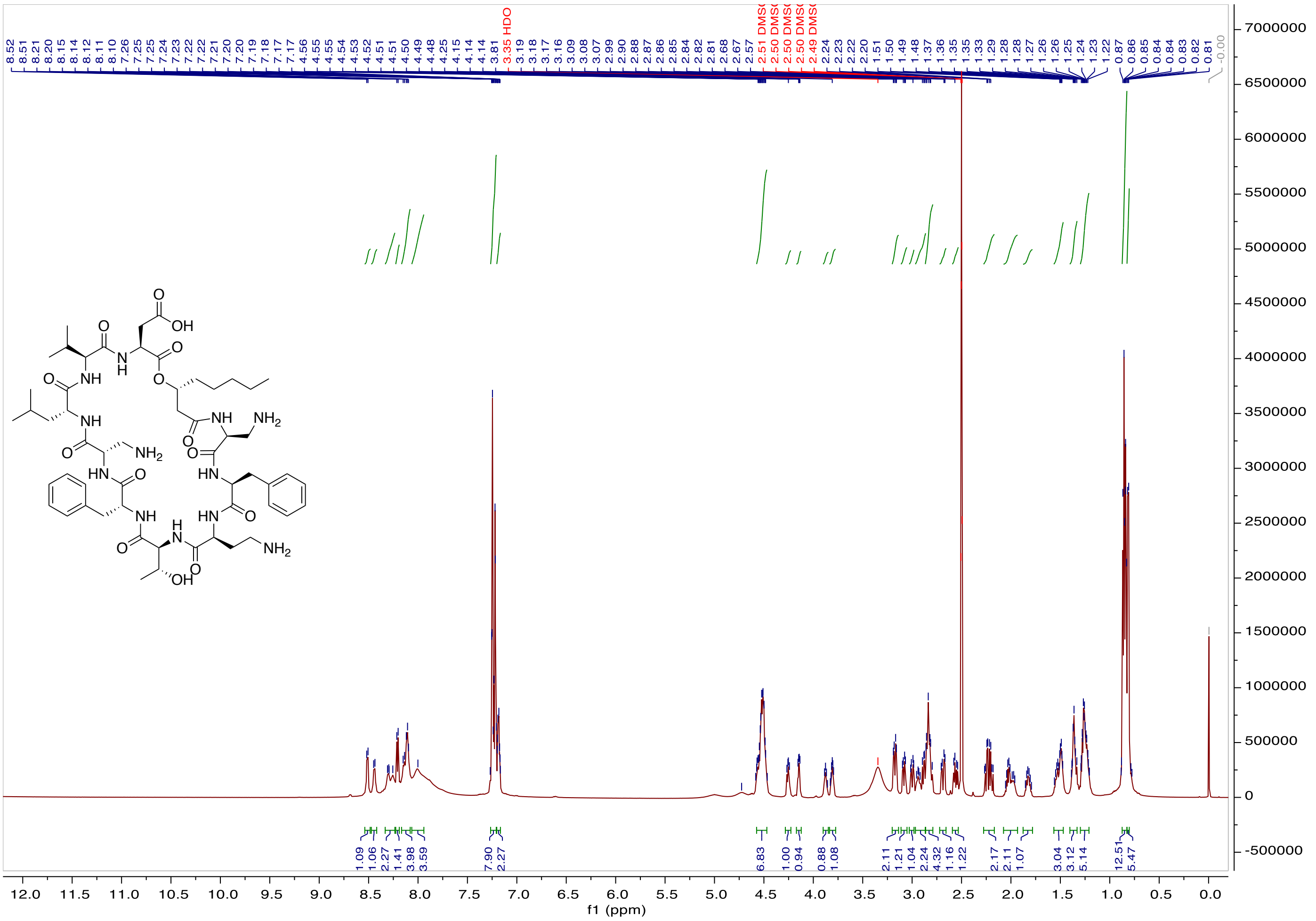

Figure S69. ^{1}H NMR spectrum of synAQU3-cFA in DMSO-d_6 (600 MHz)

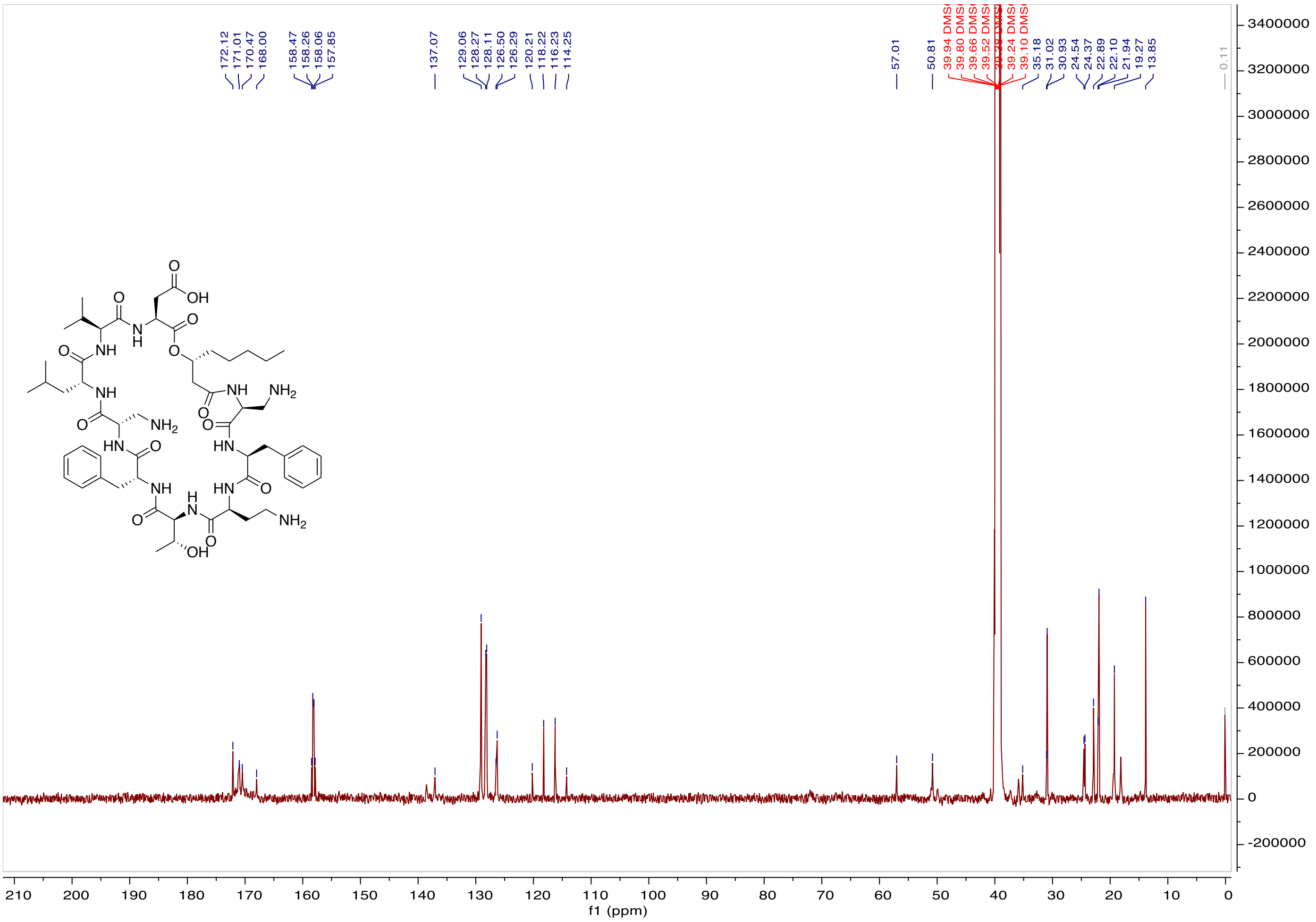

Figure S70. ^{13}C NMR spectrum of synAQU3-cFA in DMSO-d_6 (150 MHz)

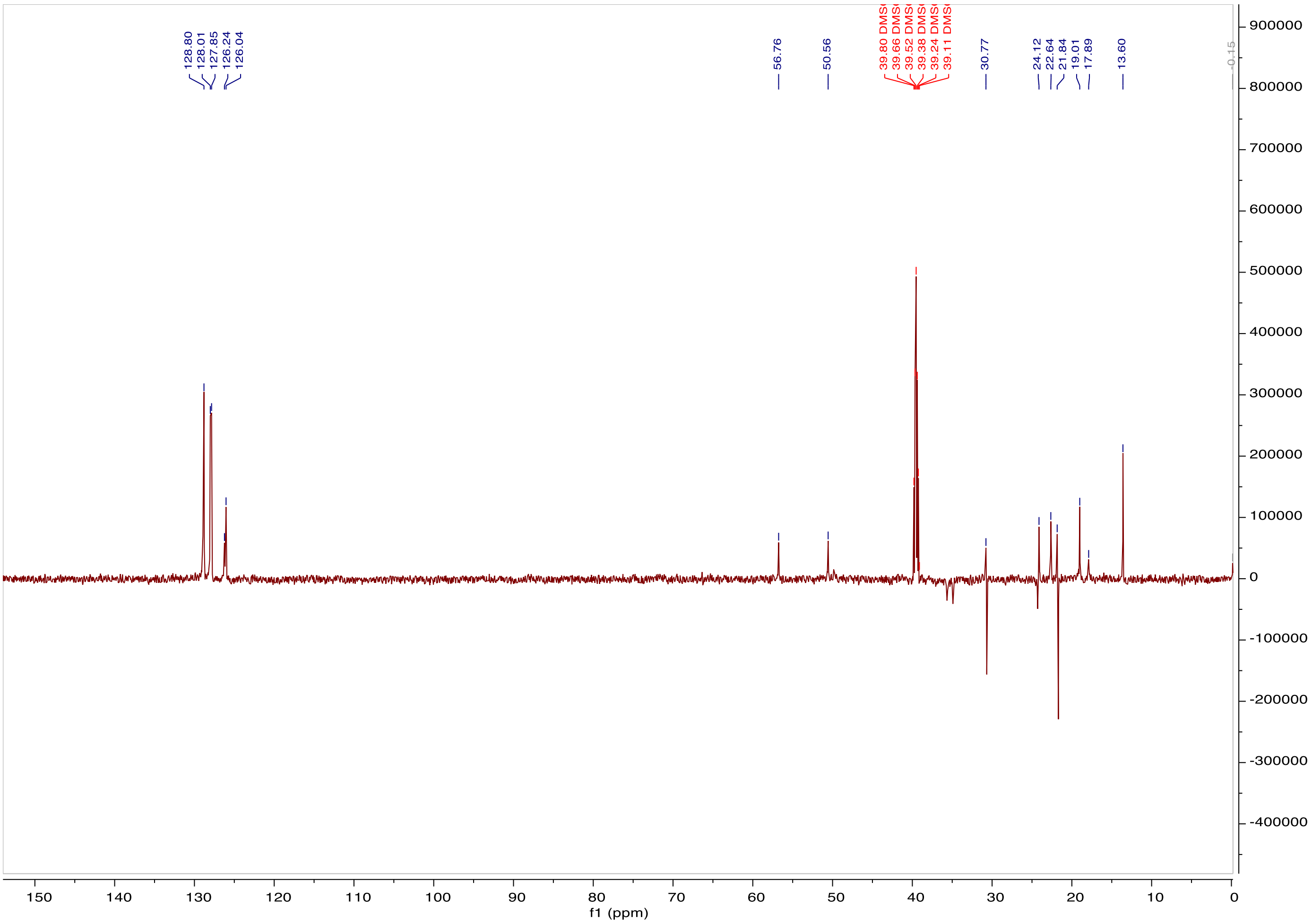

Figure S71. DEPT135 NMR spectrum of synAQU3-cFA in DMSO-d_6 (150 MHz)

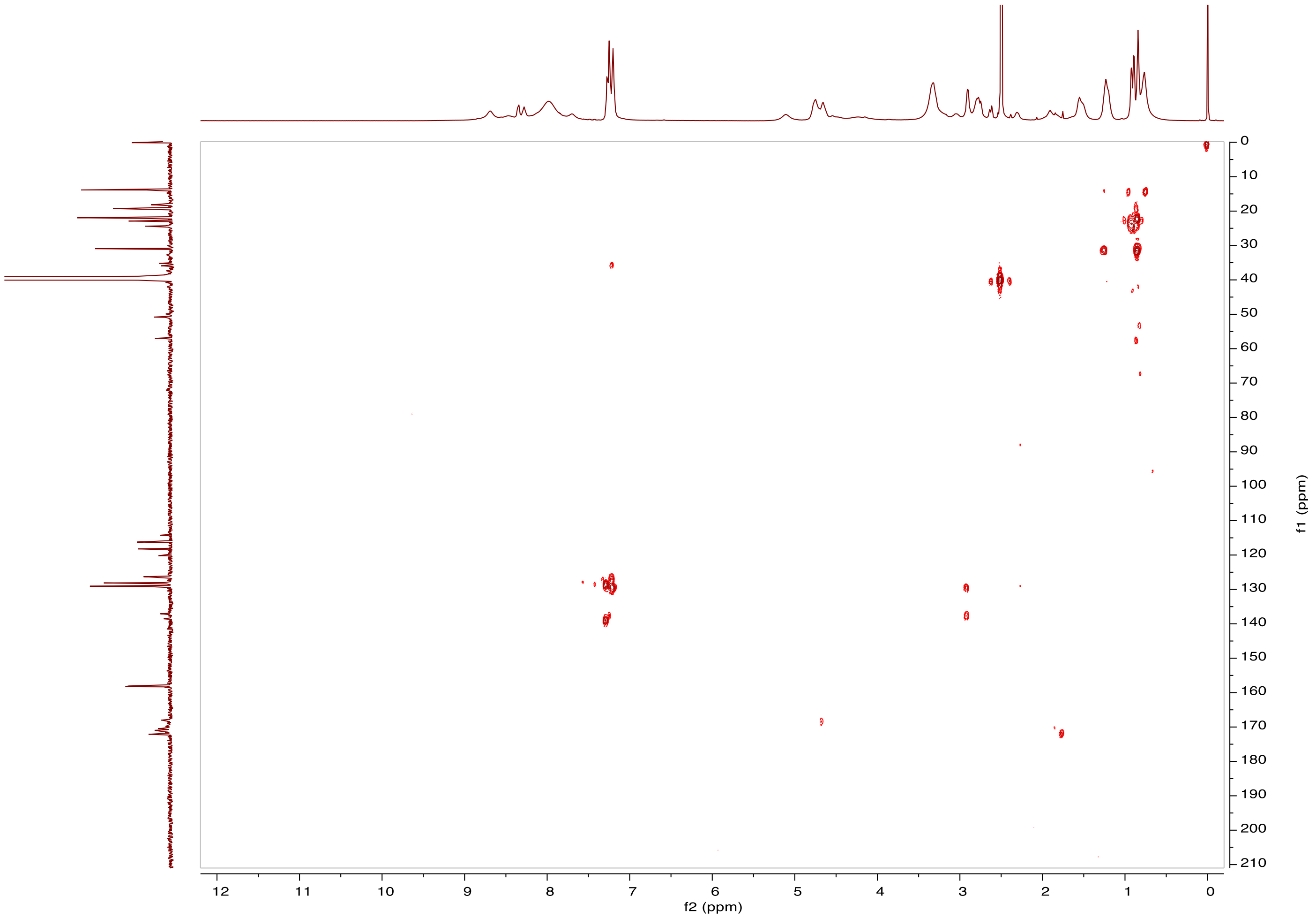

Figure S72. ^1H-^1H COSY NMR spectrum of synAQU3-cFA in DMSO-d_6 (600 MHz)

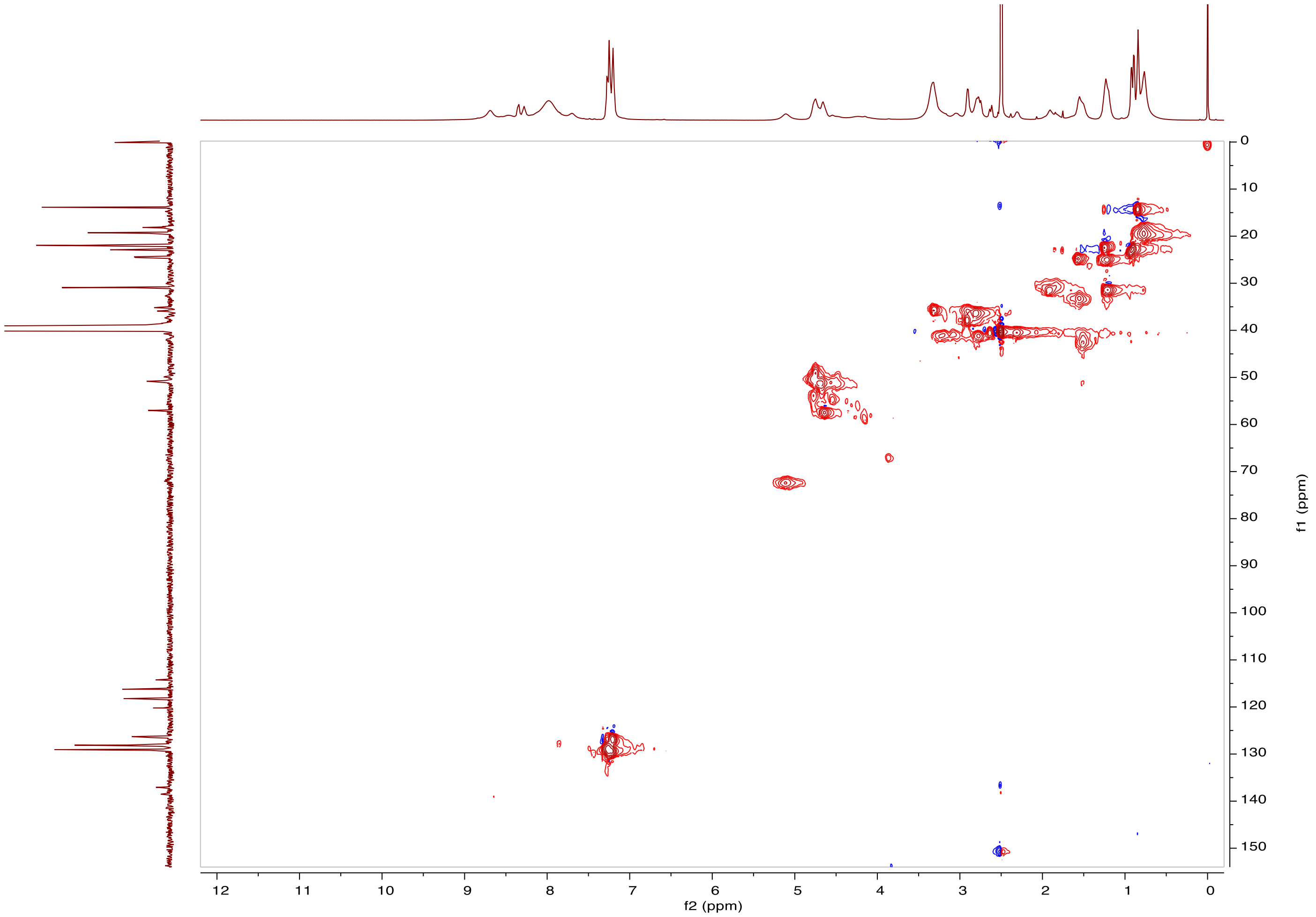

Figure S73. ^{1}H-^{13}C HSQC NMR spectrum of synAQU3-cFA in DMSO-d_6 (600 MHz)

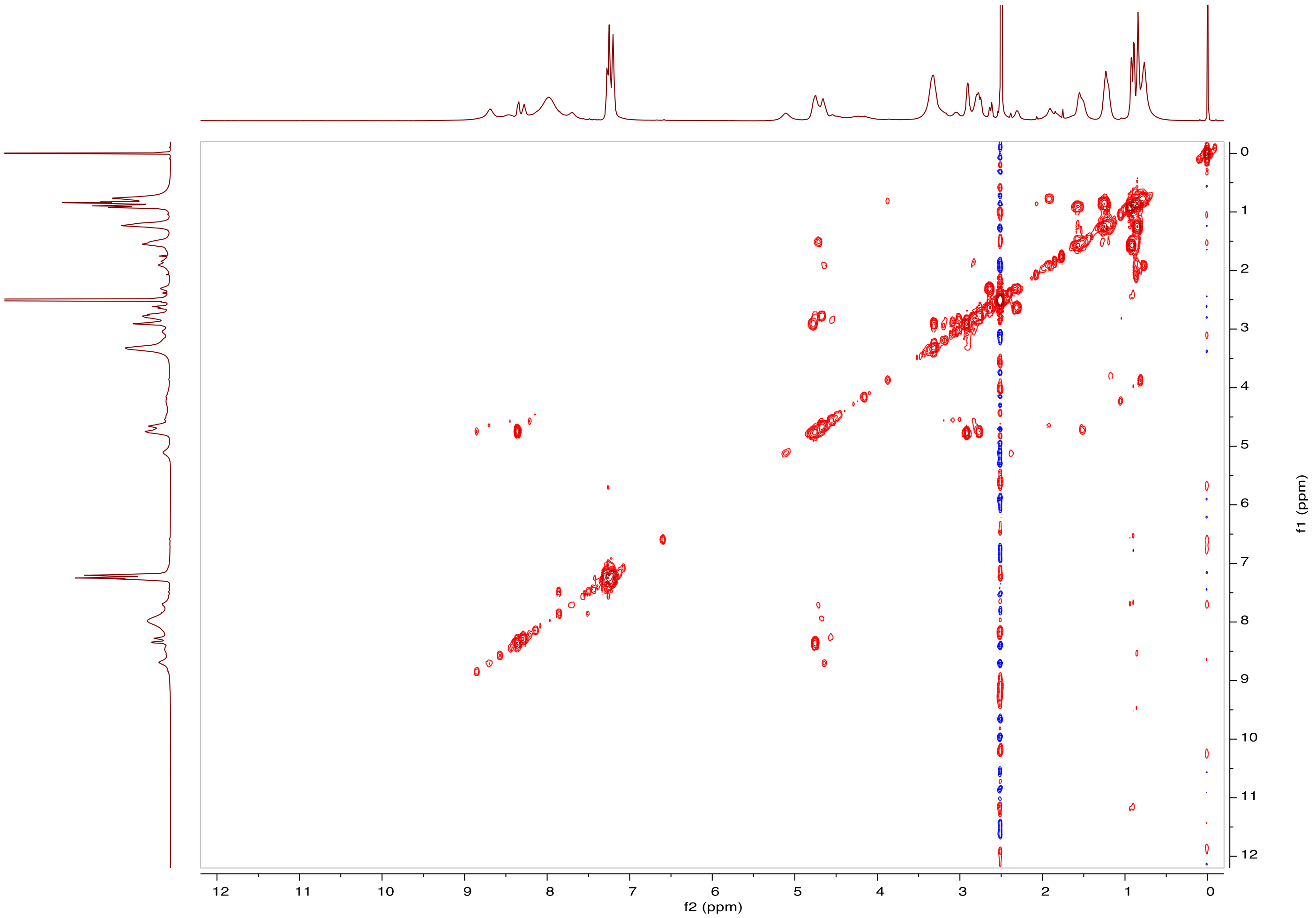

Figure S74. ^{1}H-^{13}C HMBC NMR spectrum of synAQU3-cFA in DMSO-d_6 (600 MHz)

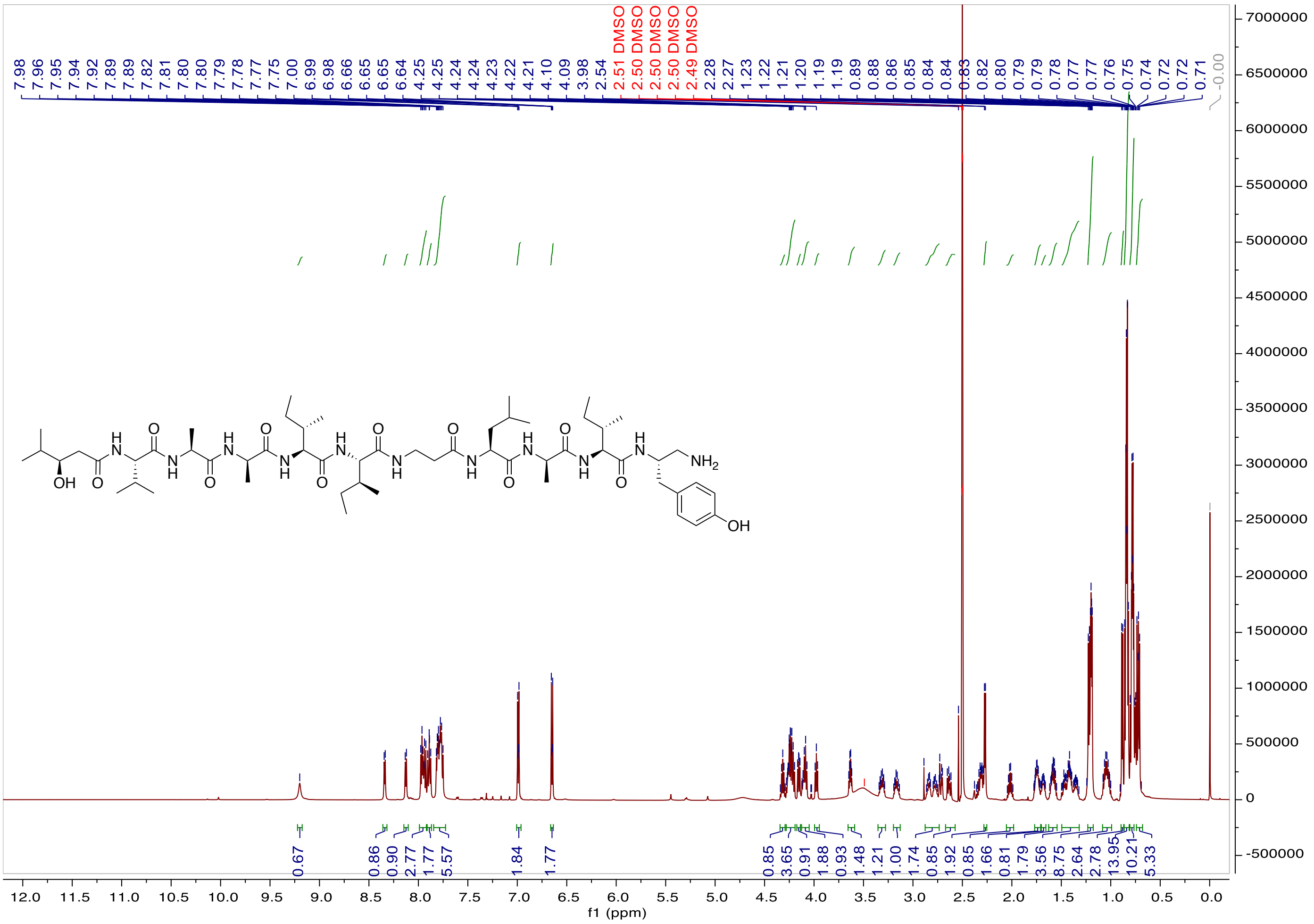

Figure S75. ^{1}H NMR spectrum of synAQU4-L in DMSO-d_6 (600 MHz)

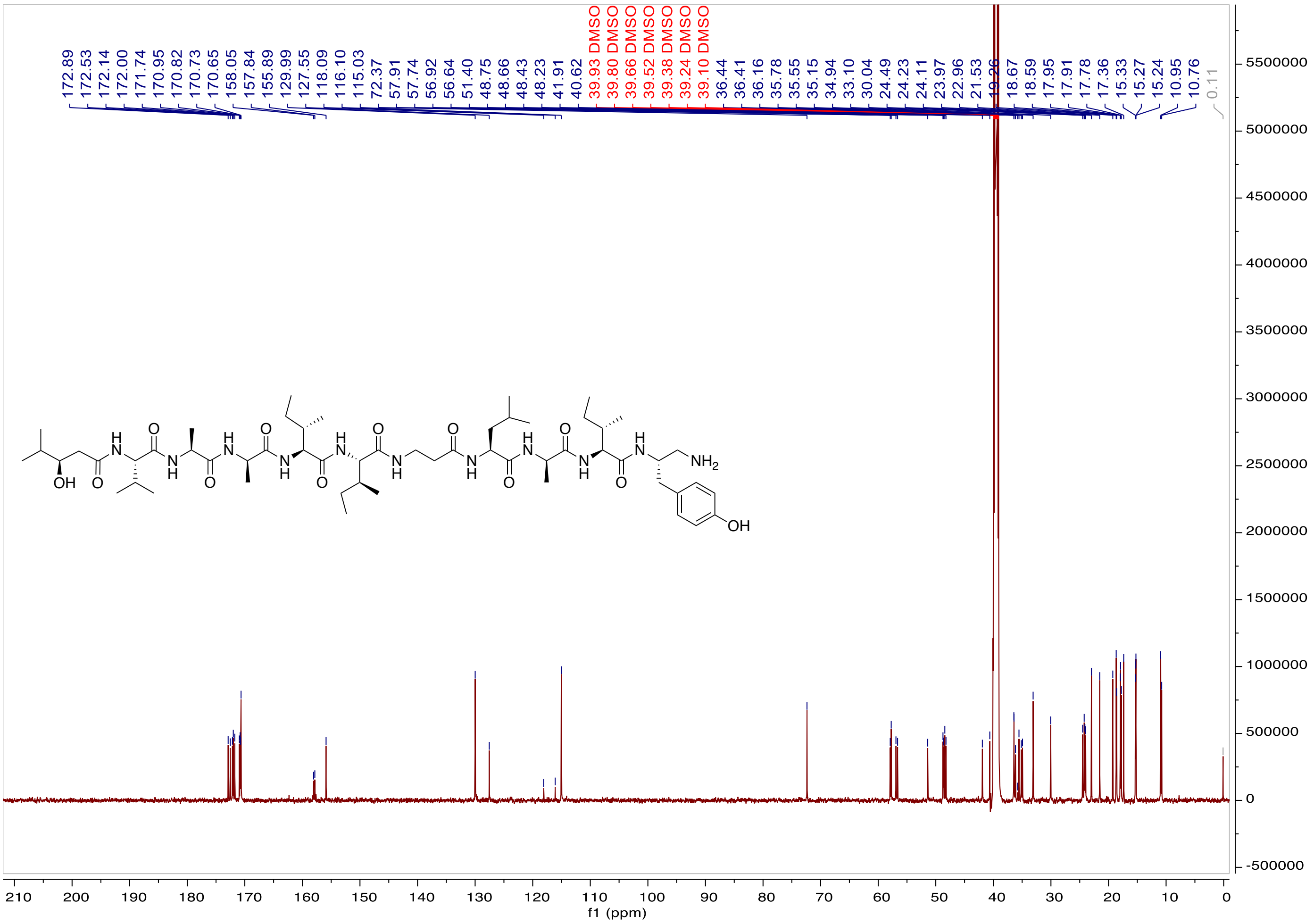

Figure S76. ^{13}C NMR spectrum of synAQU4-L in DMSO-d_6 (150 MHz)

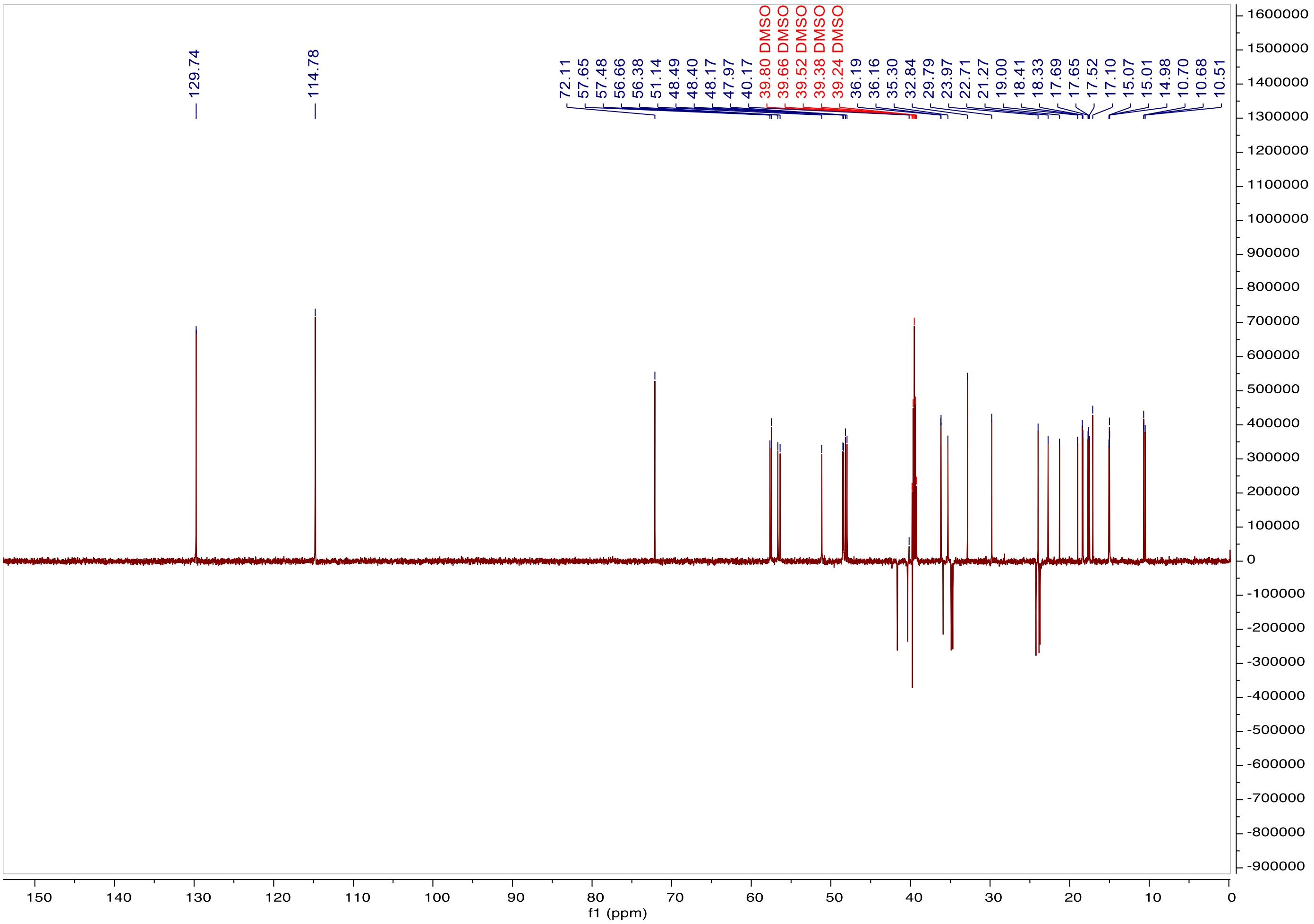

Figure S77. DEPT135 NMR spectrum of synAQU4-L in DMSO-d_6 (150 MHz)

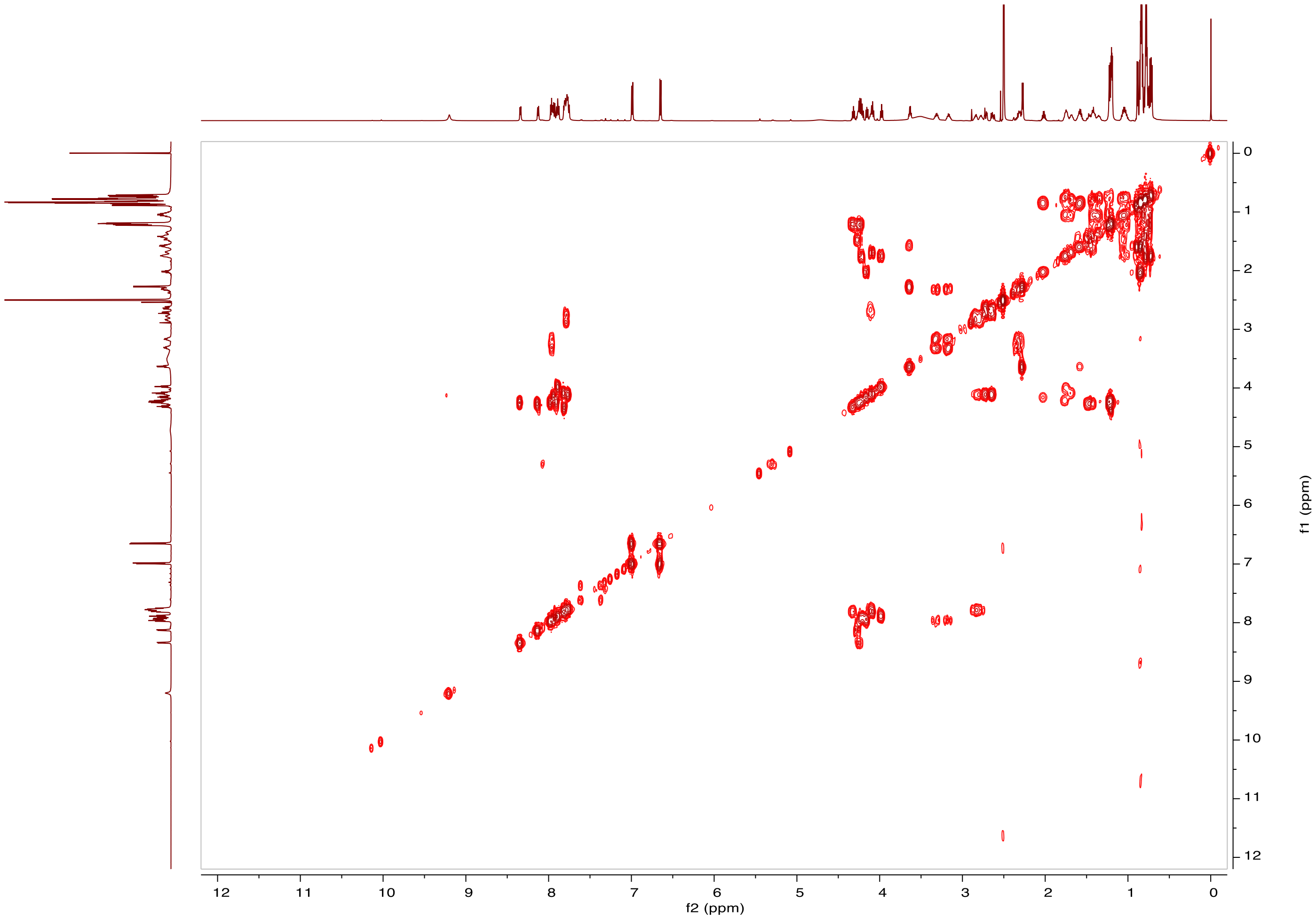

Figure S78. ^{1}H-^{1}H COSY NMR spectrum of synAQU4-L in DMSO-d_6 (600 MHz)

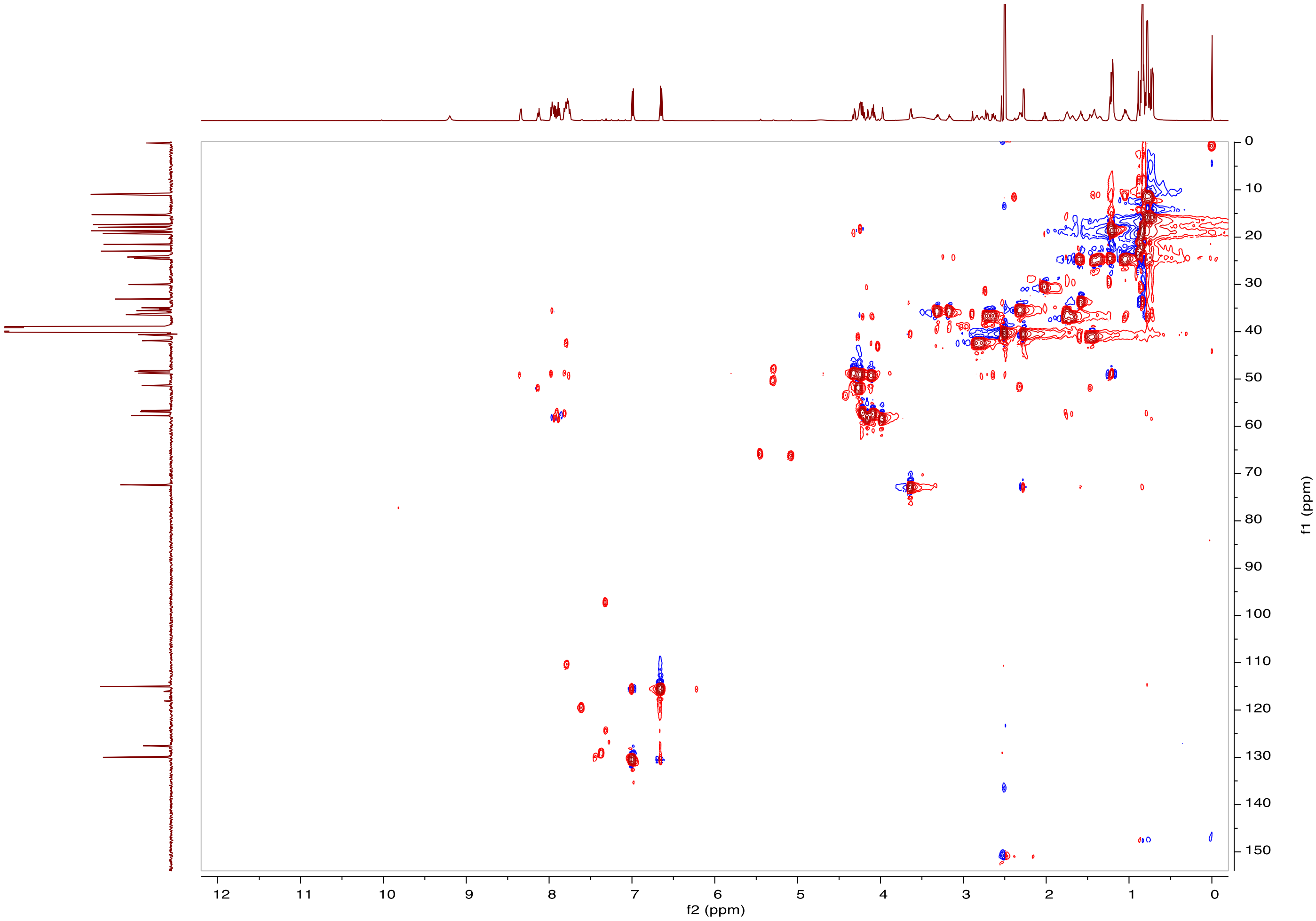

Figure S79. ^{1}H-^{13}C HSQC NMR spectrum of synAQU4-L in DMSO-d_6 (600 MHz)

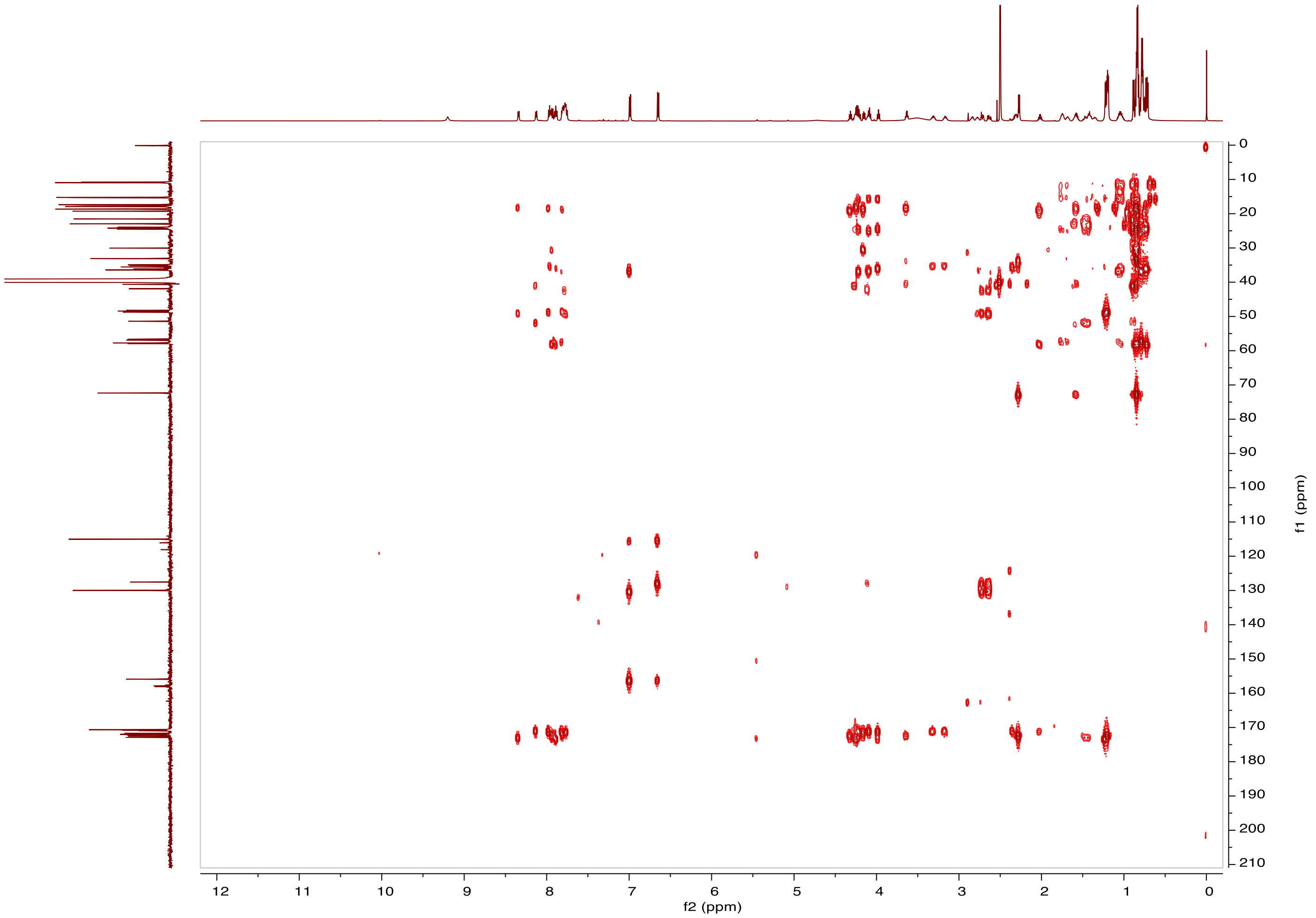

Figure S80. ^{1}H-^{13}C HMBC NMR spectrum of synAQU4-L in DMSO-d_6 (600 MHz)

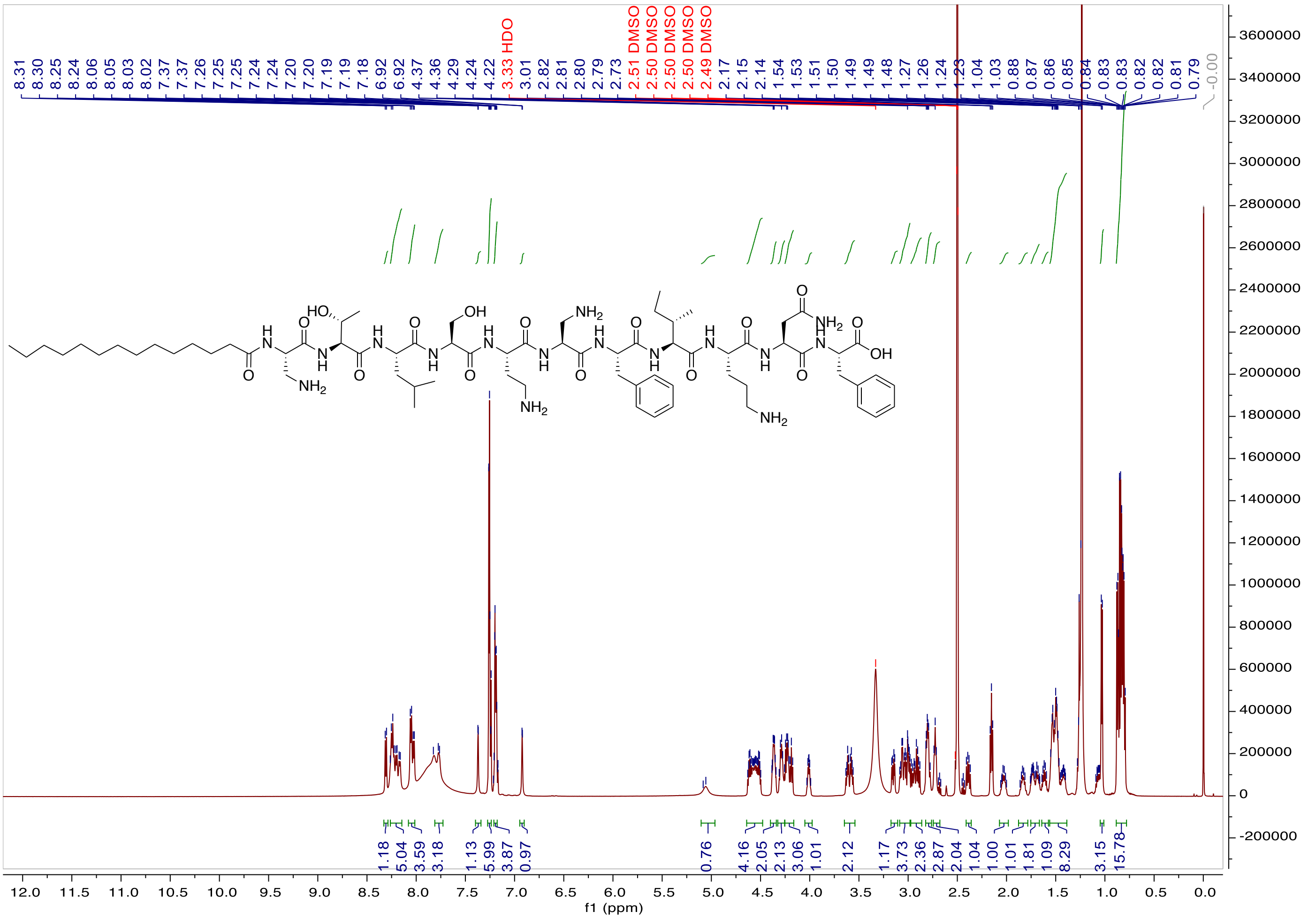

Figure S81. ^{1}H NMR spectrum of aquicidine L (synAQU5-L) in DMSO-d_6 (600 MHz)

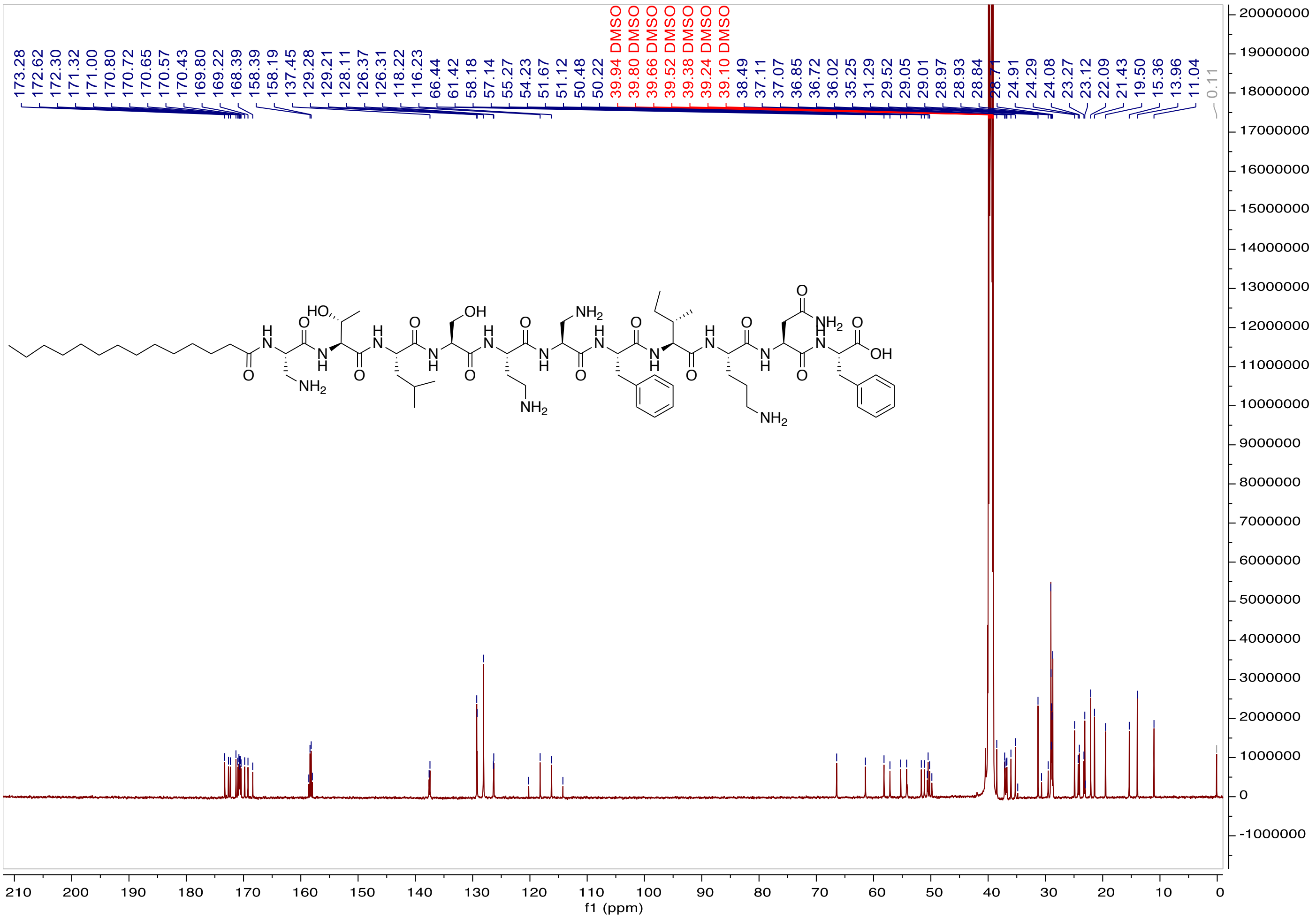

Figure S82. ^{13}C NMR spectrum of aquicidine L (synAQU5-L) in DMSO-d_6 (150 MHz)

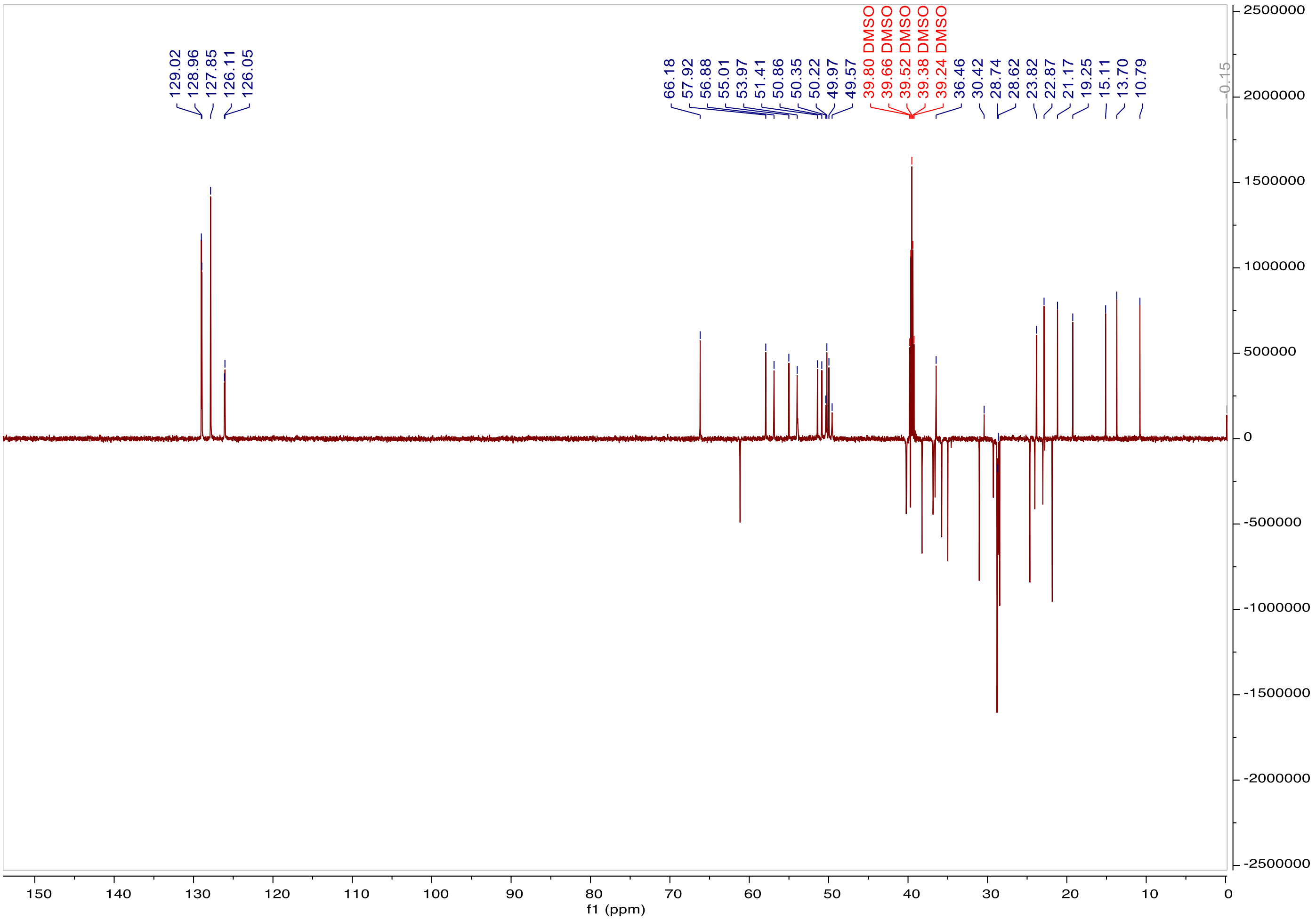

Figure S83. DEPT135 NMR spectrum of aquicidine L (synAQU5-L) in DMSO-d_6 (150 MHz)

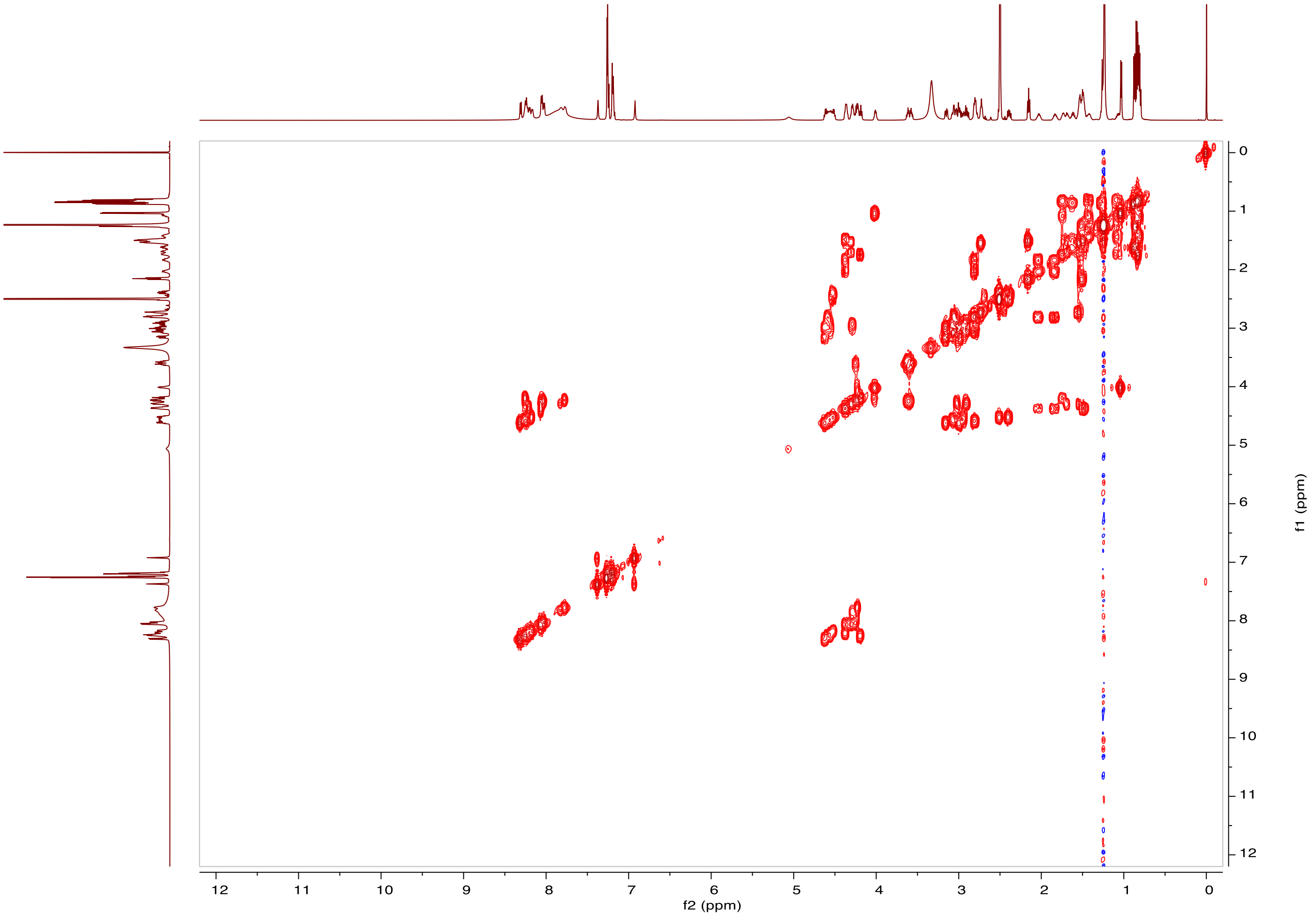

Figure S84. ^{1}H-^{1}H COSY NMR spectrum of aquicidine L (synAQU5-L) in DMSO-d_6 (600 MHz)

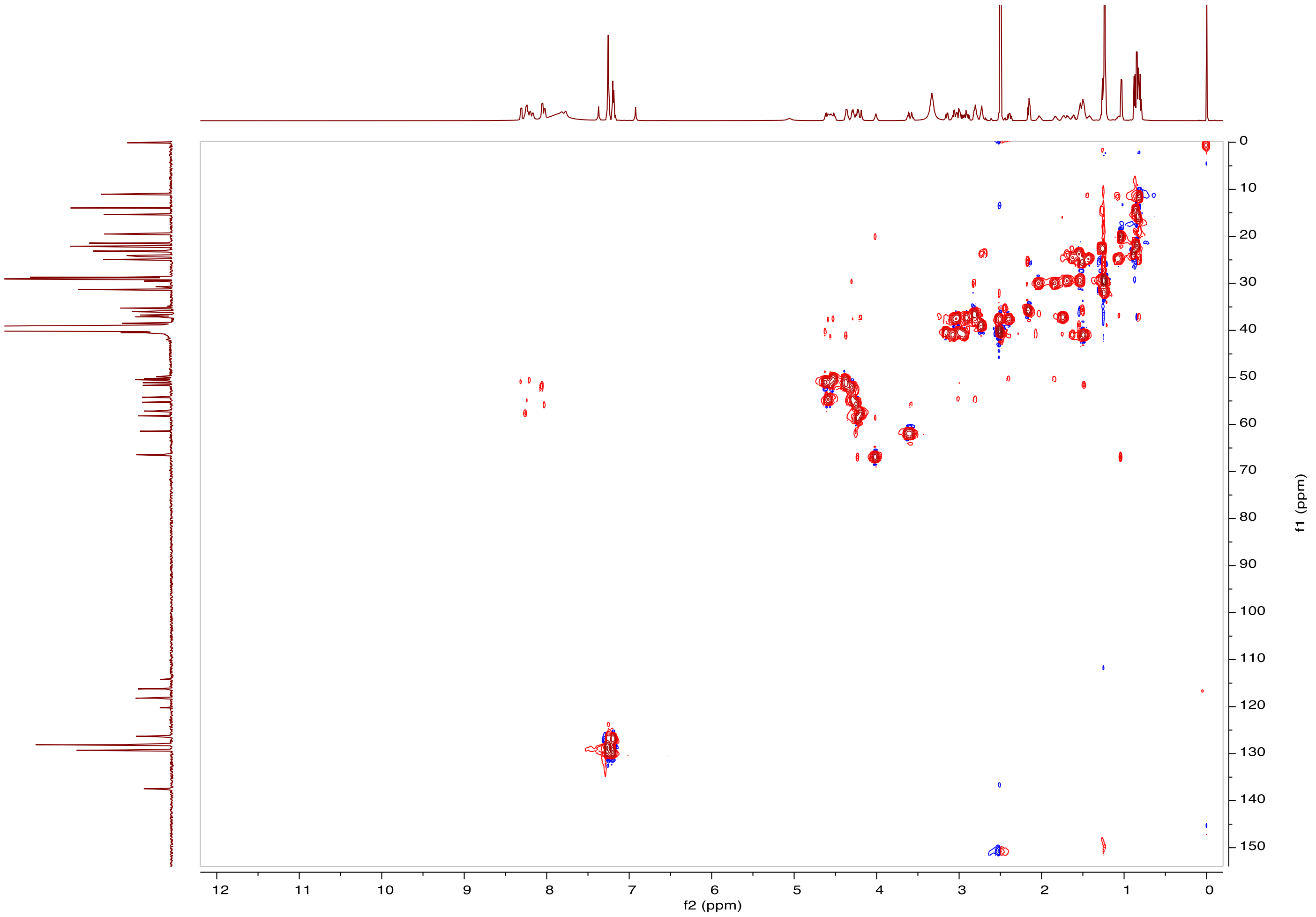

Figure S85. ^{1}H-^{13}C HSQC NMR spectrum of aquicidine L (synAQU5-L) in DMSO-d_6 (600 MHz)

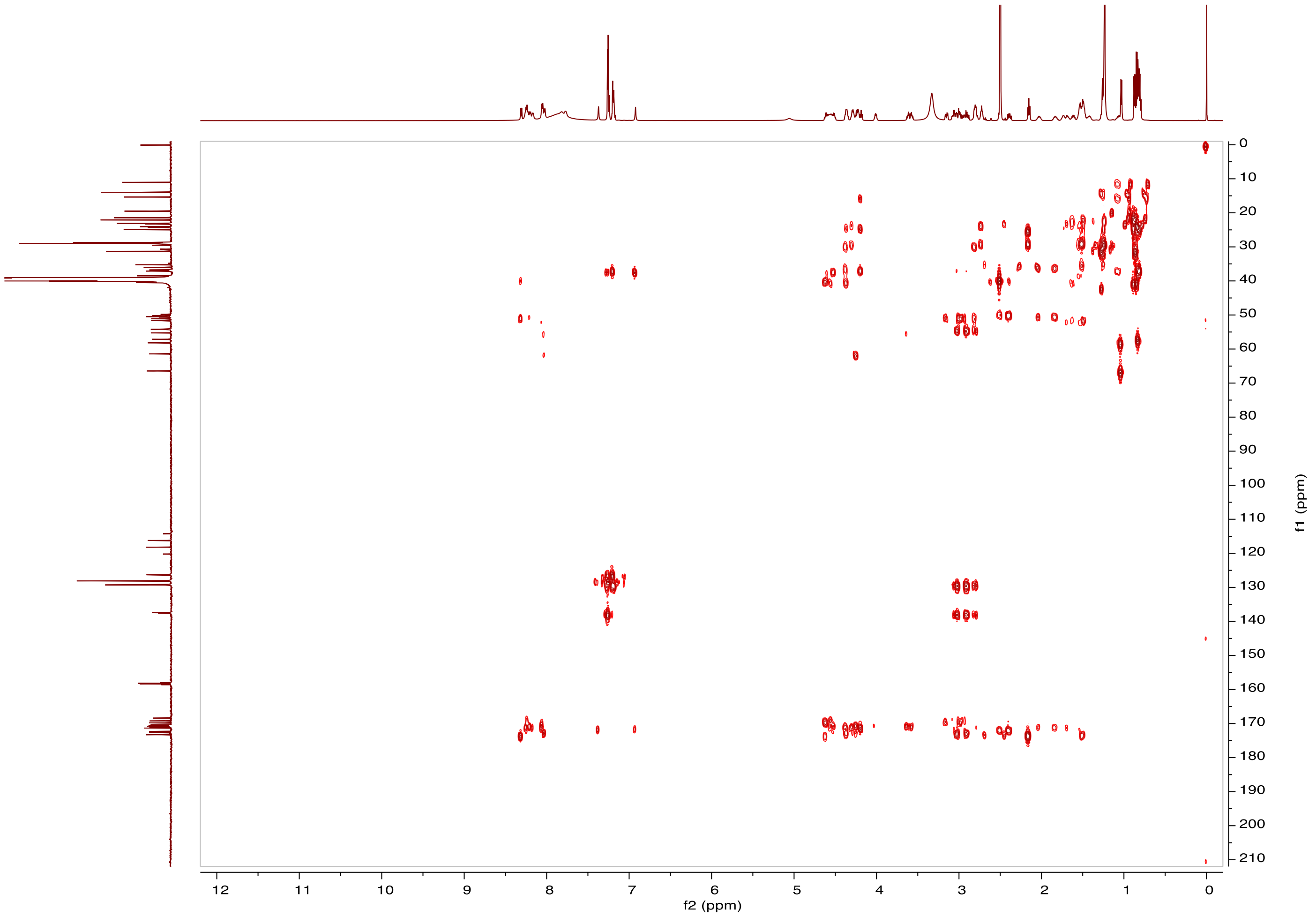

Figure S86. ^{1}H-^{13}C HMBC NMR spectrum of aquicidine L (synAQU5-L) in DMSO-d_6 (600 MHz)

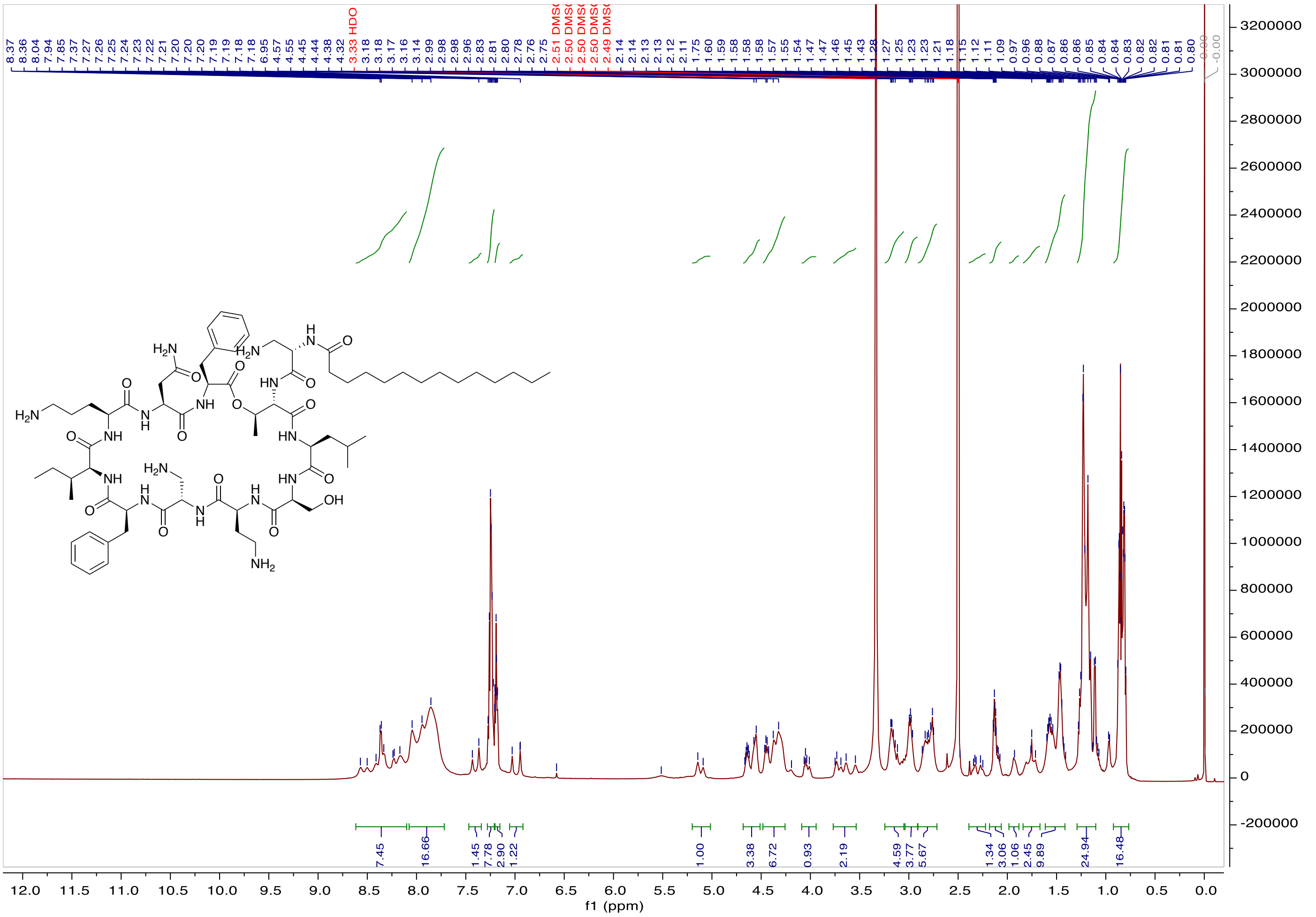

Figure S87. ^{1}H NMR spectrum of synAQU5-C2 in DMSO-d_6 (600 MHz)

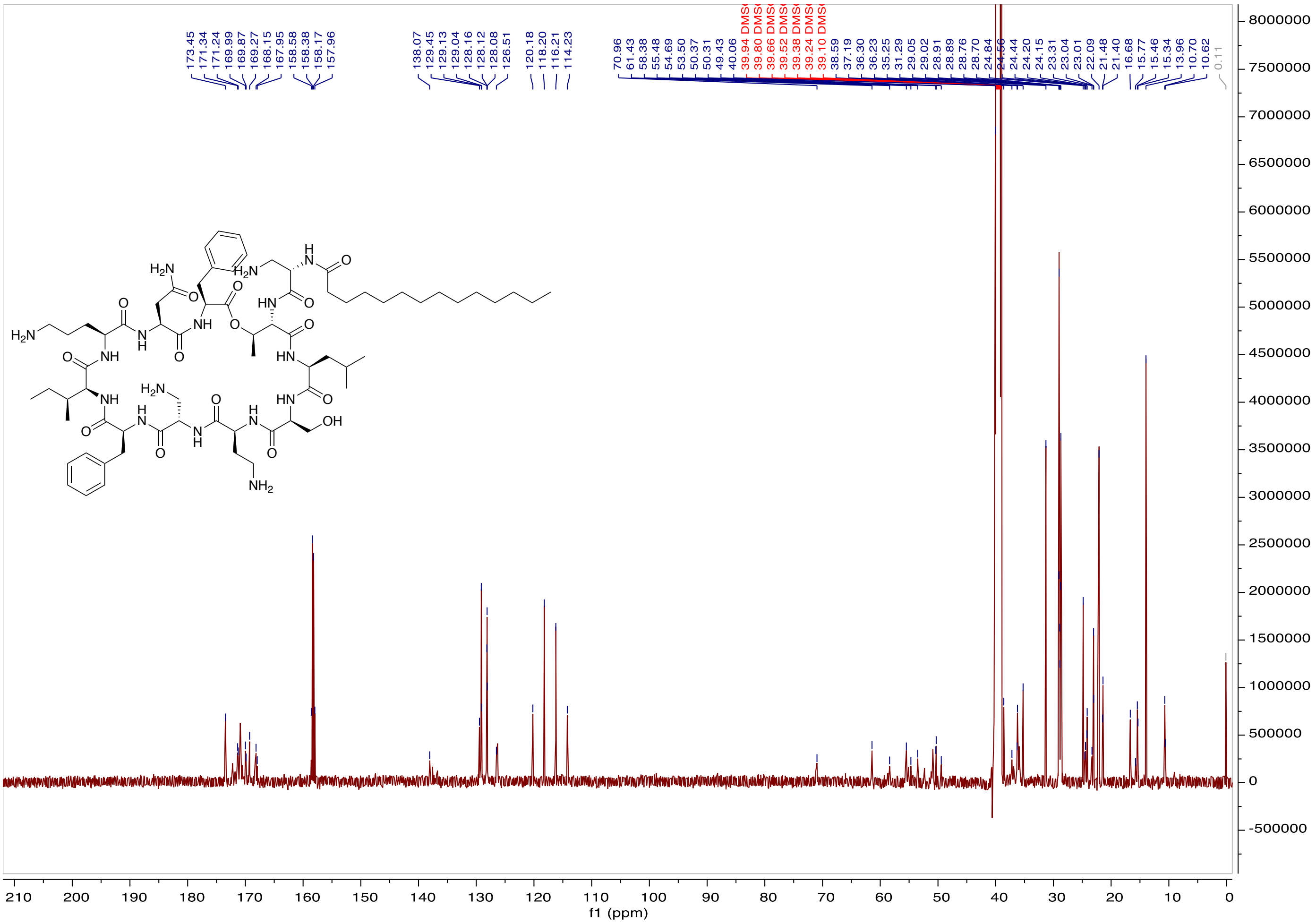

Figure S88. ^{13}C NMR spectrum of synAQU5-C2 in DMSO-d_6 (150 MHz)

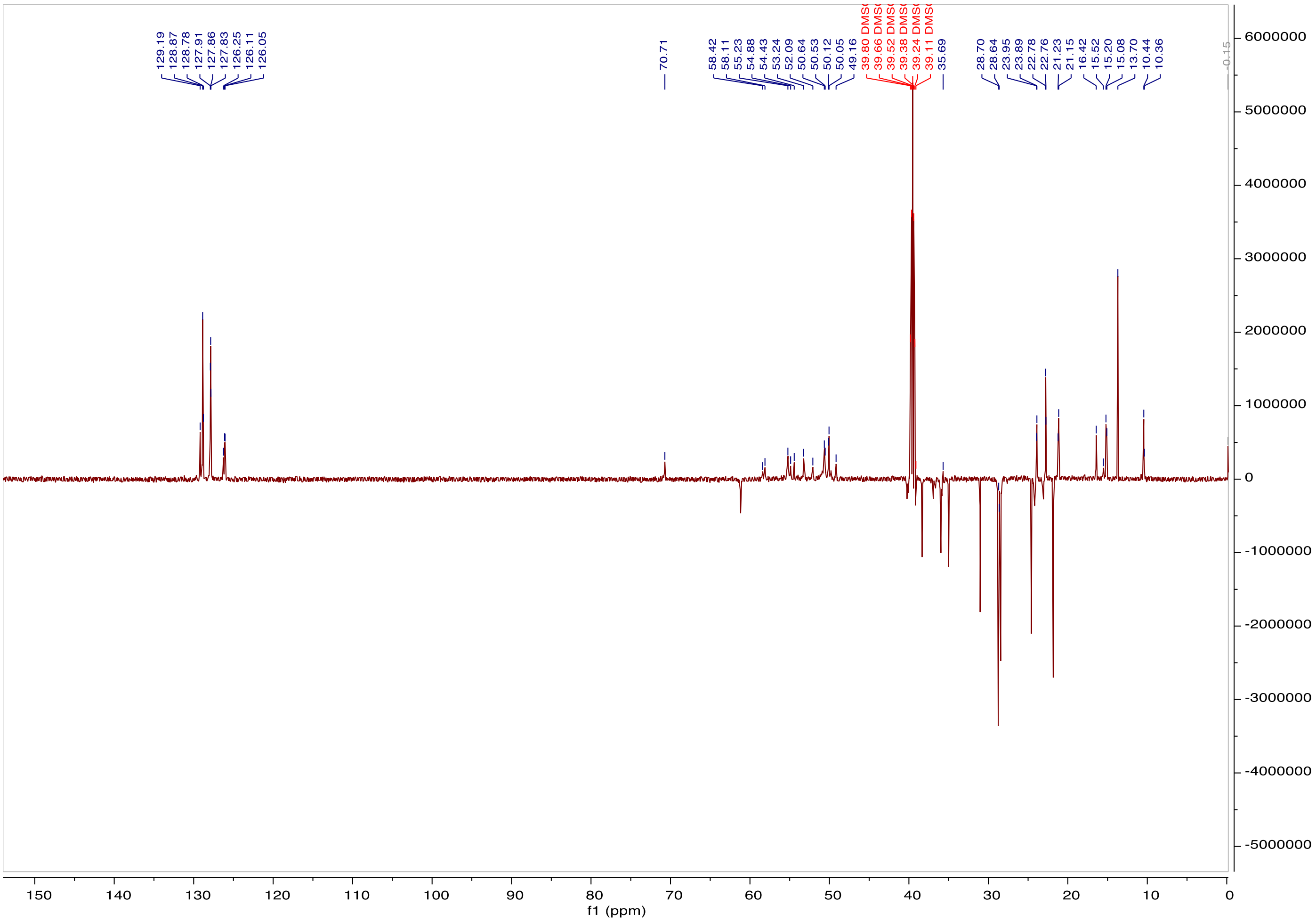

Figure S89. DEPT135 NMR spectrum of synAQU5-C2 in DMSO-d_6 (150 MHz)

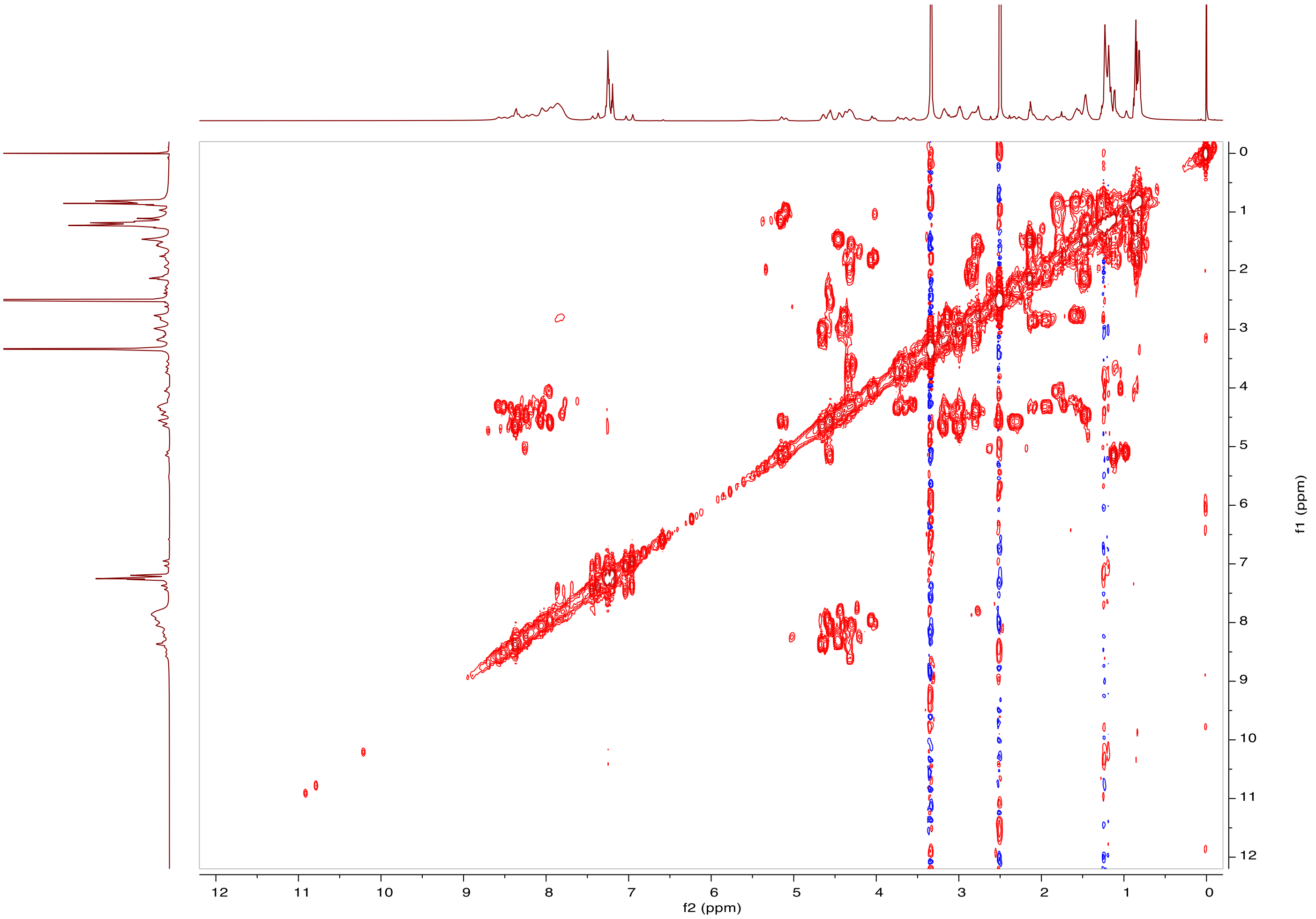

Figure S90. ^{1}H-^{1}H COSY NMR spectrum of synAQU5-C2 in DMSO-d_6 (600 MHz)

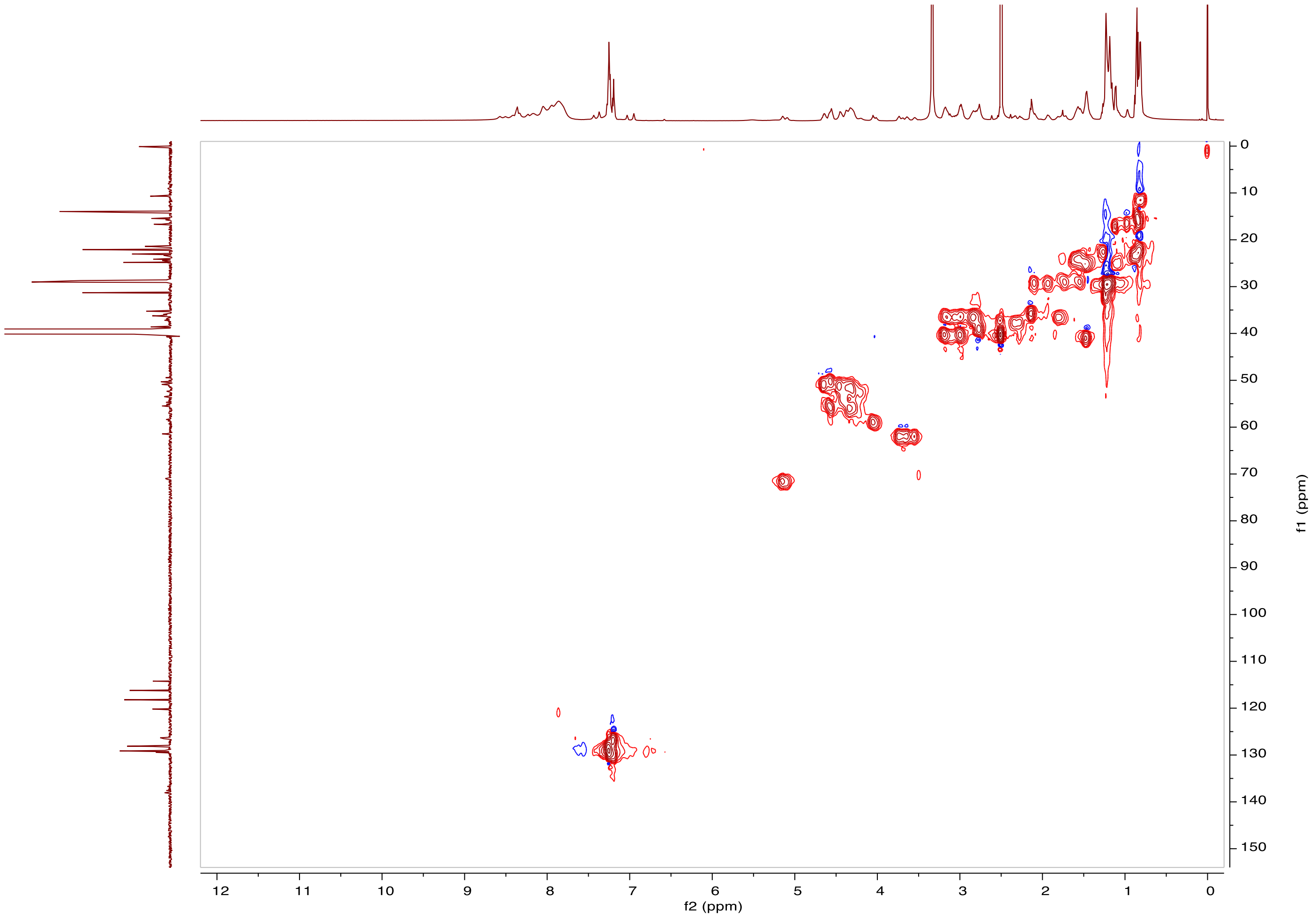

Figure S91. ^{1}H-^{13}C HSQC NMR spectrum of synAQU5-C2 in DMSO-d_6 (600 MHz)

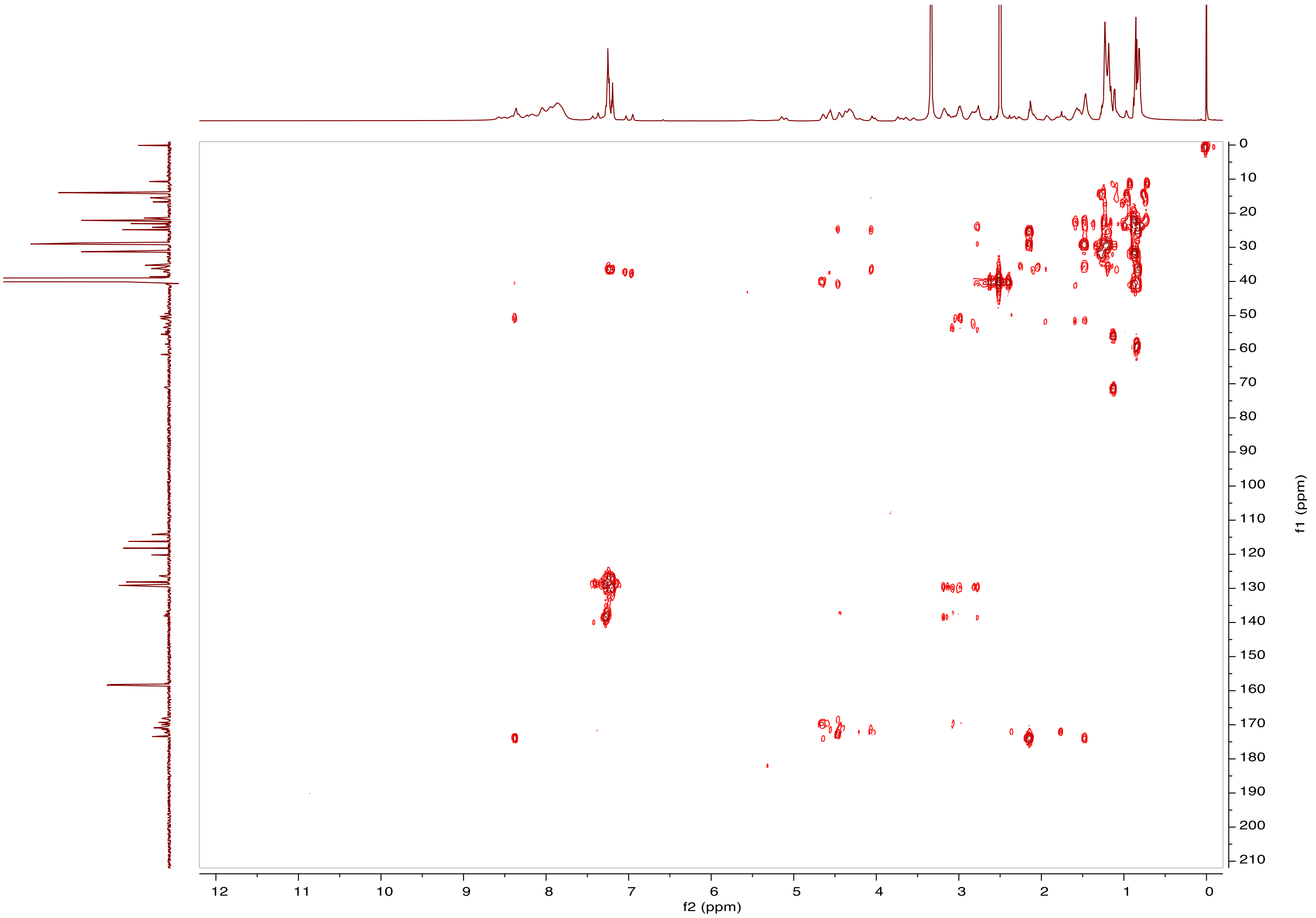

Figure S92. ^{1}H-^{13}C HMBC NMR spectrum of synAQU5-C2 in DMSO-d_6 (600 MHz)

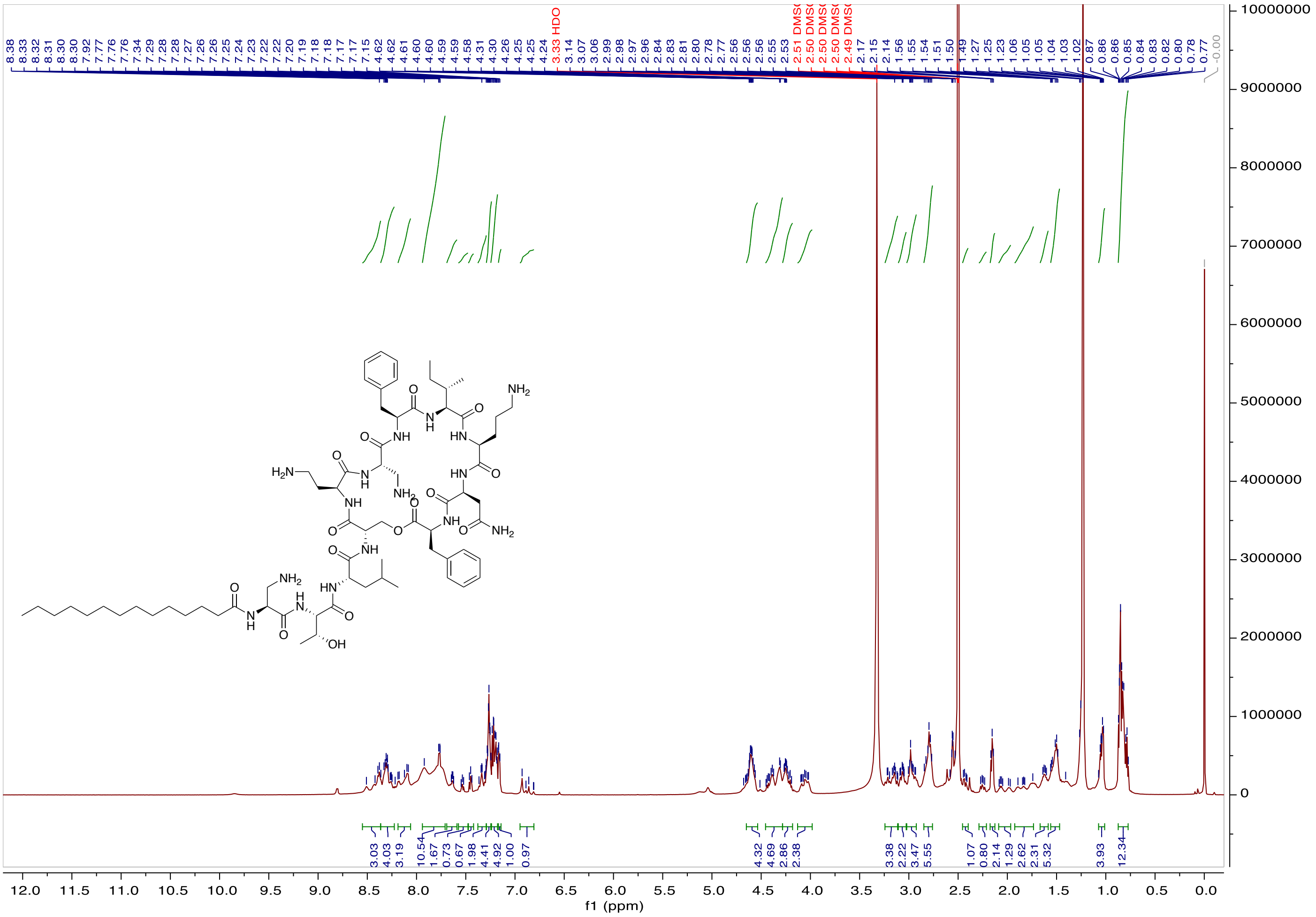

Figure S93. ^{1}H NMR spectrum of aquicidine C4 (synAQU5-C4) in DMSO-d_6 (600 MHz)

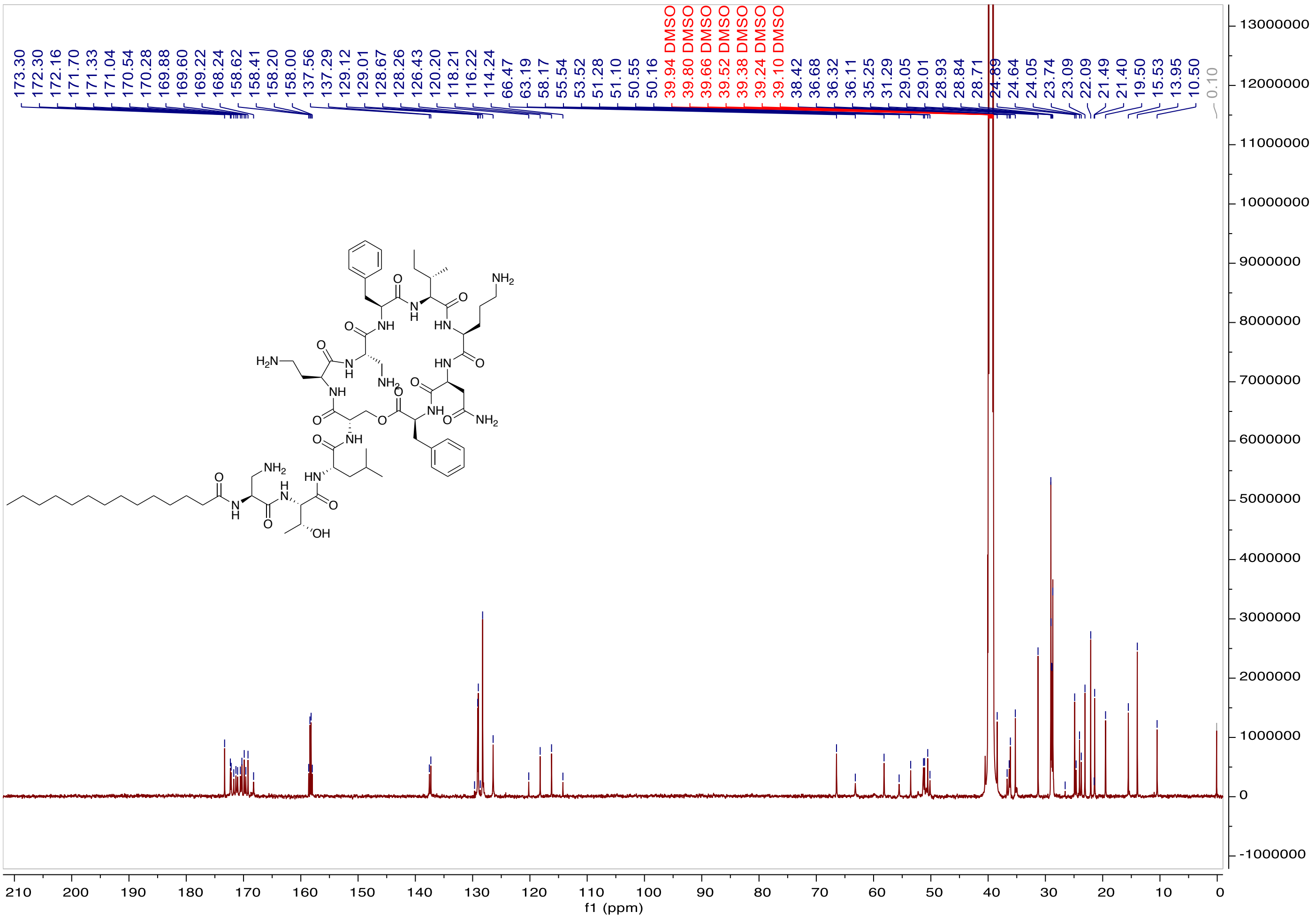

Figure S94. ^{13}C NMR spectrum of aquicidine C4 (synAQU5-C4) in DMSO-d_6 (150 MHz)

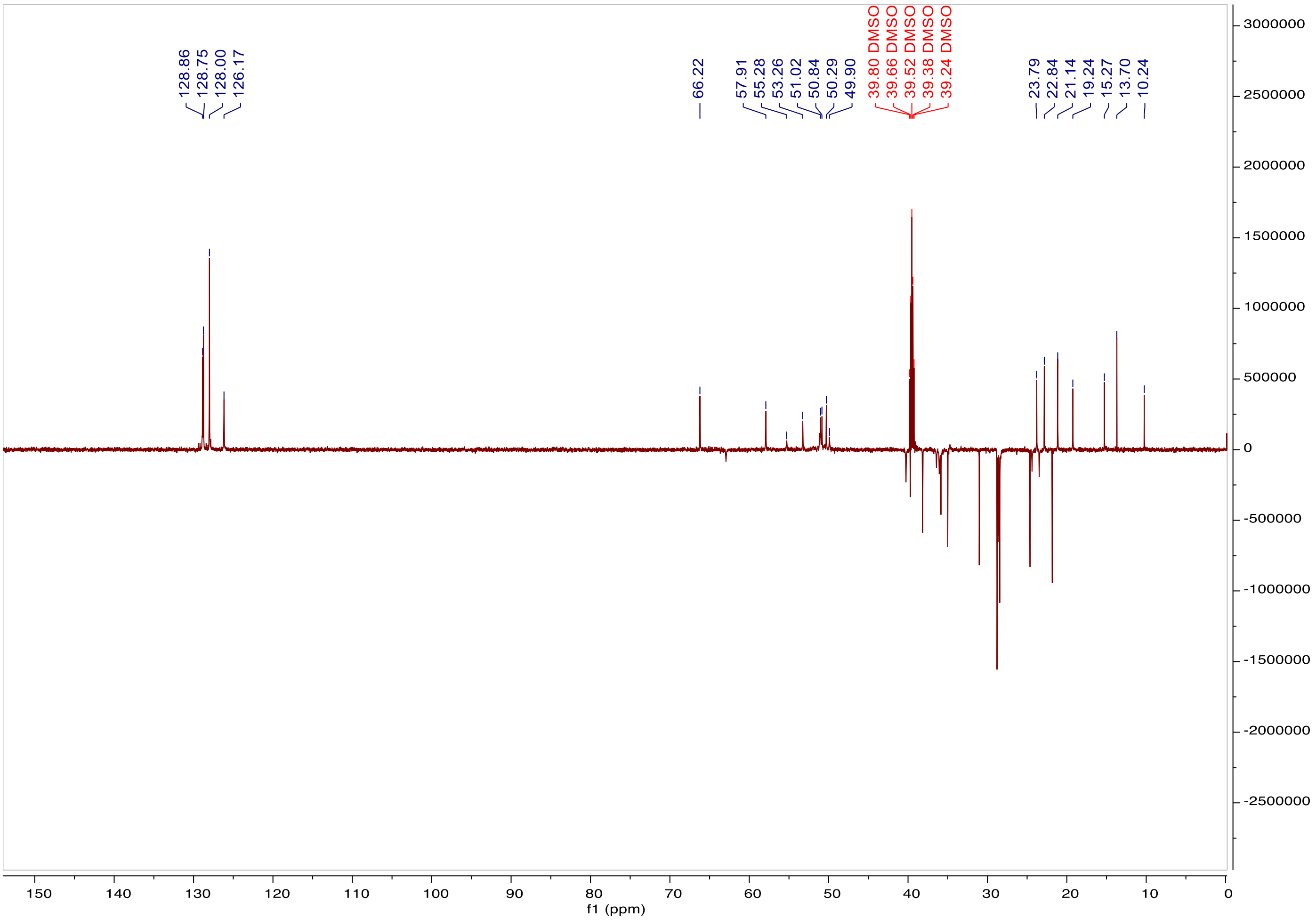

Figure S95. DEPT135 NMR spectrum of aquicidine C4 (synAQU5-C4) in DMSO-d_6 (150 MHz)

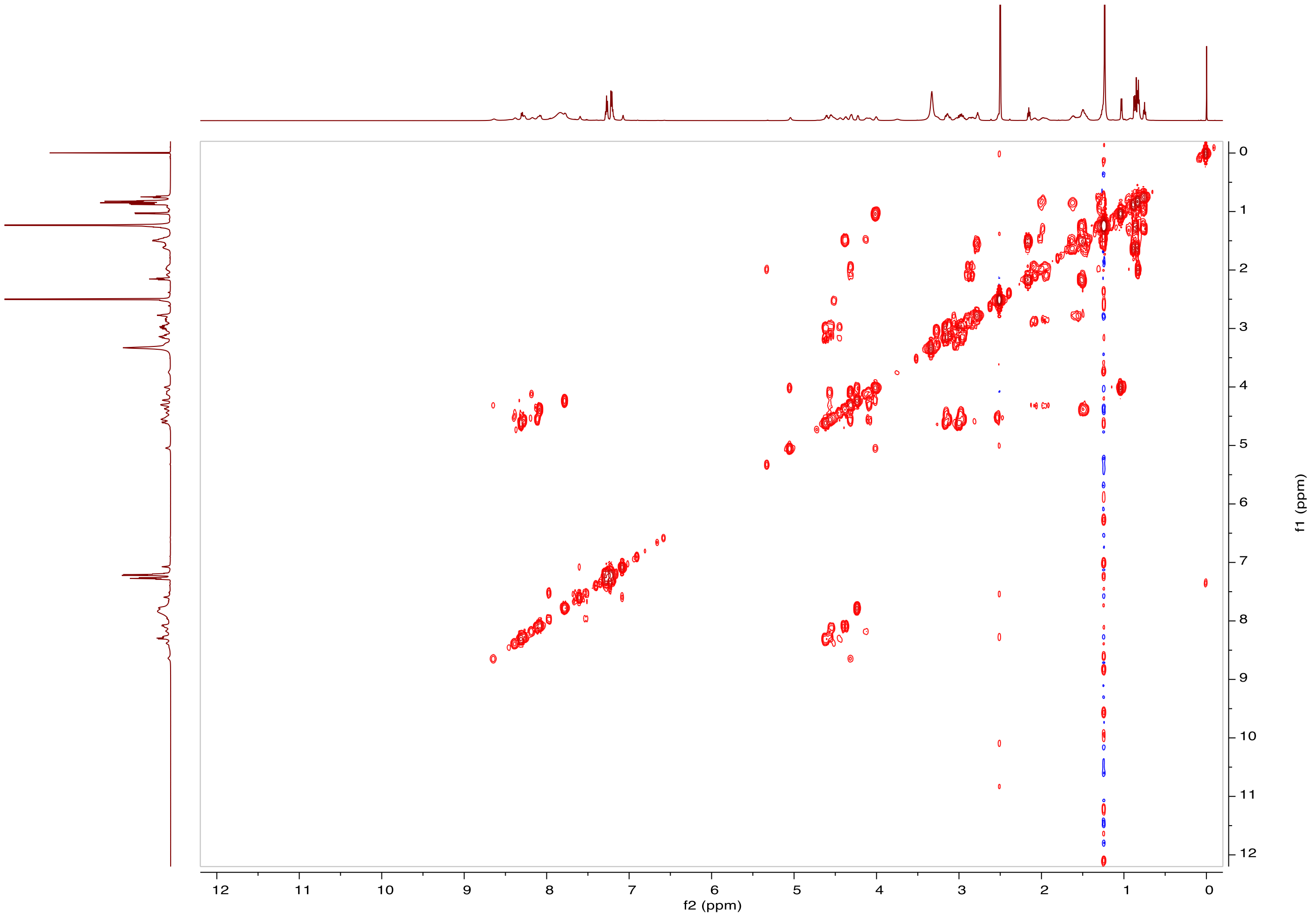

Figure S96. ^{1}H-^{1}H COSY NMR spectrum of aquicidine C4 (synAQU5-C4) in DMSO-d_6 (600 MHz)

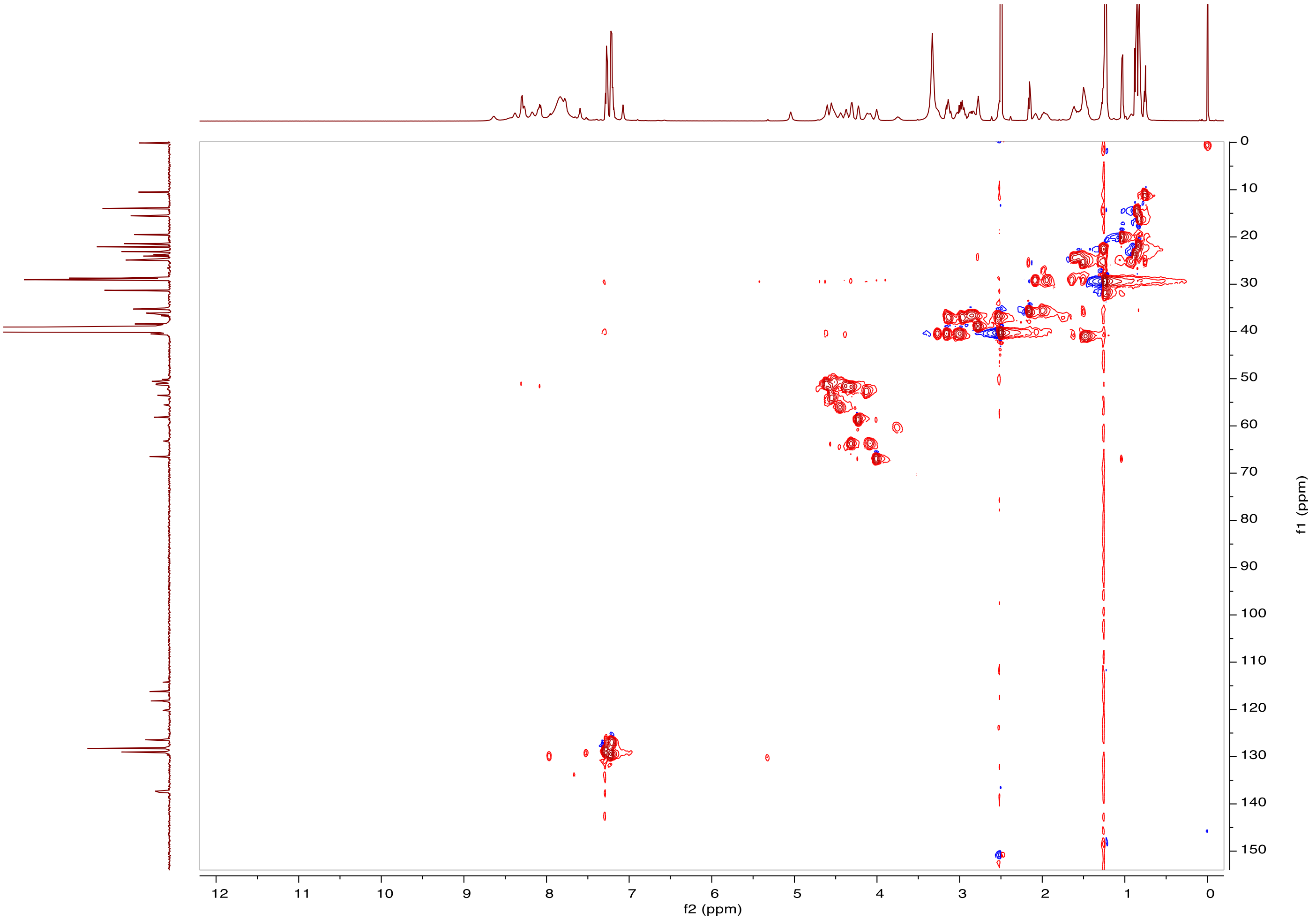

Figure S97. ^{1}H-^{13}C HSQC NMR spectrum of aquicidine C4 (synAQU5-C4) in DMSO-d_6 (600 MHz)

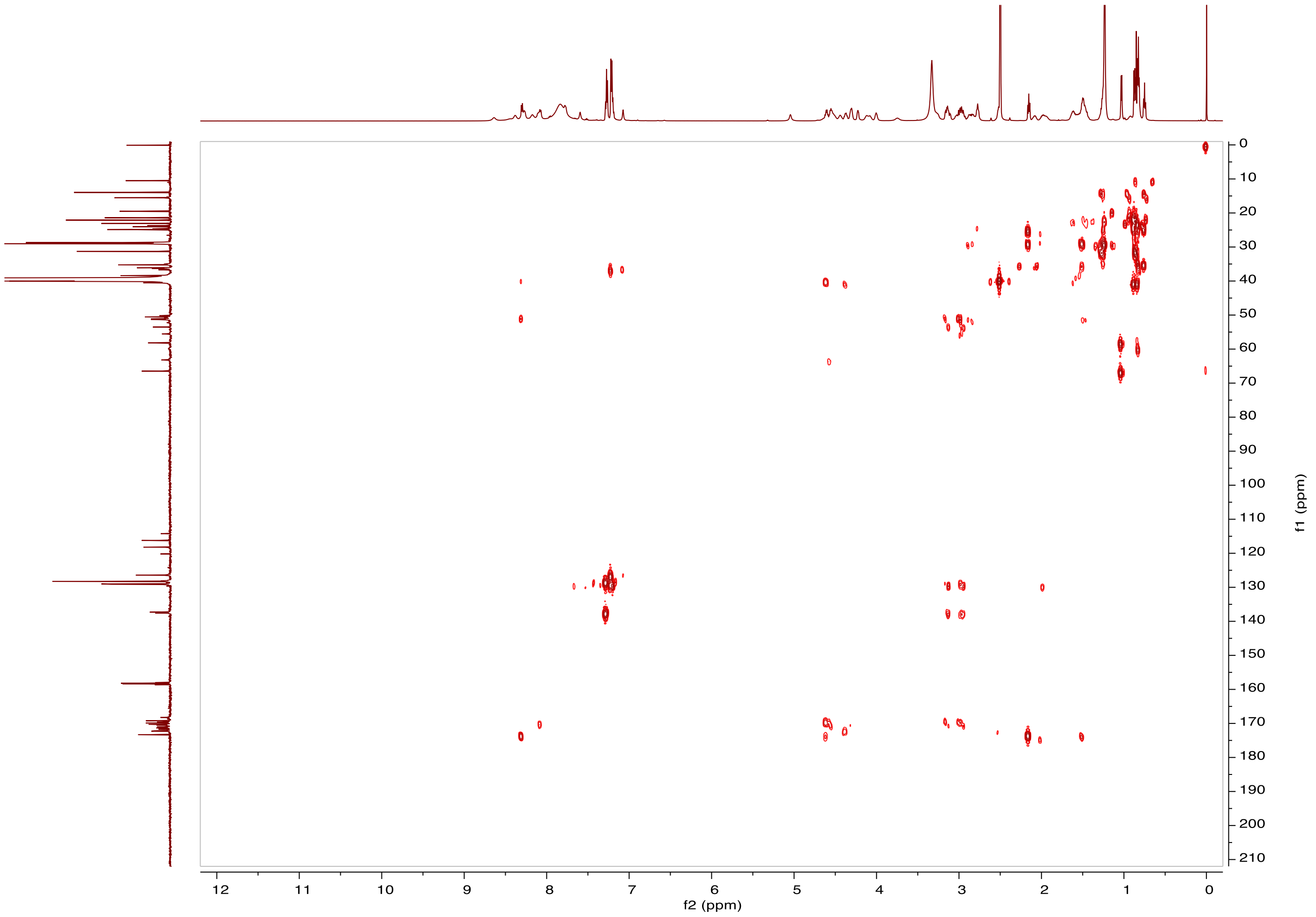

Figure S98. ^{1}H-^{13}C HMBC NMR spectrum of aquicidine C4 (synAQU5-C4) in DMSO-d_6 (600 MHz)

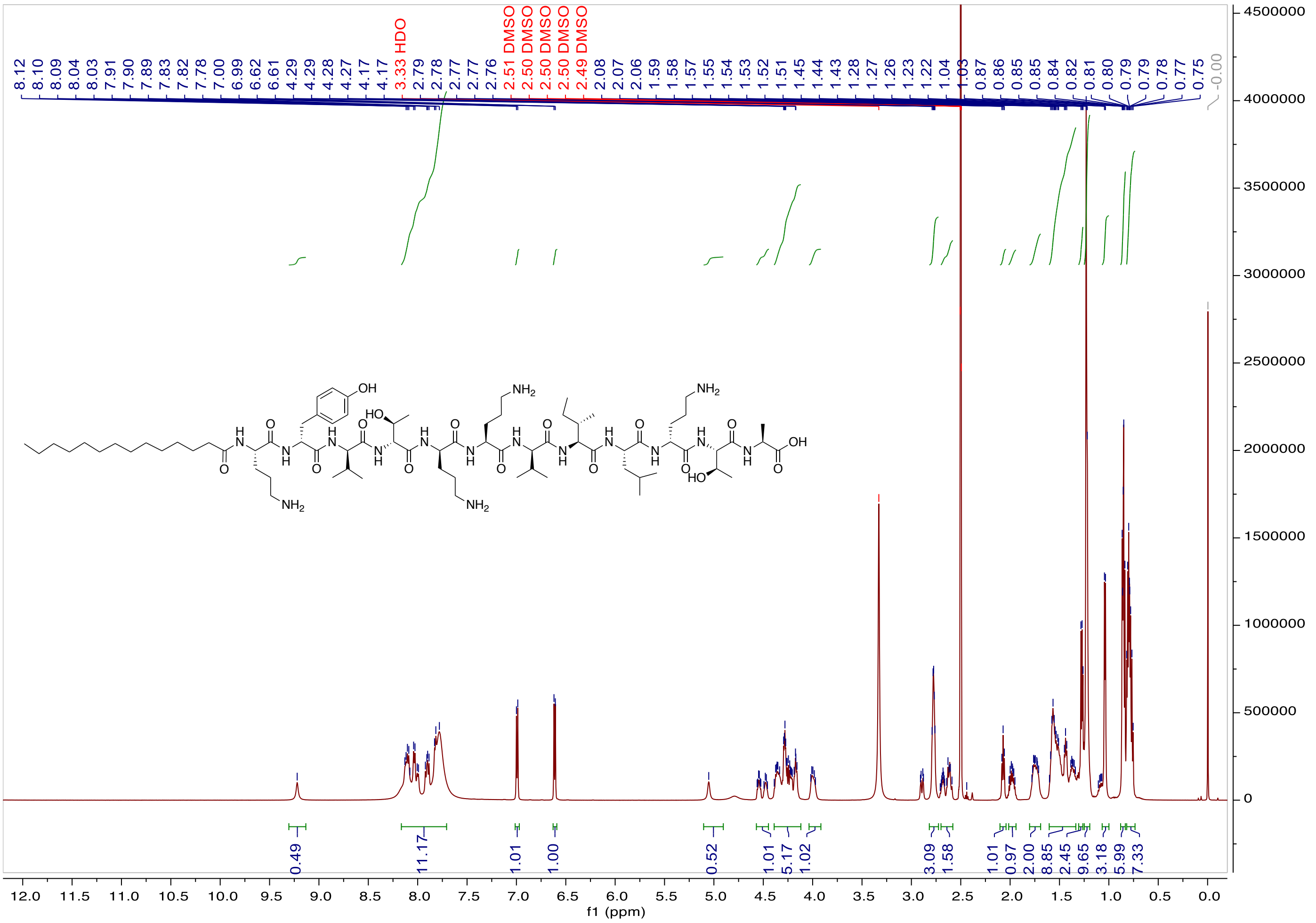

Figure S99. ^{1}H NMR spectrum of synAQU6-L in DMSO-d_6 (600 MHz)

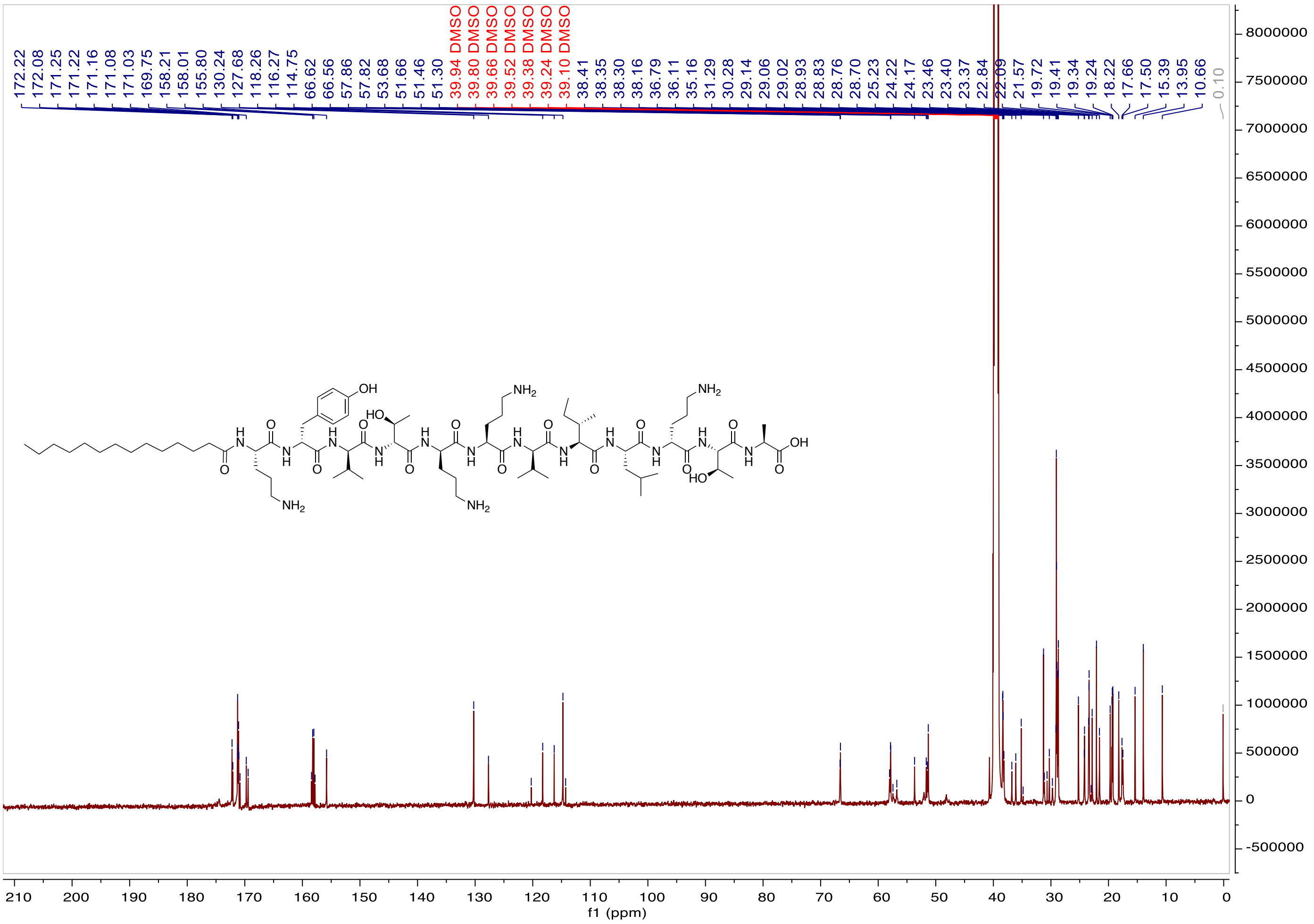

Figure S100. ^{13}C NMR spectrum of synAQU6-L in DMSO-d_6 (150 MHz)

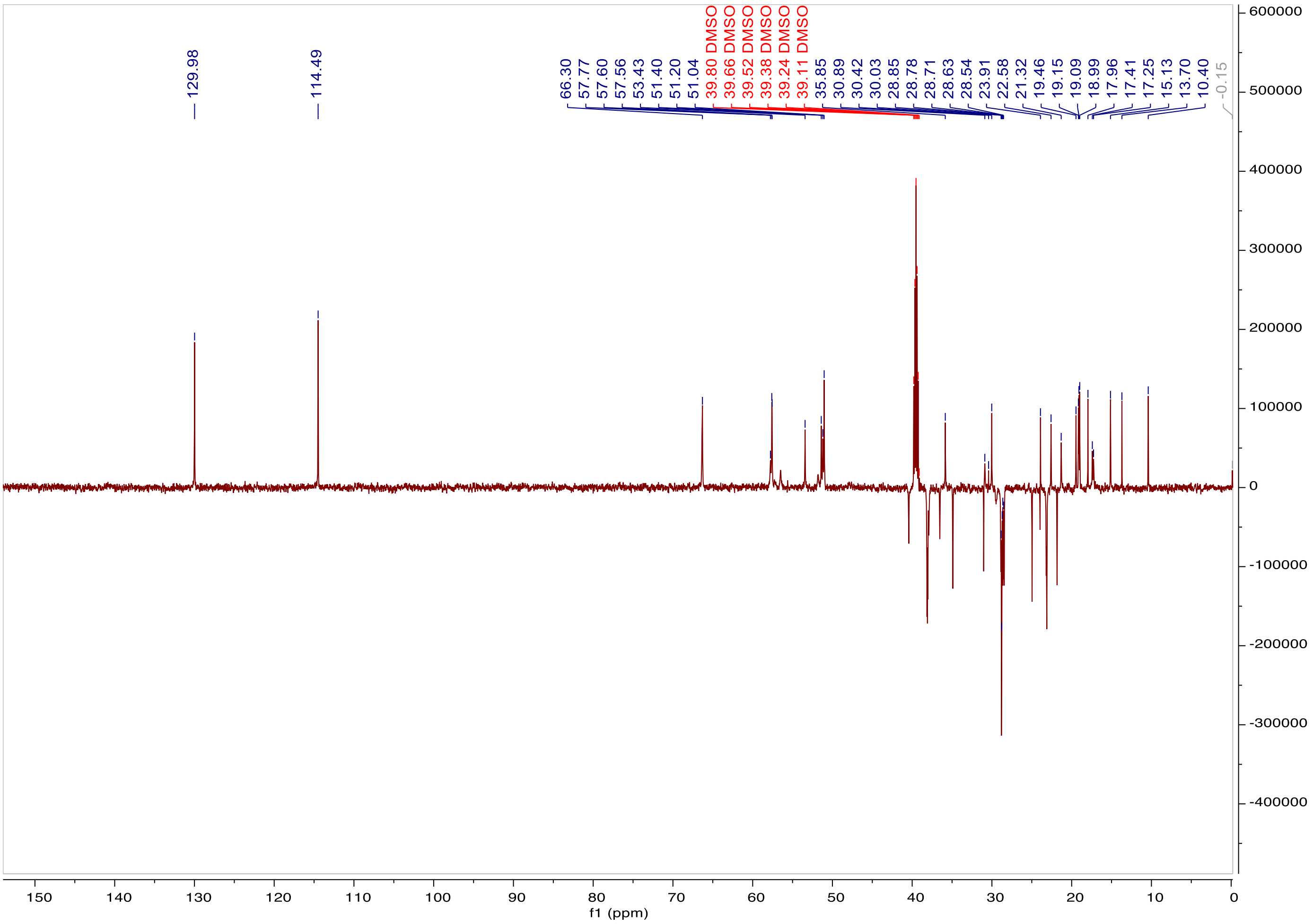

Figure S101. DEPT135 NMR spectrum of synAQU6-L in DMSO-d_6 (150 MHz)

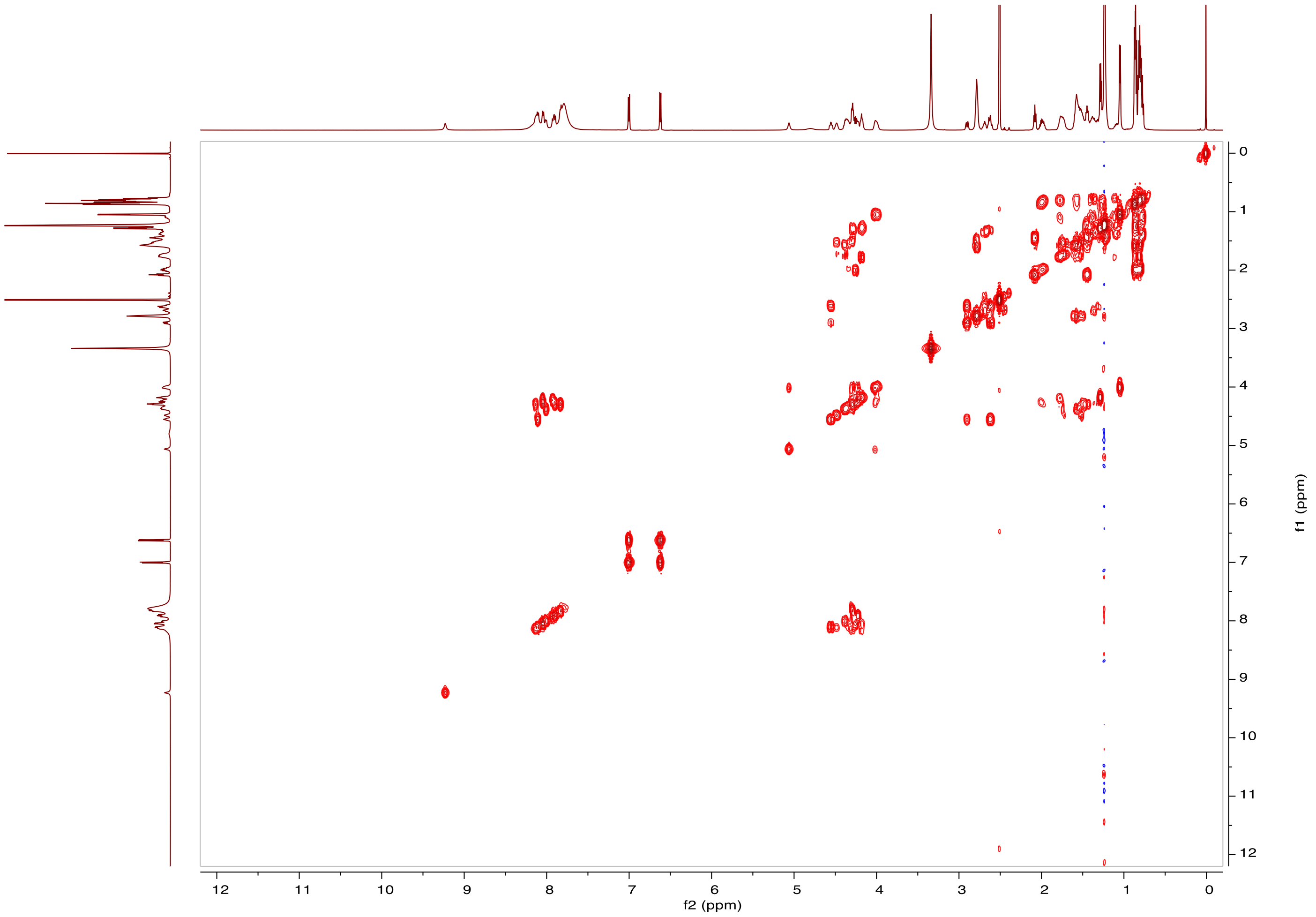

Figure S102. ^1H-^1H COSY NMR spectrum of synAQU6-L in DMSO-d_6 (600 MHz)

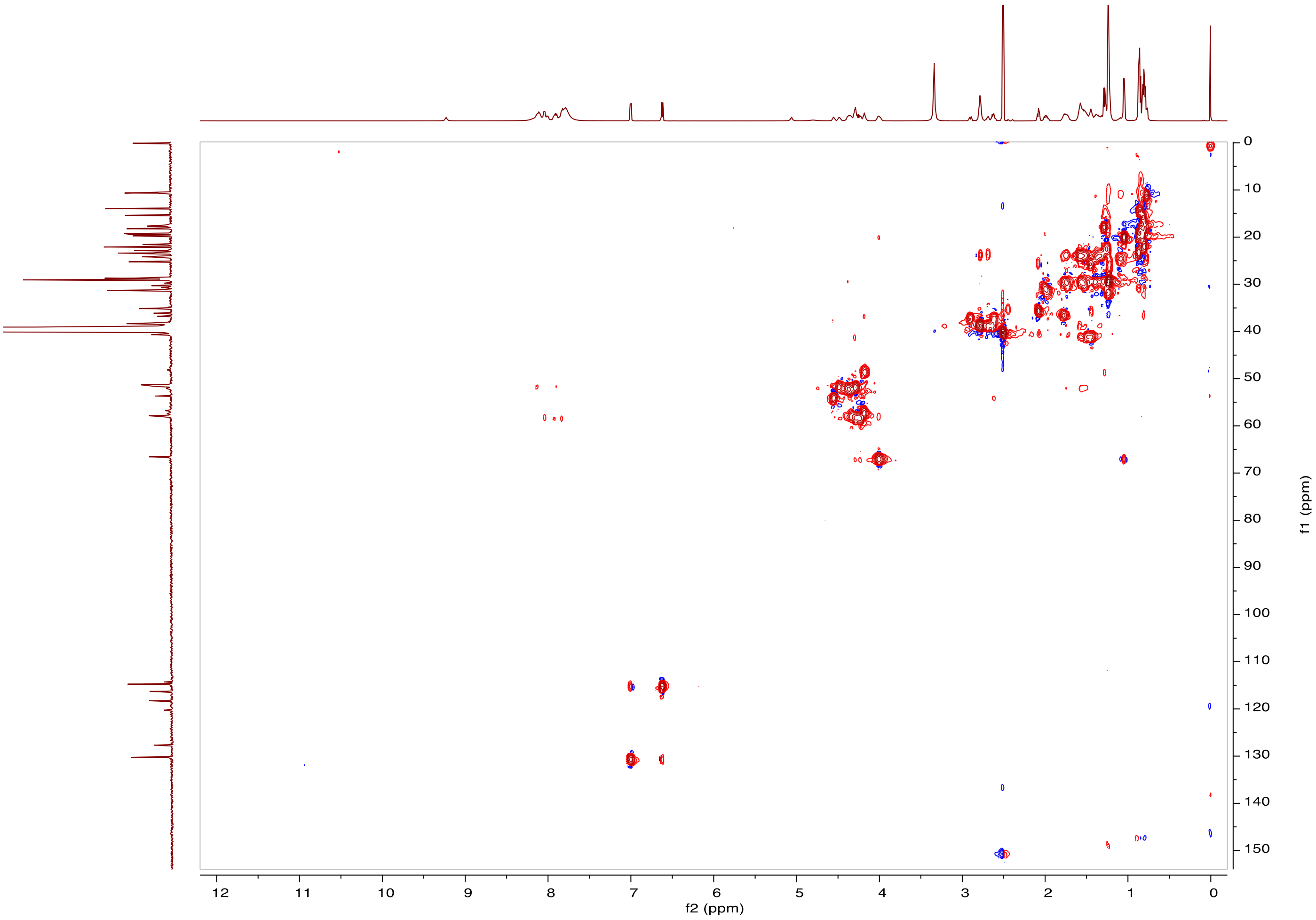

Figure S103. ^{1}H-^{13}C HSQC NMR spectrum of synAQU6-L in DMSO-d_6 (600 MHz)

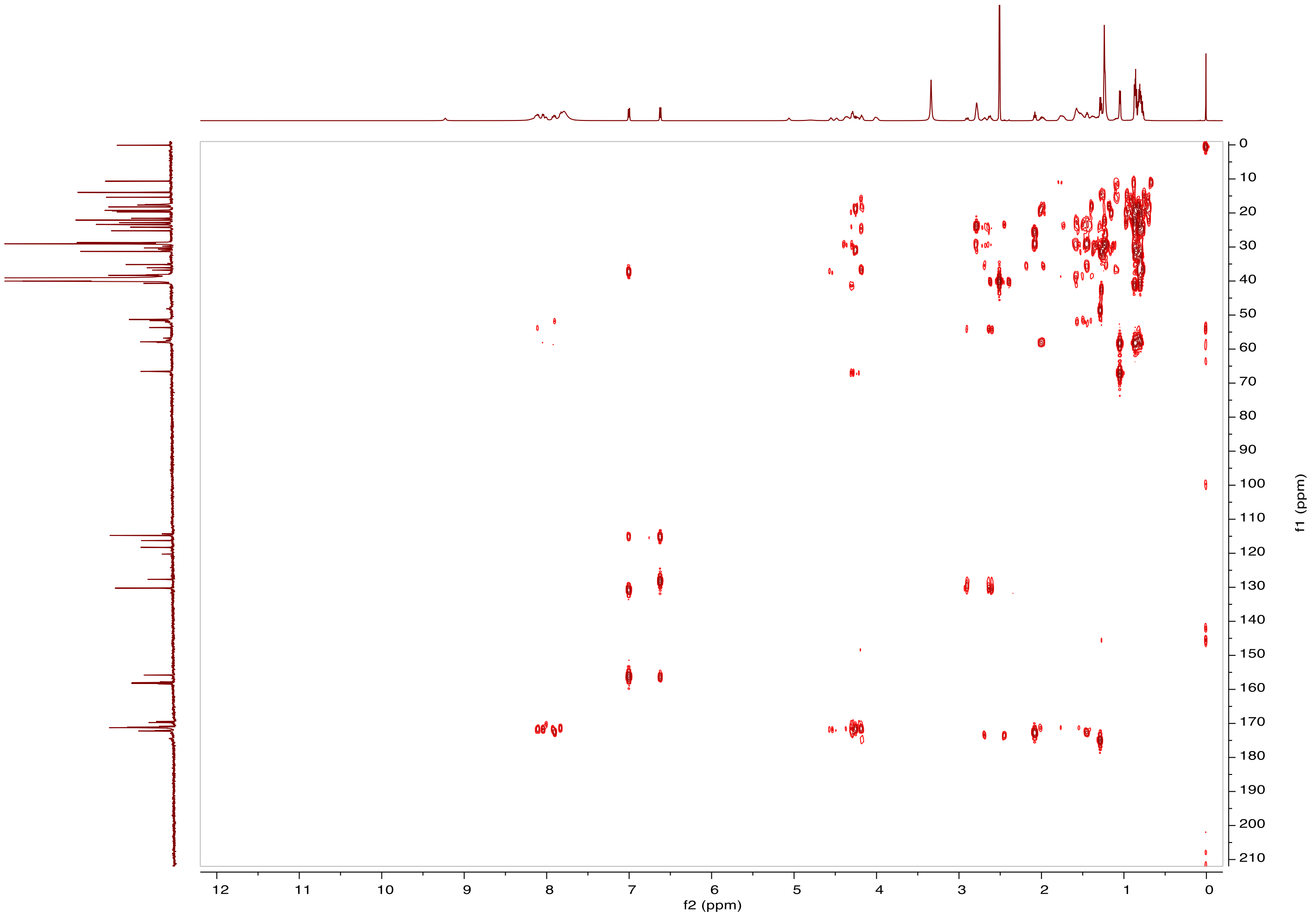

Figure S104. ^{1}H-^{13}C HMBC NMR spectrum of synAQU6-L in DMSO-d_6 (600 MHz)

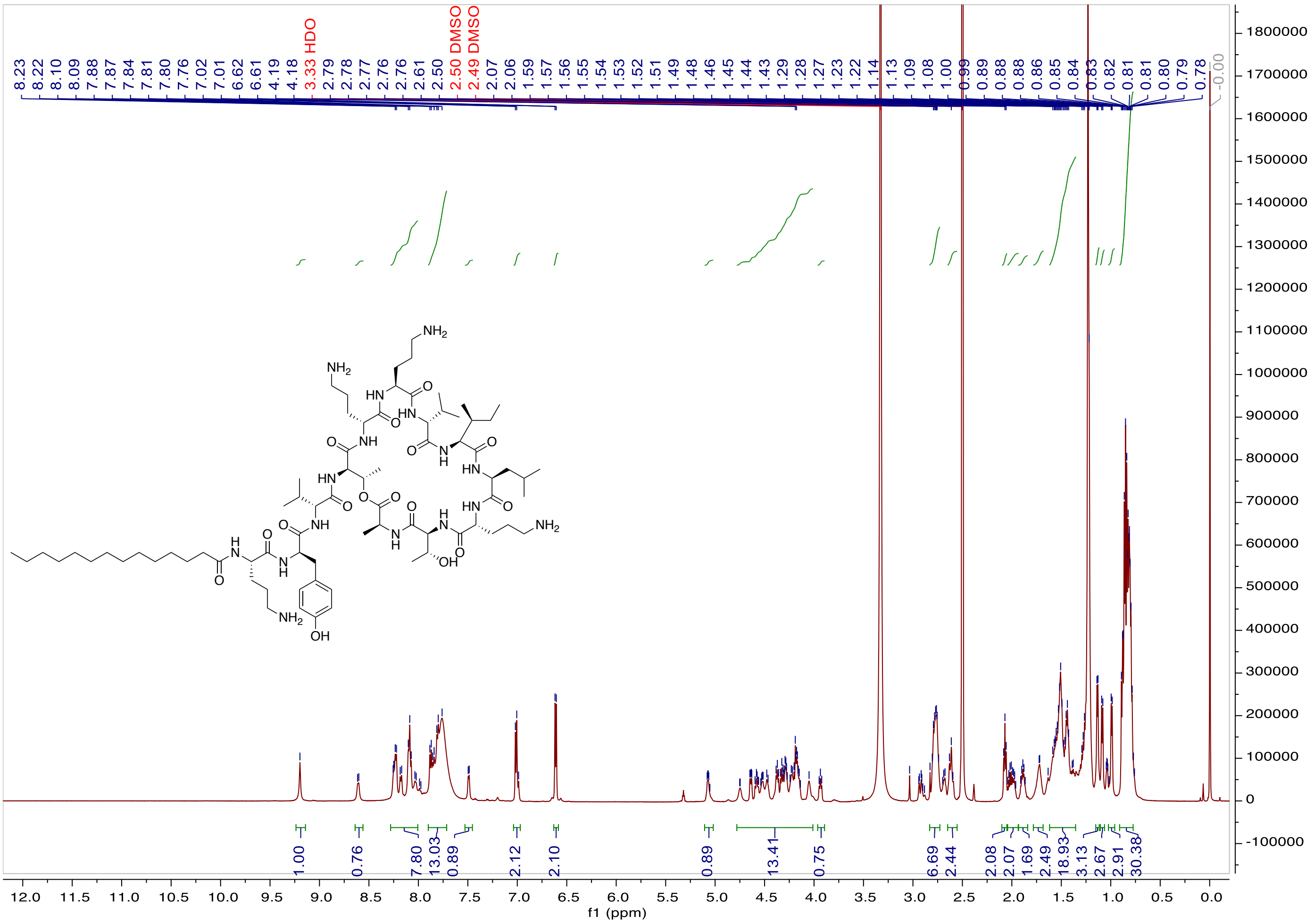

Figure S105. ^{1}H NMR spectrum of synAQU6-C4 in DMSO-d_6 (600 MHz)

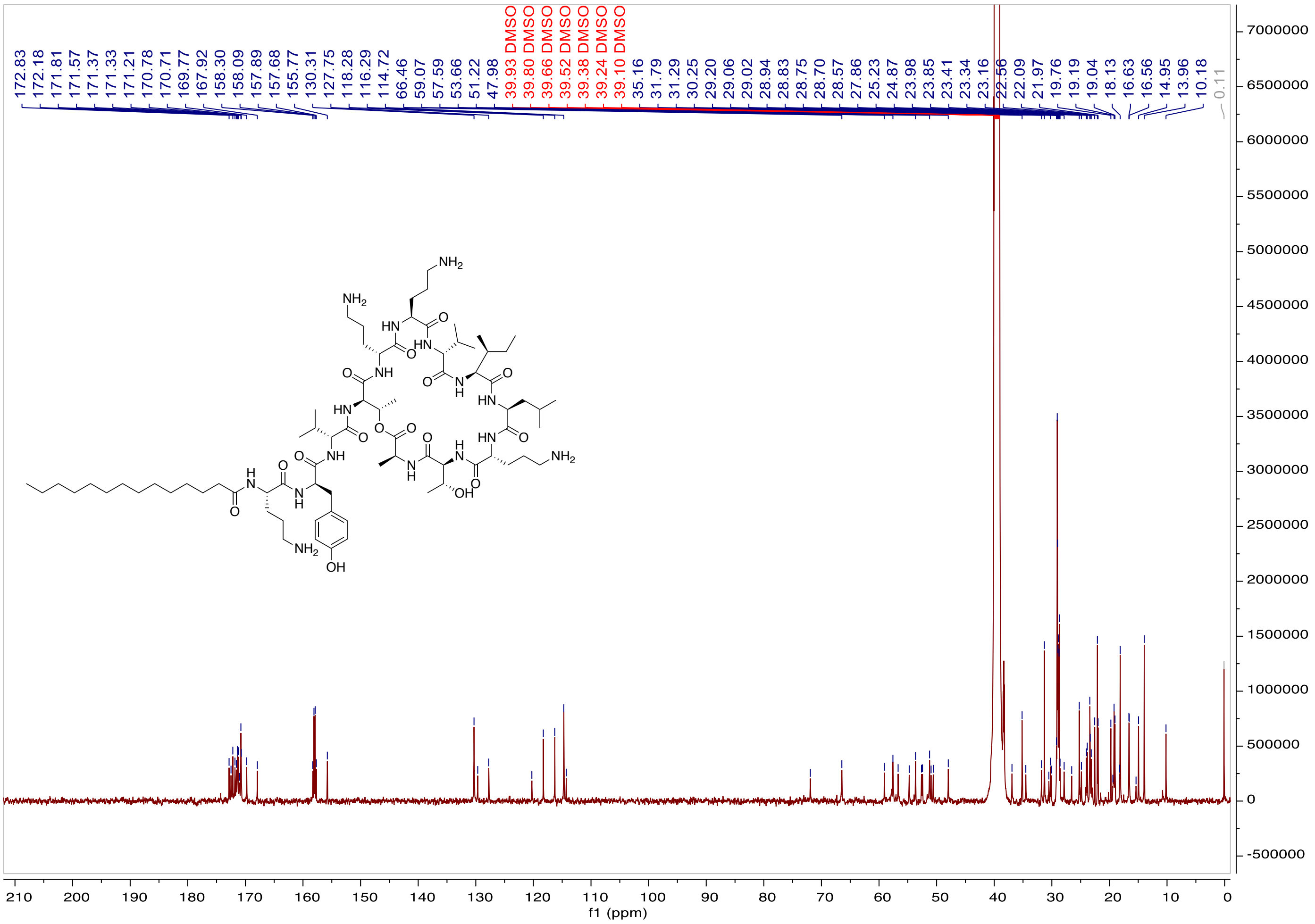

Figure S106. ^{13}C NMR spectrum of synAQU6-C4 in DMSO-d_6 (150 MHz)

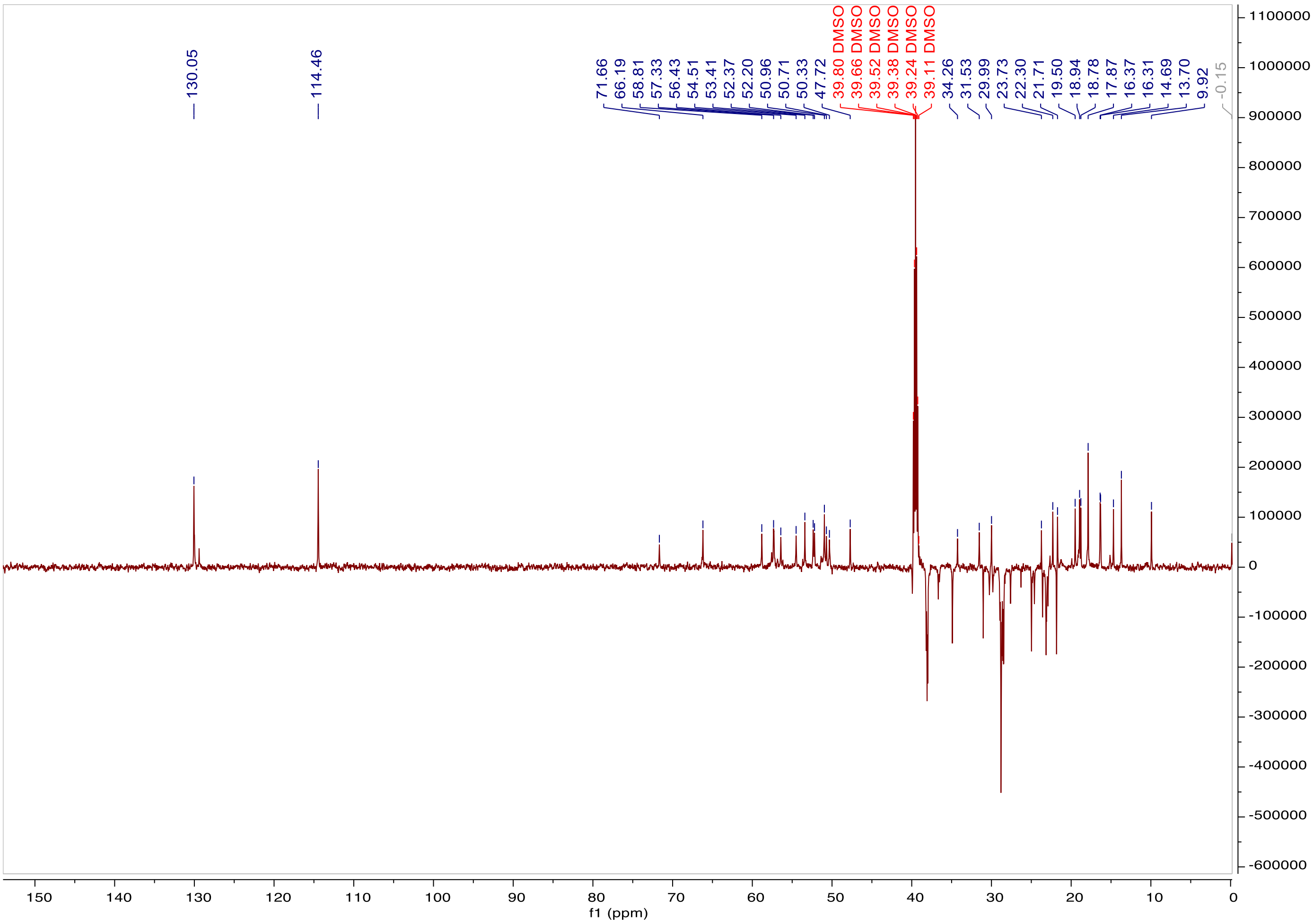

Figure S107. DEPT135 NMR spectrum of synAQU6-C4 in DMSO-d_6 (150 MHz)

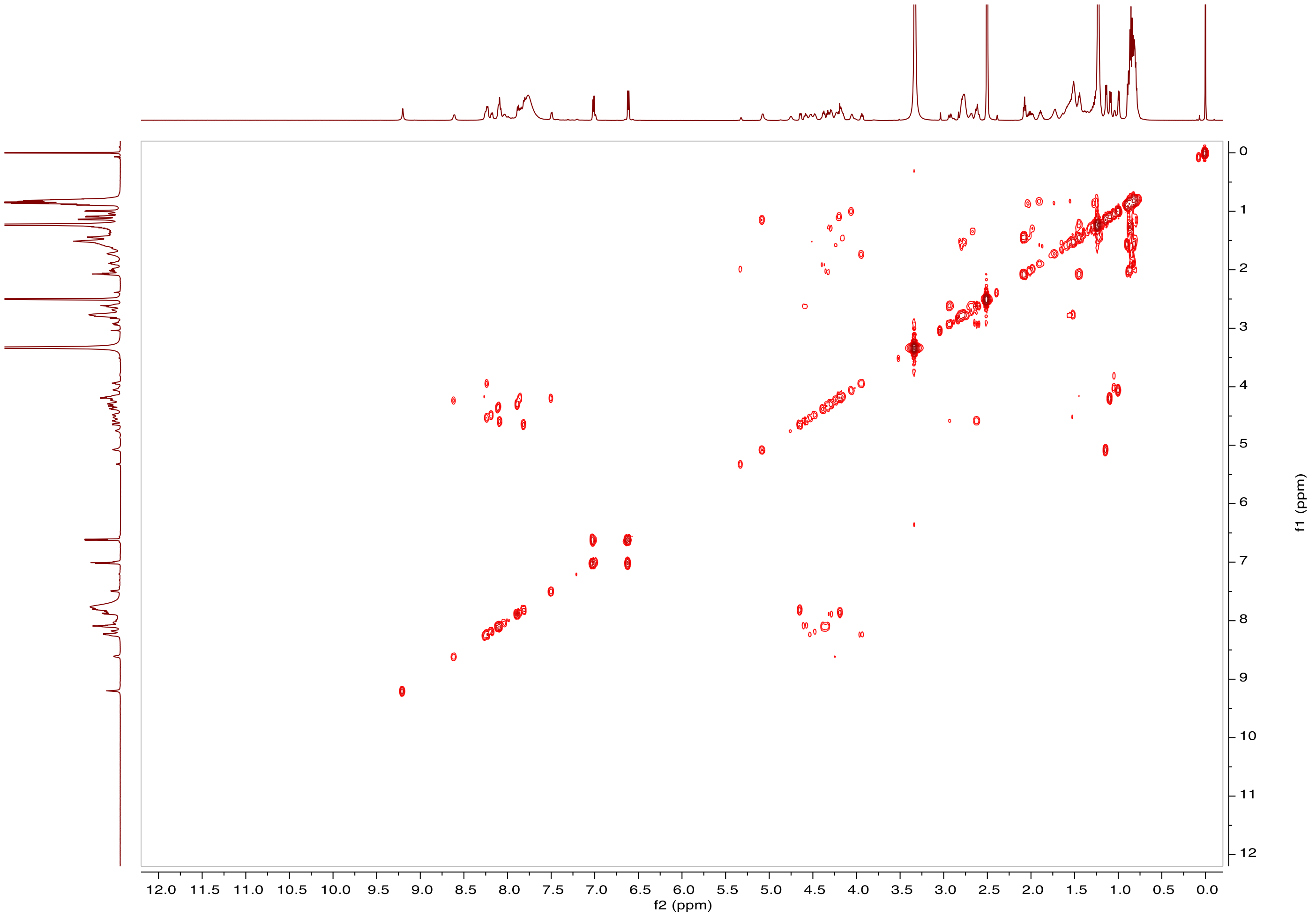

Figure S108. ^{1}H-^{1}H COSY NMR spectrum of synAQU6-C4 in DMSO-d_6 (600 MHz)

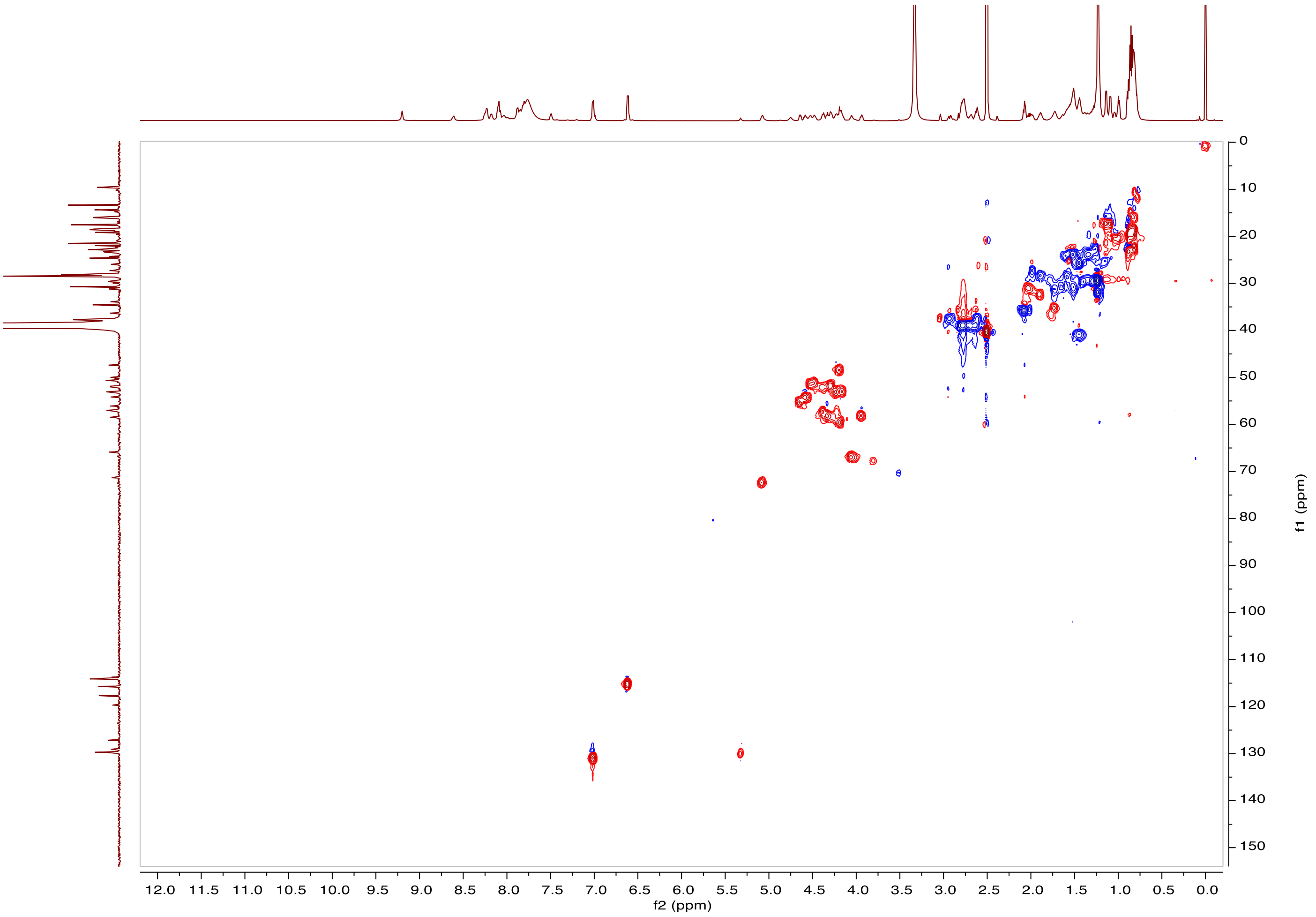

Figure S109. ^{1}H-^{13}C HSQC NMR spectrum of synAQU6-C4 in DMSO-d_6 (600 MHz)

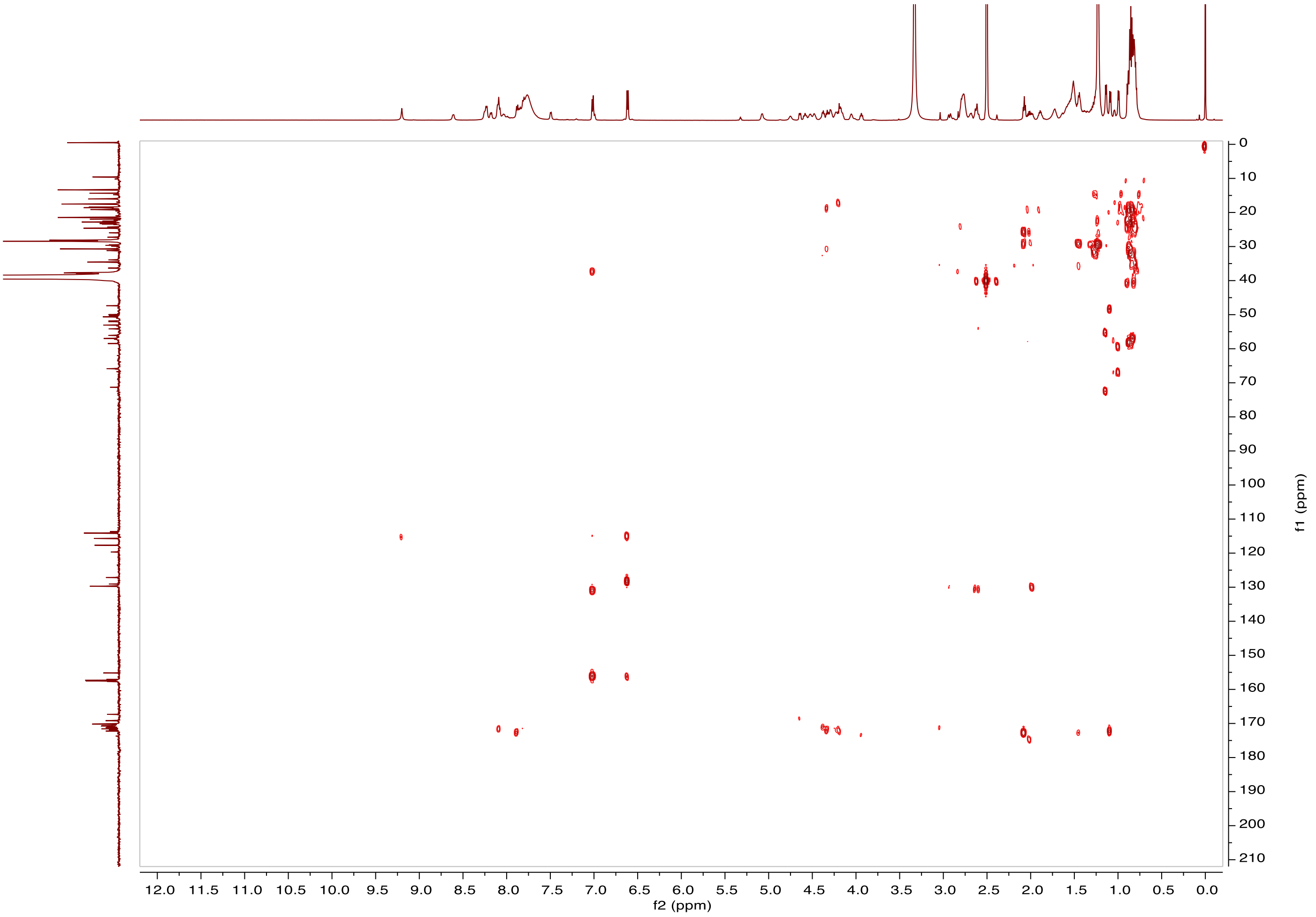

Figure S110. ^{1}H-^{13}C HMBC NMR spectrum of synAQU6-C4 in DMSO-d_6 (600 MHz)

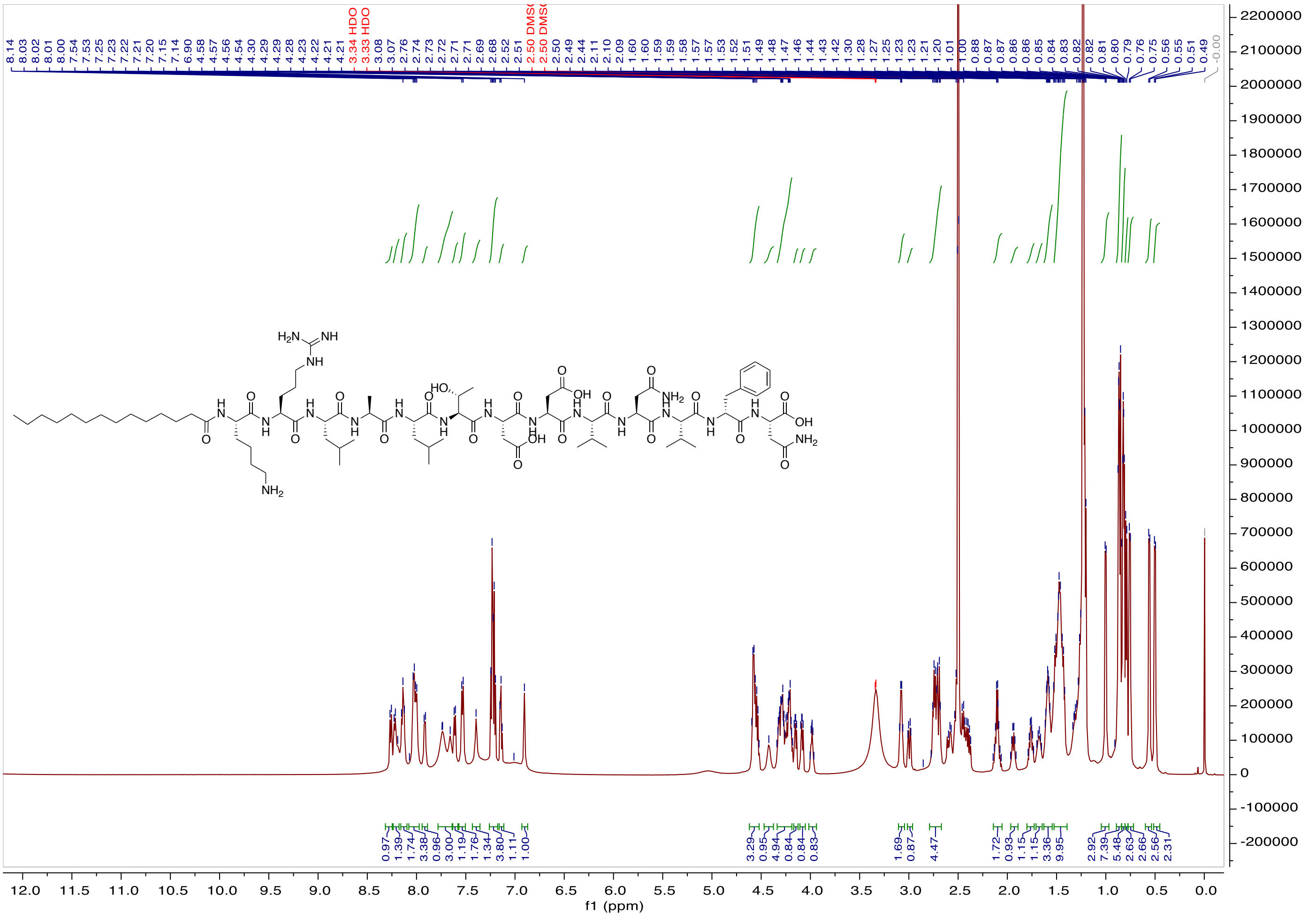

Figure S111. ^{1}H NMR spectrum of synAQU7-L in DMSO-d_6 (600 MHz)

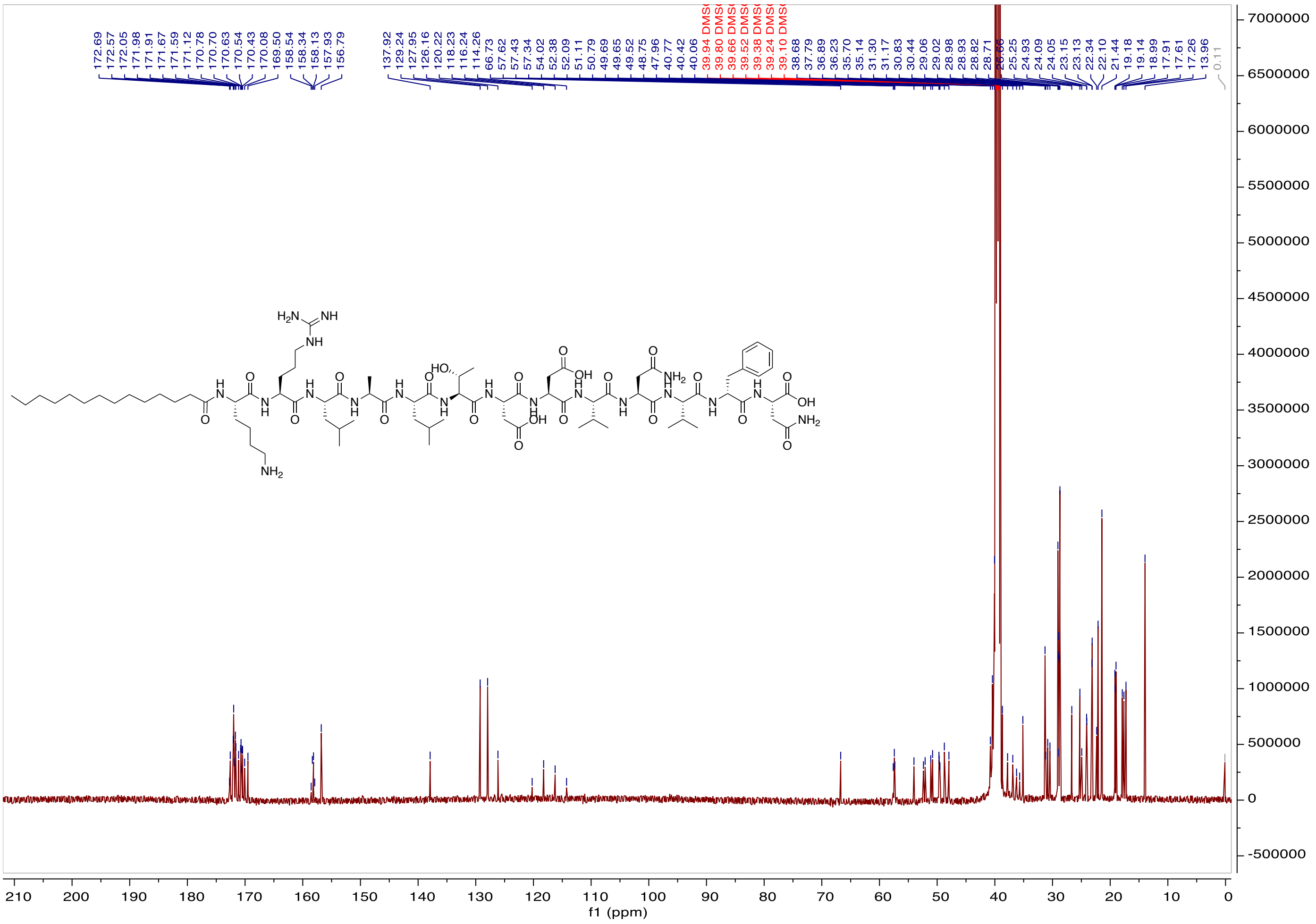

Figure S112. ^{13}C NMR spectrum of synAQU7-L in DMSO-d_6 (150 MHz)

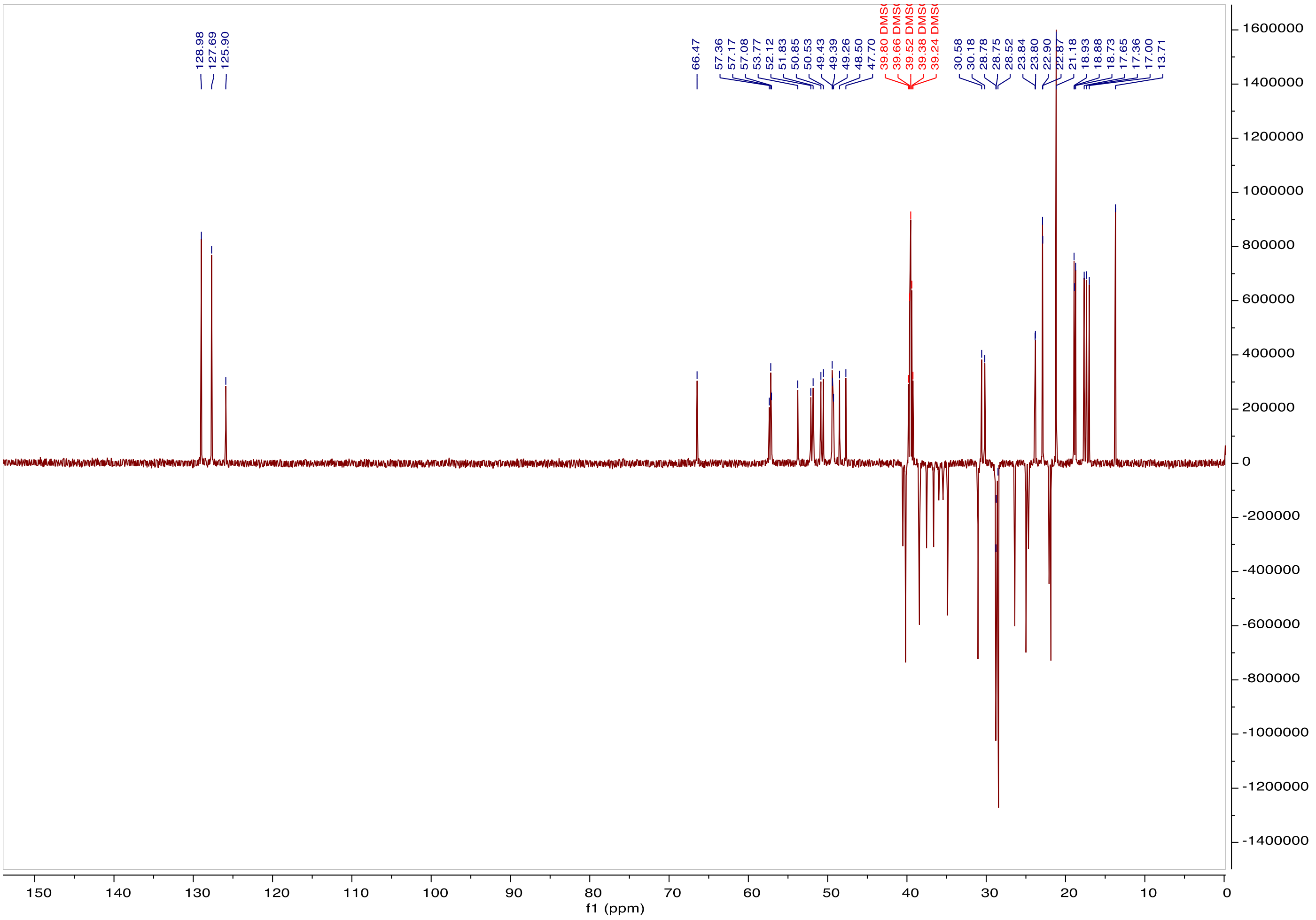

Figure S113. DEPT135 NMR spectrum of synAQU7-L in DMSO-d_6 (150 MHz)

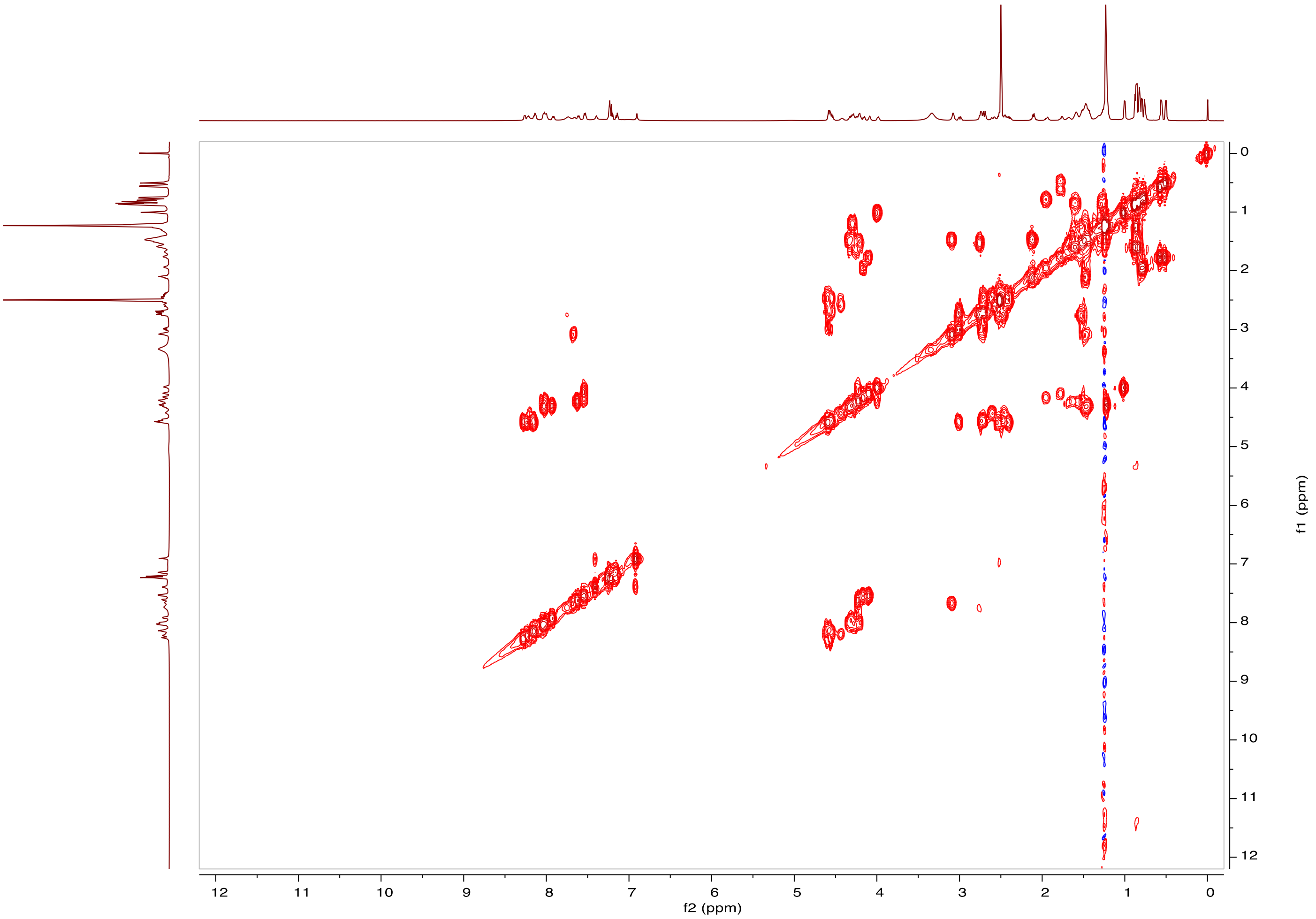

Figure S114. ^{1}H-^{1}H COSY NMR spectrum of synAQU7-L in DMSO-d_6 (600 MHz)

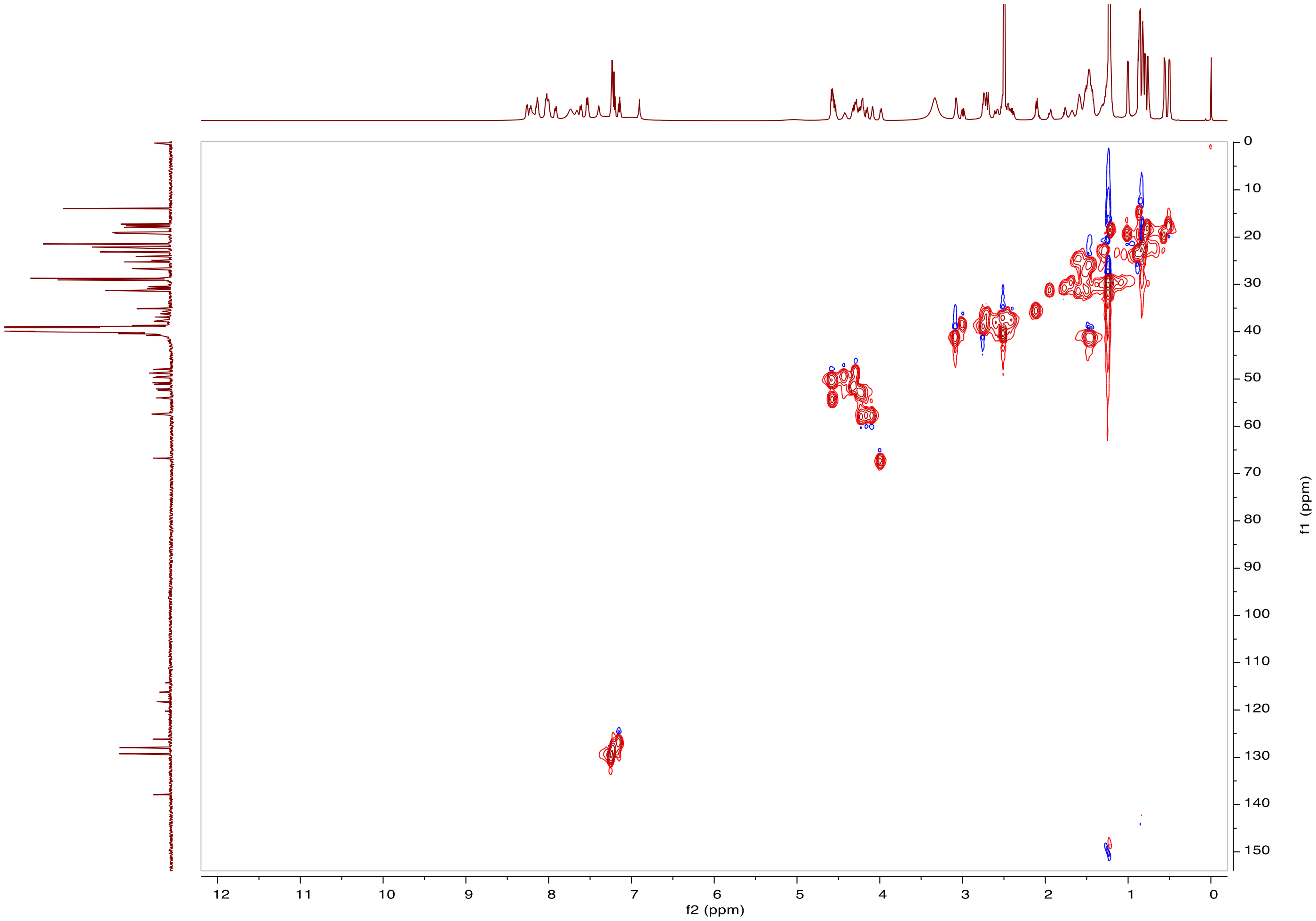

Figure S115. ^{1}H-^{13}C HSQC NMR spectrum of synAQU7-L in DMSO-d_6 (600 MHz)

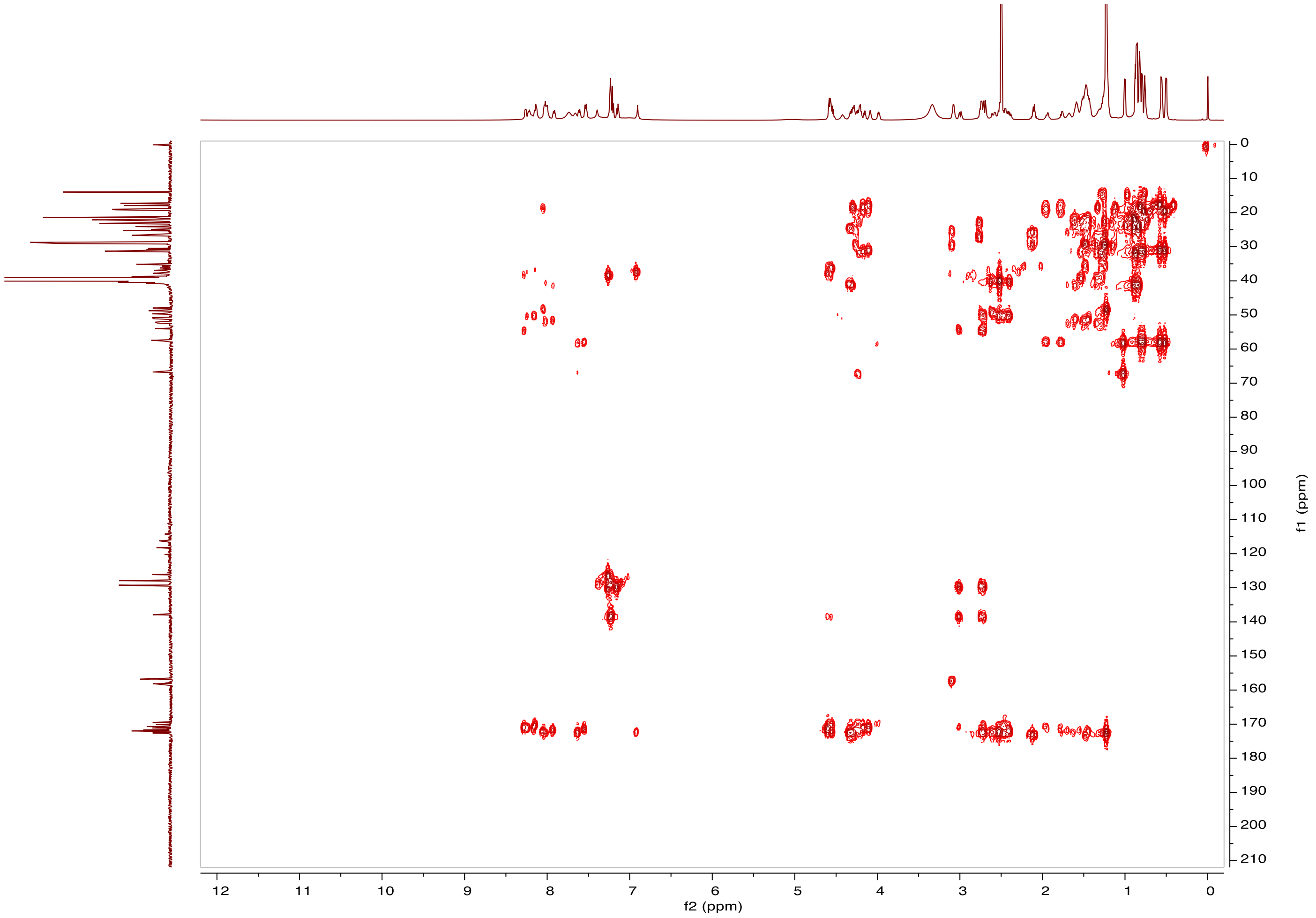

Figure S116. ^{1}H-^{13}C HSQC NMR spectrum of synAQU7-L in DMSO-d_6 (600 MHz)

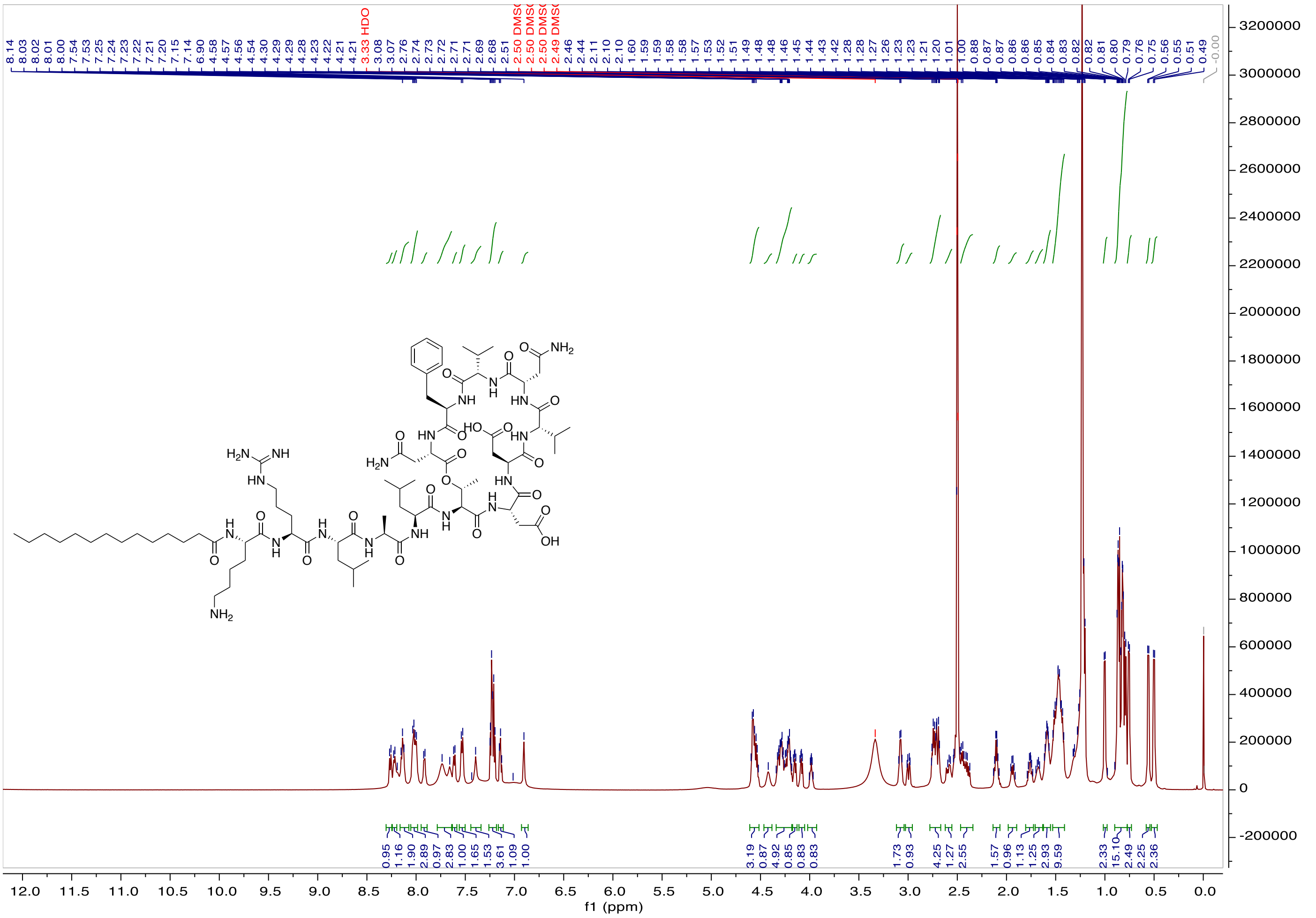

Figure S117. ^{1}H NMR spectrum of synAQU7-C6 in DMSO-d_6 (600 MHz)

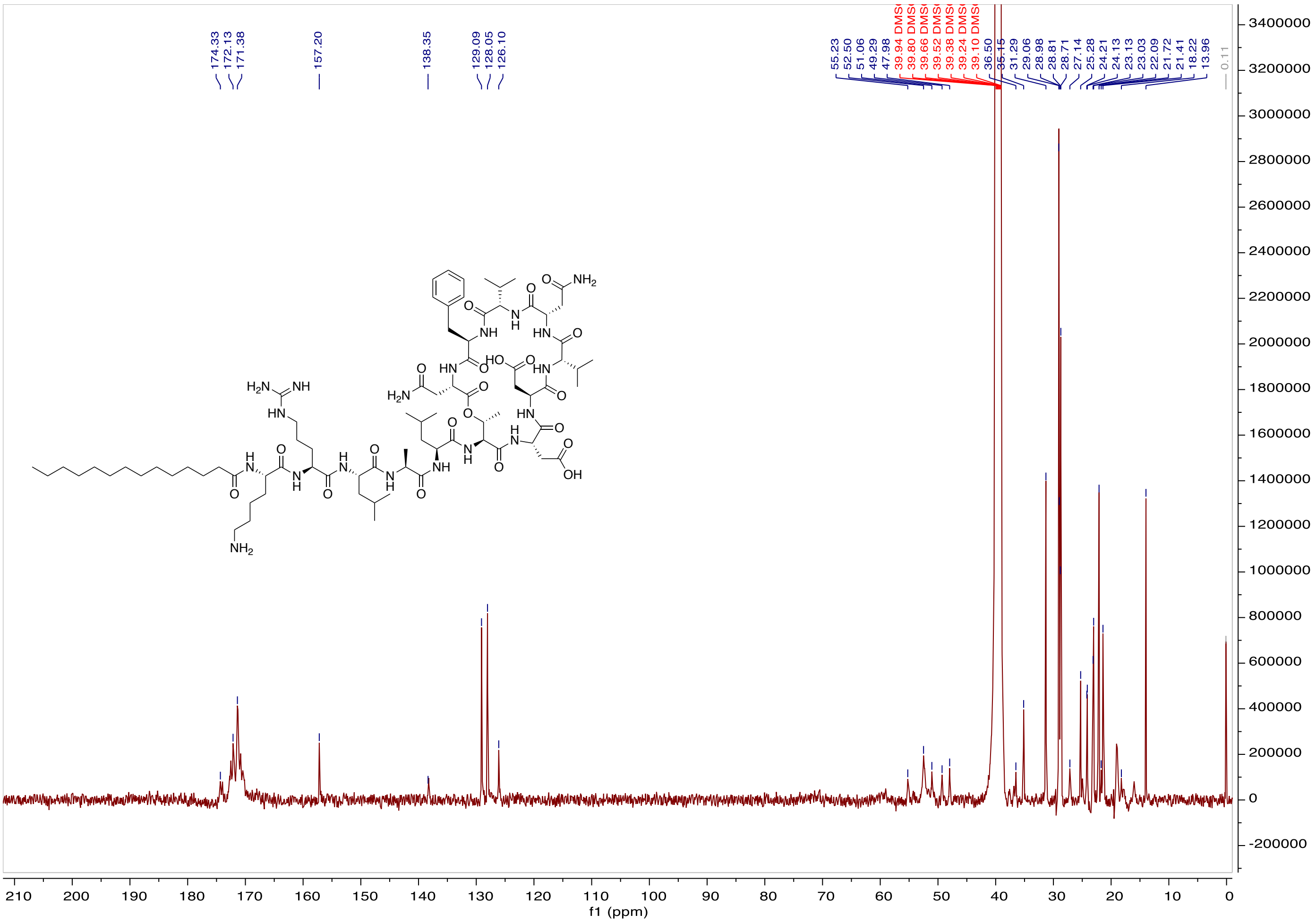

Figure S118. ^{13}C NMR spectrum of synAQU7-C6 in DMSO-d_6 (150 MHz)

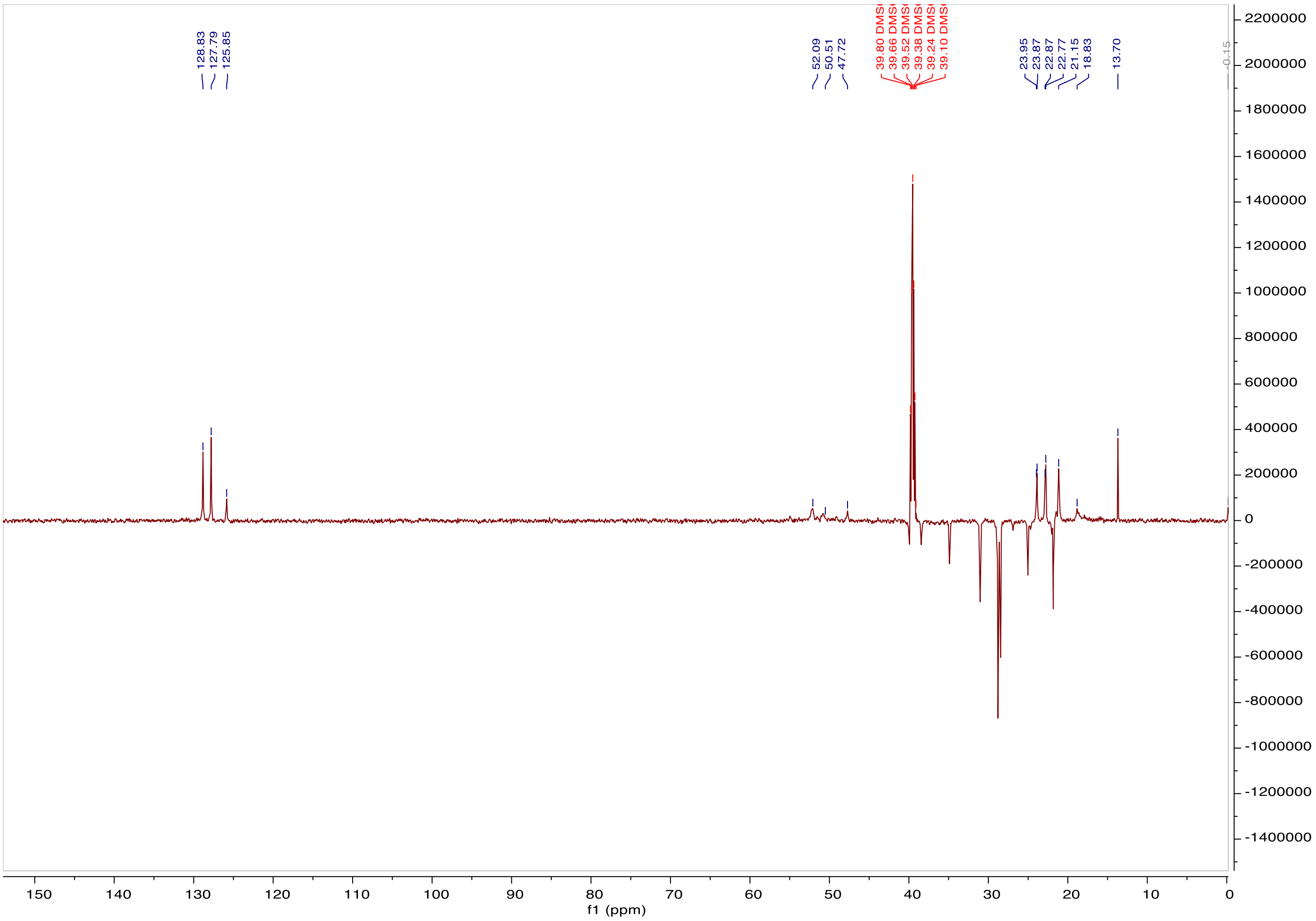

Figure S119. DEPT135 NMR spectrum of synAQU7-C6 in DMSO-d_6 (150 MHz)

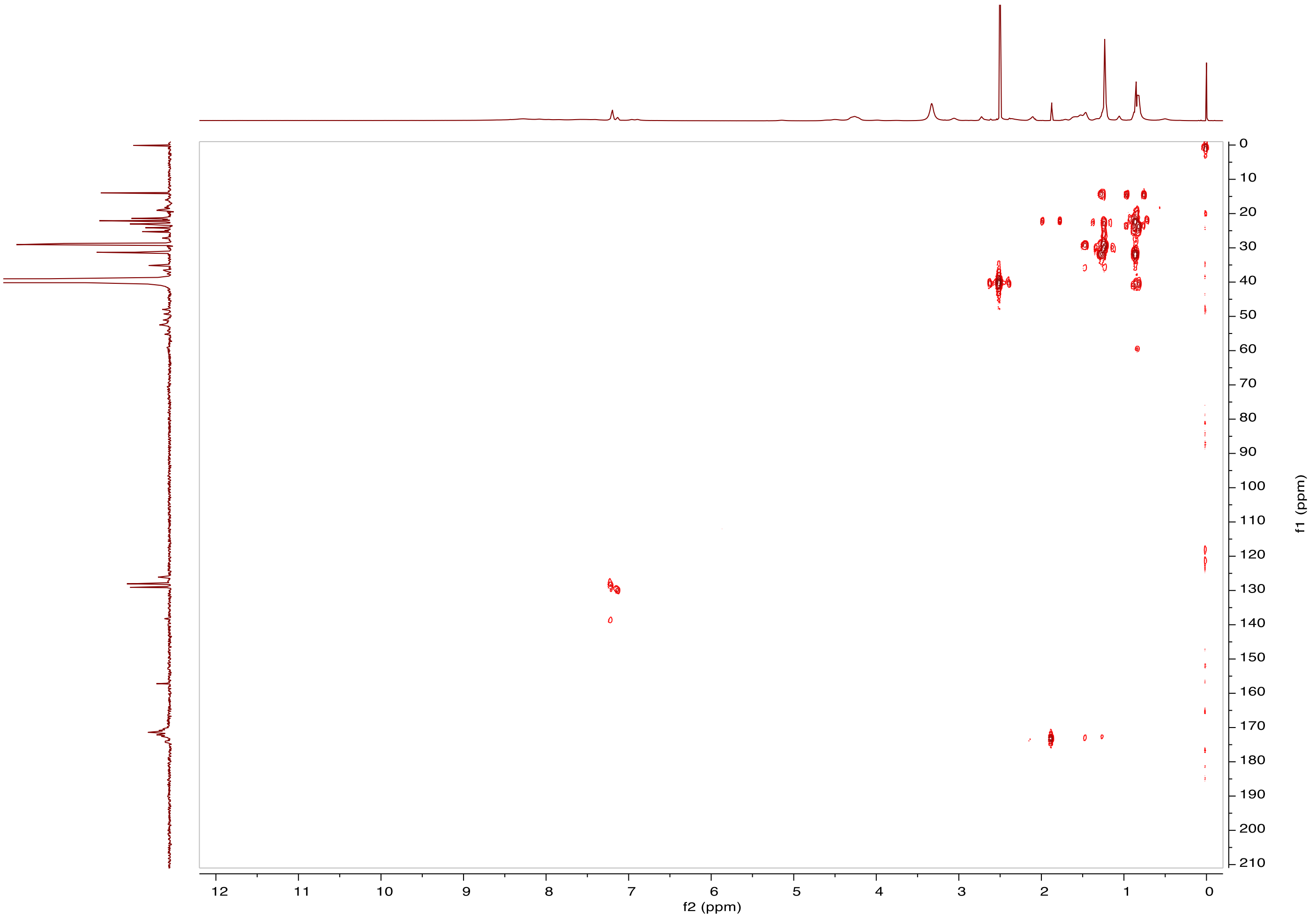

Figure S120. ^{1}H-^{1}H COSY NMR spectrum of synAQU7-C6 in DMSO-d_6 (600 MHz)

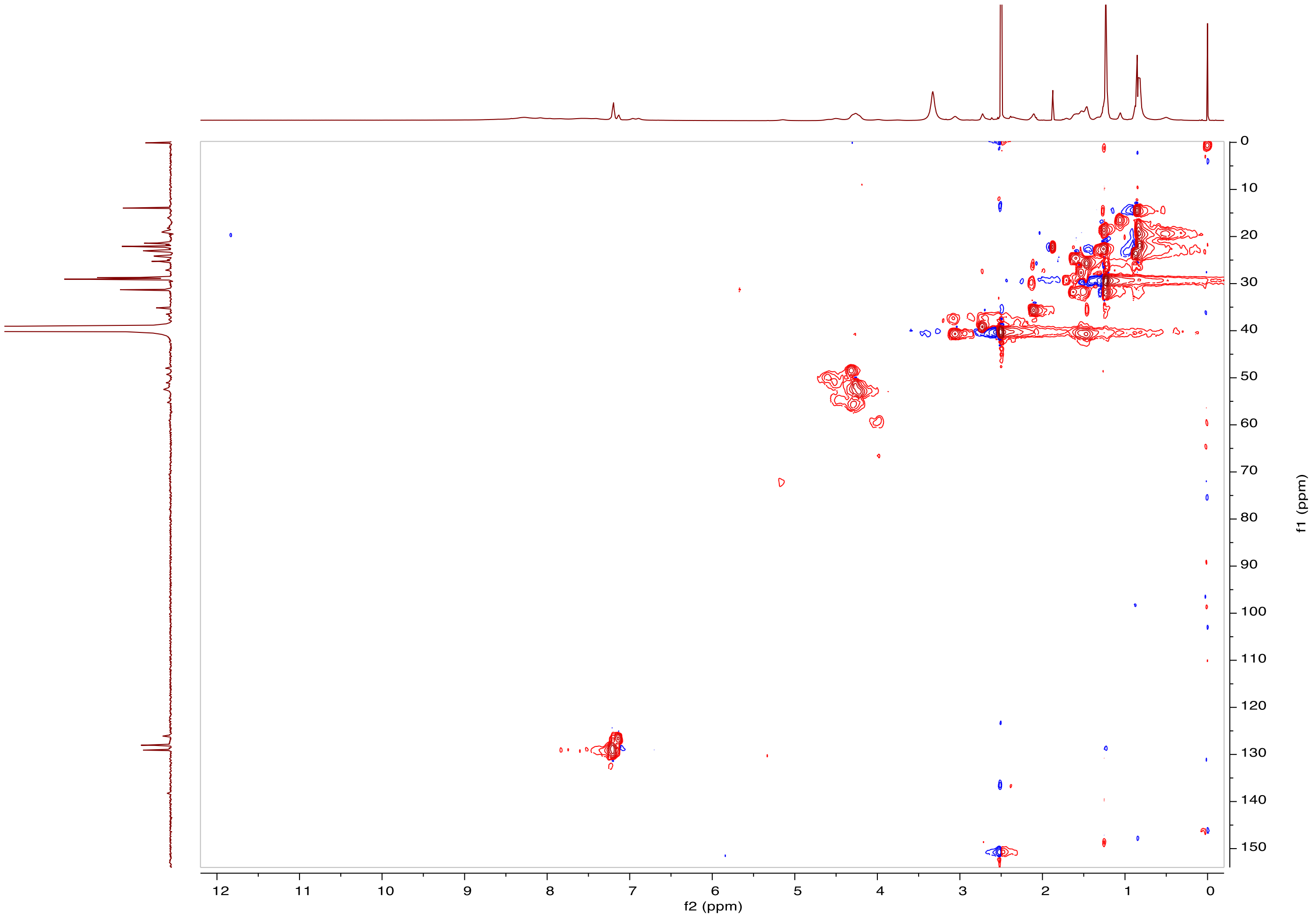

Figure S121. ^{1}H-^{13}C HSQC NMR spectrum of synAQU7-C6 in DMSO-d_6 (600 MHz)

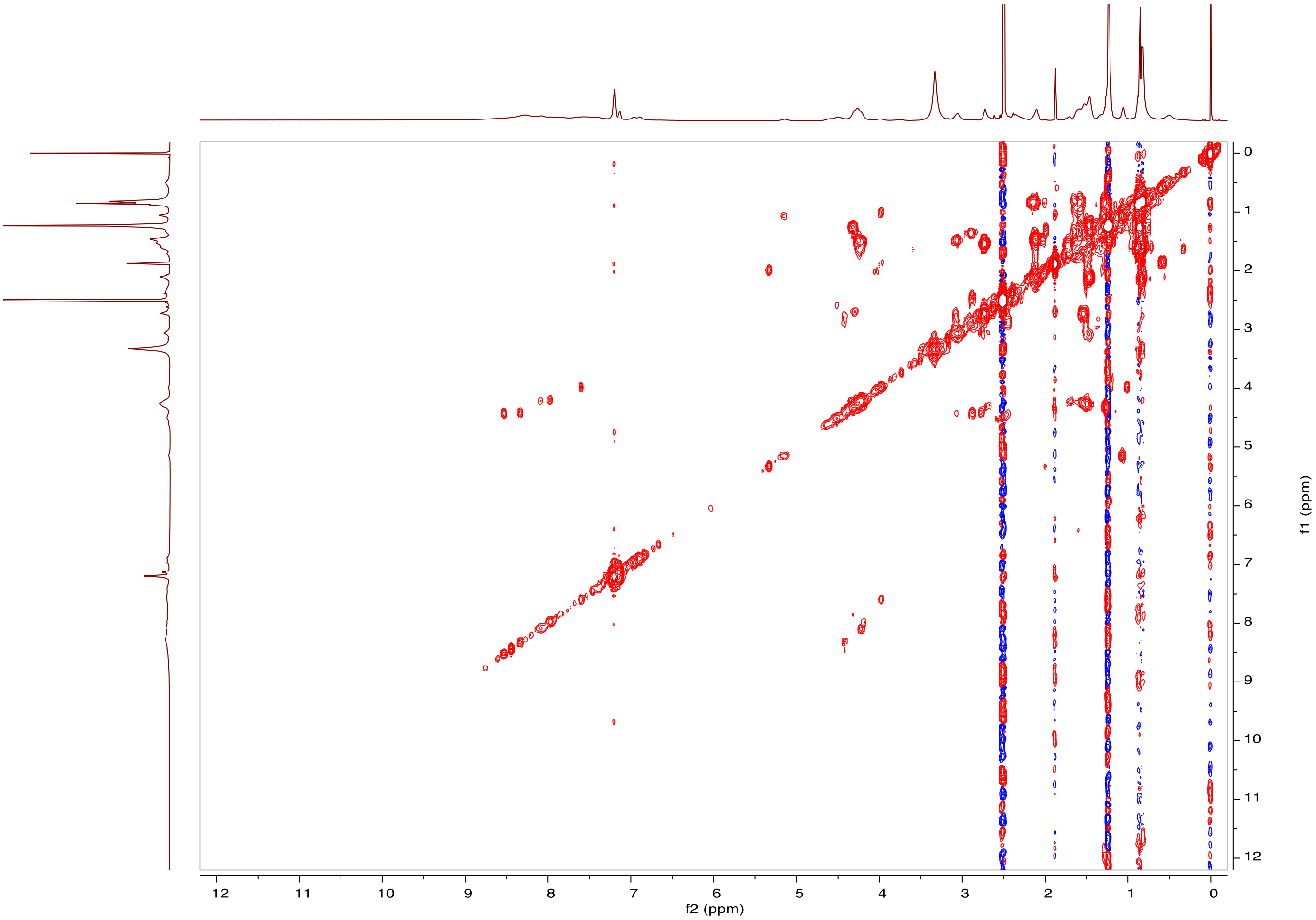

Figure S122. ^{1}H-^{13}C HMBC NMR spectrum of synAQUL-C6 in DMSO-d_6 (600 MHz)

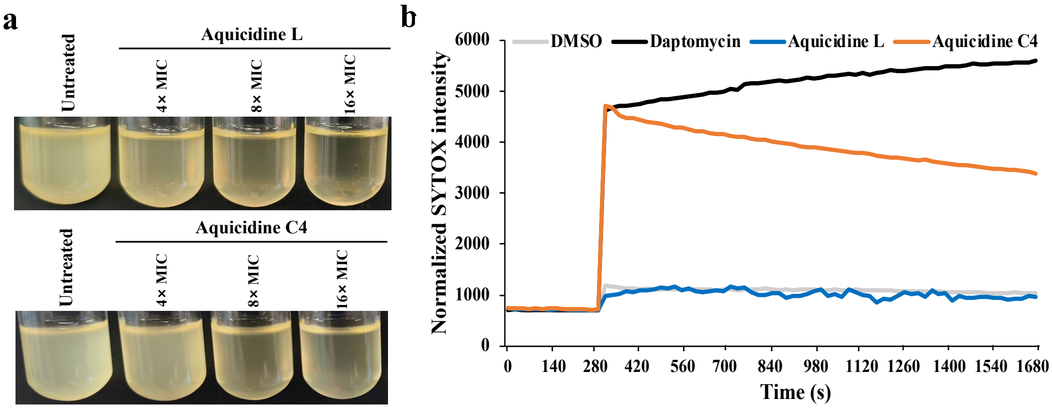

Figure S123. The bactericidal effects against *E. coli* DH5α (a) and the membrane lysis effects on *S. aureus* USA300 (b) of aquicidine L and C4. The results about bactericidal effects against *E. coli* DH5α were recorded after 12 h of aquicidine L or C4 treatment.

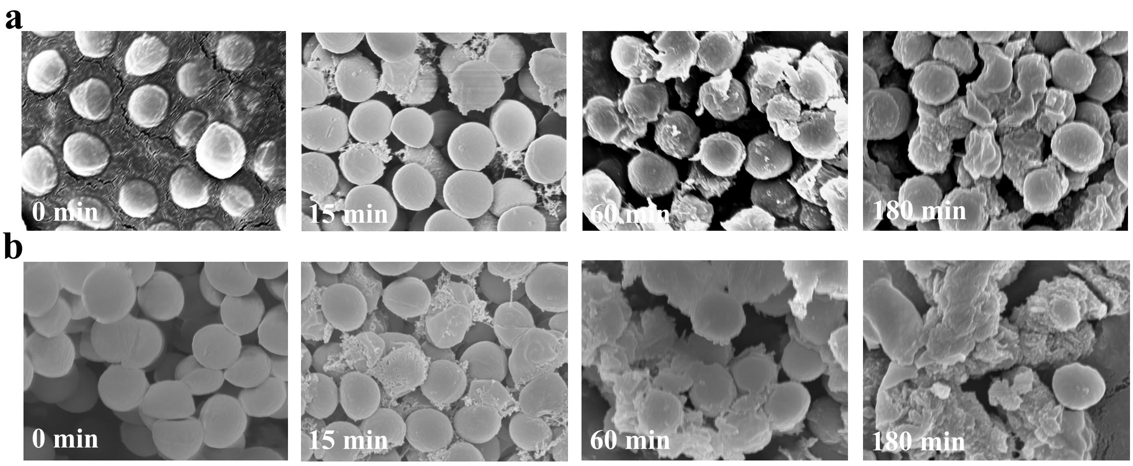

Figure S124. Scanning electron microscopy images of *S. aureus* USA300 cultures treated with aquicidine L or C4. a, Scanning electron microscopy images of *S. aureus* USA300 cultures treated with aquicidine L. b, Scanning electron microscopy images of *S. aureus* USA300 cultures treated with aquicidine C4. The images were captured for *S. aureus* USA300 after timed exposure to 4× the MIC of aquicidine L or C4.

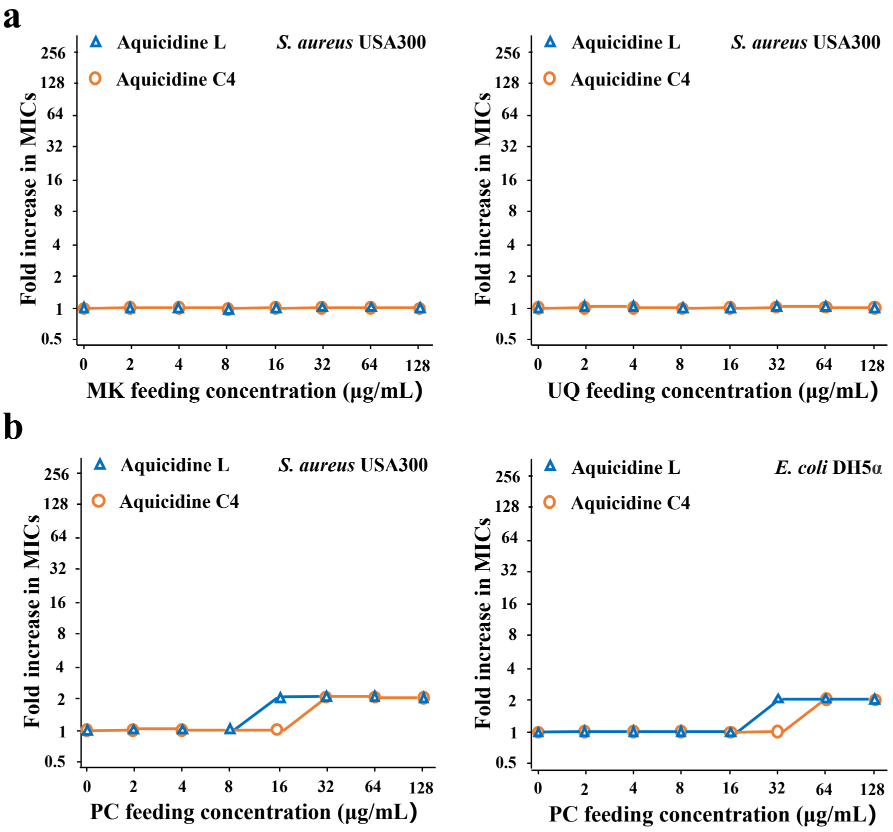

Figure S125. The antibacterial activities of aquicidine L and aquicidine C4 in the presence of MK, UQ or PC. a, The antibacterial activities of aquicidine L and aquicidine C4 against *S. aureus* USA300 in the presence of MK or UQ; b, The antibacterial activities of aquicidine L and aquicidine C4 against *S. aureus* USA300 or *E. coli* DH5α in the presence of PC.

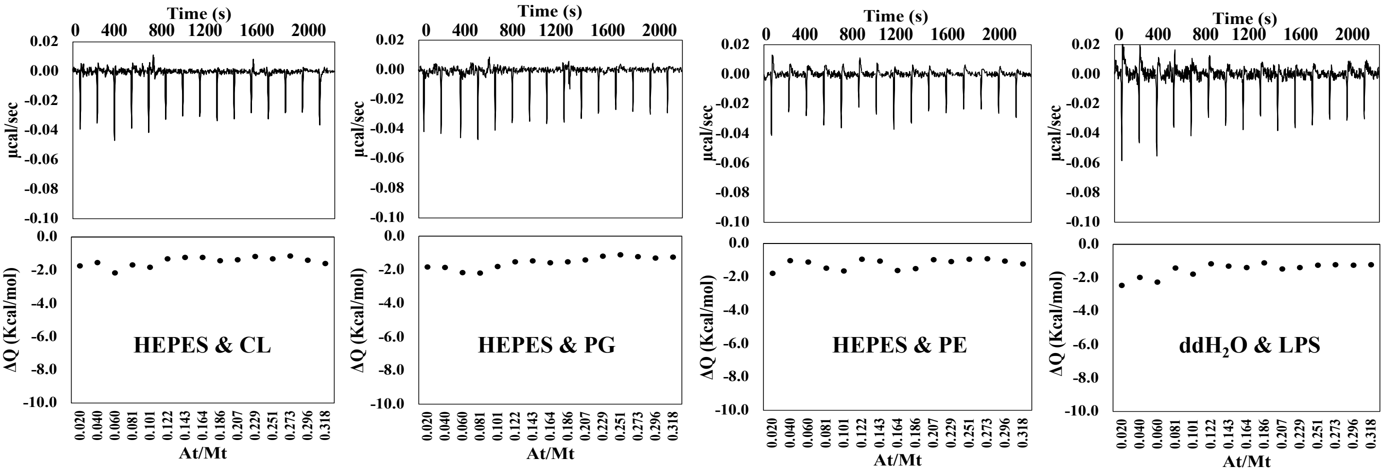

Figure S126. Isothermal titration of CL, PG, LPS or PE into the buffer (20 mM HEPES buffer or ddH_2O)

a

	Colistin	Aquicidine L
K. pneumoniae **CRKP 5452**	**0.125**	**4**
K. pneumoniae **CRKP 5452 PhoQ^{L348Q}**	**4**	**16**
K. pneumoniae **CRKP HS11286**	**0.125**	**4**
K. pneumoniae **NCTC 5056**	**32**	**8**
K. pneumoniae **15580**	**>64**	**8**

b

	Telomycin	Daptomycin	Aquicidine C4
S. aureus **USA300**	**4**	**1**	**4**
S. aureus **USA300 Cls2^{A338stop}**	**128**	**1**	**16**
S. aureus **USA300 MprF^{T345A}**	**4**	**16**	**16**

Figure S127. The antibacterial activities of aquicidine L or C4 against drug-resistant pathogens. a, The antibacterial activities of aquicidine L against colistin-resistant *K. pneumoniae* strains. The mutant *K. pneumoniae* CRKP 5452 PhoQ^{L348Q} was from the resistance acquisition experiment during serial passaging of *K. pneumoniae* CRKP5452 in the presence of sub-MIC levels of colistin. PhoQ encodes a sensor histidine kinase. The two strains *K. pneumoniae* NCTC 5056 and *K. pneumoniae* 15580 were from our collection and showed resistance to colistin. b, The antibacterial activities of aquicidine C4 against telomycin or daptomycin-resistant *S. aureus* strains. The two mutants *S. aureus* USA300 Cls2^{A388stop} and MprF^{T345A} were from the resistance acquisition experiment during serial passaging of *S. aureus* USA300 in the presence of sub-MIC levels of telomycin and daptomycin, respectively. Cls2 and MprF encode a cardiolipin synthase and a phospholipid flippase, respectively.

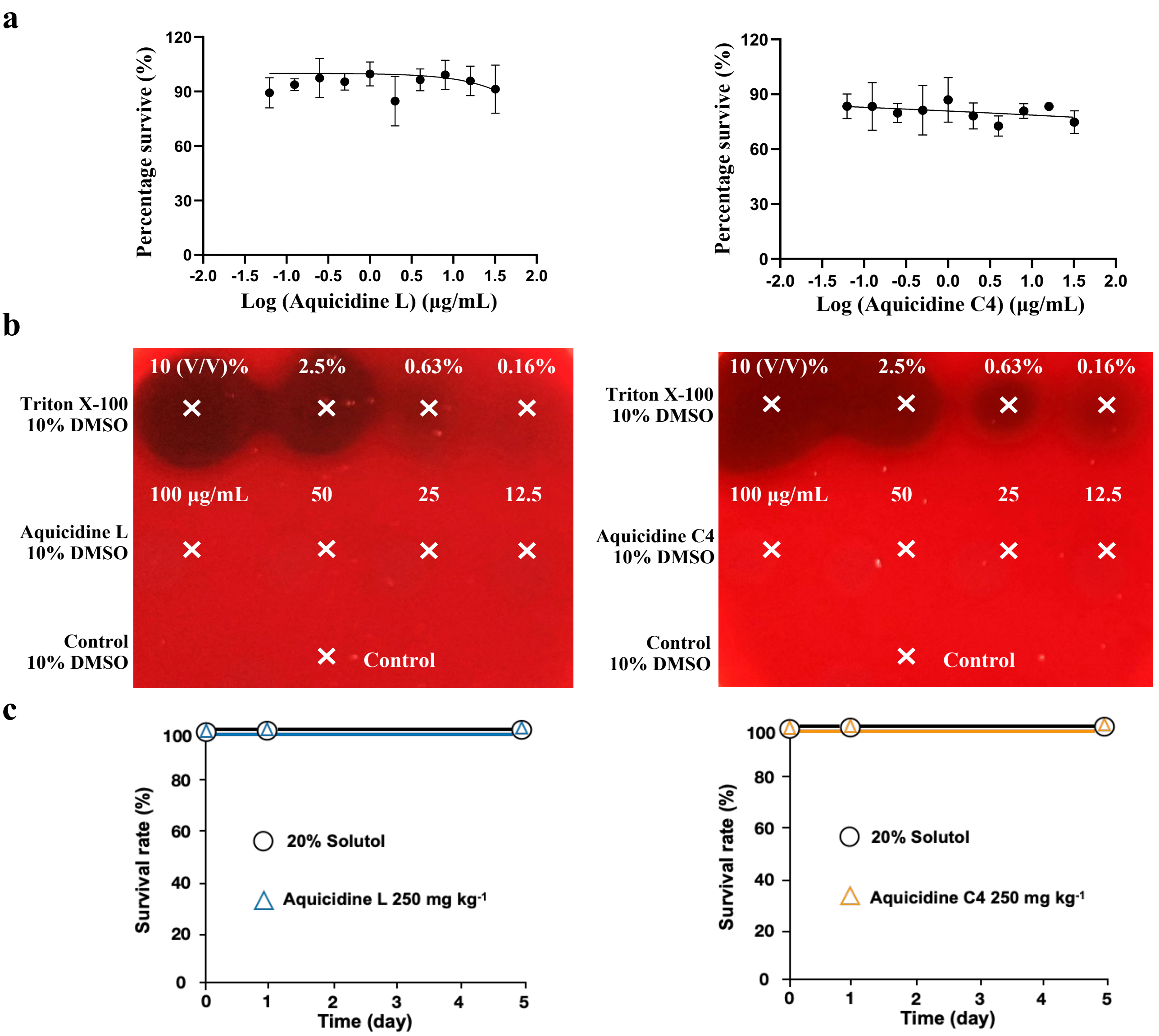

Figure S128. Cytotoxicities, haemolytic activities and *in vivo* acute toxicities of aquicidine L and aquicidine C4. a, Cytotoxicities aginast the human cell line HeLa of aquicidine L and aquicidine C4; b, Haemolytic activities of aquicidine L and aquicidine C4; c, Acute toxicities of aquicidine L and aquicidine C4 in mouse.

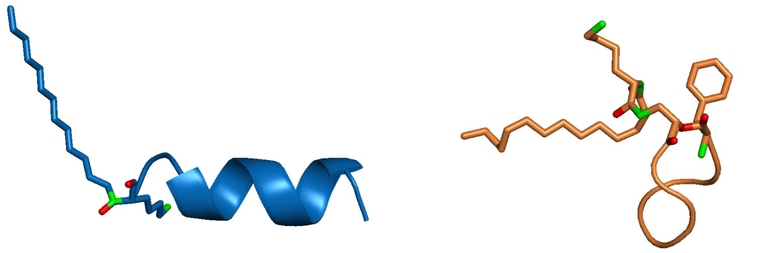

Aquicidine L (Lys-substituted) **Aquicidine C4 (Lys-substituted)**

Figure S129. Predicted secondary structures of aquicidine L (Lys-substituted) and aquicidine C4 (Lys-substituted)
